# HK1 from hepatic stellate cell–derived extracellular vesicles promotes progression of hepatocellular carcinoma

**DOI:** 10.1038/s42255-022-00642-5

**Published:** 2022-10-03

**Authors:** Qi-tao Chen, Zhi-yuan Zhang, Qiao-ling Huang, Hang-zi Chen, Wen-bin Hong, Tianwei Lin, Wen-xiu Zhao, Xiao-min Wang, Cui-yu Ju, Liu-zheng Wu, Ya-ying Huang, Pei-pei Hou, Wei-jia Wang, Dawang Zhou, Xianming Deng, Qiao Wu

**Affiliations:** 1 State Key Laboratory of Cellular Stress Biology, Innovation Center for Cell Biology, School of Life Sciences, Xiamen University, Xiamen, China; 2 Fujian Provincial Key Laboratory of Chronic Liver Disease and Hepatocellular Carcinoma, Xiamen University Affiliated ZhongShan Hospital, Xiamen, China

**Keywords:** Cancer microenvironment, Cancer metabolism, Metabolism, Protein translocation

## Abstract

Extracellular vesicles play crucial roles in intercellular communication in the tumor microenvironment. Here we demonstrate that in hepatic fibrosis, TGF-β stimulates the palmitoylation of hexokinase 1 (HK1) in hepatic stellate cells (HSCs), which facilitates the secretion of HK1 via large extracellular vesicles in a TSG101-dependent manner. The large extracellular vesicle HK1 is hijacked by hepatocellular carcinoma (HCC) cells, leading to accelerated glycolysis and HCC progression. In HSCs, the nuclear receptor Nur77 transcriptionally activates the expression of depalmitoylase ABHD17B to inhibit HK1 palmitoylation, consequently attenuating HK1 release. However, TGF-β-activated Akt functionally represses Nur77 by inducing Nur77 phosphorylation and degradation. We identify the small molecule PDNPA that binds Nur77 to generate steric hindrance to block Akt targeting, thereby disrupting Akt-mediated Nur77 degradation and preserving Nur77 inhibition of HK1 release. Together, this study demonstrates an overlooked function of HK1 in HCC upon its release from HSCs and highlights PDNPA as a candidate compound for inhibiting HCC progression.

## Abstract (web summary)

Hexokinase 1 is found to be secreted from hepatic stellate cells in large extracellular vesicles in response to TGF-β, and is subsequently taken up by hepatocellular carcinoma cells, where it promotes tumor progression and metastasis.

## Main

HCC has been linked with tumor-associated stromal cells, especially HSCs, the major subtype of stromal cells involved in liver fibrosis^1,2^. Most HCC cases develop in the context of severe liver fibrosis or cirrhosis, and the fibrotic liver facilitates the establishment of a protumoral microenvironment. In the normal liver, HSCs maintain a nonproliferative and quiescent phenotype and account for ~10% of all resident liver cells. During hepatic fibrosis, constitutively activated HSCs transdifferentiate into myofibroblasts with the characteristic of enhanced extracellular matrix production^3^. Clinical data show that activated HSCs are associated with poor prognosis in patients with HCC and affect HCC development in multiple ways^4,5^. Although therapeutic strategies targeting HSCs are becoming increasingly attractive, further elucidation of the functional mechanisms by which activated HSCs facilitate HCC progression is needed.

Extracellular vesicles (EVs) are broadly classified into exosomes and ectosomes (also termed microvesicles), according to their different biogenesis^6,7^. Since there are no available methods to completely separate exosomes from ectosomes, the term ‘small extracellular vesicles’ (sEVs) has been suggested to refer to EVs less than 200 nm in diameter, and the term ‘large extracellular vesicles’ (lEVs) has been used to refer to EVs greater than 200 nm in diameter^8^. EVs are essential for HCC malignancy and serve as biomarkers for early HCC diagnosis^9,10^. Recently, we found that HCC cell–derived lEV PKM2 can reprogram glucose metabolism in monocytes, which promotes the differentiation of monocytes into tumor-associated macrophages, resulting in the malignant progression of HCC^11^. EVs released by HCC cells also contain various oncogenic microRNAs (OncomiRs) that transform HSCs into cancer-associated HSCs (caHSCs) and jointly establish a prometastatic tumor microenvironment^12^. Therefore, investigation of the EV-mediated crosstalk between HCC cells and HSCs is expected to further benefit clinical treatment.

Hexokinase (HK), the enzyme catalyzing the first committed step of glycolysis, is essential for glucose utilization. The HK family consists of four isoenzymes. HK1 is generally expressed in most adult tissues, whereas HK2 is highly expressed in many fetal tissues and cancer cells. HK3 is expressed at comparatively low levels in almost all tissues. HK4 is specifically expressed in the liver and pancreas^13^. To adapt to metabolic reprogramming, HCC cells express high levels of HK2 and consequently show a high affinity for glucose. Accordingly, many therapeutic strategies for HCC that selectively target HK2 have been exploited over the past few years. Recently, HK1 was suggested to act as an effector regulated by K-Ras4A to promote tumor metabolic reprogramming, and overexpression of HK1 was found to predict poor prognosis in colorectal cancer^14^. However, the functions of HK1 in HCC progression are unclear. Although HKs have also been found in some EVs^15,16^, their exact regulatory functions in EVs are largely unknown.

Transforming growth factor-β (TGF-β) regulates diverse processes during development and tissue homeostasis. As the most potent fibrogenic factor, the expression of TGF-β is markedly increased in the fibrotic liver to activate HSCs^17^. Here, we found that TGF-β activates Akt to induce the phosphorylation and degradation of the orphan nuclear receptor Nur77 (also called TR3 or NGFI-B) in HSCs, thereby impeding Nur77-mediated inhibition of HK1 palmitoylation and resulting in the secretion of HK1 via lEVs. This lEV HK1 is taken up by HCC cells to facilitate tumor growth through glycolytic reprogramming. *n*-pentyl 2-[3,5-dihydroxy-2-(1-nonanoyl)phenyl]acetate (PDNPA), a compound that binds Nur77 to block Akt-induced Nur77 degradation, impedes the release of HK1 via lEVs. These findings not only demonstrate novel mechanisms underlying the regulation and protumoral function of HSC-derived lEV HK1, but also provide a potential intervention strategy for HCC.

## Results

### TGF-β induces the secretion of lEV HK1 from HSCs during fibrosis

**Fig. 1:**
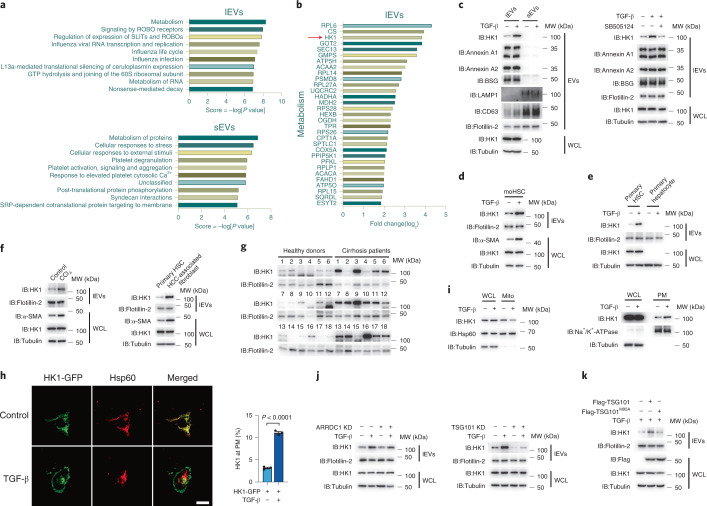
TGF-β induced secretion of lEV HK1 in hepatic fibrosis. For the experiments described in this figure, cells were treated with TGF-β (2 ng ml^−1^) for 36 h as indicated, after which cell lysates and lEVs or sEVs were prepared for the required experiments unless otherwise stated. **a**,**b**, Proteomics analysis of LX-2 cell–derived lEVs or sEVs by mass spectrometry. **a**, Pathway enrichment analysis (http://geneontology.org) of proteins under TGF-β treatment. **b**, List of proteins involved in metabolic pathways enriched in lEVs. **c**, Detection of TGF-β-induced HK1 secretion in lEVs and sEVs (left) and lEV HK1 secretion with or without SB505124 (5 μM) treatment (right). **d**,**e**, Detection of lEV HK1 secretion in immortalized moHSCs (**d**), and mouse primary HSCs and mouse primary hepatocytes (**e**). **f**, Left, comparison of HK1 secretion in primary HSCs isolated from control mice or mice with CCl_4_-induced hepatic fibrosis; right, comparison of HK1 secretion from primary HSCs and HCC-associated fibroblasts from mice with DEN/CCl_4_-induced hepatocarcinoma. **g**, HK1 levels shown in plasma lEVs derived from healthy donors and patients with liver cirrhosis (*n* = 18). **h**,**i**, HK1 localization determined by confocal microscopy (**h**; *n* = 3 independent experiments) or fractionation assays (**i**). Hsp60 and Na^+^/K^+^-ATPase were used to indicate mitochondria (Mito) and plasma membrane (PM), respectively. Scale bar, 10 μm. **j**, Detection of TGF-β-induced lEV HK1 in ARRDC1 knockdown (KD) or TSG101 KD cells. **k**, Comparison of lEV HK1 levels in TSG101-expressing and TSG101^M95A^-expressing cells. Flotillin-2 was used as a loading control for EVs. Tubulin was used as a protein loading control. WCL, whole cell lysates. Statistical data are presented as the mean ± s.e.m. Statistical analysis (**h**) was determined by two-tailed Student’s *t*-test. All western blots were repeated three times and one of them is shown. Source data

**Extended Data Fig. 1:**
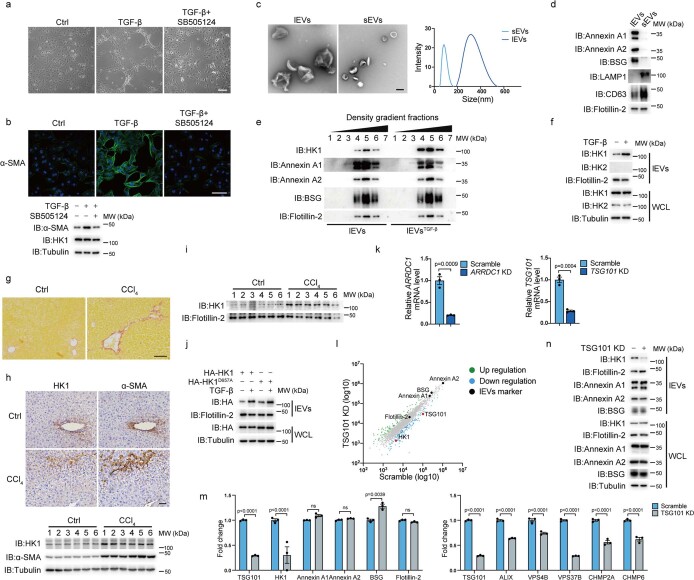
Large extracellular vesicle HK1 was secreted in a TSG101-dependent manner in hepatic fibrosis. **(a-b)** The myofibroblast-like morphology of LX-2 cells (a, 3 times experiments were repeated independently with similar results) and α-SMA expression level in LX-2 cells (b). Scale bar, 100 μm. **(c)** Analysis of the morphology (left) and size distribution (right) of LX-2 cell-derived lEVs or sEVs. Scale bar, 200 nm, 3 times experiments were repeated independently with similar results. **(d)** Analyses of lEVs and sEVs of LX-2 cells. **(e)** Density gradient fractionation of lEVs derived from LX-2 cells. After flotation of sample in iodixanol gradients, equal volumes of each fraction were loaded on SDS-PAGE gels. **(f)** TGF-β induced HK1 but not HK2 secretion in LX-2 cells. **(g-h)** CCl_4_ induced fibrosis in liver tissue from C57BL/6 mice, as indicated by Sirius Red staining (f) and α-SMA expression, accompanied by elevated HK1 levels (g, n = 6 independent mice). Scale bar, 100 μm. **(i)** HK1 levels were detected in plasma lEVs from control and CCl_4_-induced liver fibrosis mice (n = 6 independent mice). **(j)** Effect of TGF-β on HK1 and HK1^D657A^ secretion in LX-2 cells. **(k)** Efficiency of *ARRDC1* or *TSG101* KD in LX-2 cells. (**l-m**) lEV proteins from control or TSG101 KD LX-2 cells were detected by label-free quantitative mass spectrometry, and shown in scatter plot (l) and histogram (m). **(n)** The levels of proteins as indicated were detected in control or TSG101 KD LX-2 cells. Flotillin-2 was used as a loading control for EVs. Tubulin was used as a protein loading control. WCL, whole cell lysates. Statistic data are presented as the mean ± SEM. Statistical analyses were determined by two-tailed Student’s t-test (k) and one-way ANOVA, followed by Tukey’s post hoc test (m). All western blots are repeated three times and one of them is shown. Source data

TGF-β has been reported to induce fibrotic responses in different cell types^18^. Here, we showed that TGF-β induced LX-2 HSCs to adopt a myofibroblast-like cell phenotype (Extended Data Fig. 1a); the expression of α-smooth muscle actin (α-SMA), a typical marker of fibrosis, was substantially enhanced by TGF-β but totally blocked by SB505124, an inhibitor of the TGF-β receptor (Extended Data Fig. 1b). However, whether the fibrotic process is linked to protein secretion has not been reported. To study intercellular communication during fibrosis, we separately extracted lEVs and sEVs from LX-2 cells treated with or without TGF-β by differential centrifugation. Visualization via transmission electron microscopy showed appearances typical of these types of vesicles (Extended Data Fig. 1c, left). The diameter of sEVs was approximately 100 nm, whereas the diameter of the lEVs ranged from 200 nm to 600 nm (Extended Data Fig. 1c, right). Annexin A1, annexin A2 and BSG (markers of ectosomes^19–21^) were enriched in the lEVs, and CD63 and LAMP1 (markers of exosomes^19,20^) were enriched in the sEVs (Extended Data Fig. 1d), indicating that ectosomes were mainly present in the lEV fraction, and exosomes were enriched in sEVs. We then performed label-free quantitative proteomic analysis to study TGF-β-induced protein variations in these EVs. Gene Ontology (GO) enrichment analysis showed the TGF-β-induced protein changes in lEVs and sEVs; among the significantly enriched pathways, the metabolic pathway was the most enriched in lEVs but not in sEVs (Fig. 1a), and HK1 was found to be one of the most enriched proteins in lEVs upon TGF-β stimulation (Fig. 1b). Given that glycolytic reprogramming is an important feature of liver fibrosis^22^, we focused on the function of TGF-β-induced lEV HK1 secretion.

In LX-2 cells, TGF-β stimulation markedly increased the HK1 level in lEVs, which could be blocked by SB505124 (Fig. 1c). A similar result was also observed in an immortalized mouse HSC line (moHSCs) (Fig. 1d). Since differential centrifugation may result in co-isolation of non-EV components such as protein aggregates^19^, we also performed an iodixanol-based density gradient fractionation after ultracentrifugation to further increase EV purity^19,23^. Again, the results showed that TGF-β clearly elevated the protein level of HK1 in the fractions that were highly enriched for classical lEV markers (Extended Data Fig. 1e). Furthermore, HK1 secretion via lEVs was enhanced by TGF-β stimulation in mouse primary HSCs but not in mouse primary hepatocytes (Fig. 1e). Although HK2 was also expressed in LX-2 cells, it could not be detected in lEVs even in the presence of TGF-β (Extended Data Fig. 1f). Hence, these results suggest the specific secretion of HK1 from HSCs through lEVs.

To gain insight into the secretion of lEV HK1 in vivo, a mouse model of carbon tetrachloride (CCl_4_)-induced hepatic fibrosis was employed. During hepatic fibrosis, as revealed by Sirius red staining (Extended Data Fig. 1g), the number of α-SMA-positive cells was clearly increased (Extended Data Fig. 1h), indicating the expansion of activated HSCs. This HSC expansion was associated with coexpression of HK1 in α-SMA-positive HSCs (Extended Data Fig. 1h). Although the HK1 expression levels in mouse primary HSCs from control mice and mice with CCl_4_-induced hepatic fibrosis were almost the same in whole cell lysates, the secretion of HK1 via lEVs was enhanced in CCl_4_-treated mice (Fig. 1f, left). Cancer-associated fibroblasts (CAFs), which mainly derived from HSCs in HCC, also secreted more HK1 than quiescent HSCs (Fig. 1f, right). In clinical samples, the levels of HK1 were much higher in plasma lEVs isolated from patients with liver cirrhosis than those from healthy donors (Fig. 1g). Similar phenomena were also observed in plasma lEVs from fibrotic mice (Extended Data Fig. 1i). Collectively, liver fibrosis is accompanied by the secretion of HK1 via lEVs.

In LX-2 HSCs, TGF-β treatment promoted HK1 translocation from mitochondria to the plasma membrane (Fig. 1h,i), consistent with the observation that plasma membrane localization is a prerequisite for the sorting of proteins into lEVs^6^. Secretion of HK1 via lEVs was unrelated to HK1 enzyme activity, as the enzyme-dead mutant of HK1 (D657A)^24^ was still observed in lEVs upon TGF-β stimulation (Extended Data Fig. 1j). Arrestin domain–containing protein 1 (ARRDC1) and tumor susceptibility gene 101 (TSG101) are important for directing plasma membrane budding to form lEVs^25^. We found that TSG101, not ARRDC1, was required for TGF-β-induced secretion of HK1 (Fig. 1j and Extended Data Fig. 1k). Overexpression of TSG101 but not the TSG101^M95A^ mutant, which abolished the interaction of the TSG101 with an adaptor protein^26^, obviously increased the secretion of lEV HK1 (Fig. 1k). To further demonstrate the specific effect of TSG101 on HK1 secretion, we collected lEVs from control and TSG101 knockdown LX-2 cells, and proteomic analysis of these fractions (Extended Data Fig. 1l) revealed that knockdown of TSG101 markedly decreased the level of HK1 but did not reduce the levels of flotillin-2, annexin A1, annexin A2 or BSG (Extended Data Fig. 1m, left). Similar results were also found by western blotting (Extended Data Fig. 1n), suggesting that TSG101 has no obvious effect on the amount of released lEVs. As a positive control, the secretion of several members of the ESCRT complex that have been reported to assemble sorting complex together with TSG101, such as ALIX, VPS4B, VPS37B, CHMP2A and CHMP6^27^, were impaired upon TSG101 knockdown (Extended Data Fig. 1m, right). Together, these findings indicate that TGF-β induces the translocation of mitochondrial HK1 to the plasma membrane, in which TSG101 further assists the secretion of HK1 via lEVs.

### HK1 palmitoylation facilitates HK1 targeting to the plasma membrane for secretion

**Fig. 2:**
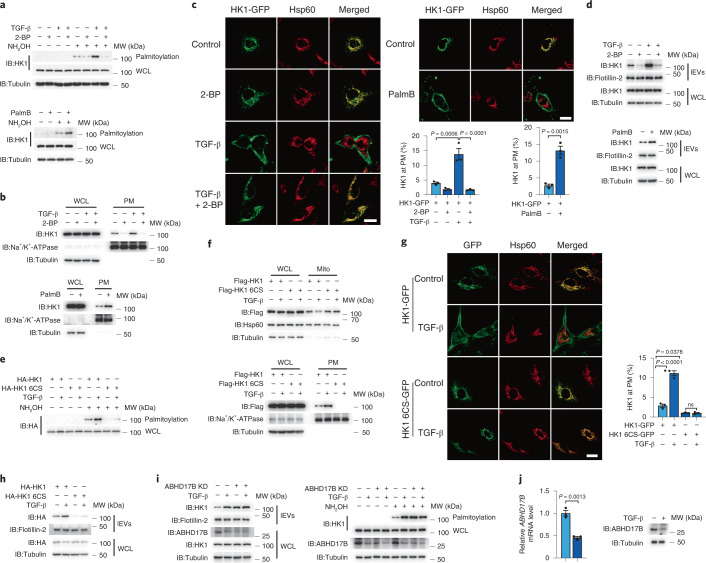
TGF-β-induced palmitoylation promotes HK1 translocation to the plasma membrane. For the experiments described in this figure, LX-2 cells were treated with TGF-β (2 ng ml^−1^) for 36 h as indicated, and cell lysates and lEVs were prepared for western blotting unless specifically indicated otherwise. **a**, Detection of HK1 palmitoylation in the presence of 2-BP (100 μM) (top) or PalmB (50 μM) (bottom) with or without TGF-β treatment. **b**,**c**, Effect of 2-BP and PalmB on HK1 translocation, analyzed by fractionation assays (**b**) and confocal microscopy (**c**; *n* = 3 independent experiments). Scale bar, 10 μm. **d**, Effect of 2-BP and PalmB on HK1 secretion. **e**–**h**, Comparison of HK1 palmitoylation (**e**), localization (**f**, **g**; *n* = 3 independent experiments) and secretion (**h**) in HK1-expressing and HK1 6CS-expressing LX-2 cells. Scale bar, 10 μm. **i**, Detection of HK1 palmitoylation (right) and secretion (left) in ABHD17B knockdown cells. **j**, Effect of TGF-β on *ABHD17B* mRNA and protein expression levels (*n* = 3 independent experiments). Flotillin-2 was used as a loading control for EVs. Tubulin was used as a protein loading control. Statistical data are presented as the mean ± s.e.m. Statistical analyses were determined by two-tailed Student’s *t*-test (**c**, right; **j**) and one-way ANOVA, followed by Tukey’s post hoc test (**c**, left; **g**). All western blots were repeated three times and one of them is shown. Source data

**Extended Data Fig. 2:**
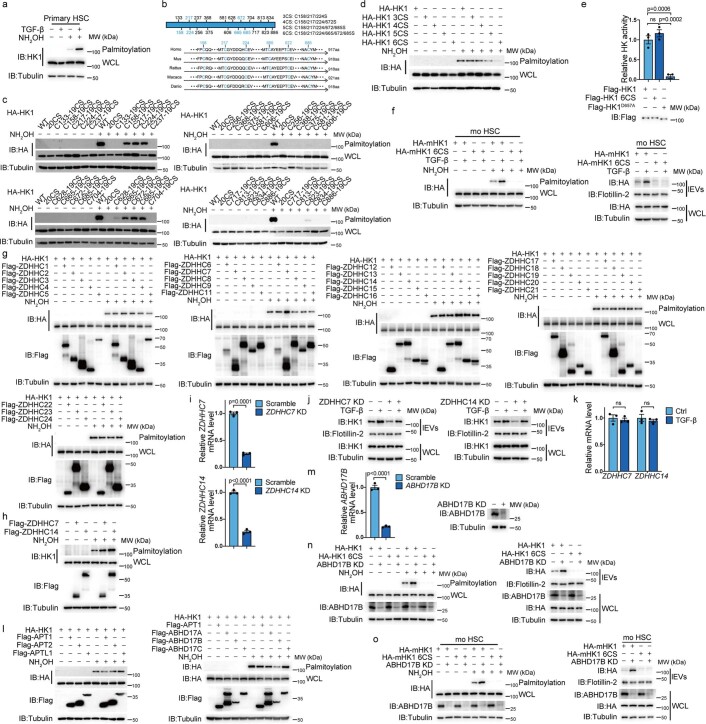
Palmitoylation of HK1 is required for its secretion. **(a)** Palmitoylation of HK1 was detected in primary HSCs. **(b)** Distribution of twenty cysteine residues in the HK1 monomer, the combination of different palmitoylation point mutants (top), and comparison of HK1 sequences in different species (bottom). **(c)** Comparison of palmitoylation of HK1 and related single-point HK1 rescue mutants based on HK1 20CS in 293T. **(d)** Comparison of palmitoylation of HK1 and HK1 mutants in LX-2. **(e)** Comparison of HK1, HK1 6CS and HK1^D657A^ enzyme activity. **(f)** Comparison palmitoylation and secretion of mouse HK1 and HK1 6CS. **(g-h)** Effect of different ZDHHC palmitoyltransferases on HK1 palmitoylation. Different palmitoyltransferases and HK1 were transfected into 293T cells (g) or LX-2 cells (h) as indicated, and HK1 palmitoylation was detected. **(i)** Efficiency of *ZDHHC7* and *ZDHHC14* KD in LX-2 cells. **(j)** The KD of ZDHHC7 or ZDHHC14 did not abolish the TGF-β-induced secretion of lEV HK1 in LX-2 cells. **(k)** TGF-β had no effect on the mRNA expression of *ZDHHC7* or *ZDHHC14* in LX-2 cells. **(l)** Effect of different depalmitoylases on HK1 palmitoylation. Different depalmitoylases and HK1 were transfected into 293T cells, and HK1 palmitoylation was detected. **(m)** Efficiency of ABHD17B KD in LX-2 cells. **(n-o)** Effect of ABHD17B on HK1 palmitoylation and secretion. HK1 and HK1 6CS were transfected into control or ABHD17B KD LX-2 cells (n) or immortalized mouse HSCs (o). Flotillin-2 was used as a loading control for EVs. Tubulin was used as a protein loading control. Statistic data are presented as the mean ± SEM. Statistical analyses were determined by two-tailed Student’s t-test (i, k, m) and one-way ANOVA, followed by Tukey’s post hoc test (e). All western blots are repeated three times and one of them is shown. Source data

The mechanism by which HK1 translocates to the plasma membrane for secretion was further investigated. Palmitoylation has been reported to assist protein targeting to the membrane^28^ and modulate protein cargo sorting into EVs^29^. Here, palmitoylated HK1 was detected in LX-2 cells and mouse primary HSCs, which was substantially enhanced by treatment with depalmitoylase inhibitor palmostatin B (PalmB) or TGF-β (Fig. 2a and Extended Data Fig. 2a). In contrast, treatment with palmitoylation inhibitor 2-bromopalmitate (2-BP) abolished HK1 palmitoylation (Fig. 2a, top). This modification was necessary and sufficient for the plasma membrane localization of HK1, as HK1 localization at the plasma membrane was blocked by 2-BP even in the presence of TGF-β but enhanced by PalmB even in the absence of TGF-β (Fig. 2b,c). Under these conditions, 2-BP inhibited and PalmB elevated lEV HK1 secretion (Fig. 2d). Hence, TGF-β-induced palmitoylation facilitates HK1 translocation from mitochondria to the plasma membrane for subsequent secretion via lEVs.

Palmitoylation usually occurs at cysteine (C) residues^28^, and the HK1 molecule contains 20 C residues (Extended Data Fig. 2b). When all 20 C residues were mutated to serine (S) (20CS), its palmitoylation was completely abolished (Extended Data Fig. 2c). Based on the 20CS mutant, the C mutations were rescued individually. Under these conditions, palmitoylation was found to occur mainly at six C residues: 158, 217, 224, 665, 672 and 685 (Extended Data Fig. 2c). Combined mutation of these candidate sites in HK1 demonstrated that palmitoylation was abolished only when all six C residues (HK1 6CS) were mutated (Extended Data Fig. 2d). Although HK1 6CS exhibited enzymatic activity comparable to that of wild-type HK1 (Extended Data Fig. 2e), it exhibited very little TGF-β-induced palmitoylation, plasma membrane localization, and secretion in LX-2 cells (Fig. 2e–h). These palmitoylated residues are conserved in several species (Extended Data Fig. 2b), and mutation of these sites in mouse HK1 also abolished TGF-β-induced palmitoylation and lEV HK1 secretion (Extended Data Fig. 2f). Hence, these six C residues in HK1 are critical for its palmitoylation, which controls HK1 targeting to the plasma membrane and subsequent secretion.

Palmitoylation is dynamically regulated by enzymes that catalyze palmitoylation or depalmitoylation^30^. To identify the enzymes involved in regulating HK1 palmitoylation, we individually transfected plasmids encoding 23 ZDHHC palmitoyltransferases into 293T cells. ZDHHC7 and ZDHHC14 likely activated HK1 palmitoylation in 293T cells (Extended Data Fig. 2g) and LX-2 cells (Extended Data Fig. 2h). However, knockdown of ZDHHC7 or ZDHHC14 could not abolish TGF-β-induced secretion of HK1 (Extended Data Fig. 2i,j), and TGF-β stimulation did not influence the mRNA levels of *ZDHHC7* and *ZDHHC14* (Extended Data Fig. 2k). Conversely, ABHD17B was the only depalmitoylase examined that markedly decreased HK1 palmitoylation (Extended Data Fig. 2l). When ABHD17B was knocked down (Extended Data Fig. 2m), the palmitoylation and secretion of HK1 but not HK1 6CS were enhanced in both LX-2 cells (Extended Data Fig. 2n) and immortalized mouse HSCs (Extended Data Fig. 2o), and TGF-β lost its ability to further induce the palmitoylation and secretion of endogenous HK1 (Fig. 2i). Since the expression of ABHD17B was suppressed by TGF-β (Fig. 2j), we concluded that during liver fibrosis, TGF-β facilitates HK1 secretion by inhibiting ABHD17B-dependent depalmitoylation of HK1.

### HCC cells hijack HSC-derived lEV HK1 to accelerate glycolysis

**Fig. 3:**
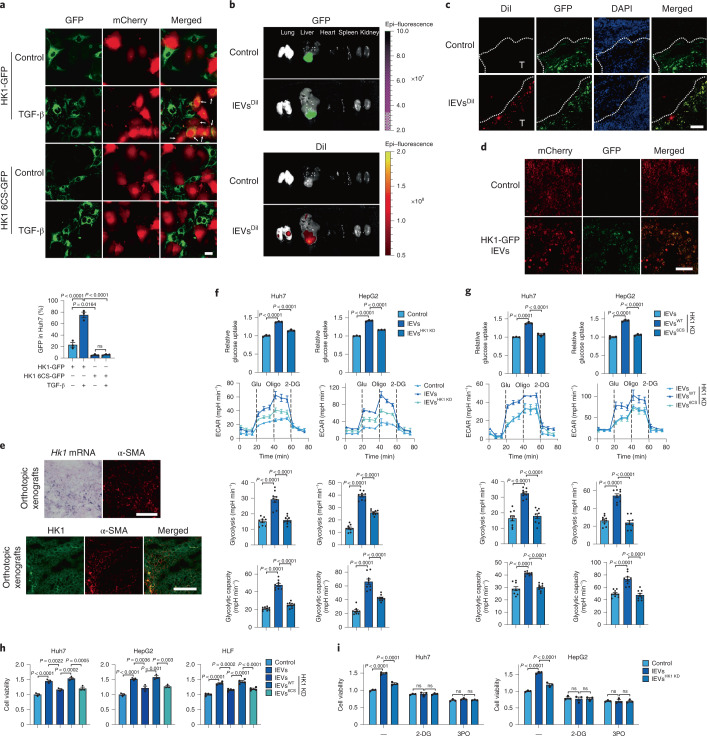
lEV HK1 is hijacked by HCC cells to promote glycolysis. **a**, HK1-GFP-expressing or HK1 6CS-GFP-expressing LX-2 cells and mCherry-expressing Huh7 cells were cocultured for 48 h, and the proportion of GFP taken up by Huh7 cells (%) is indicated. Scale bar, 10 μm. *n* = 3 independent experiments. **b**,**c**, GFP-expressing Hepa1-6 cells were orthotopically inoculated into mice liver. Two weeks later, DiI-labeled (0.5 μM) lEVs derived from TGF-β-treated moHSCs were intravenously injected into these mice for 12 h. GFP and DiI signals in different tissues are shown (**b**), and corresponding xenograft tissue sections were observed (**c**; scale bar, 100 μm). **d**, Uptake of lEV HK1 by hepatoma cells in vivo. mCherry-expressing Hepa1-6 cells were used to generate orthotopic xenografts in C57BL/6 mice. lEVs derived from activated HK1-GFP-expressing moHSCs were intravenously injected into the mice. Scale bar, 100 μm. **e**, The mRNA level of *Hk1* was detected by in situ hybridization experiment, and the protein levels of HK1 and α-SMA were detected by immunofluorescence assay in the sections from orthotopic xenografts (scale bar, 100 μm). **f**,**g**, HK1 was knocked down with or without re-expression of HK1 or HK1 6CS in LX-2 cells. Huh7 and HepG2 cells were incubated with different groups of lEVs derived from activated LX-2 cells as indicated for 12 h, and the ECAR (*n* = 9, 3 independent samples were detected and each sample was measured three times during 20–40 min of the timeline shown as ECAR measurement curves) and glucose uptake (*n* = 3 independent experiments) were determined. **h**,**i**, Effect of different groups of lEVs or inhibitors on Huh7, HepG2 and HLF cell viability. Cells were incubated with different groups of lEVs for 72 h (**h**; *n* = 3 independent experiments for Huh7 and HepG2 cells; *n* = 4 independent experiments for HLF cells) with or without 2-DG (10 mM) and 3-PO (20 μM) treatment (**i**; *n* = 3 independent experiments). WT, wild-type. Statistical data are presented as the mean ± s.e.m. Statistical analyses were determined by one-way ANOVA followed by Tukey’s post hoc test (**a**, **f**, **g**, **h**) and two-way ANOVA followed by Tukey’s multiple comparison test (**i**). Source data

**Extended Data Fig. 3:**
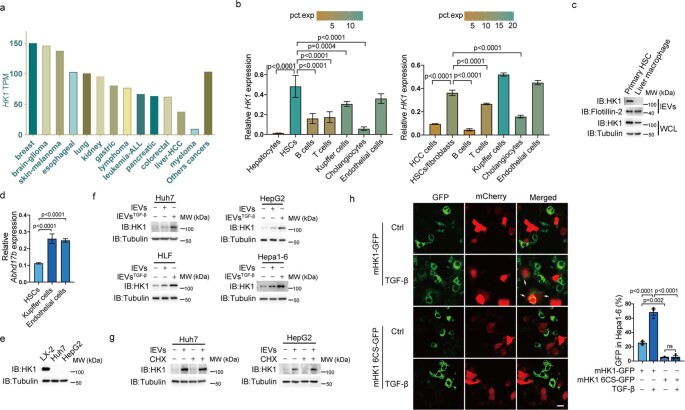
HCC cells hijack lEV HK1 derived from HSCs. **(a)** *HK1* expression levels in different types of cancer cell lines from the Cancer Cell Line Encyclopedia (CCLE) collection. **(b)** *HK1* expression levels were analyzed using scRNA-seq data from normal liver tissues (GSE158723, cell numbers: 9823 hepatocytes, 124 HSCs, 184 B cells, 186 T cells, 1405 Kupffer cells, 515 cholangiocytes and 472 endothelial cells) and HCC tissues (GSE151530, cell numbers: 20640 HCC cells, 1820 HSCs, 1167 B cells, 21131 T cells, 5983 Kupffer cells, 2659 cholangiocytes and 3321 endothelial cells). **(c)** lEV HK1 was detected in primary HSCs and primary macrophages from CCl_4_-induced liver fibrosis mice. **(d)** *Abhd17b* expression levels in HSCs, Kupffer cells and endothelial cells were analyzed using scRNA-seq data (GSE171904, cell numbers: 6472 HSCs, 465 Kupffer cells, 3439 endothelial cells). **(e)** The HK1 protein was undetectable in Huh7 or HepG2 cells. **(f)** Huh7, HepG2 and HLF cells were treated with lEVs derived from LX-2 cells. Hepa1-6 cells was treated with lEVs derived from mo HSCs. **(g)** Effect of CHX on HK1 transmission into Huh7 and HepG2 cells. Cells were incubated with CHX (100 µg/mL) and lEVs extracted from TGF-β-treated LX-2 cells for 12 hours. **(h)** Comparison of HK1-GFP and HK1 6CS-GFP uptake by Hepa1-6 cells (indicated by arrows). HK1-GFP- or HK1 6CS-GFP-expressing mo HSCs cultured together with mCherry-expressing Hepa1-6 cells for 48 hours with or without TGF-β treatment. Scale bar, 10 μm, n = 3 independent experiments. Tubulin was used as a protein loading control. Statistic data are presented as the mean ± SEM. Statistical analyses were determined by one-way ANOVA, followed by Tukey’s post hoc test (b, d, h). All western blots are repeated three times and one of them is shown. Source data

HK1 expression was found to be much lower in hepatoma cell lines than in nonhepatoma cell lines analyzed from the Cancer Cell Line Encyclopedia (CCLE) collection (Extended Data Fig. 3a). Analysis of publicly available single-cell RNA sequencing (scRNA-seq) data revealed that in healthy livers, hepatocytes express a relatively low level of *HK1*, whereas HSCs are the major cell type that expresses *HK1*. In HCC samples, the expression of *HK1* in HCC cells was much lower than that in HSCs or fibroblasts (Extended Data Fig. 3b). Although *HK1* is also highly expressed in Kupffer cells or macrophages, and endothelial cells (Extended Data Fig. 3b), we did not detect obvious secretion of HK1 from primary liver macrophages (Extended Data Fig. 3c), which might be because of the high *Abhd17b* expression in Kupffer cells and endothelial cells (Extended Data Fig. 3d). Therefore, we proposed that HSC-derived lEV HK1 might be taken up by HCC cells. In line with the above analysis, HK1 protein expression was undetectable in the human HCC cell lines Huh7 and HepG2 (Extended Data Fig. 3e). When Huh7, HepG2 and HLF cells (another HCC cell line with a higher degree of aggressiveness) were incubated with lEVs derived from TGF-β-stimulated LX-2 cells, or Hepa1-6 mouse HCC cells were treated with activated moHSC-derived lEVs, the protein level of HK1 was clearly elevated compared with the corresponding control cells (Extended Data Fig. 3f), and the protein level of HK1 was not reduced by cycloheximide, an inhibitor of de novo protein synthesis (Extended Data Fig. 3g), implying the direct transfer of the HK1 protein from LX-2-derived lEVs to HCC cells. To further verify this finding, we cocultured HSCs (LX-2 cells or moHSCs) that overexpressed HK1-green fluorescent protein (GFP) or HK1 6CS-GFP with mCherry-expressing HCC cells (Huh7 or Hepa1-6 cells). TGF-β elevated the HK1-GFP signal but not the HK1 6CS-GFP signal in HCC cells (Fig. 3a and Extended Data Fig. 3h). Therefore, HSC-derived lEV HK1 is taken up by HCC cells.

To further confirm this finding in vivo, we intravenously injected 1,1′-dioctadecyl-3,3,3′,3′-tetramethylindocarbocyanine perchlorate (DiI)-labeled moHSC-derived lEVs into C57BL/6 mice bearing orthotopic xenografts derived from GFP-expressing Hepa1-6 cells. The DiI signal was detected mainly in the liver, and approximately 58% of the total DiI signal was overlapped with the GFP signal. Less DiI signal was also taken up in the lung (Fig. 3b). In xenograft tissue sections, most of the DiI signal was colocalized with the GFP signal (Fig. 3c), suggesting that HCC cells may take up HSC-derived lEVs more efficiently than noncancerous cells. Similarly, when lEVs isolated from moHSCs expressing HK1-GFP were injected intravenously into mice bearing mCherry-expressing Hepa1-6 cell–derived orthotopic xenografts, the HK1-GFP signal was observed mainly in mCherry-positive cells (Fig. 3d). Moreover, the in situ mRNA detection showed that *Hk1* mRNA expression was mainly detected in the region with α-SMA-positive cells (Fig. 3e, top), indicating that HSCs or fibroblasts are the main source of HK1. However, HK1 protein expression was clearly detected in both α-SMA-positive and α-SMA-negative cells (Fig. 3e, bottom). Therefore, it is likely that the HK1 protein in HCC cells is derived from HSCs or fibroblasts but not expressed by themselves.

Given the important role of HK1 in glycolysis, we tested whether LX-2 cell–derived lEV HK1 can promote glycolysis in HCC cells. Glucose uptake and the extracellular acidification rate (ECAR) in Huh7 and HepG2 cells were clearly elevated upon incubation with lEVs from control cells but not with those from HK1 knockdown LX-2 cells (Fig. 3f). In HK1 knockdown LX-2 cells, re-expression of HK1 but not HK1 6CS rescued the ability of LX-2-derived lEVs to elevate glucose uptake and the ECAR in Huh7 and HepG2 cells (Fig. 3g). As a result, uptake of LX-2-derived lEVs led to enhanced proliferation of Huh7, HepG2 and HLF cells (Fig. 3h). However, knockdown of HK1 or abolishment of lEV HK1 secretion through HK1 6CS mutant expression in LX-2 cells markedly suppressed the proliferation-promoting effect of the lEVs. When Huh7 and HepG2 cells were treated with 2-deoxy-D-glucose (2-DG) or 3-(3-pyridinyl)-1-(4-pyridinyl)-2-propen-1-one (3-PO), two glycolysis inhibitors, Huh7 and HepG2 cell proliferation was no longer enhanced by lEVs from LX-2 cells (Fig. 3i). Thus, HK1 released from HSCs is hijacked by HCC cells, which then promotes HCC cell proliferation through glycolytic reprogramming.

### HSC-derived lEV HK1 promotes HCC progression in mouse models

**Fig. 4:**
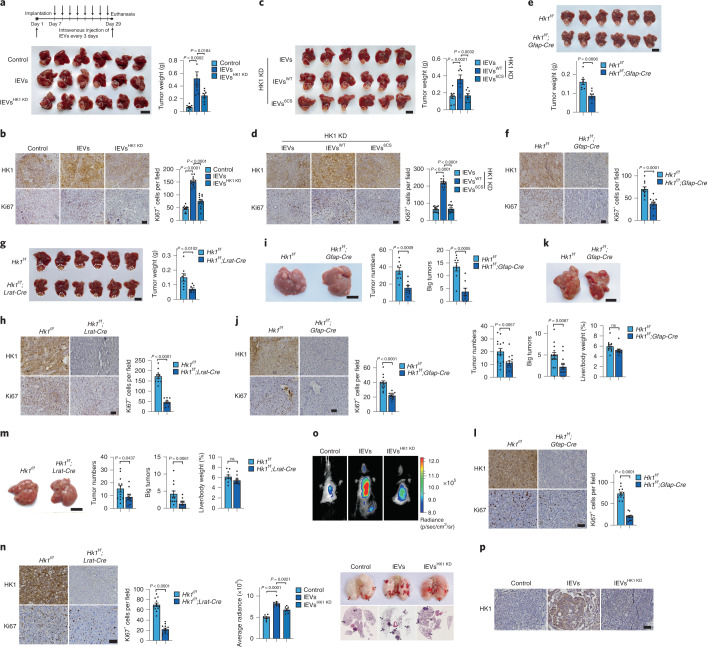
Mouse models demonstrate the role of lEV HK1 in HCC progression. **a**–**d**, Mice bearing orthotopic xenografts were intravenously injected with lEVs derived from different activated moHSCs indicated. Representative images (scale bar, 1 cm) and weights of orthotopic tumors are indicated (**c**; *n* = 8 independent mice). The expression of HK1 and Ki67 is shown (**b**, **d**; scale bar, 100 μm; *n* = 12 fields from three independent tumor tissues). **e**–**h**, Representative images (scale bar, 1 cm) and weights of Hepa1-6 orthotopic liver tumors in *Hk1*^*f/f*^ and *Hk1*^*f/f*^;*Gfap-Cre* (**e**) or *Hk1*^*f/f*^;*Lrat-Cre* (**g**) mice are indicated (**e**, **g**; *n* = 8 independent mice). The expression of HK1 and Ki67 in tumors is shown (**f**, **h**; scale bar, 100 μm; *n* = 10 fields from three independent tumor tissues). **i**,**j**, Representative images of the livers (scale bar, 1 cm) in *Hk1*^*f/f*^ and *Hk1*^*f/f*^;*Gfap-Cre* mice with DEN/CCl_4_-induced HCC (*n* = 8 independent mice) are indicated, and tumor number and large tumor number (Φ > 3 mm) per mouse were quantified (**i**). The expression of HK1 and Ki67 in tumors is shown (**j**; scale bar, 100 μm; *n* = 12 fields from three independent tumor tissues). **k**–**n**, Representative images of the liver (scale bar, 1 cm), total tumor number and large tumor number (Φ > 3 mm) per mouse and liver/body weight ratio in *Hk1*^*f/f*^ and *Hk1*^*f/f*^;*Gfap-Cre* mice (**k**; *n* = 14 independent mice), or *Hk1*^*f/f*^*;Lrat-Cre* mice (**m**; *n* = 12 independent mice). The expression of HK1 and Ki67 is shown (**l**, **n**; scale bar, 100 μm, *n* = 12 fields from three independent tumor tissues). **o**,**p**, lEVs derived from control or HK1 knockdown moHSCs were injected via tail vein into mice that were intravenously inoculated with luciferase-expressing Hepa1-6 cells. Tumor metastases were indicated by the luciferous signals and hematoxylin and eosin (H&E) staining (**o**; *n* = 8 independent mice). The HK1 expression in metastatic tumors are shown (**p**; scale bar, 100 μm. Three independent mice in each group were detected with similar results. Statistical data are presented as the mean ± s.e.m. Statistical analyses were determined by two-tailed Student’s *t*-test (**e**–**n**) and one-way ANOVA, followed by Tukey’s post hoc test (**a**–**d**, **o**). Source data

The role of LX-2 cell–derived lEV HK1 in promoting HCC progression was further verified in mouse models. Intravenous injection of lEVs derived from control moHSCs but not those derived from HK1 knockdown moHSCs notably promoted the growth of Hepa1-6 cell–derived orthotopic xenograft tumors (Fig. 4a), associated with elevated protein levels of HK1 and Ki67, a marker of cell proliferation, in tumor tissues (Fig. 4b). When lEV HK1 secretion in HSCs was abolished by mutation of HK1 6CS, HSC-derived lEVs lost their ability to promote xenograft tumor growth (Fig. 4c), and HK1 and Ki67 expression was reduced in tumor tissues (Fig. 4d). Hence, HSC-derived lEV HK1 accelerates xenograft tumor growth.

**Extended Data Fig. 4:**
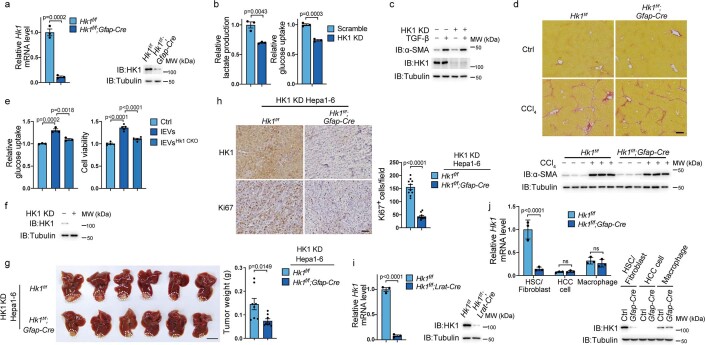
Large extracellular vesicle HK1 derived from HSCs promoted HCC proliferation. **(a)** Primary HSCs were isolated from *Hk1*^*f/f*^ or *Hk1*^*f/f*^*;Gfap-Cre* mice. The HK1 level was detected by qRT-PCR and western blotting, n = 3 independent experiments. **(b)** Detection of lactate production and glucose uptake with or without HK1 KD in LX-2 cells, n = 3 independent experiments. **(c)** α-SMA expression were detected in control or HK1 KD LX-2 cells with or without TGF-β (2 ng/mL) stimulation for 36 hours. **(d)** CCl_4_-induced liver fibrosis in *Hk1*^*f/f*^ or *Hk1*^*f/f*^*;Gfap-Cre* mice were determined by Sirius Red staining (top) and the detection of α-SMA (bottom, n = 3 independent mice) in liver tissue. Scale bar, 100 μm. **(e)** Glucose uptake (n = 3 independent experiments) and cell proliferation (n = 5 independent experiments) of Hepa1-6 cells were determined after incubation of activated primary HSC-derived lEVs. **(f)** HK1 knockdown efficiency in Hepa1-6 cells. **(g)** Representative images (scale bar, 1 cm) and weights of HK1 KD Hepa1-6 cell-derived orthotopic xenografts in *Hk1*^*f/f*^ and *Hk1*^*f/f*^*;Gfap-Cre* mice (n = 8 independent mice). **(h)** Immunohistochemical staining showed the expression of HK1 and Ki67 in corresponding tumor samples. Scale bar, 100 μm, n = 10 fields from 3 independent tumor tissues. **(i)** Primary HSCs were isolated from *Hk1*^*f/f*^ or *Hk1*^*f/f*^;*Lrat-Cre* mice. The mRNA and protein levels of HK1 were detected (n = 3 independent experiments). **(j)** Systematic analysis of HK1 expression in HFD/STZ-induced hepatocarcinoma between *Hk1*^*f/f*^ and *Hk1*^*f/f*^;*Gfap-Cre* mice. Primary cells as indicated were isolated from livers of HCC mice and the mRNA and protein levels of HK1 were detected (n = 3 independent experiments). Statistic data are presented as the mean ± SEM. Statistical analyses were determined by two-tailed Student’s t-test (a, b, g, h, i, j) and one-way ANOVA, followed by Tukey’s post hoc test (e). All western blots are repeated three times and one of them is shown. Source data

To further demonstrate the protumoral function of HSC-derived HK1, we tried to specifically knock out *Hk1* in the HSCs. To this end, *Hk1*^*f/f*^ mice were hybridized with mice expressing glial fibrillary acid protein promoter‐driven Cre recombinase (*Gfap-Cre*)^1^ to generate *Hk1*^*f/f*^;*Gfap-Cre* mice. We then isolated primary HSCs from these mice and found that HK1 was efficiently knocked out (Extended Data Fig. 4a). Although HK1 was important for glucose metabolism in LX-2 cells (Extended Data Fig. 4b), knocking down HK1 barely regulated the activation of LX-2 cells (Extended Data Fig. 4c). Similarly, knockout of HK1 in HSCs did not influence hepatic fibrosis in mice of the CCl_4_-induced liver fibrosis model (Extended Data Fig. 4d). When Hepa1-6 cells were incubated with lEVs released from primary HSCs of *Hk1*^*f/f*^ mice, glucose uptake and cell proliferation were obviously upregulated, but these effects were not seen after incubation with lEVs released from primary HSCs of *Hk1*^*f/f*^;*Gfap-Cre* mice (Extended Data Fig. 4e). Thus, in *Hk1*^*f/f*^;*Gfap-Cre* mice, the growth of Hepa1-6-derived orthotopic xenografts and the expression of HK1 and Ki67 in tumor tissues were substantially decreased compared with those in *Hk1*^*f/f*^ mice (Fig. 4e,f). To verify the transfer of HK1 from HSCs, as well as the absence of de novo expression of HK1 in HCC cells in vivo, we used HK1 knockdown Hepa1-6 cells (Extended Data Fig. 4f) to establish orthotopic xenografts in *Hk1*^*f/f*^ and *Hk1*^*f/f*^;*Gfap-Cre* mice. HK1 expression was clearly detected in orthotopic xenografts derived from HK1 knockdown Hepa1-6 cells in *Hk1*^*f/f*^ mice but not in *Hki1*^*f/f*^;*Gfap-Cre* mice (Extended Data Fig. 4h), suggesting that the HK1 protein in tumor cells is likely transferred from HSCs in mice. This transfer of HK1 was accompanied by the growth of xenografts (Extended Data Fig. 4g) and the proliferation of tumor cells (Ki67) (Extended Data Fig. 4h). Moreover, we established another mouse model using the promoter of lecithin retinyl acyltransferase (Lrat) to drive HSC-specific Cre expression in floxed HK1 mice^31^. HSC-specific knockout of HK1 (Extended Data Fig. 4i) was clearly observed in these mice (*Hk1*^*f/f*^;*Lrat-Cre* mice), which was accompanied with the retarded tumor growth and suppressed expressions of HK1 and Ki67 in tumor tissues (Fig. 4g,h). Together, it can be concluded that the HSC-derived HK1 promotes the development of HCC xenografts.

Furthermore, different primary HCC mouse models were used to verify the protumoral function of lEV HK1 secreted by HSCs. In a diethylnitrosamine (DEN) and CCl_4_-induced mouse HCC model, which mimics many features of the development of human HCC such as fibrogenesis^32^, conditional knockout of HK1 in HSCs of *Hk1*^*f/f*^;*Gfap-Cre* mice obviously retarded the progression of HCC (Fig. 4i) and decreased HK1 and Ki67 expression in primary tumor tissues (Fig. 4j). In a streptozotocin (STZ) and high-fat diet (HFD)-induced hepatocarcinogenic mouse model, which mimics the nonalcoholic steatohepatitis (NASH)–hepatocarcinogenic process and closely follows human HCC progression^32^, conditional knockout of HK1 in HSCs of either *Hk1*^*f/f*^;*Gfap-Cre* mice or *Hk1*^*f/f*^;*Lrat-Cre* mice consistently retarded the development of HCC, accompanied by downregulated HK1 and Ki67 expression in primary tumor tissues (Fig. 4k–n). To further verify the specific deletion of HK1 in HSCs of *Hk1*^*f/f*^;*Gfap-Cre* mice in the STZ/HFD model, we isolated primary HSCs, macrophages and HCC cells. It was demonstrated that the mRNA and protein levels of HK1 in HSCs were much higher than those in macrophages and HCC cells, and GFAP-Cre-mediated recombination clearly diminished HK1 expression in HSCs but not in macrophages or HCC cells (Extended Data Fig. 4j). Finally, we found that HSC-derived HK1 can also influence extrahepatic metastasis of HCC, because lEVs derived from control Hepa1-6 cells but not those derived from HK1 knockdown Hepa1-6 cells significantly promoted lung tumor metastasis (Fig. 4o), with elevated expression of HK1 in the metastatic tissues (Fig. 4p). Together, these in vivo results consistently confirm that lEV HK1 from the fibrotic microenvironment is hijacked by HCC cells to promote tumor progression.

### Nur77 inhibits the secretion of lEV HK1 from HSCs

**Fig. 5:**
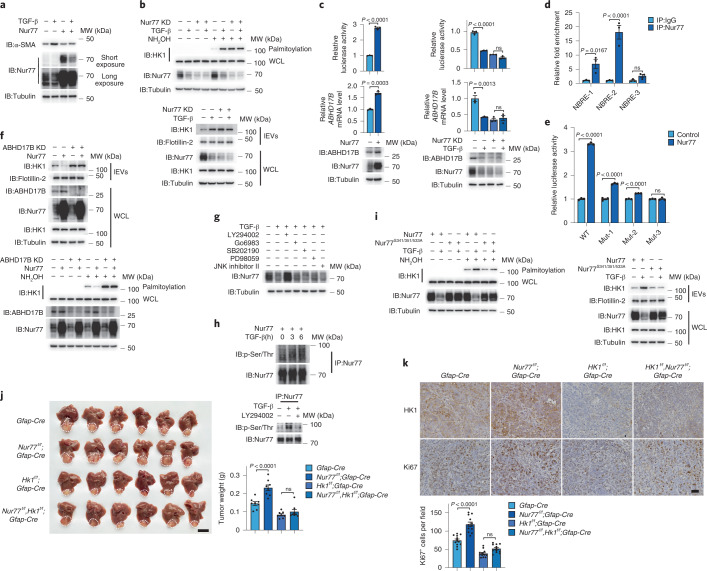
Nur77 positively represses lEV HK1 secretion from stromal HSCs. For the experiments described in this figure, LX-2 cells were treated with TGF-β (2 ng ml^−1^) for 36 h as indicated, and cell lysates and lEVs were prepared for western blotting unless specifically stated otherwise. **a**, Effect of Nur77 on TGF-β-induced α-SMA expression. **b**, Detection of TGF-β-induced HK1 palmitoylation (top) and secretion (bottom) with or without Nur77 knockdown. **c**, Analysis of *ABHD17B* promoter activity and *ABHD17B* mRNA and protein expression levels in cells in which Nur77 was overexpressed (left) or knocked down (right) (*n* = 3 independent experiments). **d**, Nur77 bound NBRE-like sequences at the *ABHD17B* promoter detected by ChIP assay (*n* = 3 independent experiments). **e**, The mutation of NBRE-like sequences impaired Nur77 activation on the *ABHD17B* promoter (*n* = 3 independent experiments). **f**, Nur77 inhibited HK1 palmitoylation and secretion through ABHD17B mediation. **g**, Effect of different inhibitors on TGF-β-induced Nur77 degradation. Cells were treated with TGF-β and different protein kinase inhibitors for 6 h (LY294002 (20 μM), Go6983 (100 nM), SB202190 (10 μM), PD98059 (20 μM) and JNK inhibitor II (20 μM)). **h**, TGF-β-induced Nur77 phosphorylation (top) was inhibited by LY294002 (bottom). **i**, Effect of Nur77 or its phosphorylation mutant on HK1 palmitoylation and secretion. **j**,**k**, Effect of Nur77 and HK1 knockout in HSCs on the growth of orthotopic xenograft tumors (**j**; scale bar, 1 cm; *n* = 8 independent mice) and the expressions of HK1 and Ki67 in tumor tissues (**k**; scale bar, 100 μm; *n* = 12 fields from three independent tumor tissues). Flotillin-2 was used as a loading control for EVs. Tubulin was used as a protein loading control. Statistical data are presented as the mean ± s.e.m. Statistical analyses were determined by two-tailed Student’s *t*-test (**c**, left), one-way ANOVA followed by Tukey’s post hoc test (**c**, right; **j**, **k**), and two-way ANOVA followed by Sidak’s multiple comparison test (**d**, **e**). All western blots were repeated three times and one of them is shown. Source data

**Extended Data Fig. 5:**
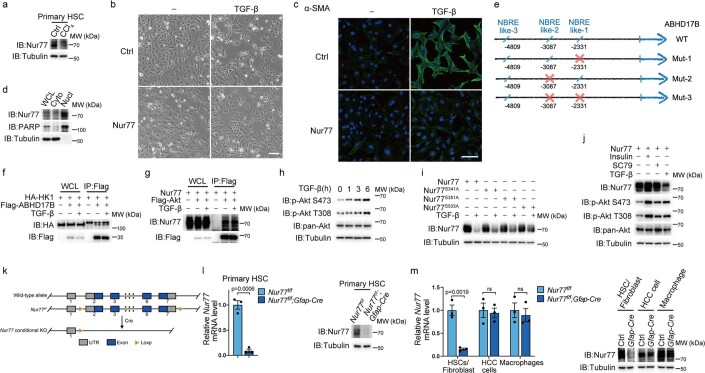
Nur77 degradation was induced by TGF-β in Akt-dependent manner. **(a)** Nur77 expression was detected in primary HSCs from control and CCl_4_-induced liver fibrosis mice. **(b-c)** Effect of Nur77 on the myofibroblast-like morphology (b) and α-SMA expression (c) in LX-2 cells. Scale bar, 100 μm. **(d)** Localization of endogenous Nur77 in LX-2 was detected by fractionation assay. **(e)** Three Nur77-binding response element (NBRE)-like sequences in the *ABHD17B* promoter are indicated. **(f)** Effect of TGF-β on the interaction between HK1 and ABHD17B. Cells were treated with TGF-β (2 ng/mL) for 3 hours. **(g)** Effect of TGF-β on the interaction between Nur77 and Akt. **(h)** Effect of TGF-β on Akt activity in LX-2 cells. **(i)** Effects of Nur77 mutation at the three indicated phosphorylation sites. LX-2 cells were treated for 6 hours with or without TGF-β (2 ng/mL). **(j)** The levels of Nur77, Akt and Akt phosphorylation were detected in LX-2 cells that were treated with insulin (5 μg/mL), SC79 (10 μM) or TGF-β 2 ng/mL) for 6 hours. **(k)** Scheme showing construction of the *Nur77*^*f/f*^ mouse strain. **(l)** The mRNA and protein expression of Nur77 were detected in primary HSCs from *Nur77*^*f/f*^ or *Nur77*^*f/f*^;*Gfap-Cre* mice. **(m)** Systematic analysis of Nur77 expression in Hepa1-6-induced orthotopic HCC between *Nur77*^*f/f*^ and *Nur77*^*f/f*^;*Gfap-Cre* mice. Primary cells as indicated were isolated from fresh livers in HCC mice and the mRNA and protein levels of Nur77 were detected by qRT-PCR and western blotting. Tubulin was used as a protein loading control. Statistic data are presented as the mean ±·SEM. Statistical analyses were determined by two-tailed Student’s t-test (l, m). All western blots are repeated three times and one of them is shown. Source data

The results described above suggest that blocking TGF-β-induced HK1 secretion may represent a strategy to inhibit the development of HCC. Nur77, an orphan nuclear receptor, has been reported to suppress TGF-β-induced fibrosis^33^. Given that TGF-β was found to trigger lEV HK1 secretion in HSCs and that Nur77 expression was suppressed during liver fibrosis (Extended Data Fig. 5a), we hypothesized that Nur77 is involved in the regulation of HK1 secretion in HSCs. Transfection of Nur77 into LX-2 cells inhibited TGF-β-induced fibrosis (Extended Data Fig. 5b,c), accompanied by the inhibition of α-SMA (Fig. 5a). Knockdown of Nur77 enhanced secretion of HK1 in lEVs, associated with enhanced HK1 palmitoylation (Fig. 5b), implying that Nur77 inhibits HK1 palmitoylation to impede the secretion of lEV HK1. In LX-2 cells, Nur77 was mainly localized in the nucleus (Extended Data Fig. 5d), which suggests that Nur77 may perform its function as a transcription factor to indirectly regulate HK1.

The promoter sequence of *ABHD17B* contains three Nur77-binding response element (NBRE)-like motifs (Extended Data Fig. 5e), suggesting that the transcriptional level of *ABHD17B* might be regulated by Nur77. Indeed, Nur77 markedly enhanced *ABHD17B* promoter activity, leading to elevated *ABHD17B* mRNA and protein levels in LX-2 cells (Fig. 5c, left). In contrast, knockdown of Nur77 inhibited the promoter activity, mRNA and protein expression of *ABHD17B* (Fig. 5c, right). A chromatin immunoprecipitation (ChIP) assay revealed that Nur77 mainly bound NBRE-1 and NBRE-2 but not NBRE-3 in the *ABHD17B* promoter (Fig. 5d). When both NBRE-1 and NBRE-2 were mutated, the ability of Nur77 to regulate the *ABHD17B* promoter activity was abolished (Fig. 5e). Hence, Nur77 transcriptionally upregulates ABHD17B expression. This upregulation of ABHD17B is required for Nur77 to regulate HK1 release, as knockdown of ABHD17B abolished the inhibitory effect of Nur77 on the palmitoylation and secretion of lEV HK1 in LX-2 cells (Fig. 5f). Collectively, Nur77 suppresses the secretion of lEV HK1 by stimulating the expression of the depalmitoylase ABHD17B, which inhibits HK1 palmitoylation.

Although TGF-β did not interfere with the interaction of ABHD17B and HK1 (Extended Data Fig. 5f), it clearly inhibited the expression of Nur77 (Fig. 5a–c). When Nur77 was knocked down in LX-2 cells, TGF-β not only lost its ability to suppress ABHD17B expression (Fig. 5c, right), but also failed to promote HK1 palmitoylation and lEV HK1 secretion (Fig. 5b). It is likely that TGF-β-induced degradation of Nur77 is required to induce the palmitoylation and secretion of lEV HK1. Since phosphorylation has been reported to regulate the protein stability of Nur77^34,35^, inhibitors of different protein kinases were used to treat LX-2 cells. Only LY294002, an inhibitor of the PI3K-Akt pathway, abolished TGF-β-induced degradation of Nur77 (Fig. 5g). Although TGF-β did not affect the Nur77–Akt interaction, it clearly activated Akt phosphorylation (Extended Data Fig. 5g,h), leading to phosphorylation of Nur77 in a PI3K-Akt-dependent manner (Fig. 5h). We further generated three Nur77 point mutants at sites potentially phosphorylated by Akt^36^. The single point mutations in Nur77 barely impaired TGF-β-induced Nur77 degradation (Extended Data Fig. 5i); only when all three phosphorylation sites were simultaneously mutated (Nur77^S341/351/533A^) was Nur77 resistant to TGF-β-induced degradation, thereby diminishing TGF-β-induced HK1 palmitoylation and lEV HK1 release (Fig. 5i). Other Akt activators, such as insulin and SC79^37^, could not induce Nur77 degradation (Extended Data Fig. 5j). Hence, phosphorylation of Nur77 by Akt is required, but inadequate, for TGF-β-induced Nur77 degradation, and preventing Nur77 phosphorylation by Akt is sufficient to block TGF-β-induced Nur77 degradation.

To further verify the effect of Nur77 in a mouse model, we generated the *Nur77*^*f/f*^ mouse strain (Extended Data Fig. 5k). When these mice were crossed with *Gfap-Cre* mice, Nur77 was specifically deleted in the HSCs (Extended Data Fig. 5l,m), which clearly promoted the growth of orthotopic xenograft tumors (Fig. 5j), leading to enhanced expression of HK1 and Ki67 in tumor tissues (Fig. 5k). When HK1 was also knocked out specifically in HSCs, deletion of Nur77 no longer influenced tumor growth (Fig. 5j,k). Thus, the expression of Nur77 in HSCs suppresses HCC progression by regulating the secretion of lEV HK1 from HSCs.

### The small molecule PDNPA targets Nur77 to inhibit lEV HK1 secretion

**Fig. 6:**
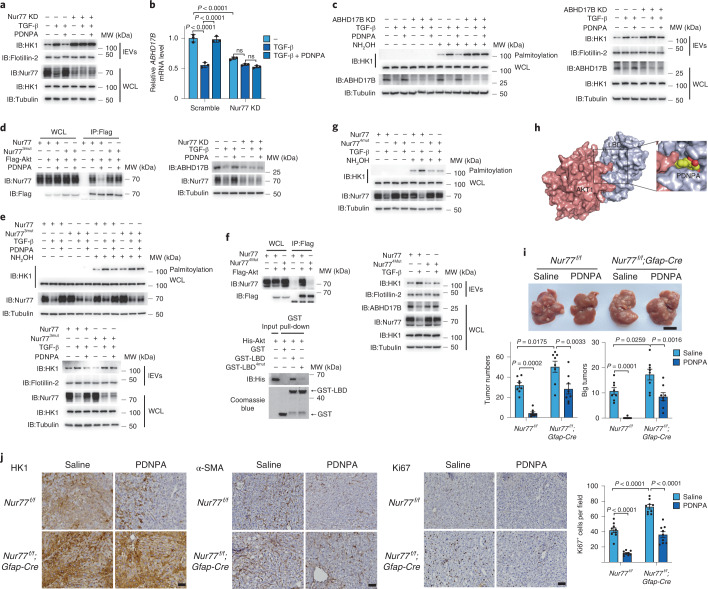
PDNPA targets Nur77 to inhibit lEV HK1 release. For the experiments described in this figure, LX-2 cells were treated with TGF-β (2 ng ml^−1^) or PDNPA (10 μM) for 36 h as required unless specifically defined otherwise. **a**, Effect of PDNPA on TGF-β-induced HK1 secretion in the presence or absence of Nur77. **b**, Effect of PDNPA on *ABHD17B* mRNA and protein expression levels mediated by Nur77. **c**, Roles of PDNPA in HK1 palmitoylation and secretion upon ABHD17B knockdown. **d**, Effect of PDNPA on the interaction between Nur77 or a Nur77 mutant (Nur77^3*mut*^) and Akt. **e**, Comparison of the effect of PDNPA on Nur77-mediated and Nur77^3*mut*^-mediated HK1 palmitoylation and secretion. **f**, Assessment of the interaction between Nur77 or a Nur77 mutant (Nur77^4*mut*^) and Akt upon transfection (top) and GST pull-down assay (bottom). **g**, Effect of Nur77 or Nur77^4*mut*^ on HK1 palmitoylation and secretion. **h**, A docking model indicates that the binding of PDNPA to the Nur77 LBD produces steric hindrance that blocks AKT targeting. **i**,**j**, Effect of PDNPA on DEN/CCl_4_-induced HCC development in *Nur77*^*f/f*^ and *Nur77*^*f/f*^;*Gfap-Cre* mice (**I**; *n* = 8 independent mice; scale bar, 1 cm). Total tumor number and large tumor number (Φ > 3 mm) per mouse were quantified. The expressions of HK1, α-SMA and Ki67 in corresponding tumor samples are shown (**j**; scale bar, 100 μm; *n* = 9 fields from three independent tumor tissues). Flotillin-2 was used as a loading control for EVs. Tubulin was used as a protein loading control. Statistical data are presented as the mean ± s.e.m. Statistical analyses were determined by two-way ANOVA followed by Tukey’s (**b**) and Sidak’s (**i**, **j**) multiple comparison tests. All western blots were repeated three times and one of them is shown. Source data

**Extended Data Fig. 6:**
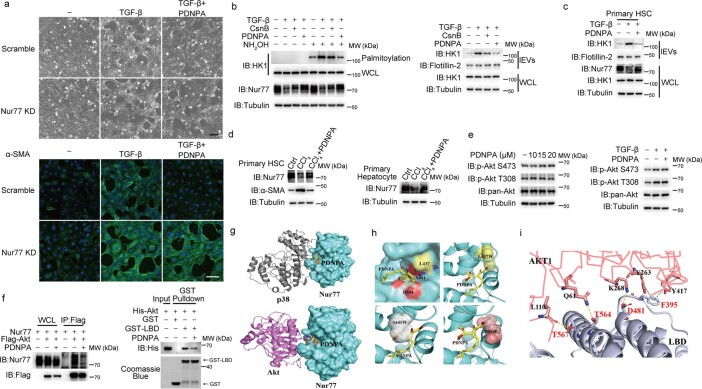
The binding of PDNPA to Nur77 generated hindrance to impede the interaction between Nur77 and Akt. **(a)** PDNPA inhibited TGF-β-mediated induction of a myofibroblast-like morphology (top) and α-SMA expression (bottom) in LX-2 cells in a Nur77-dependent manner. Scale bar, 100 μm, 3 times experiments were repeated independently with similar results. **(b)** Comparison of the effect of PDNPA and Csn-B on TGF-β-induced HK1 palmitoylation and secretion. TGF-β (2 ng/mL), PDNPA (10 μM) or Csn-B (10 μM) were used to treat LX-2 cells for 36 hours. **(c)** Effect of PDNPA on Nur77 expression and HK1 secretion detected in primary HSCs. **(d)** Nur77 protein levels were detected in primary HSCs and hepatocytes isolated from control mice and CCl_4_-induced liver fibrosis mice treated with or without PDNPA. **(e)** PDNPA had no effect on TGF-β-induced Akt activity. **(f)** Detection of the interaction between the Nur77 LBD and Akt in the presence of PDNPA upon transfection in LX-2 cells (left) and in an *in vitro* GST pulldown assay (right). **(g)** A docking model showing that Akt and p38 bind the same pocket of the Nur77 LBD. **(h)** Crystal structures of the S441W and L437W D594E mutants shows that the introduction of bulkier residues prevents PDNPA binding. **(i)** A docking model indicates that F395A, D481A, T564A and T567A impair the interaction between the Nur77 LBD and Akt. Tubulin was used as a protein loading control. All western blots are repeated three times and one of them is shown. Source data

The finding above supports that blockade of the interaction between Akt and Nur77 attenuates the effect of TGF-β on Nur77, thereby preventing lEV HK1 release. Although cytosporone B (Csn-B), originally identified by our team as an agonist of Nur77^38^, has been reported to inhibit fibrosis of the skin, lung, liver and kidney in mice^33^, it barely inhibited HK1 palmitoylation or lEV HK1 release in LX-2 cells. In contrast, PDNPA, a derivative of Csn-B that also binds to Nur77 to regulate its function^39^, was identified from our in-house library. PDNPA obviously restored the TGF-β-reduced Nur77 protein level (Fig. 6a), thereby not only inhibiting the TGF-β-induced fibrotic morphology and α-SMA expression in LX-2 cells (Extended Data Fig. 6a), but also suppressing TGF-β-induced palmitoylation and secretion of HK1 in a Nur77-dependent manner (Fig. 6a and Extended Data Fig. 6b). The effects of PDNPA on elevating Nur77 expression and suppressing HK1 secretion were also demonstrated in TGF-β-treated primary HSCs (Extended Data Fig. 6c) or primary HSCs derived from the CCl_4_-induced liver fibrosis model (Extended Data Fig. 6d, left). We also observed PDNPA-elevated Nur77 expression in primary hepatocytes derived from the CCl_4_-induced liver fibrosis model (Extended Data Fig. 6d, right), suggesting that the effect of PDNPA on Nur77 expression is not restricted to HSCs. Moreover, PDNPA treatment rescued ABHD17B expression at both the gene and protein levels in a Nur77-dependent manner (Fig. 6b, top and bottom), thereby abolishing TGF-β-induced HK1 palmitoylation and secretion mediated by ABHD17B (Fig. 6c). Hence, PDNPA is a potential candidate compound to block HK1 release from HSCs via activating the Nur77–ABHD17B axis.

Although PDNPA did not impair Akt phosphorylation even in the presence of TGF-β (Extended Data Fig. 6e), it attenuated the Nur77–Akt interaction detected in vitro and upon transfection (Extended Data Fig. 6f). We previously demonstrated that PDNPA binds to the ligand-binding domain (LBD) of Nur77 to prohibit the interaction of Nur77 and p38^39^. Interestingly, structural docking analysis indicates that Akt and p38 bind to the same pocket in the Nur77 LBD (Extended Data Fig. 6g), echoing the finding that PDNPA also impedes the Nur77–Akt interaction. When three sites (L437, S441 and D594) in Nur77 critical for PDNPA binding were mutated (Extended Data Fig. 6h; Nur77^3*mut*^)^39^, PDNPA no longer inhibited the Nur77–Akt interaction, TGF-β-induced Nur77 degradation, HK1 palmitoylation or lEV HK1 secretion (Fig. 6d,e). According to the docking model, F395, D481, T564 and T567 in Nur77 are important for the binding of Akt (Extended Data Fig. 6i). Mutation of these four amino acid residues (Nur77^4*mut*^) impaired the Nur77–Akt interaction and diminished TGF-β-induced HK1 palmitoylation and lEV HK1 secretion (Fig. 6f,g). Together, these findings demonstrate that the binding of PDNPA to the Nur77 LBD generates steric hindrance that blocks Akt targeting (Fig. 6h).

The role of PDNPA in repressing fibrosis and HCC progression was further determined in a DEN/CCl_4_-induced mouse model of HCC. PDNPA administration significantly reduced the tumor number (Fig. 6i), accompanied by decreased HK1 and Ki67 expression in tumor tissues (Fig. 6j). However, after specific deletion of Nur77 in HSCs (*Nur77*^*f/f*^;*Gfap-Cre*), the inhibitory effects of PDNPA on HCC development and the expression of HK1 and Ki67 were diminished. These results suggest a causal relationship in vivo, in which the elevation of Nur77 expression induced by PDNPA inhibits HCC development by suppressing the secretion of lEV HK1 from HSCs.

## Discussion

**Fig. 7:**
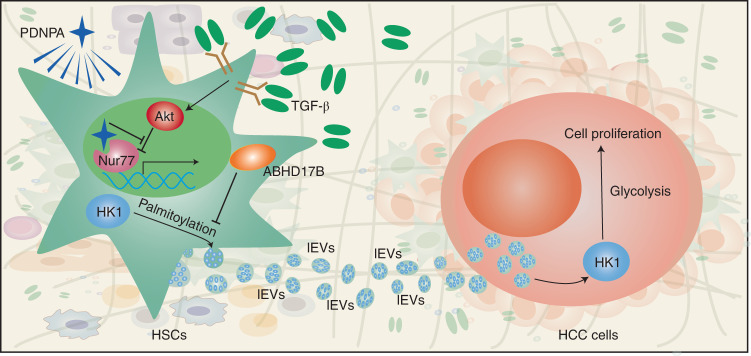
During hepatic fibrosis, TGF-β-activated Akt phosphorylates Nur77 to induce its degradation in HSCs, leading to the suppressed expression of depalmitoylase ABHD17B, a downstream target gene of Nur77. The palmitoylation of HK1 is thus elevated, which promotes HK1 translocation to the plasma membrane for subsequent secretion via lEVs. The HSC-derived lEV HK1 is hijacked by HCC cells, which facilitates the reprogramming of glycolysis to promote HCC progression. Compound PDNPA antagonizes TGF-β-induced Nur77 degradation and inhibits HK1 secretion, thereby effectively repressing HCC progression.

EVs derived from cancer cells and stromal cells are considered some of the most important mediators of cellular crosstalk between tumors and the tumor microenvironment. On the one hand, EVs derived from cancer cells promote angiogenesis and coagulation, modulate the immune system and remodel the surrounding parenchymal tissue. On the other hand, EVs derived from stromal cells are involved in tumor growth, invasion, metastasis and even drug resistance^40,41^. In this study, we demonstrated that lEV HK1 secretion from the stroma supported HCC progression. During TGF-β-induced liver fibrosis, the palmitoylation of HK1 was enhanced in activated HSCs, leading to lEV HK1 secretion. Importantly, HCC cells that expressed relatively low levels of HK1 hijacked lEV HK1 to enhance their proliferation through glucose metabolic reprogramming. The nuclear receptor Nur77 was shown to attenuate HK1 release from HSCs, and the Nur77-targeting compound PDNPA strengthened the inhibitory effect of Nur77 on HK1 release by disrupting Akt-mediated Nur77 degradation (Fig. 7).

HKs are important for tumor growth because of the addiction of tumor cells to glycolysis. In normal hepatocytes, HK4 is the major HK isoform expressed. However, with the development of HCC, HK4 expression is suppressed, whereas HK2 expression is induced^13,42^. Genetic ablation of *Hk2* in the liver decreases the incidence and growth of tumors in a DEN-induced mouse model of HCC^13^. Therefore, HK2 is believed to be an ideal cancer-specific target for HCC therapy. However, the role of HK1 in HCC development has long been ignored. Although analysis of cell lines from the CCLE collection revealed that most HCC cell lines express only HK2 and not HK1^43,44^, we found that HCC cells hijacked lEV HK1 secreted from activated HSCs to enhance their proliferation, reflecting the resourcefulness of tumor cells. Since more than 80% of HCC cases develop in the context of fibrosis or cirrhosis^45,46^, the functions of HSC-derived HK1 in HCC development cannot be neglected. Ablation of HK1 in HSCs or blockade of secretion of IEV HK1 from HSCs substantially impaired the development of HCC, emphasizing the promotive action of HSC-derived lEV HK1 in HCC development. Given that the Michaelis–Menten constant (*K*_m_) of HK1 for glucose is much lower than that of HK2^47^, supplementary lEV HK1 from the stromal microenvironment may not only strengthen glycolysis, but also expand the scope of glucose utilization to adapt the glucose limitation situation in tumors. Since rapidly growing tumors are usually confronted with glucose starvation because of the poor vascular supply^48^, and HCC is characterized by rapid tumor growth but seldom hypervascularity^49^, it is reasonable to speculate that HSC-derived lEV HK1 may play an important role for the metabolic plasticity of HCC cells. Although it is well accepted that HK2 is an effective target for the suppression of HK1-negative and HK2-positive (*HK1*^−^*HK2*^+^) tumors from a wide variety of tissue origins^44,50^, our study suggests that combined inhibition of HK2 and secretion of lEV HK1 from HSCs may be a therapeutic strategy for HCC that develops from hepatic fibrosis.

Plasma membrane localization is a prerequisite for the sorting of cargo into lEVs^6^. HK1 mainly localizes to the mitochondrial outer membrane^44^. Once palmitoylated, HK1 is directed to the plasma membrane for subsequent secretion. The trafficking of HK1 between the plasma membrane and mitochondria may be dynamically regulated by palmitoylation and depalmitoylation^51^. Specific inhibition of a single ZDHHC enzyme may not suppress HK1 palmitoylation because of functional redundancy. In contrast, the regulation of HK1 depalmitoylation is more straightforward. ABHD17B is the only enzyme found to catalyze the depalmitoylation of HK1 in our study. In this regard, ABHD17B is fundamental in orchestrating the precise intercellular distribution of HK1 and is thus a functional hub for the regulation of lEV HK1 secretion. Therefore, the expression of ABHD17B may determine lEV HK1 secretion. Analysis of the scRNA-seq data indicates that HSCs have much lower *Abhd17b* mRNA levels than macrophages and endothelial cells, which may at least partially explain why HK1 is inclined to be secreted via lEV in HSCs but not in macrophages or endothelial cells.

Nur77 has been reported to be an endogenous inhibitor of fibrosis through the recruitment of the SP1–SIN3A–CoREST–LSD1–HDAC1 repressor complex, inhibiting the expression of TGF-β target genes in fibroblasts;^33^ however, our study suggests that Nur77 might suppress HK1 secretion by promoting ABHD17B-mediated HK1 depalmitoylation and inhibiting the activation of HSCs. In both of these instances, the inhibitory effect of Nur77 was abrogated by TGF-β through Akt phosphorylation. On the one hand, Akt-dependent phosphorylation of Nur77 at S351 induced by TGF-β leads to inactivation of Nur77 at the transcriptional level in fibroblasts, thus facilitating fibrosis^52^. On the other hand, we found that TGF-β-activated Akt phosphorylated Nur77 to promote Nur77 degradation at the protein level in HSCs, resulting in enhanced HK1 secretion. Therefore, blocking Akt-mediated Nur77 phosphorylation may release Nur77 from TGF-β-induced inhibition at either the transcriptional or post-transcriptional level. Csn-B, an agonist of Nur77 identified by our group^38^, inhibits Akt-mediated Nur77 phosphorylation, thereby strengthening the inhibitory effect of Nur77 against fibrotic responses in fibroblasts^33^. This effect of Csn-B is consistent with our previous conclusion that Csn-B specifically binds to the Nur77 LBD and stimulates Nur77-dependent transactivation activity^38^. However, Csn-B barely inhibited lEV HK1 release from active HSCs, implying that it acts through a different mechanism dependent on cell type and tissue specificity in fibrosis. Interestingly, PDNPA, a Csn-B derivative, effectively repressed HK1 secretion by enhancing Nur77 protein stability, thus suppressing the progression of HCC. From a structural perspective, the binding of PDNPA to the Nur77 LBD generated steric hindrance to impede the interaction of Akt and Nur77, abolishing Akt-mediated Nur77 phosphorylation and degradation. We previously reported that PDNPA, as a competitive inhibitor, binds to the Nur77 LBD to inhibit the Nur77–p38 interaction, thereby relieving the suppression of Nur77 by p38-mediated phosphorylation and enhancing the anti-inflammatory effect of Nur77^39^. Given the anti-inflammatory activity of PDNPA^39^ and that HCC and hepatic fibrosis are usually accompanied by inflammation^53^, we speculate that PDNPA could be a promising multifunctional lead compound that ameliorates hepatic fibrosis and suppresses HCC development.

## Methods

### Cell culture and transfection

The human HSC line LX-2 (SCC064) was purchased from Millipore. The human embryonic kidney cell line 293T (CRL-11268), the human hepatoma cell line HepG2 (HB-8065) and the mouse hepatoma cell line Hepa1-6 (CRL-1830) were obtained from American Type Culture Collection (ATCC). The human hepatoma cell line Huh7 (TCHu182) was purchased from Cell Bank in the Chinese Academy of Sciences. moHSCs were isolated from livers of BALB/c mice and immortalized spontaneously. Primary mouse HSCs and primary mouse hepatocytes were isolated from livers of C57BL/6 mice. Cells were cultured in Dulbecco’s Modified Eagle’s Medium (DMEM) supplemented with 10% fetal bovine serum (FBS; Gemini), penicillin (100 IU) and streptomycin (100 mg ml^−1^) (Bio Basic Inc.), maintained at 37 °C in an atmosphere containing 5% CO_2_. All of these cell lines were routinely tested and found to be negative for mycoplasma. Transfections were performed using ViaFect Transfection Reagent (Promega) for LX-2 according to the manufacturer’s instructions, and the calcium–phosphate precipitation method for 293T cells.

### Antibodies and reagents

The goat anti-rabbit Alexa Fluor 594 (A-11037, 1:200 for immunofluorescence (IF)), goat anti-rabbit (31210, 1:5,000 for immunoblotting (IB)) and anti-mouse (31160, 1:5,000 for IB) antibodies were purchased from Thermo Fisher Scientific. Anti-Flag (F3165, 1:5,000 for IB), anti-HA (H9658, 1:5,000 for IB) and anti-tubulin (T4026, 1:5,000 for IB) antibodies were purchased from Sigma. Anti-HK1 (2024, 1:5,000 for IB), anti-HK2 (2867, 1:5,000 for IB), anti-flotillin-2 (3436, 1:5,000 for IB), anti-Nur77 (3960, 1:1,000 for IB), anti-PARP (9532, 1:2,000 for IB), anti-Akt (pan) (2920, 1:2,500 for IB), anti-phospho-Akt (Ser473) (3787, 1:2,500 for IB), anti-phospho-Akt (Thr308) (2965, 1:2,500 for IB) and anti-mouse IgG Alexa Fluor 488 (4408, 1:200 for IF) antibodies were purchased from Cell Signaling Technology. Anti-α-SMA (ab124964, 1:10,000 for IB), anti-Ki67 (ab16667, 1:200 for immunohistochemistry), anti-LAMP1 (ab25630, 1:2,500 for IB), anti-annexin A1 (ab214486, 1:5,000 for IB), anti-CD63 (ab134045, 1:5,000 for IB) and anti-alpha 1 sodium potassium ATPase (ab7671, 1:5,000 for IB) antibodies were purchased from Abcam. Anti-Hsp60 antibody (SC-376240, 1:5,000 for IB and 1:200 for IF) was purchased from Santa Cruz Biotechnology. Anti-Nur77 (12235-1-AP, 1:200 for ChIP), anti-annexin A2 (11256-1-AP, 1:5,000 for IB) and anti-His (HRP-66005, 1:5,000 for IB) antibodies were purchased from Proteintech. Anti-phospho-serine/threonine (612549, 1:1,000 for IB) and anti-CD11b-PE (561098, 1:100 for fluorescence-activated cell sorting (FACS)) antibodies were purchased from BD Biosciences. Anti-BSG antibody (A16662, 1:5,000 for IB) was purchased from ABclonal Technology. Anti-mouse Nur77 (14-5965-82, 1:1,000 for IB), anti-CD45-eFluor 450 (48-0451-82, 1:100 for FACS) and anti-F4/80-FITC (11-4801-82, 1:100 for FACS) antibodies were purchased from eBioscience. Anti-DIG-AP antibody (11093274910, 1:3,000 for in situ hybridization) was purchased from Roche. The rabbit polyclonal antibody against ABHD17B (1:1,000 for IB) was generated by immunizing rabbits with synthetic peptide corresponding to human ABHD17B (aa 50-64).

TGF-β (8915LC) and cycloheximide (2112S) were purchased from Cell Signaling Technology. SB505124 (HY-13521), LY294002 (HY-10108), PD98059 (HY-12028), 3PO (HY-19824) and pronase E (HY-114158) were purchased from MedChemExpress. DAPI (62247) and 2-NBDG (N13195) were purchased from Thermo Fisher Scientific. DiI (C1036) was purchased from Beyotime. DNase I (A610099) was purchased from Bio Basic Inc. 2-Bromohexadecanoic acid (2-BP, 21604), hydroxylamine hydrochloride ReagentPlus (NH_2_OH, 159417), *S*-methyl methanethiosulfonate purum (MMTS, 64306), SB202190 (S7067), Go6983 (G1918) and collagenase type IV (V900893) were purchased from Sigma. PalmB (178581) was purchased from Millipore. The Plasma Membrane Protein Extraction Kit (ab65400) was purchased from Abcam. GoTaq qPCR Master Mix (A6001) was purchased from Promega. Sirius red (G1018) was purchased from Servicebio.

### Generation of the lentiviral system

The lentiviral-based vector pLL3.7 or pLKO.1 was used to express short hairpin RNA (shRNA) in cells. The lentiviruses were generated by transfecting 293T cells with the lentiviral vector and packaged plasmids using calcium phosphate transfection. The viral supernatants were collected 48 h after the transfection, filtered through a 0.45 μm filter (Millipore), centrifuged at 75,000*g* for 90 min and resuspended. The cells were infected with lentiviruses plus polybrene for 24 h, then selected by puromycin. The oligonucleotide sequences for shRNA are provided in Supplementary information.

### Quantitative real-time PCR

Total RNA was extracted using TRI Reagent (Sigma), then reverse transcription was performed using a reverse transcriptase kit (Tiangen). Complementary DNA was used as a template, and quantitative real-time PCR experiments were performed using the GoTaq qPCR Master Mix. *β-actin* was used as a normalization control. The primer sequences are provided in Supplementary information.

### Immunoprecipitation

Immunoprecipitation was performed as previously described^54^. In brief, cells were lysed with lysis buffer (50 mM Tris-HCl (pH 7.6), 150 mM NaCl, 100 mM NaF, 0.5% Nonidet P-40) containing protease inhibitor cocktails (MedChemExpress) on ice. Cell lysates were incubated with corresponding antibodies and protein G-Sepharose beads (Millipore) at 4 °C for 3 h. The immunoprecipitants were collected and washed three times with lysis buffer, and subjected to western blotting analysis.

### ChIP assay

Cells were crosslinked by adding formaldehyde directly to culture medium to a final concentration of 1%, then incubated for 10 min at 37 °C. After washing with ice-cold PBS three times, pellets were suspended in SDS lysis buffer (50 mM Tris (pH 8.1), 10 mM EDTA and 1% SDS) containing protease inhibitors, and then sonicated chromatin to 500–1,000 base pairs. After centrifugation, soluble chromatin was incubated with rabbit IgG (negative control) or appropriate antibodies overnight at 4 °C. The antibody–chromatin complexes were pulled down using protein G-Sepharose beads, and the immunoprecipitants were then washed and eluted. The immunoprecipitants were subjected to crosslink reversal and proteinase K treatment. Eventually, the immunoprecipitated DNA was purified using a DNA purification kit (Axygen) and analyzed by qPCR with corresponding primers. The primer sequences are provided in Supplementary information.

### Preparation and detection of lEVs and sEVs

lEVs and sEVs were obtained with differential centrifugation as previously described^11^. Cells were cultured in DMEM containing 10% sEV-depleted FBS for 36 h. The supernatant of cultured cells was collected and centrifuged at 300*g* for 10 min at 4 °C to remove cells. Recycled supernatant was then centrifuged at 2,000*g* for 20 min at 4 °C to remove cell debris. Collected supernatant was centrifuged at 16,500*g* for 30 min at 4 °C to get lEV pellets. The remaining supernatant underwent ultracentrifugation at 100,000*g* for 90 min at 4 °C to isolate sEVs. Then, the obtained lEV and sEV pellets were washed once with PBS and resuspended in appropriate buffers. The homogeneity and size of lEVs and sEVs were detected by dynamic light scattering (DLS), and the structural and morphological features were visualized by transmission electron microscopy. OptiPrep density gradient centrifugation was performed as previously described with minor modifications^19,29^. lEVs isolated by ultracentrifugation were loaded onto a seven-step OptiPrep gradient consisting of 30%, 25%, 20%, 15%, 10%, 5% and 2.5% iodixanol in 20 mM HEPES (pH 7.2), 150 mM NaCl, 1 mM Na_3_VO_4_ and 50 mM NaF. Separation was performed by ultracentrifugation at 100,000*g* for 4 h. Seven fractions were collected and washed in PBS by another spin at 100,000*g* for 1 h and resuspended in appropriate buffer.

lEV isolation from plasma was performed as previously described^11^.

### Isolation and detection of palmitoylated proteins

Isolation of palmitoylated proteins was performed as previously described^55^. In brief, cells were lysed in buffer B (2.5% SDS, 1 mM EDTA and 100 mM HEPES (pH 7.5)). For the detection of protein palmitoylation by resin-assisted capture of S-acylated proteins (Acyl-RAC) assay, the free thiol group was blocked by incubating with 0.1% MMTS at 42 °C for 15 min. Proteins were precipitated with threefold prechilled acetone at −20 °C for 2 h. Precipitants were washed with 70% cold acetone three times, then resuspended in 0.7 ml of buffer C (1% SDS, 1 mM EDTA and 100 mM HEPES (pH 7.5)). Samples were divided into two tubes (0.3 ml each), and incubated with 20 μl of thiopropyl Sepharose 6B in the presence of 40 μl of 2 M NaCl or NH_2_OH at 26 °C for 3 h. The palmitoylated Cys residues of proteins were specifically released by NH_2_OH (NaCl was used as a negative control) and subsequently captured by thiopropyl Sepharose 6B. Beads were washed five times with buffer C containing 8 M urea and then eluted with buffer C containing 50 mM dithiothreitol (DTT) for 20 min at room temperature. Eluted fractions were mixed with SDS loading buffer and incubated at 37 °C for 1 h, then subjected to western blot.

### Mass spectrometry

lEVs and sEVs were extracted and washed with PBS three times. Then, 1% sodium deoxycholate solution was used to dissolve vesicles. A total of 30 μg of protein solution was prepared for label-free mass spectrometric detection. Proteins were analyzed with TripleTOF 5600+ (AB Sciex) or timsTOF Pro (Bruker). The data obtained were analyzed using Peaks Studio X software (Bioinformatics Solutions Inc.) and searched against the human UniProt Reference Proteome with isoforms.

### Mouse models

C57BL/6J and *Gfap-Cre* (C57BL/6J background, strain no. 012886) mice were obtained from the Jackson Laboratory. HK1-flox mice (*Hk1*^*f/f*^, C57BL/6J background, strain no. T052189) and *Lrat-Cre* mice (*Lrat-P2A-iCre*, C57BL/6J background, strain no. T006205) were purchased from GemPharmatech. Nur77-flox mice (*Nur77*^*f/f*^, C57BL/6J background) were generated by the Model Animal Research Center of Nanjing University. In brief, single guide RNA (sgRNA) directs Cas9 endonuclease cleavage in intron 1-2 and the downstream sequence of exon 7 of the *Nur77* gene, and results in loxp sites being inserted in intron 1-2 and the downstream sequence of exon 7 by homologous recombination. All mice were maintained at the Laboratory Animal Center of Xiamen University. Mice were housed on a standard condition, with a temperature of 22–24 °C, controlled 12 h/12 h light/dark cycle and humidity of 60%, with free access to food and water. All mouse experiments were approved by the Animal Ethics Committee of Xiamen University, and all tumor burdens did not exceed the permission of the Animal Ethics Committee of Xiamen University.

For the CCl_4_-induced liver fibrosis mouse model, 10∼12-week-old male mice were intraperitoneally injected with 20% CCl_4_ (dissolved in 0.5 ml of corn oil per kg body weight) twice a week for 4 weeks.

The DEN/CCl_4_-induced hepatocarcinoma mouse model was performed as previously described^32^. In brief, 15-day-old male mice were intraperitoneally injected once with DEN (dissolved in PBS, 25 mg kg^−1^). One week later, the mice were intraperitoneally injected with 10% CCl_4_ (dissolved in 0.5 ml of corn oil per kg body weight) weekly until the indicated time. Mice in the control group were intraperitoneally injected with 0.5 ml of corn oil per kg body weight.

The STZ/HFD-induced hepatocarcinoma mouse model was performed as previously described^32^. In brief, 2-day-old male mice were intraperitoneally injected with STZ (dissolved in citric acid buffer, 200 μg per mouse). Four weeks later, mice were fed with HFD (60% of calories from fat; Research Diets, Inc.) until 18 weeks of age.

For the orthotopic HCC model, 2 × 10^6^ Hepa1-6 cells were orthotopically injected into the subcapsular region of the left liver lobe in 8-week-old male and female mice. The mice were then euthanized around 30 days after inoculation for necropsy. In lEV-injected mouse models, lEVs were extracted from 20 dishes (Φ150 mm) of immortalized moHSCs (about 1 × 10^7^ cells per dish), and lEVs containing approximately 5 μg of protein were injected into mice via the tail vein every 3 days.

For lung metastatic experiments, luciferase-expressing Hepa1-6 cells (2 × 10^6^) were intravenously inoculated into 8-week-old male and female C57BL/6 mice. One week later, mice were injected with lEVs derived from control or HK1 knockdown moHSCs every 3 days via tail vein for 3 weeks. Mice were intraperitoneally injected with 3 mg of D-luciferin (15 mg ml^−1^ in PBS) 10 min before collection. Tumor metastases were detected by an IVIS Lumina II system (Caliper Life Sciences).

### Isolation of different primary cells from mice

Primary mouse HSCs were isolated as previously described^56^. In brief, male or female mice were anesthetized, and abdominal cavities were opened. The liver was perfused with Hanks’ Buffered Salt Solution (HBSS) through portal veins, followed with pronase E and collagenase perfusion. The liver was then gently removed with sterile forceps and transferred into sterile dishes. The hepatic capsule was teared apart, and the liver was gently shaken to release cells. The cells were suspended in solution containing pronase E, collagenase and DNase, then digested at 37 °C for 20 min. Hepatocytes were isolated using two rounds of centrifugation at 50*g* for 3 min. The supernatant was further centrifuged to obtain HSCs at 450*g* for 8 min, then precipitates were suspended with 18% Nycodenz solution to prepare density gradient centrifugation with 12% Nycodenz, 8.2% Nycodenz and Gey’s Balanced Salt Solution (GBSS). Eventually, cells were purified from the layer of 8.2% Nycodenz to obtain HSCs.

Primary liver macrophages were isolated as described^57^. In brief, mice were perfused with pronase-collagenase, and the cell suspension was prepared as above. The cell suspension was centrifuged at 50*g* for 3 min to separate hepatocytes (pellet) and nonparenchymal cells (NPCs; supernatant). The NPC suspension was centrifuged at 163*g* for 7 min, then the NPC pellet was resuspended with 1 ml of ACK lysis buffer to lyse red blood cells. Cells were washed with PBS, and the cell suspension was centrifuged at 163*g* for 7 min to repellet NPCs. Then, NPCs were stained for FACS, and liver macrophages were identified as CD45^+^, F4/80^hi^ and CD11b^int^ cells.

Primary HCC cells were isolated from fresh HCC samples in male mice. Liver tumors were dissected from killed mice and washed with PBS in plates. Tumor tissues were minced into ~1 mm fragments, and 0.05% collagenase solution was added for digestion at 37 °C for 30 min. The cell solutions were filtered through meshes, then centrifuged at 300*g* for 5 min. Pellets were resuspended in fresh DMEM supplemented with 10% FBS. The cell types are mixed at the beginning and become uniform after five or six cell passages.

CAFs were isolated as previously mentioned^58^. Fresh HCC samples from male mice were washed with serum-free DMEM and minced into small pieces of approximately 0.2 mm × 0.2 mm. The minced samples were incubated in fresh DMEM supplemented with 10% FBS for 24 h to allow samples to attach to the culture plates. The unattached cells were discarded, and the remaining cells were cultured on plates for 2 weeks. During this period, culture medium was replenished once every other day until CAFs started to grow out.

### Subcellular fractionation

Cells were lysed on ice for 10 min in 0.5 ml of hypotonic NP-40 buffer (10 mM HEPES (pH 7.9), 0.1 mM EGTA, 0.1 mM EDTA, 10 mM KCl, 0.15% NP-40 and protease inhibitors). Cell lysates were centrifuged at 3,000*g* for 5 min to prepare the supernatant (cytoplasmic fraction). The pellets containing the nuclei were washed with NP-40 buffer three times, sonicated, and then resuspended in SDS lysis buffer (50 mM Tris-HCl (pH 8.0), 1% SDS, 10 mM EDTA and protease inhibitors).

The plasma membrane fractions from cells were extracted using the Plasma Membrane Protein Extraction Kit according to the manufacturer’s instructions.

Mitochondria isolation was performed using a Mitochondria Isolation Kit (Thermo Fisher Scientific) according to the manufacturer’s instructions.

### Hexokinase activity assay

Cells were lysed on ice with lysis buffer (150 mM NaCl, 100 mM NaF, 50 mM Tris-HCl, 0.5% Nonidet P-40 and 1 mM phenylmethylsulfonyl fluoride (PMSF)), then centrifuged at 12,000*g* for 30 min at 4 °C. Supernatants were incubated with antibody and protein G-Sepharose beads for 3 h. Then, the protein G-Sepharose beads were washed with lysis buffer three times. Hexokinase protein was eluted with 3× Flag peptide. A total of 50 μl of eluents was added to 2× reaction buffer (200 mM Tris-HCl (pH 8.0), 1 mM EDTA, 20 mM ATP, 20 mM MgCl_2_, 4 mM glucose, 0.2 mM NADP^+^ and 0.2 U ml^−1^ glucose-6-phosphate dehydrogenase (G6PD)). HK activity was determined by following G6PD-dependent conversion of NADP^+^ to NADPH spectrophotometrically at 340 nm.

### Immunofluorescence

Immunofluorescence was performed as previously described^11^. Cells were fixed with 4% paraformaldehyde (PFA), followed with blocking buffer (0.2% Triton X-100 and 3% BSA in PBS). Fixed cells were incubated with corresponding primary antibodies at 4 °C overnight. Samples were washed with washing buffer (0.05% Triton X-100 and 0.2% BSA in PBS), and then incubated with Alexa Fluor 594-conjugated or Alexa Fluor 488-conjugated secondary antibodies at room temperature for 1 h. Samples were stained with 4′,6-diamidino-2-phenylindole (DAPI; 50 μg ml^−1^) at room temperature for 10 min. Images were captured by a Zeiss LSM 780 confocal microscope.

### Immunohistochemical staining and scoring

A thin section (5 μm) was deparaffinized and rehydrated with xylene and ethanol at different concentrations (100%, 95%, 80%, 70% and 50%), followed by washing in double-distilled water (ddH_2_O). The section was submitted to antigen retrieval by microwaving in the 10 mM sodium citrate buffer (pH 6.0) for 10 min. Subsequently, the section was blocked in 10% normal goat serum or kit of mouse-on-mouse immunodetection for 1 h at room temperature, followed by incubation with primary antibody overnight at 4 °C. Then, peroxidase-labeled polymer and substrate-chromogen were used to visualize the staining of the protein of interest.

Immunohistochemical staining was quantitated using the immunoreactive score (IRS) system as previously described^32^. IRS was calculated by a combination between staining intensity score (0–3) and proportion score (0–4). Intensity score was evaluated with the average intensity of staining (0, no staining; 1, yellow; 2, claybank; 3, tawny). Proportion score was evaluated with the percentage of positive-staining cells (0, no positive cells; 1, <10% positive cells; 2, 10–50% positive cells; 3, 51–80% positive cells; 4, >80% positive cells). Every sample was evaluated by three people in a blinded manner.

### In situ hybridization

The *Hk1* coding sequence was cloned into a pGEM-T vector with the forward primer 5′-ACCTTTGTCCGGTCCATTCC and reverse primer 5′-AGGGATCCCCGGTCTAACTC. The vector was used to synthesize a digoxigenin (DIG) riboprobe with Sp6 or T7 polymerase. Liver sections (15 μm) were fixed with 4% PFA and washed with DEPC-PBS. Sections were permeabilized by digesting with proteinase K for 10 min, then digestion was stopped with glycine. After sections were prehybridized at 65 °C for 2 h, sections were hybridized with 200 ng ml^−1^ DIG-labeled probe in hybridization buffer overnight at 65 °C. Sections were washed and incubated with blocking reagent at room temperature for 1 h. The blocking reagent was replaced with anti-DIG-AP solution, and sections were incubated overnight at 4 °C. After washing with PBST, sections were stained with AP-conjugated anti-DIG antibodies. AP reaction products were visualized by NBT-BCIP (dark purple).

### Metabolic assays

For the glucose uptake detection, cells were incubated with 2-(*N*-(7-nitrobenz-2-oxa-1,3-diazol-4-yl)amino)-2-deoxyglucose (2-NBDG; 20 μM), a fluorescent indicator for direct glucose uptake detection, at 37 °C for 1 h. The uptake of 2-NBDG was measured by flow cytometry.

For the ECAR detection, HCC cells plated in XF96 plates were incubated with different lEVs for 12 h, then acclimatized at 37 °C for 1 h in XF Base Medium with 2 mM glutamine. Measurement was performed under basal conditions and in response to 10 mM glucose, 5 μM oligomycin and 100 mM 2-DG using a Seahorse Biosciences XF96 analyzer, with the following assay conditions: 3 min of mixture, 3 min of waiting and 3 min of measurement.

Lactate production assays were performed as previously described^59^. In brief, LX-2 cells were cultured in DMEM without FBS for 12 h. Culture media were collected, and an aliquot of 540 μl of culture media of each sample was mixed with 60 μl of deuterium oxide (D_2_O) in 5 mm magnetic resonance tubes. Data were collected using a Bruker Avance III 600 MHz NMR magnet system.

### Luciferase reporter assay

Cells were transfected with luciferase reporter vector, and plasmids encoding β-galactosidase (β-gal) and corresponding proteins. At 24 h after transfection, cells were lysed, and the luciferase activities and β-gal activities were measured. β-gal activity was used to normalize for transfection efficiency.

### Glutathione S-transferase (GST) pull-down assay

GST pull-down assays were carried out as previously described^39^. GST-Nur77 LBD or its point mutants, and His-Akt were expressed in *Escherichia coli* strain BL21, and purified using glutathione ­Sepharose (Thermo Fisher) or Ni-­NTA agarose (Qiagen), respectively. The bead­-bound GST­-fusion proteins (2 μg) were incubated with His-­tagged protein (1 μg) in the presence or absence of PDNPA (10 μM) in 1 ml of modified ELB (50 mM Tris­-HCl, pH 7.6, 100 mM NaF, 150 mM NaCl, 0.5% Nonidet P-­40 and 1 mM PMSF) at 4 °C for 1 h. Beads were washed with modified ELB three times, and resuspended in SDS loading buffer for western blotting.

### Statistical analysis

Data in this study were presented as the mean ± s.e.m. Two-tailed Student’s *t*-test was applied for statistical analysis between two groups, and analyses of variance (ANOVAs) were applied for multiple group comparisons (followed by either Tukey’s or Sidak’s multiple comparison tests). The statistical analysis was performed using GraphPad Prism 7. *P* < 0.05 was considered statistically significant, *P* < 0.01 was considered highly significant and *P* < 0.001 was considered extremely significant.

### Reporting summary

Further information on research design is available in the Nature Research Reporting Summary linked to this article.

## Supplementary information

Reporting Summary

Supplementary Table 1 Information about primer sequences and oligonucleotide sequences.

Supplementary Table 2 Information about patients with cirrhosis.

Supplementary Table 3 Quantification of western blots in figures.

Supplementary Table 4 Quantification of western blots in Extended Data figures.

## Data Availability

Source data that support the plots within this manuscript are provided with this paper. The mass spectrometry proteomics data have been deposited to the ProteomeXchange Consortium (http://proteomecentral.proteomexchange.org) via the iProX partner repository^60^ with the dataset identifier PXD035911. All materials and reagents are available from the corresponding author upon reasonable request. Source data are provided with this paper.

## Source data

Source Data Fig. 1 Statistical source data.
Source Data Fig. 1 Unprocessed western blots.
Source Data Fig. 2 Statistical source data.
Source Data Fig. 2 Unprocessed western blots.
Source Data Fig. 3 Statistical source data.
Source Data Fig. 4 Statistical source data.
Source Data Fig. 5 Statistical source data.
Source Data Fig. 5 Unprocessed western blots.
Source Data Fig. 6 Statistical source data.
Source Data Fig. 6 Unprocessed western blots.
Source Data Extended Data Fig. 1 Statistical source data.
Source Data Extended Data Fig. 1 Unprocessed western blots.
Source Data Extended Data Fig. 2 Statistical source data.
Source Data Extended Data Fig. 2 Unprocessed western blots.
Source Data Extended Data Fig. 3 Statistical source data.
Source Data Extended Data Fig. 3 Unprocessed western blots.
Source Data Extended Data Fig. 4 Statistical source data.
Source Data Extended Data Fig. 4 Unprocessed western blots.
Source Data Extended Data Fig. 5 Statistical source data.
Source Data Extended Data Fig. 5 Unprocessed western blots.
Source Data Extended Data Fig. 6 Unprocessed western blots.
