# Supplementary material for: HK1 from hepatic stellate cell–derived extracellular vesicles promotes progression of hepatocellular carcinoma
Source: Nat Metab. 2022 Oct 3;4(10):1306–21. doi: 10.1038/s42255-022-00642-5 (PMC9584821; doi:10.1038/s42255-022-00642-5)
Supplement: Supplementary file 1 — Reporting Summary [file 42255_2022_642_MOESM1_ESM.pdf]

nature portfolio

Corresponding author(s): Qiao Wu, Hang-zi Chen

Last updated by author(s): Aug 14, 2022

Reporting Summary

Nature Portfolio wishes to improve the reproducibility of the work that we publish. This form provides structure for consistency and transparency in reporting. For further information on Nature Portfolio policies, see our Editorial Policies and the Editorial Policy Checklist.

Statistics

For all statistical analyses, confirm that the following items are present in the figure legend, table legend, main text, or Methods section.

n/a	Confirmed	
☐	☒	The exact sample size (n) for each experimental group/condition, given as a discrete number and unit of measurement
☐	☒	A statement on whether measurements were taken from distinct samples or whether the same sample was measured repeatedly
☐	☒	The statistical test(s) used AND whether they are one- or two-sided<br>*Only common tests should be described solely by name; describe more complex techniques in the Methods section.*
☐	☒	A description of all covariates tested
☐	☒	A description of any assumptions or corrections, such as tests of normality and adjustment for multiple comparisons
☐	☒	A full description of the statistical parameters including central tendency (e.g. means) or other basic estimates (e.g. regression coefficient) AND variation (e.g. standard deviation) or associated estimates of uncertainty (e.g. confidence intervals)
☐	☒	For null hypothesis testing, the test statistic (e.g. F, t, r) with confidence intervals, effect sizes, degrees of freedom and P value noted<br>*Give P values as exact values whenever suitable.*
☒	☐	For Bayesian analysis, information on the choice of priors and Markov chain Monte Carlo settings
☒	☐	For hierarchical and complex designs, identification of the appropriate level for tests and full reporting of outcomes
☒	☐	Estimates of effect sizes (e.g. Cohen's d, Pearson's r), indicating how they were calculated

Our web collection on statistics for biologists contains articles on many of the points above.

Software and code

Policy information about availability of computer code

Data collection	Confocal images were acquired by Zeiss LSM 780 confocal microscope. Images of Immunohistochemical were gained by Leica Application Suite X (LAS X). The results of Real-Time RCR were obtained by CFX96 Touch Real-Time PCR Detection System. AB Sciex TripleTOF 5600+ and Bruker timsTOF Pro were used to acquire MS results. ECAR results were collected by Seahorse Biosciences XF96 analyzer Wave 2.6.1 (North Billerica, MA, USA). Tumor metastases were detected by an IVIS@ Lumina II system (Caliper Life Sciences, Hopkinton, MA, USA). Bruker Avance III 600Mz NMR magnet system (Bruker, Billerica, MA, USA) was used to collect the data of lactate production.
Data analysis	Confocal images were analysed by ZEN 2.3 (blue edition). All statistical analyses were performed and P values were obtained using the GraphPad Prism software 7. Docking between LBD of Nur77 and Akt was performed by Schrödinger Computational Suite, Maestro Version 11.5.011, MMshare Version 4.1.011, Release 2018-1, Platform Linux-x86_64. ProteinPilot software (version 5.0) was used for AB Sciex TripleTOF 5600+ and Peaks Studio (version X+) was used for results analyses of Bruker timsTOF Pro .

For manuscripts utilizing custom algorithms or software that are central to the research but not yet described in published literature, software must be made available to editors and reviewers. We strongly encourage code deposition in a community repository (e.g. GitHub). See the Nature Portfolio guidelines for submitting code & software for further information.

Data

Policy information about availability of data

All manuscripts must include a data availability statement. This statement should provide the following information, where applicable:

- Accession codes, unique identifiers, or web links for publicly available datasets
- A description of any restrictions on data availability
- For clinical datasets or third party data, please ensure that the statement adheres to our policy

All source data that support the plots of this study are provided within this paper. The mass spectrometry proteomics data have been deposited to the ProteomeXchange Consortium (http://proteomecentral.proteomexchange.org) via the iProX partner repository60 with the dataset identifier PXD035911. All other materials and reagents are available from the corresponding author upon reasonable request.

Field-specific reporting

Please select the one below that is the best fit for your research. If you are not sure, read the appropriate sections before making your selection.

- [x] Life sciences
- [ ] Behavioural & social sciences
- [ ] Ecological, evolutionary & environmental sciences

For a reference copy of the document with all sections, see nature.com/documents/nr-reporting-summary-flat.pdf

Life sciences study design

All studies must disclose on these points even when the disclosure is negative.

Sample size	No statistical method were used to determine sample size. For cell experiments study, generally three independent samples were performed for data analyses, which is standard in this filed. For mouse model study, samples size was based on previously published study using similar methodologies. (Hou. P P et al. Ectosomal PKM2 Promotes HCC by Inducing Macrophage Differentiation and Remodeling the Tumor Microenvironment. Molecular cell 78, 1192-1206.e1110 (2020), doi: 10.1016/j.molcel.2020.05.004)
Data exclusions	No data were excluded from analyses.
Replication	Reproducibility of findings was verified using biological replicates and independent experiments and all exact n values were indicated in figure legends.
Randomization	For cell experiments, cells from same source were allocated to different dished and then treated with corresponding conditions. For all of the animal experiments, mice were randomized into different groups with approximately equivalent numbers before treatment.
Blinding	Investigators involved in histopathology analysis and immunohistochemistry analyses were blinded. Investigators were not blinded for most in vivo and vitro experiments since they need to perform these experiments with different treatment.

Reporting for specific materials, systems and methods

We require information from authors about some types of materials, experimental systems and methods used in many studies. Here, indicate whether each material, system or method listed is relevant to your study. If you are not sure if a list item applies to your research, read the appropriate section before selecting a response.

Materials & experimental systems

n/a	Involved in the study	
☐	☒	Antibodies
☐	☒	Eukaryotic cell lines
☒	☐	Palaeontology and archaeology
☐	☒	Animals and other organisms
☐	☒	Human research participants
☒	☐	Clinical data
☒	☐	Dual use research of concern

Methods

n/a	Involved in the study	
☒	☐	ChIP-seq
☒	☐	Flow cytometry
☒	☐	MRI-based neuroimaging

Antibodies

Antibodies used	The goat anti-rabbit Alexa Fluor 594 (A-11037, 1:200 for IF), goat anti-rabbit (31210, 1:5000 for IB) and anti-mouse (31160, 1:5000 for IB) antibodies were purchased from Thermo Fisher Scientific (Bremen, Germany). Anti-Flag (F3165, 1:5000 for IB), anti-HA (H9658, 1:5000 for IB), and anti-tubulin (T4026, 1:5000 for IB) antibodies were purchased from Sigma (St. Louis, MO, USA). Anti-HK1 (2024, 1:5000 for IB), anti-HK2 (2867, 1:5000 for IB), anti-Flotillin-2 (3436, 1:5000 for IB), anti-Nur77 (3960, 1:1000 for IB), anti-PARP (9532, 1:2000 for IB), anti-Akt (pan) (2920, 1:2500 for IB), anti-phospho-Akt (Ser473) (3787, 1:2500 for IB), anti-phospho-Akt (Thr308)

(2965, 1:2500 for IB) and anti-mouse IgG Alexa Fluor 488 (4408, 1:200 for IF) antibodies were purchased from Cell Signaling Technology (Beverly, MA, USA). Anti-αSMA (ab124964, 1:10000 for IB), anti-Ki67 (ab16667, 1:200 for IHC), anti-LAMP1 (ab25630, 1:2500 for IB), anti-Annexin A1 (ab214486, 1:5000 for IB), anti-CD63 (ab134045, 1:5000 for IB) and anti-alpha 1 sodium potassium ATPase (ab7671, 1:5000 for IB) antibodies were purchased from Abcam (Cambridge, MA, USA). Anti-Hsp60 antibody (SC-376240, 1:5000 for IB and 1:200 for IF) was purchased from Santa Cruz Biotechnology (CA, USA). Anti-Nur77 (12235-1-AP, 1:200 for ChIP), anti-Annexin A2 (11256-1-AP, 1:5000 for IB) and anti-His (HRP-66005, 1:5000 for IB) antibodies were purchased from ProteinTech (Wuhan, Hubei, China). Anti-phospho-serine/threonine (612549, 1:1000 for IB) and anti-CD11b-PE (561098, 1:100 for FACS) antibodies were purchased from BD Biosciences (San Jose, CA, USA). Anti-BSG (A16662, 1:5000 for IB) was purchased from Abclonal technology (Wuhan, China). Anti-mouse Nur77 (14-5965-82, 1:1000 for IB), anti-CD45-eFluor 450 (48-0451-82, 1:100 for FACS) and anti-F4/80-FITC antibodies (11-4801-82, 1:100 for FACS) were purchased from eBioscience. Anti-DIG-AP (11093274910, 1:3000 for in situ hybridization) was purchased from Roche. The rabbit polyclonal antibody against ABHD17B (1:1000 for IB) was generated by immunizing rabbits with synthetic peptide corresponding to human ABHD17B (aa 50-64).

Validation

We verified the applicability of ABHD17B antibody by knockdown experiments in human and mouse hepatic stellate cells (Extended Data Fig. 2m-o). Other antibodies have been validated according to the manufacture's websites as following:
Goat anti-rabbit Alexa Fluor 594 (A-11037), https://www.thermofisher.cn/cn/zh/antibody/product/Goat-anti-Rabbit-IgG-H-L-Highly-Cross-Adsorbed-Secondary-Antibody-Polyclonal/A-11037.
Goat anti-rabbit (31210), https://www.thermofisher.cn/cn/zh/antibody/product/Goat-anti-Rabbit-IgG-H-L-Secondary-Antibody-Polyclonal/31210.
Goat anti-mouse (31160), https://www.thermofisher.cn/cn/zh/antibody/product/Goat-anti-Mouse-IgG-H-L-Secondary-Antibody-Polyclonal/31160.
Anti-Flag (F3165), https://www.sigmaaldrich.cn/CN/zh/product/sigma/f3165.
Anti-HA (H9658), https://www.sigmaaldrich.cn/CN/zh/product/sigma/h9658.
Anti-tubulin (T4026), https://www.sigmaaldrich.cn/CN/zh/product/sigma/t4026.
Anti-HK1 (2024), https://www.cellsignal.cn/products/primary-antibodies/hexokinase-i-c35c4-rabbit-mab/2024?site-search-type=Products&N=4294956287&Ntt=2024&fromPage=plp&_requestid=4015346.
Anti-HK2 (2867), https://www.cellsignal.cn/products/primary-antibodies/hexokinase-ii-c64g5-rabbit-mab/2867?site-search-type=Products&N=4294956287&Ntt=2867&fromPage=plp&_requestid=4015514.
Anti-Flotillin-2 (3436), https://www.cellsignal.cn/products/primary-antibodies/flotillin-2-c42a3-rabbit-mab/3436?site-search-type=Products&N=4294956287&Ntt=3436&fromPage=plp&_requestid=4015662.
Anti-Nur77 (3960), https://www.cellsignal.cn/products/primary-antibodies/nur77-d63c5-xp-rabbit-mab/3960?_=1660278031027&Ntt=3960&tahead=true.
Anti-PARP (9532), https://www.cellsignal.cn/products/primary-antibodies/parp-46d11-rabbit-mab/9532?site-search-type=Products&N=4294956287&Ntt=9532&fromPage=plp&_requestid=4015949.
Anti-Akt (pan) (2920), https://www.cellsignal.cn/products/primary-antibodies/akt-pan-40d4-mouse-mab/2920?_=1660278175035&Ntt=2920&tahead=true.
Anti-phospho-Akt (Ser473) (3787), https://www.cellsignal.cn/products/primary-antibodies/phospho-akt-ser473-736e11-rabbit-mab/3787?site-search-type=Products&N=4294956287&Ntt=3787&fromPage=plp&_requestid=4016155.
Anti-phospho-Akt (Thr308) (2965), https://www.cellsignal.cn/products/primary-antibodies/phospho-akt-thr308-c31e5e-rabbit-mab/2965?_=1660278262432&Ntt=2965&tahead=true.
Anti-mouse IgG Alexa Fluor 488 (4408), https://www.cellsignal.cn/products/secondary-antibodies/anti-mouse-igg-h-l-f-ab-2-fragment-alexa-fluor-488-conjugate/4408?_=1660278321049&Ntt=4408&tahead=true.
Anti--SMA (ab124964), https://www.abcam.com/alpha-smooth-muscle-Actin-antibody-EPR5368-ab124964.html.
Anti-Ki67 (ab16667), https://www.abcam.com/ki67-antibody-sp6-ab16667.html.
Anti-LAMP1 (ab25630), https://www.abcam.com/lamp1-antibody-h4a3-ab25630.html.
Anti-Annexin A1 (ab214486), https://www.abcam.com/annexin-a1anxa1-antibody-epr19342-bsa-and-azide-free-ab222398.html.
Anti-CD63 (ab134045), https://www.abcam.com/cd63-antibody-epr5702-ab134045.html.
Anti-alpha 1 sodium potassium ATPase (ab7671), https://www.abcam.com/alpha-1-sodium-potassium-atpase-antibody-4646-ab7671.html.
Anti-Hsp60 antibody (SC-376240), https://www.scbt.com/zh/p/hsp-60-antibody-c-10.
Anti-Nur77 (12235-1-AP), https://www.ptgcn.com/products/NR4A1-Antibody-12235-1-AP.htm.
Anti-Annexin A2 (11256-1-AP), https://www.ptgcn.com/products/ANXA2-Antibody-11256-1-AP.htm.
Anti-His (HRP-66005), https://www.ptgcn.com/products/6-His,-His-Tag-Antibody-HRP-66005.htm.
Anti-phospho-serine/threonine (612549), https://www.bdbiosciences.com/en-us/products/reagents/microscopy-imaging-reagents/immunofluorescence-reagents/purified-mouse-anti-phosphoserine-threonine.612549.
Anti-CD11b-PE (561098), https://www.bdbiosciences.com/en-us/products/reagents/flow-cytometry-reagents/research-reagents/single-color-antibodies-ruo/pe-cy-7-rat-anti-cd11b.561098.
Anti-BSG (A16662), https://ap.abclonal.com/catalog-antibodies/CD147BSGRabbitpAb/A16662.
Anti-mouse Nur77 (14-5965-82), https://www.thermofisher.cn/cn/zh/antibody/product/Nur77-Antibody-clone-12-14-Monoclonal/14-5965-82.
Anti-CD45-eFluor 450 (48-0451-82), https://www.thermofisher.cn/cn/zh/antibody/product/CD45-Antibody-clone-30-F11-Monoclonal/48-0451-82.
Anti-F4/80-FITC antibodies (11-4801-82), https://www.thermofisher.cn/cn/zh/antibody/product/F4-80-Antibody-clone-BM8-Monoclonal/11-4801-82.
Anti-DIG-AP (11093274910), https://krackeler.com/catalog/sigma/ROCHE/11093274910.

Eukaryotic cell lines

Policy information about cell lines

Cell line source(s)

The human hepatic stellate cell line LX-2 (SCC064) was purchased from Millipore (Temecula, CA, USA). The human embryonic kidney cell line 293T (CRL-11268), the human hepatoma cell line HepG2 (HB-8065) and the mouse hepatoma cell line

	Hepa1-6 (CRL-1830) were obtained from American Type Culture Collection (Manassas, VA, USA). The human hepatoma cell line Huh7 (TCHu182) was purchased from Cell Bank in the Chinese Academy of Sciences (Shanghai, China). The human hepatoma cell line HLF was purchased from Meisen Chinese Tissue Culture Collections (Hangzhou, China).The mouse hapatic stellate cells (mo HSCs) was isolated from livers of BALB/c mice and immortalized spontaneously.
Authentication	Huh7 and HepG2 cells were authenticated by short tandem repeat (STR) profiling analysis by Guangzhou Cellcook Biological Science and Technology Ltd. Other cell lines used were not authenticated.
Mycoplasma contamination	Cells are negative for mycoplasma contamination.
Commonly misidentified lines (See ICLAC register)	There was no misidentified line in our study.

Animals and other organisms

Policy information about studies involving animals; ARRIVE guidelines recommended for reporting animal research

Laboratory animals	C57BL/6J and GFAP-Cre (C57BL/6J background, Strain No. 012886) mice were obtained from the Jackson Laboratory. HK1-flox mice (HK1f/f, C57BL/6J background, Strain No.: T052189) and Lrat-Cre mice (Lrat-P2A-iCre, C57BL/6J background, Strain No.: T006205) were purchased from GemPharmatech company (Nanjing, China). Nur77-flox mice (Nur77f/f, C57BL/6J background) were generated by Nanjing BioMedical Research, Institute of Nanjing University (Nanjing, China). Briefly, sgRNA direct Cas9 endonuclease cleavage in intron 1-2 and downstream sequence of exon 7 of Nur77 gene and create a double-strand break. Such breaks will be repaired by donor mediated homologous recombination, and result in loxp sites inserted in intron 1-2 and downstream sequence of exon 7 respectively by homologous recombination. Exon 2-7 would be removed via crossing with Cre-driver lines, leading to the disruption of Nur77. The transgenic mice were genotyped by PCR, followed by sequence analysis.<br>℃In the CCl4-induced liver fibrosis mouse model, 10-12 weeks aged male mice were conducted for the experiment. In orthotopic HCC model, we performed on balanced cohorts of 8-week around male and female mice, and no obvious phenotype difference was observed between male and female mice. For the DEN/CCl4- and the HFD/STZ-induced primary hepatocarcinoma mouse model, we conducted experiments on 15 days aged and 2 days aged male mice separately since male mice were reported to be more susceptible to development HCC than female mice.<br>Mice were housed on a standard condition, with 22-24℃° C, controlled light/dark cycle (12 hours light, 12 hours darkness) and humidity (60%) with free access to food and water.℃
Wild animals	No wild animal was used.
Field-collected samples	No field-collected sample was used.
Ethics oversight	All mice were maintained at Laboratory Animal Center in Xiamen University (Xiamen, China), in accordance with the institutional guidelines. All animal experiments were approved by the Animal Ethics Committee of Xiamen University (acceptance no. XMULAC20170294).

Note that full information on the approval of the study protocol must also be provided in the manuscript.

Human research participants

Policy information about studies involving human research participants

Population characteristics	Fresh blood samples and HCC carcinoma and para-carcinoma tissues were obtained from Zhongshan Hospital, Xiamen University. These samples were used for plasma lEVs isolation and immunofluorescence.<br>Blood samples were collected from cirrhosis patients (4 female and 14 male, the ages of patients range from 36 to 75 and the majority of them (13 of 18) range from 50 to 70) and 18 healthy donors (the information of healthy donors were not collected). No phenotype difference was observed between male and female patients.
Recruitment	Human blood samples were randomly collected from healthy donors and cirrhosis donors (4 female and 14 male). It seems no phenotype difference between male and female patients based on these samples, further more samples should be collected to conduct sex-based analysis.
Ethics oversight	All blood samples and fresh HCC carcinoma and para-carcinoma tissues were collected with patient informed consent and approval of the Medical Ethical Committee of Zhongshan Hospital.

Note that full information on the approval of the study protocol must also be provided in the manuscript.
