# Supplementary material for: HK1 from hepatic stellate cell–derived extracellular vesicles promotes progression of hepatocellular carcinoma
Source: Nat Metab. 2022 Oct 3;4(10):1306–21. doi: 10.1038/s42255-022-00642-5 (PMC9584821; doi:10.1038/s42255-022-00642-5)
Supplement: Source Data Fig. 1 — Unprocessed western blots. [file 42255_2022_642_MOESM7_ESM.pdf]

# Uncropped western blot images
# Figure 1

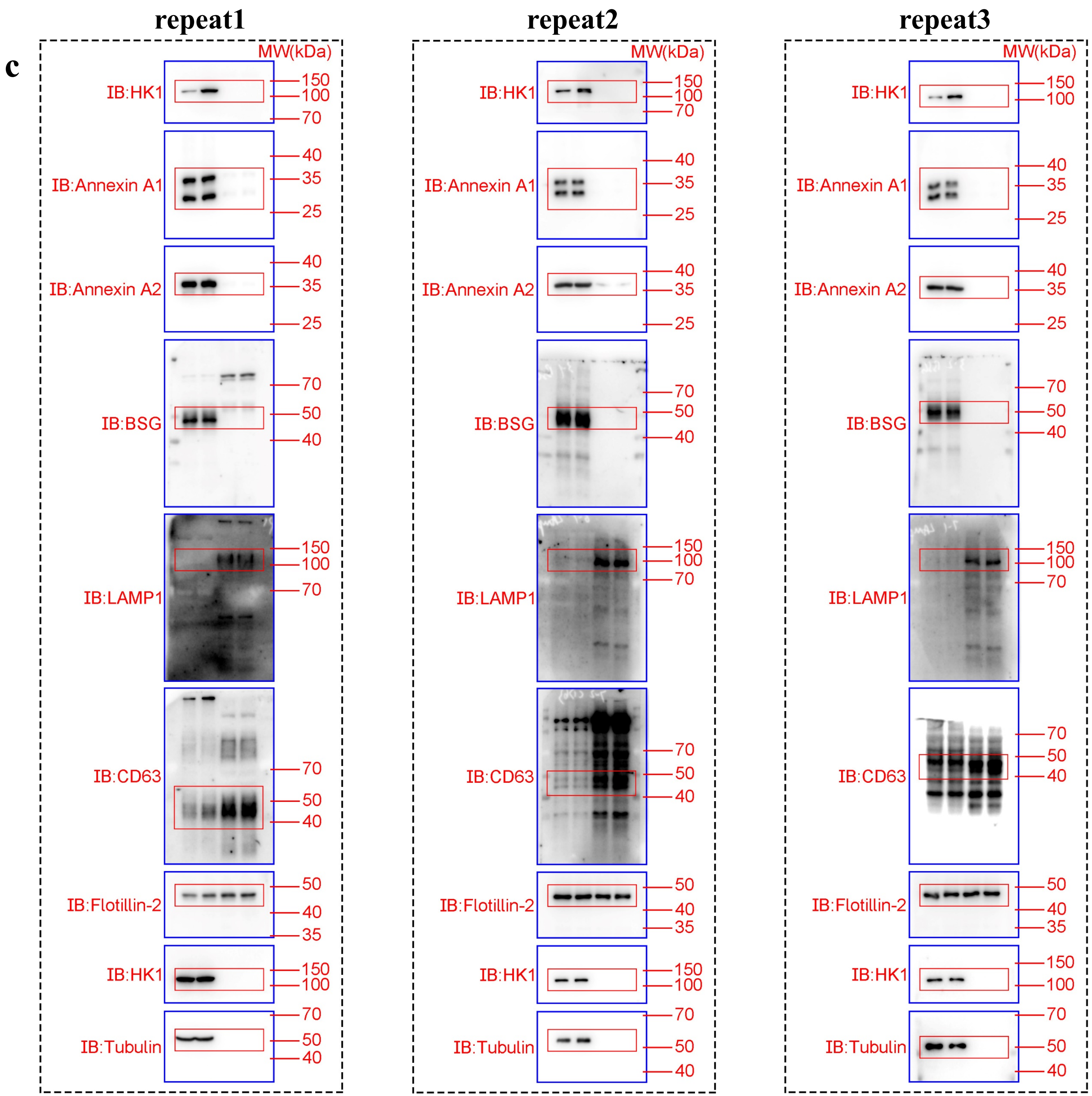

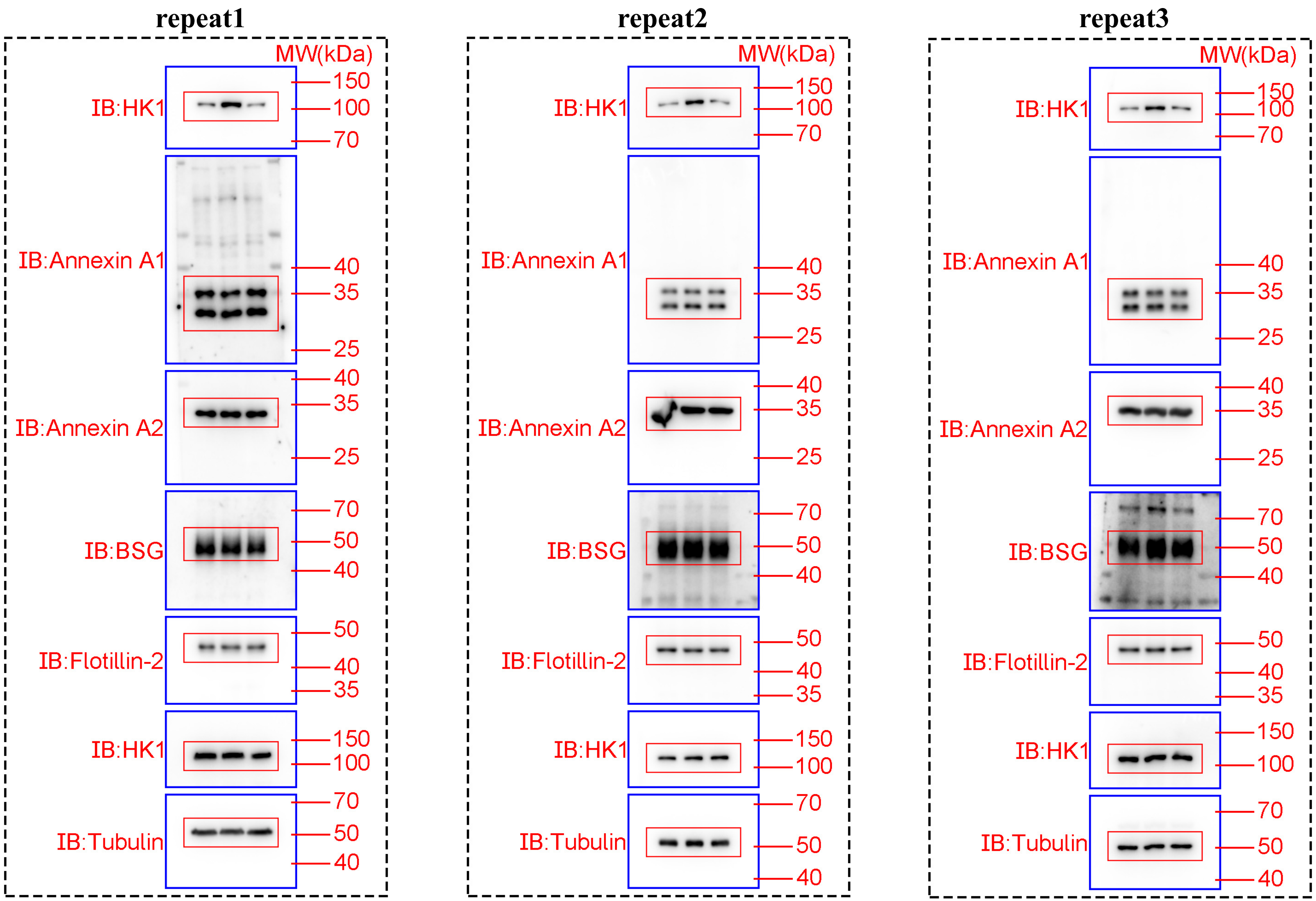

repeat1
repeat2
repeat3
MW(kDa)
IB:HK1
IB:Annexin A1
IB:Annexin A2
IB:BSG
IB:Flotillin-2
IB:Tubulin
150
100
70
40
35
25
50

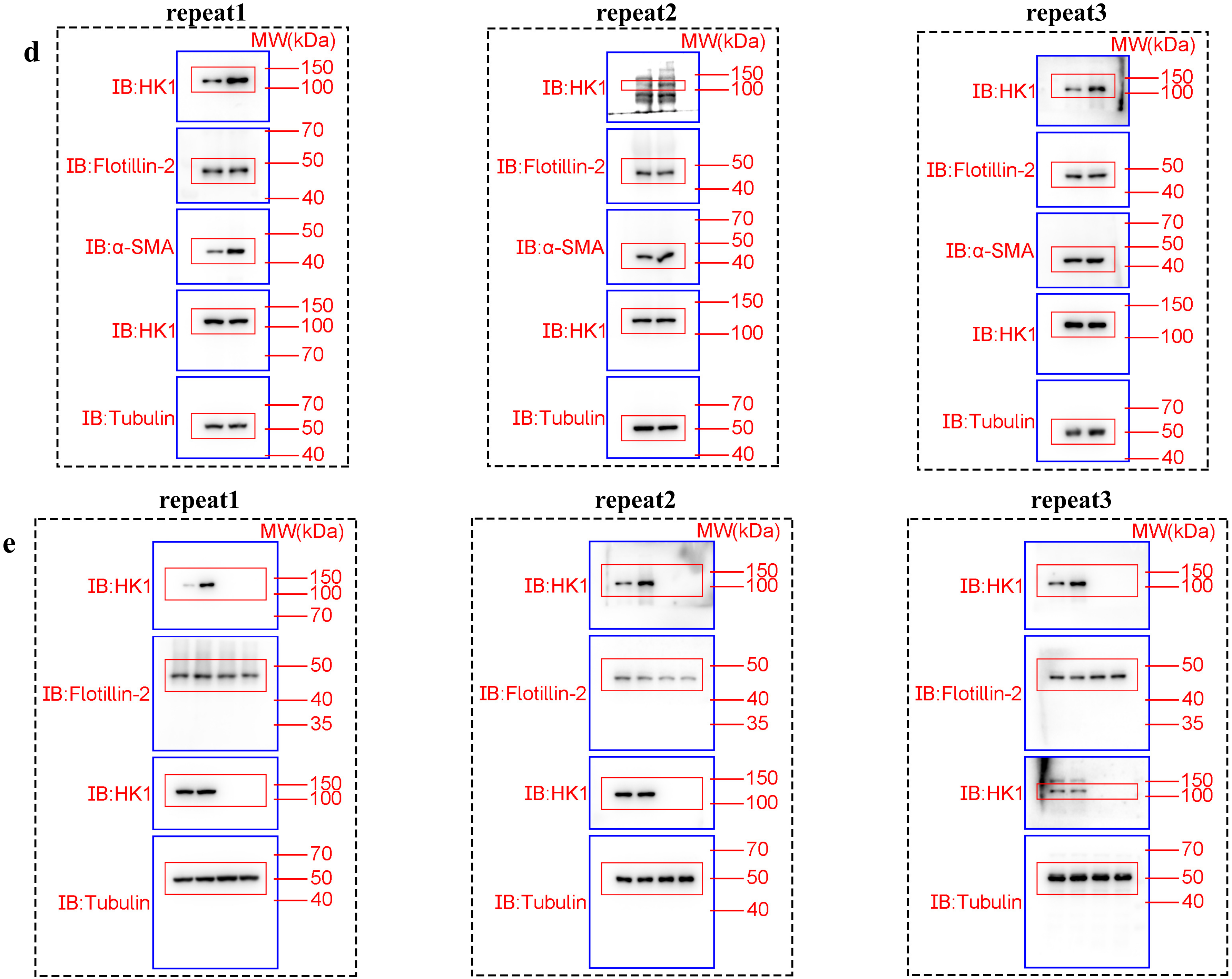
d
repeat1
MW(kDa)
IB:HK1
150
100
70
IB:Flotillin-2
50
40
IB:α-SMA
50
40
IB:HK1
150
100
70
IB:Tubulin
70
50
40
repeat2
MW(kDa)
IB:HK1
150
100
IB:Flotillin-2
50
40
IB:α-SMA
70
50
40
IB:HK1
150
100
IB:Tubulin
70
50
40
repeat3
MW(kDa)
IB:HK1
150
100
IB:Flotillin-2
50
40
IB:α-SMA
70
50
40
IB:HK1
150
100
IB:Tubulin
70
50
40
e
repeat1
MW(kDa)
IB:HK1
150
100
70
IB:Flotillin-2
50
40
35
IB:HK1
150
100
IB:Tubulin
70
50
40
repeat2
MW(kDa)
IB:HK1
150
100
IB:Flotillin-2
50
40
35
IB:HK1
150
100
IB:Tubulin
70
50
40
repeat3
MW(kDa)
IB:HK1
150
100
IB:Flotillin-2
50
40
35
IB:HK1
150
100
IB:Tubulin
70
50
40

f

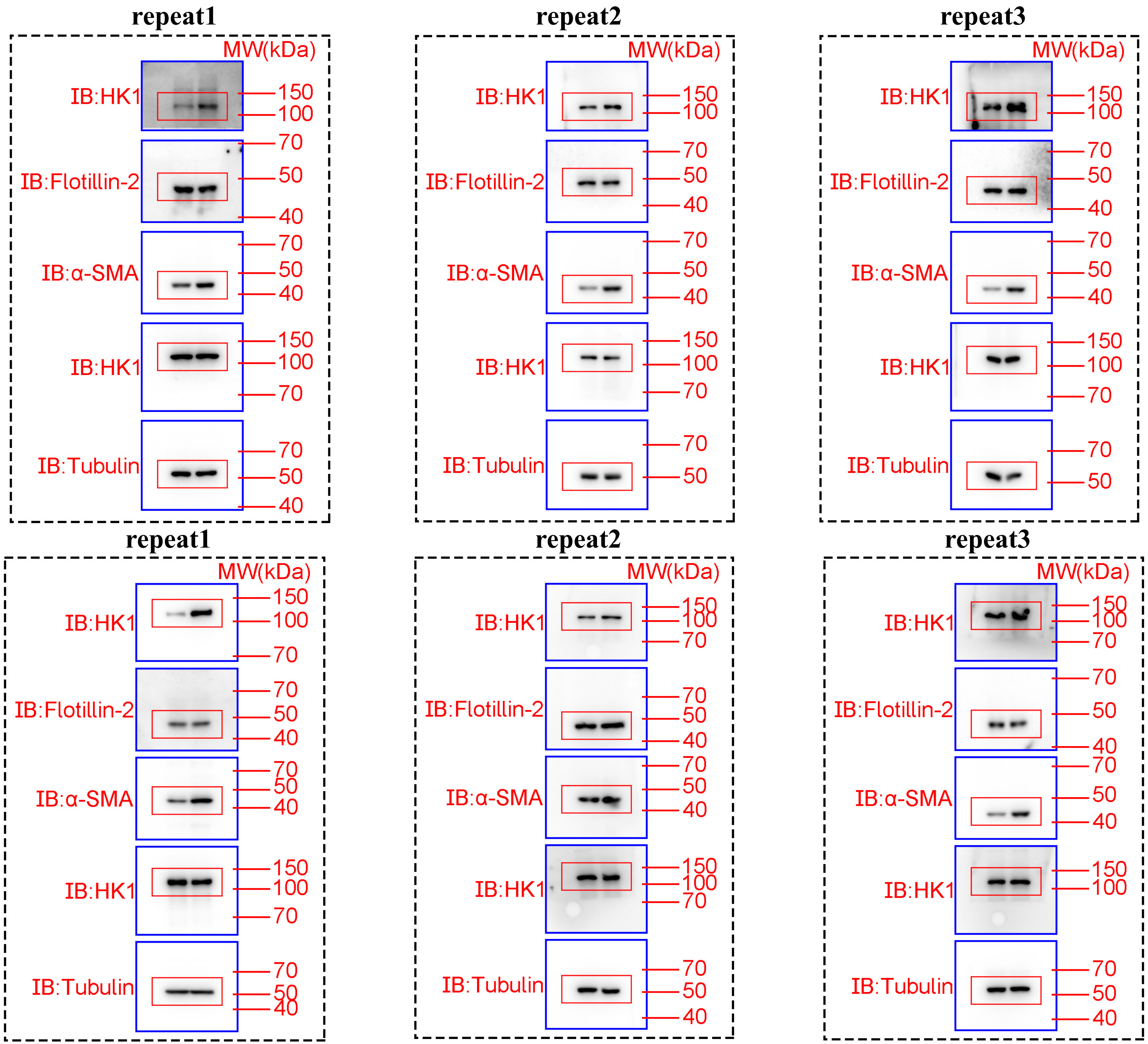

repeat1
repeat2
repeat3
MW(kDa)
IB:HK1
IB:Flotillin-2
IB:α-SMA
IB:HK1
IB:Tubulin
150
100
70
50
40

g

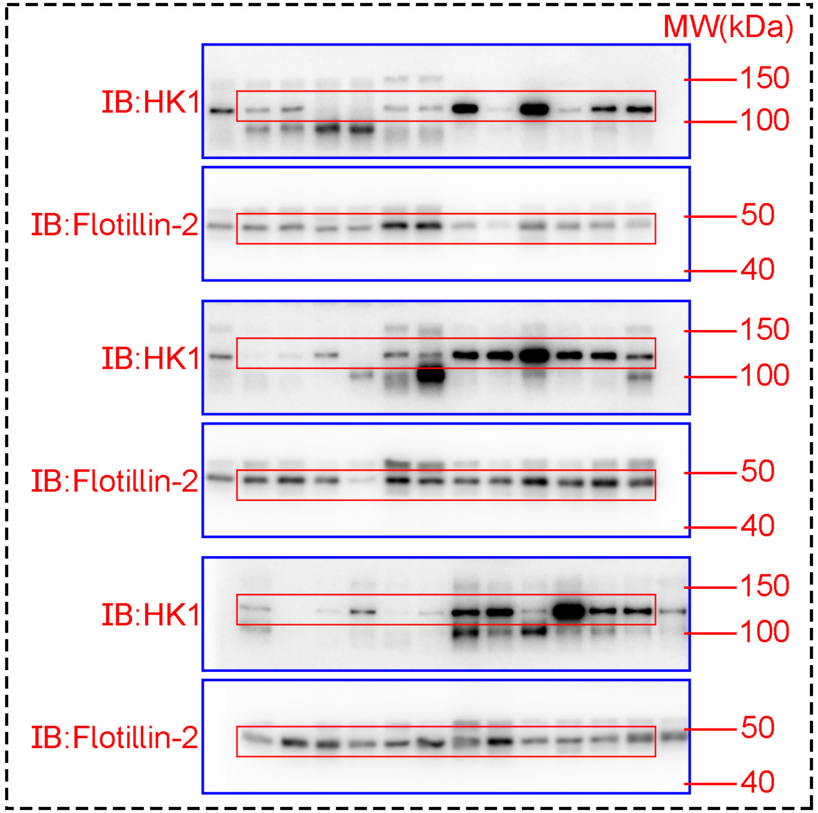

MW(kDa)
IB:HK1
150
100
IB:Flotillin-2
50
40
IB:HK1
150
100
IB:Flotillin-2
50
40
IB:HK1
150
100
IB:Flotillin-2
50
40


i

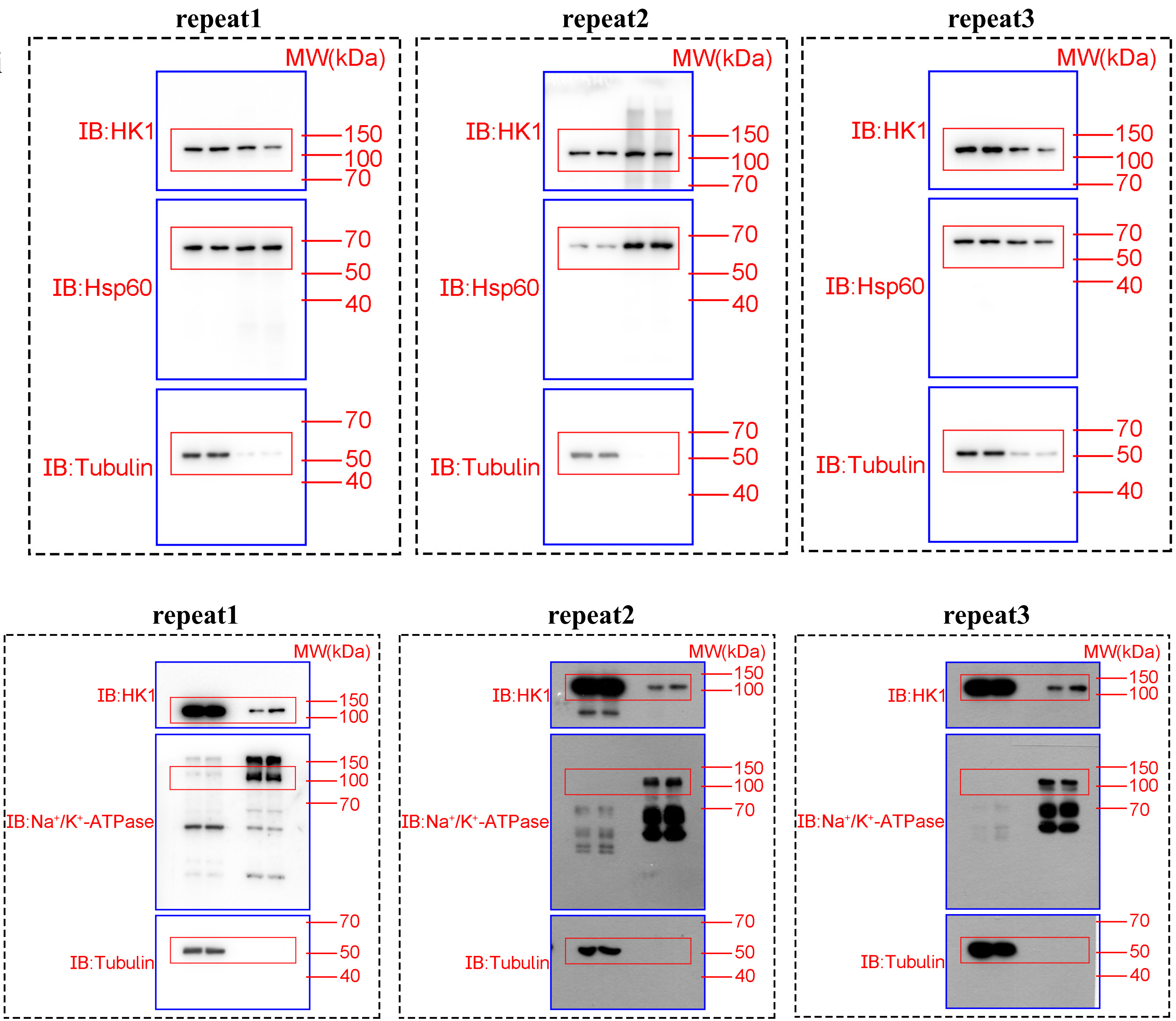

repeat1
repeat2
repeat3
MW(kDa)
IB:HK1
150
100
70
IB:Hsp60
70
50
40
IB:Tubulin
70
50
40
repeat1
repeat2
repeat3
MW(kDa)
IB:HK1
150
100
IB:Na+/K+-ATPase
150
100
70
IB:Tubulin
70
50
40

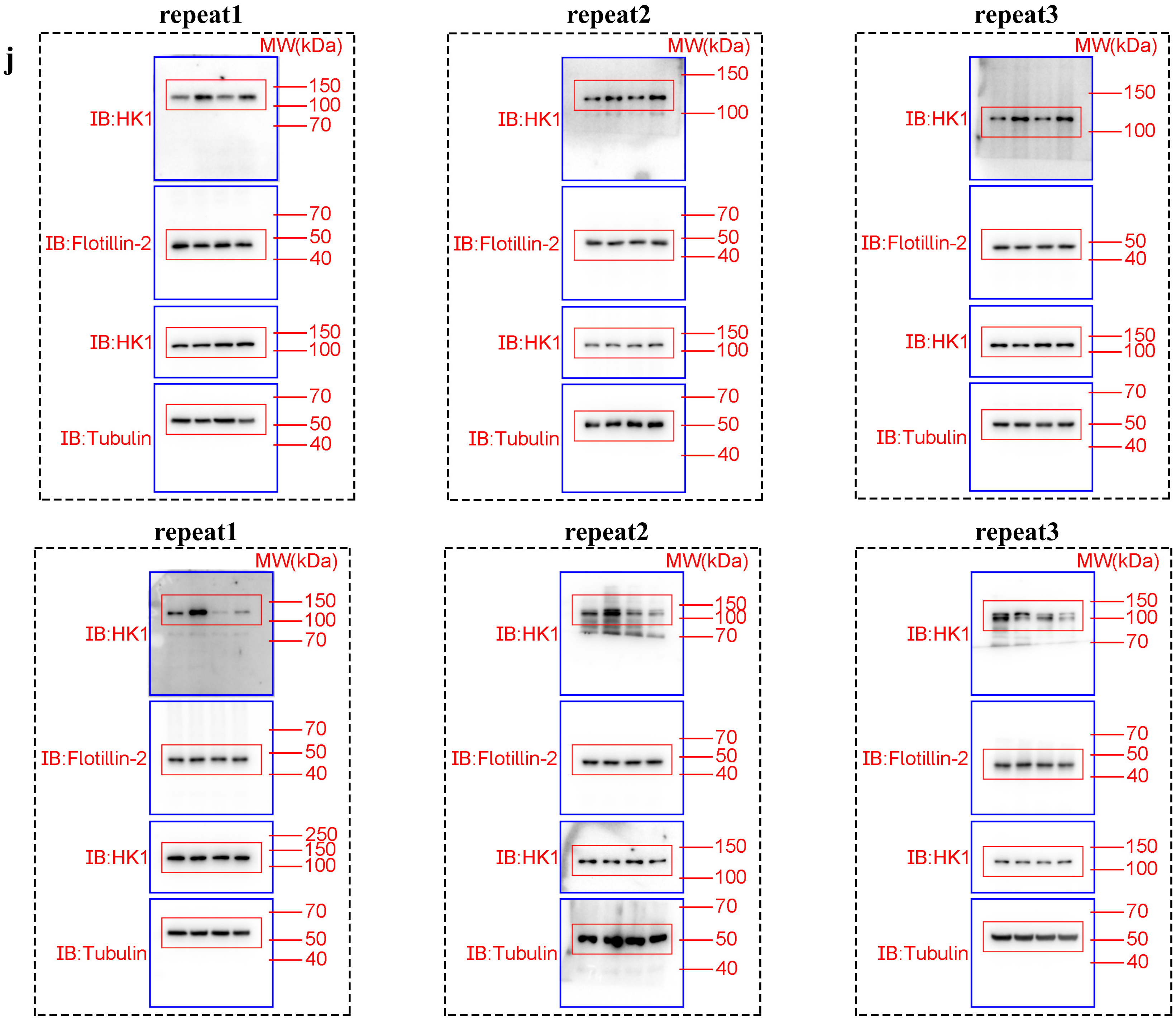
j
repeat1
repeat2
repeat3
MW(kDa)
IB:HK1
IB:Flotillin-2
IB:HK1
IB:Tubulin
150
100
70
50
40
250

k

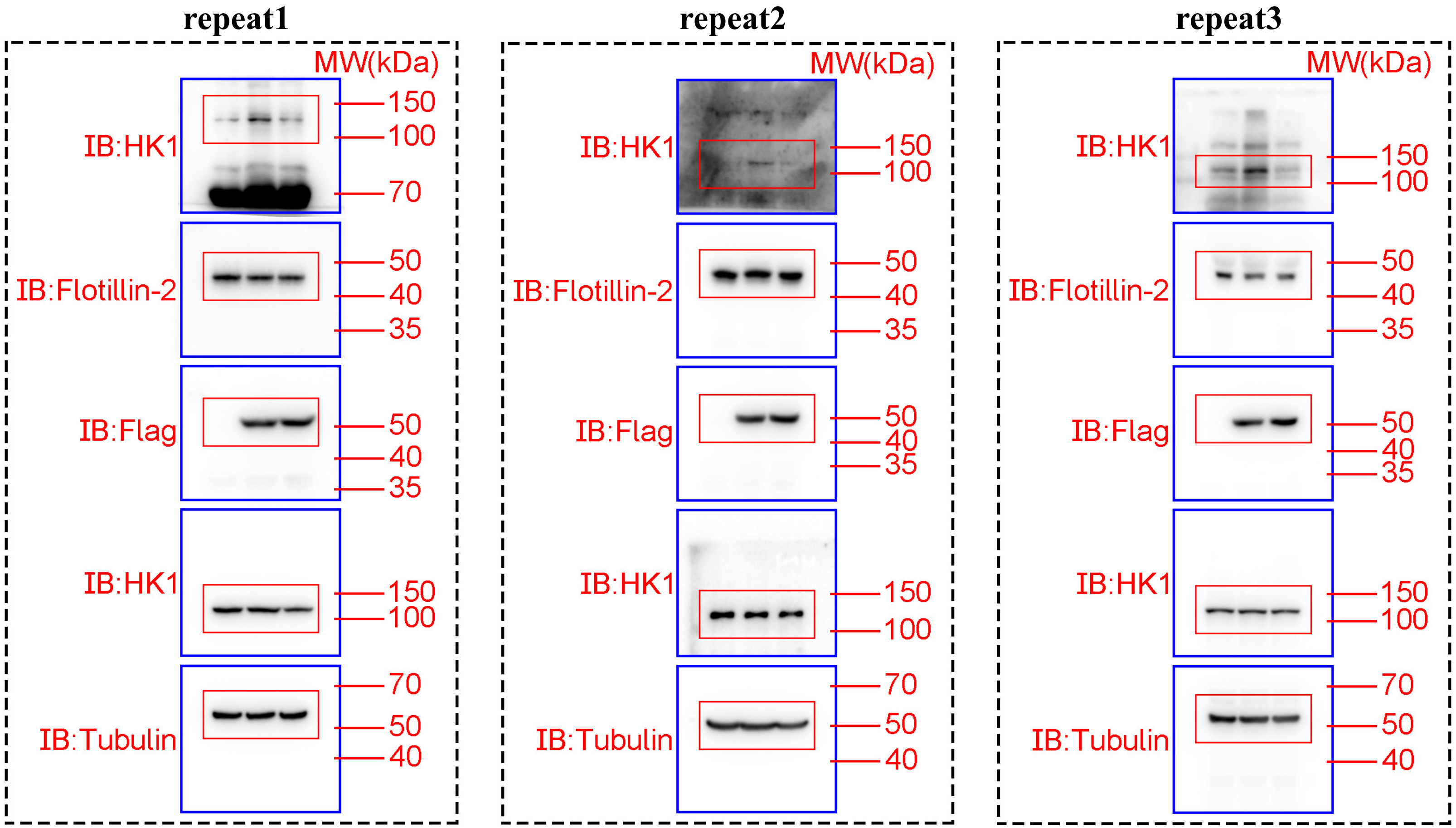

repeat1
repeat2
repeat3
MW(kDa)
IB:HK1
IB:Flotillin-2
IB:Flag
IB:HK1
IB:Tubulin
150
100
70
50
40
35
