# Supplementary material for: HK1 from hepatic stellate cell–derived extracellular vesicles promotes progression of hepatocellular carcinoma
Source: Nat Metab. 2022 Oct 3;4(10):1306–21. doi: 10.1038/s42255-022-00642-5 (PMC9584821; doi:10.1038/s42255-022-00642-5)
Supplement: Source Data Fig. 2 — Unprocessed western blots. [file 42255_2022_642_MOESM9_ESM.pdf]

Uncropped western blot images

Figure 2

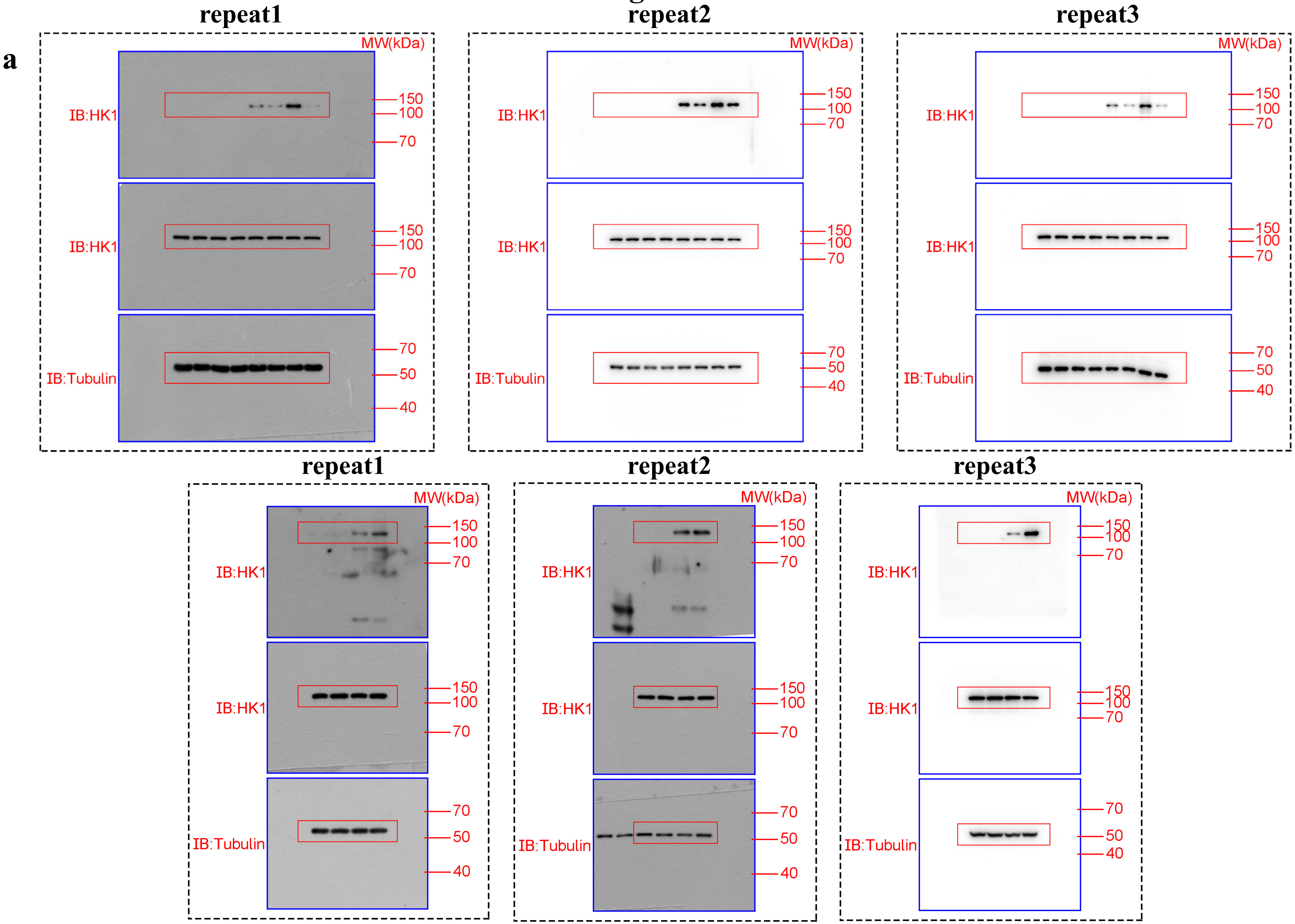

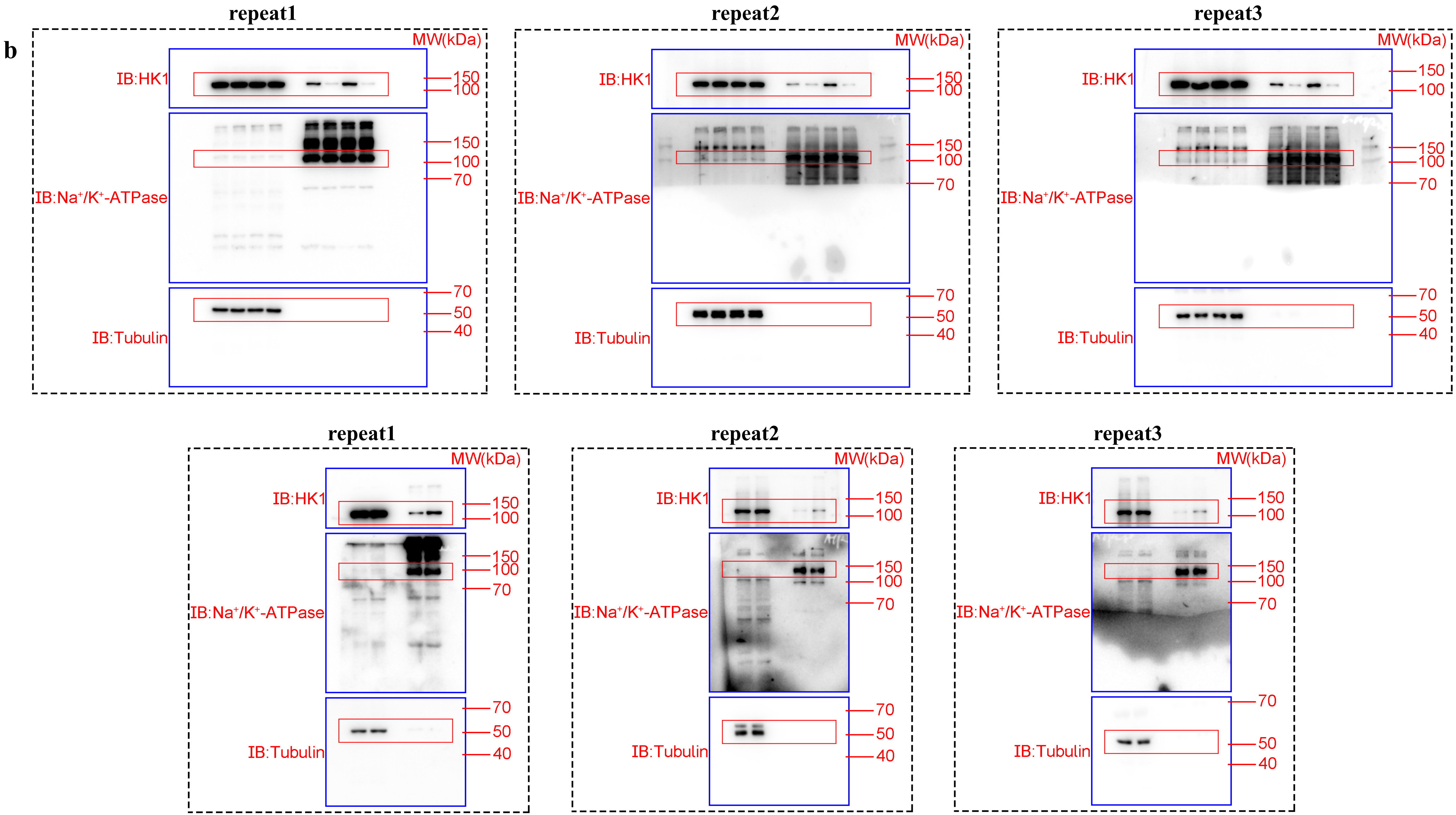
b
repeat1
repeat2
repeat3
MW(kDa)
IB:HK1
IB:Na+/K+-ATPase
IB:Tubulin
150
100
70
50
40
repeat1
repeat2
repeat3
MW(kDa)
IB:HK1
IB:Na+/K+-ATPase
IB:Tubulin
150
100
70
50
40

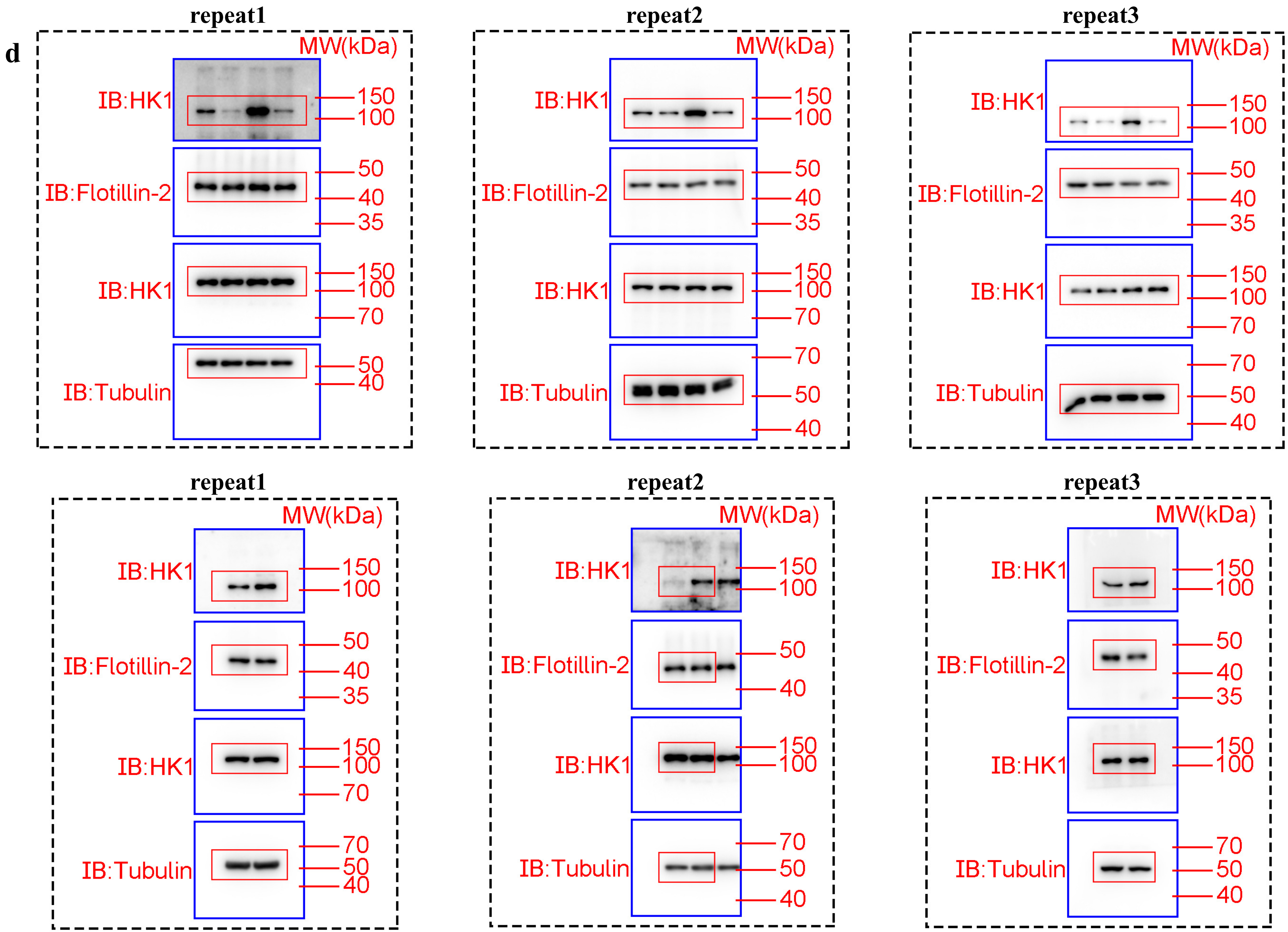
d
repeat1
repeat2
repeat3
MW(kDa)
IB:HK1
IB:Flotillin-2
IB:HK1
IB:Tubulin
150
100
50
40
35
70

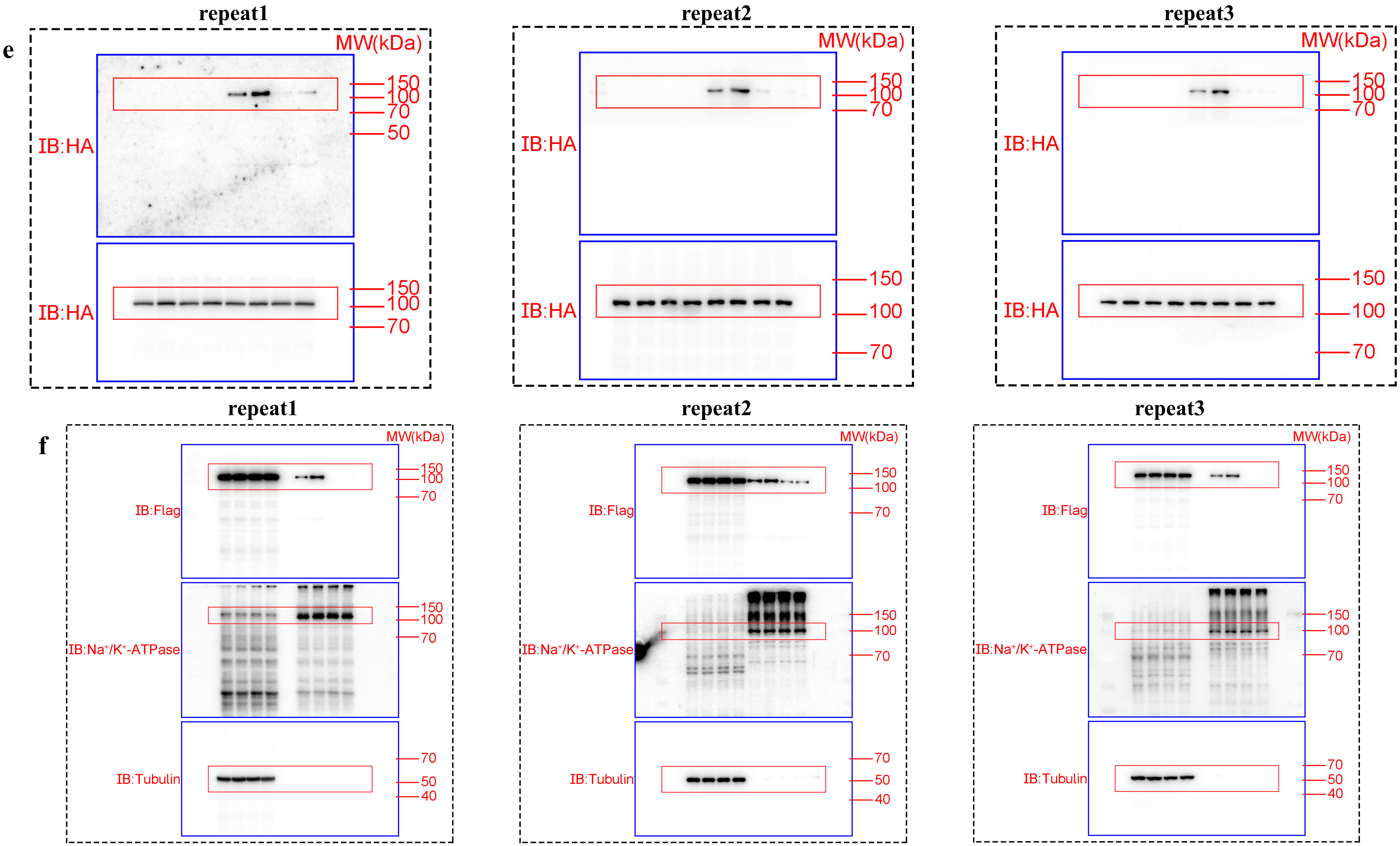

e
repeat1
repeat2
repeat3
MW(kDa)
IB:HA
150
100
70
50
f
IB:Flag
IB:Na+/K+-ATPase
IB:Tubulin
40

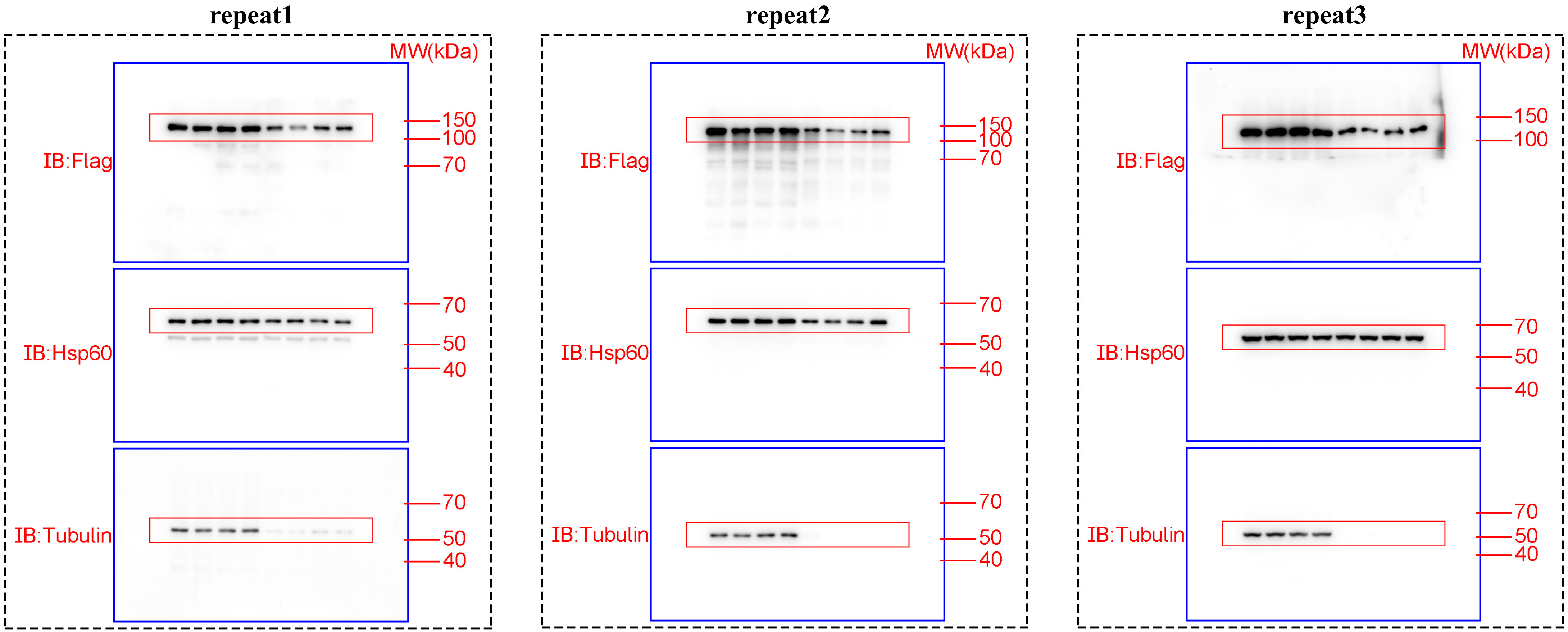

repeat1
repeat2
repeat3
MW(kDa)
IB:Flag
IB:Hsp60
IB:Tubulin
150
100
70
50
40

h

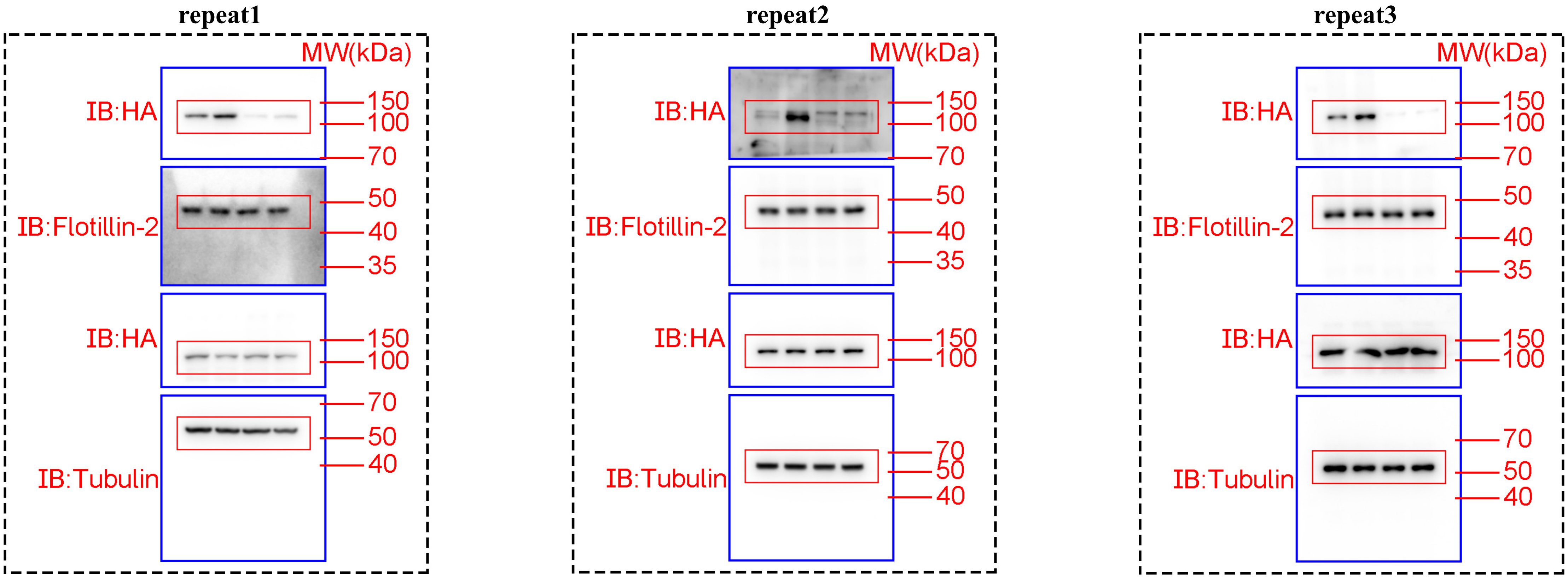

repeat1
repeat2
repeat3
MW(kDa)
IB:HA
IB:Flotillin-2
IB:HA
IB:Tubulin
150
100
70
50
40
35

j

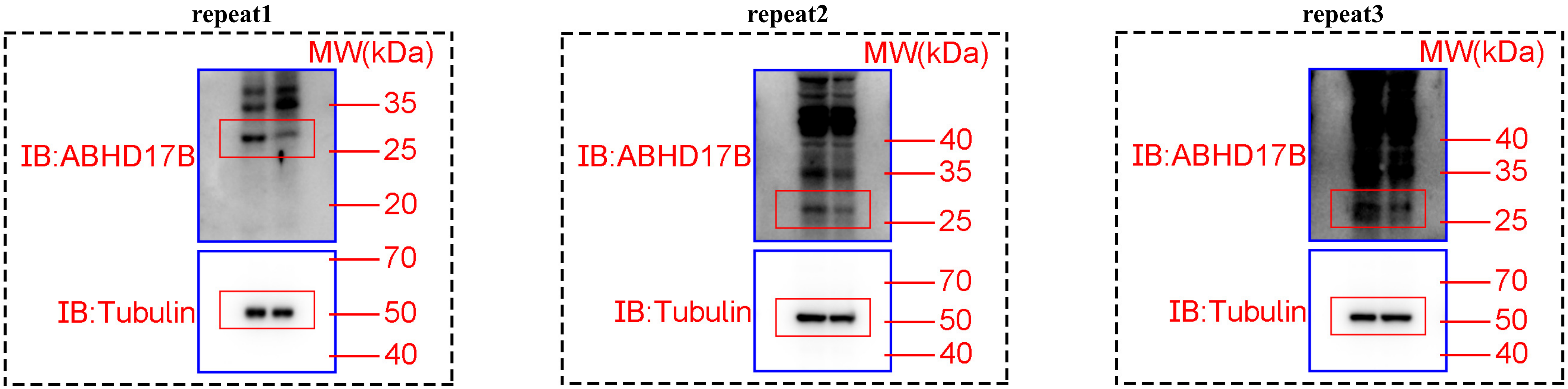

repeat1
repeat2
repeat3
MW(kDa)
IB:ABHD17B
IB:Tubulin
40
35
25
20
70
50

i

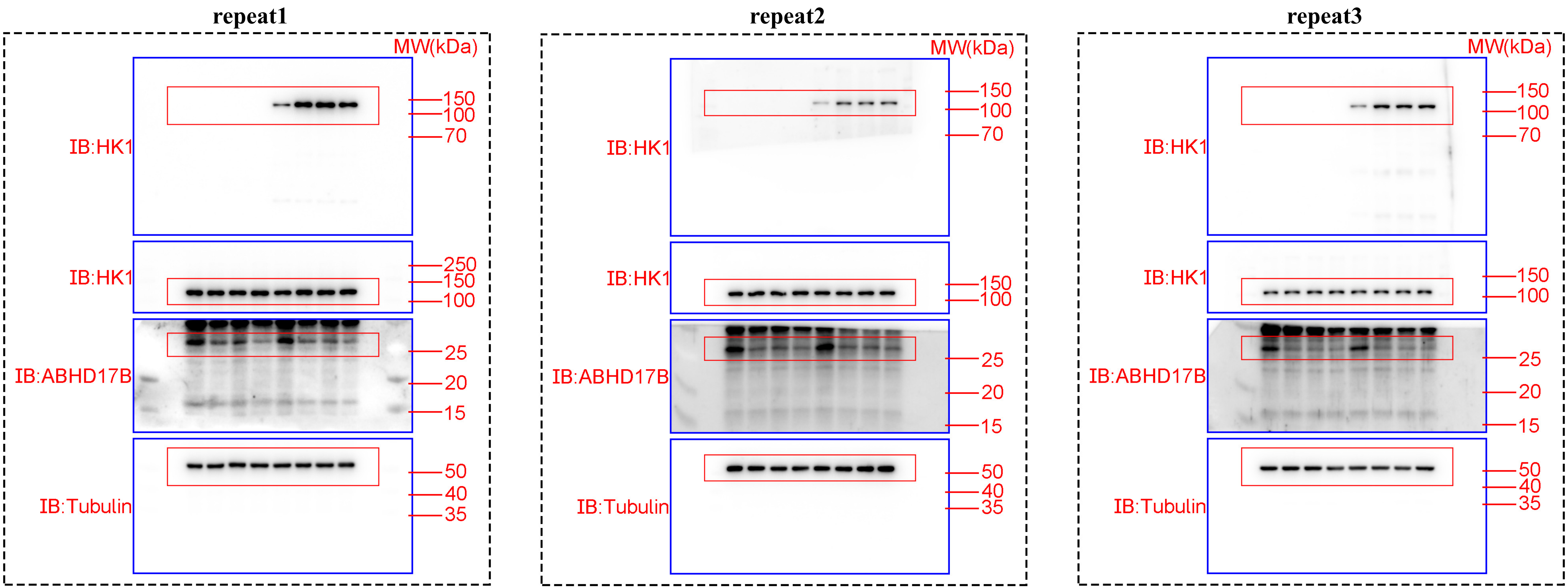
repeat1
repeat2
repeat3
MW(kDa)
IB:HK1
IB:HK1
IB:ABHD17B
IB:Tubulin
150
100
70
250
150
100
25
20
15
50
40
35

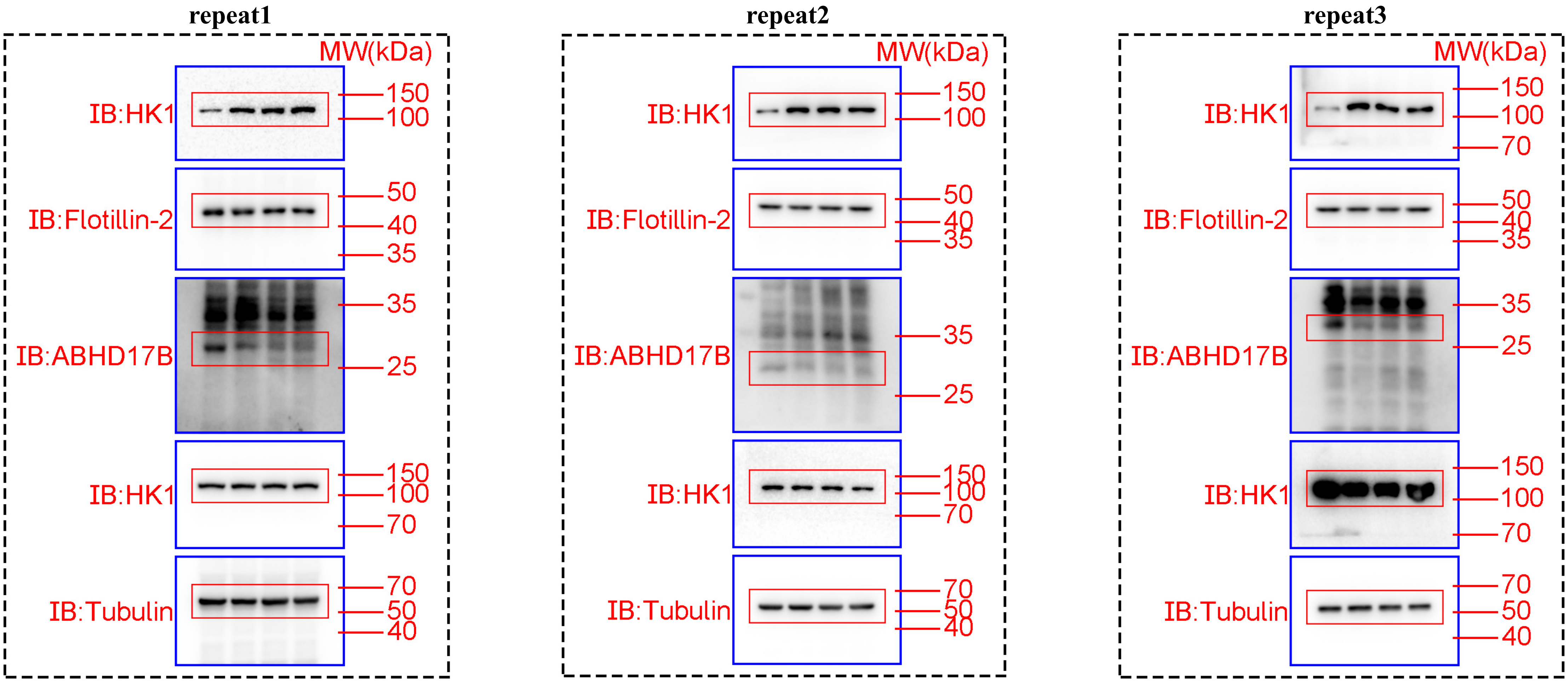
repeat1
MW(kDa)
IB:HK1
150
100
IB:Flotillin-2
50
40
35
IB:ABHD17B
35
25
IB:HK1
150
100
70
IB:Tubulin
70
50
40
repeat2
MW(kDa)
IB:HK1
150
100
IB:Flotillin-2
50
40
35
IB:ABHD17B
35
25
IB:HK1
150
100
70
IB:Tubulin
70
50
40
repeat3
MW(kDa)
IB:HK1
150
100
70
IB:Flotillin-2
50
40
35
IB:ABHD17B
35
25
IB:HK1
150
100
70
IB:Tubulin
70
50
40
