# Supplementary figures and images for: HK1 from hepatic stellate cell–derived extracellular vesicles promotes progression of hepatocellular carcinoma
Source: Nat Metab. 2022 Oct 3;4(10):1306–21. doi: 10.1038/s42255-022-00642-5 (PMC9584821; doi:10.1038/s42255-022-00642-5)

# Uncropped western blot images

Figure 5

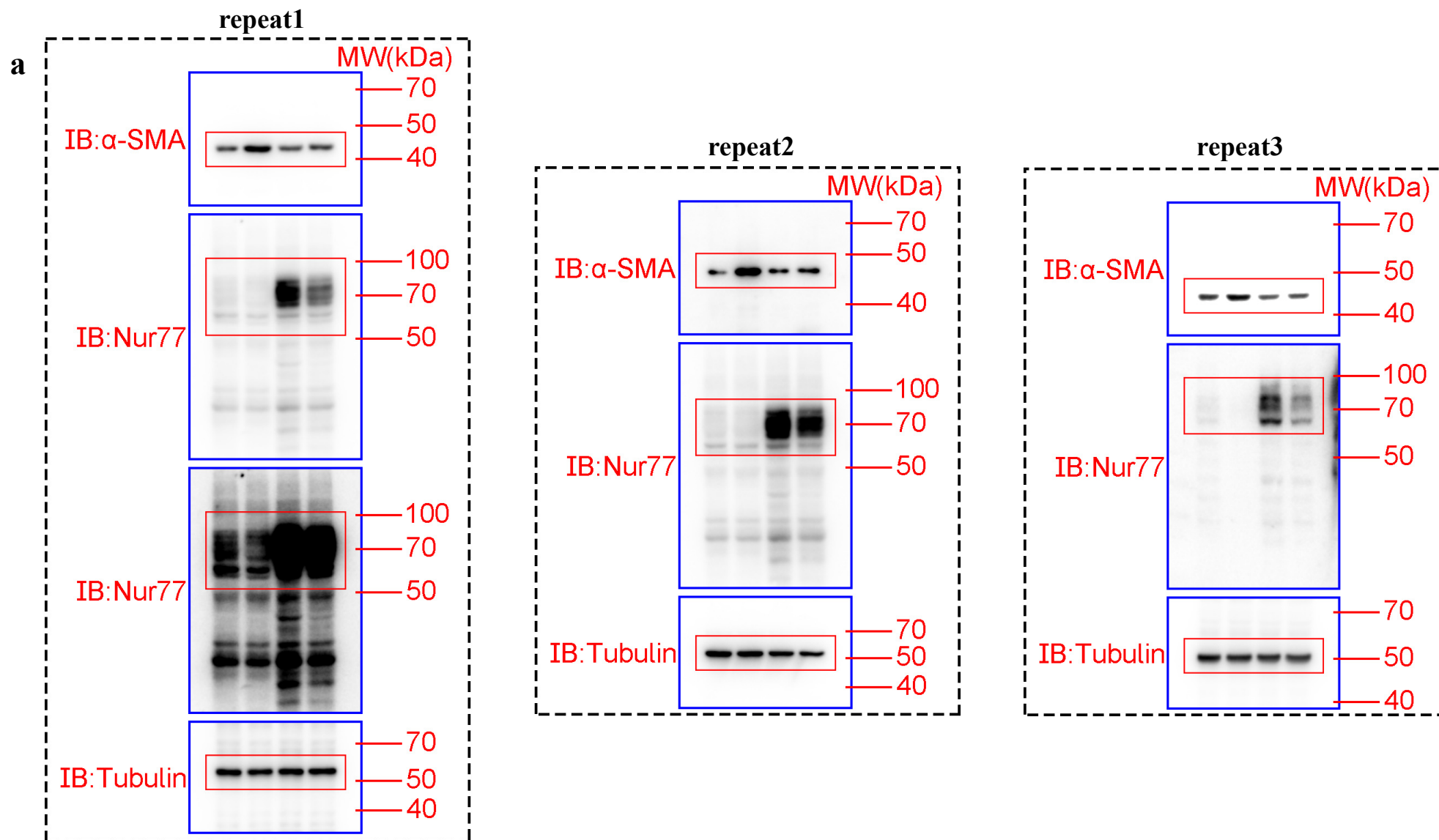

**b**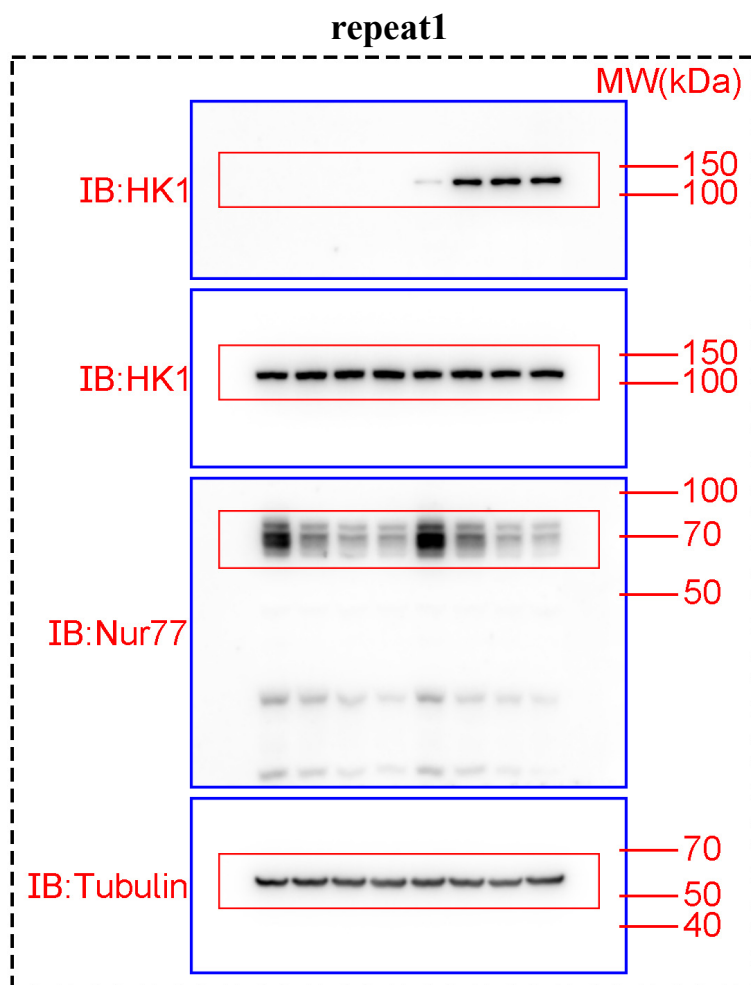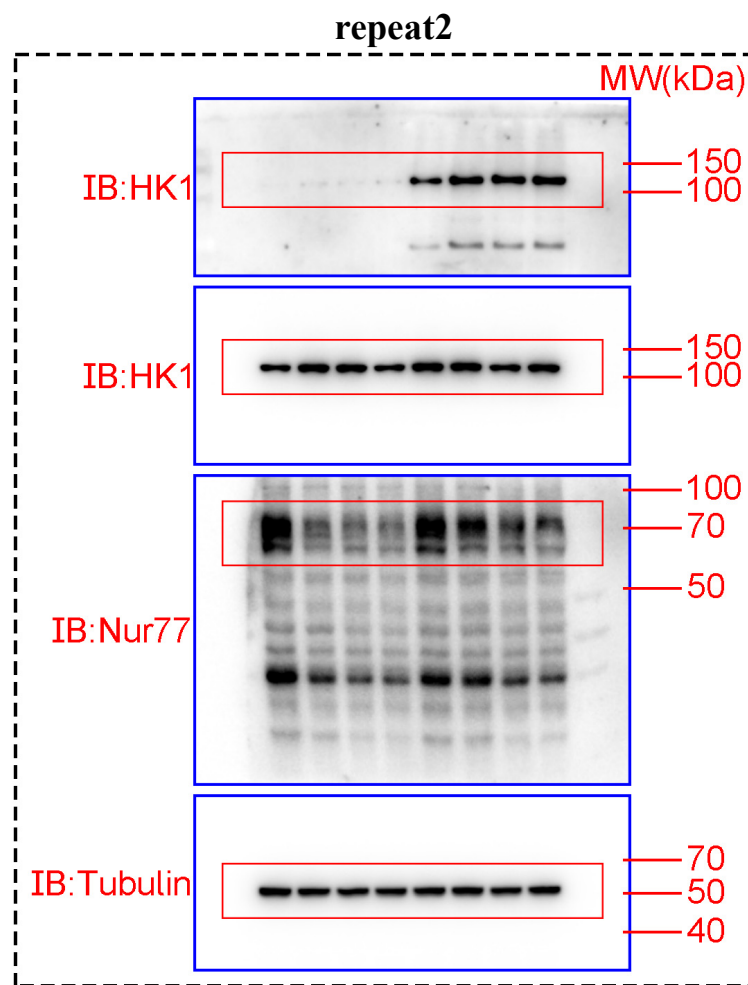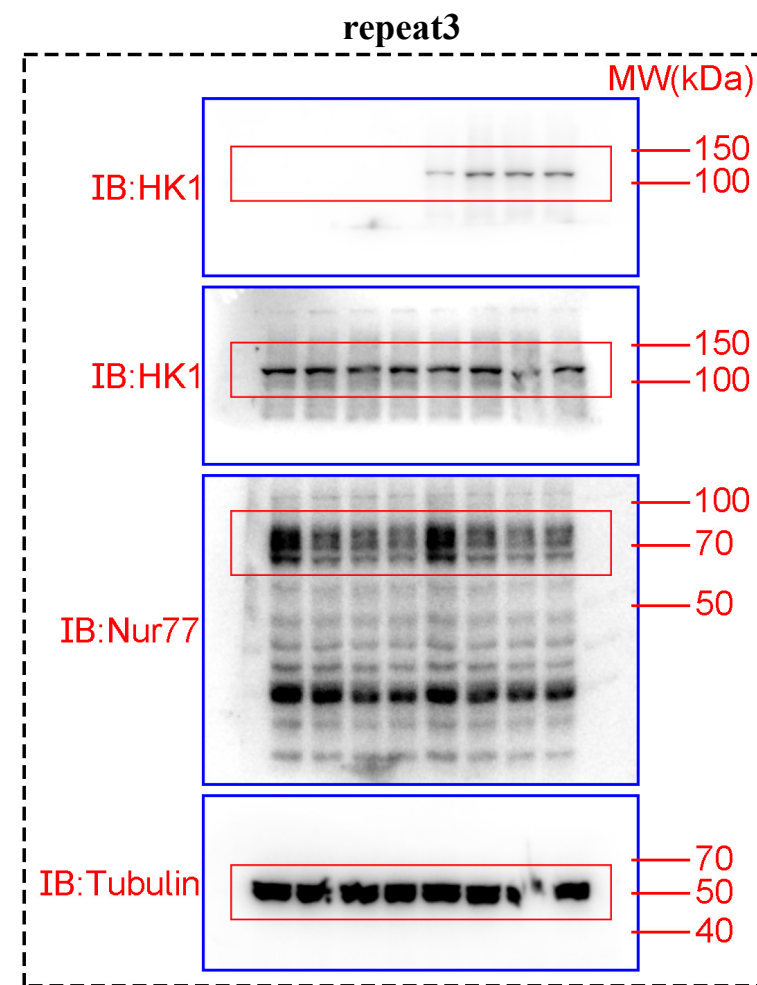

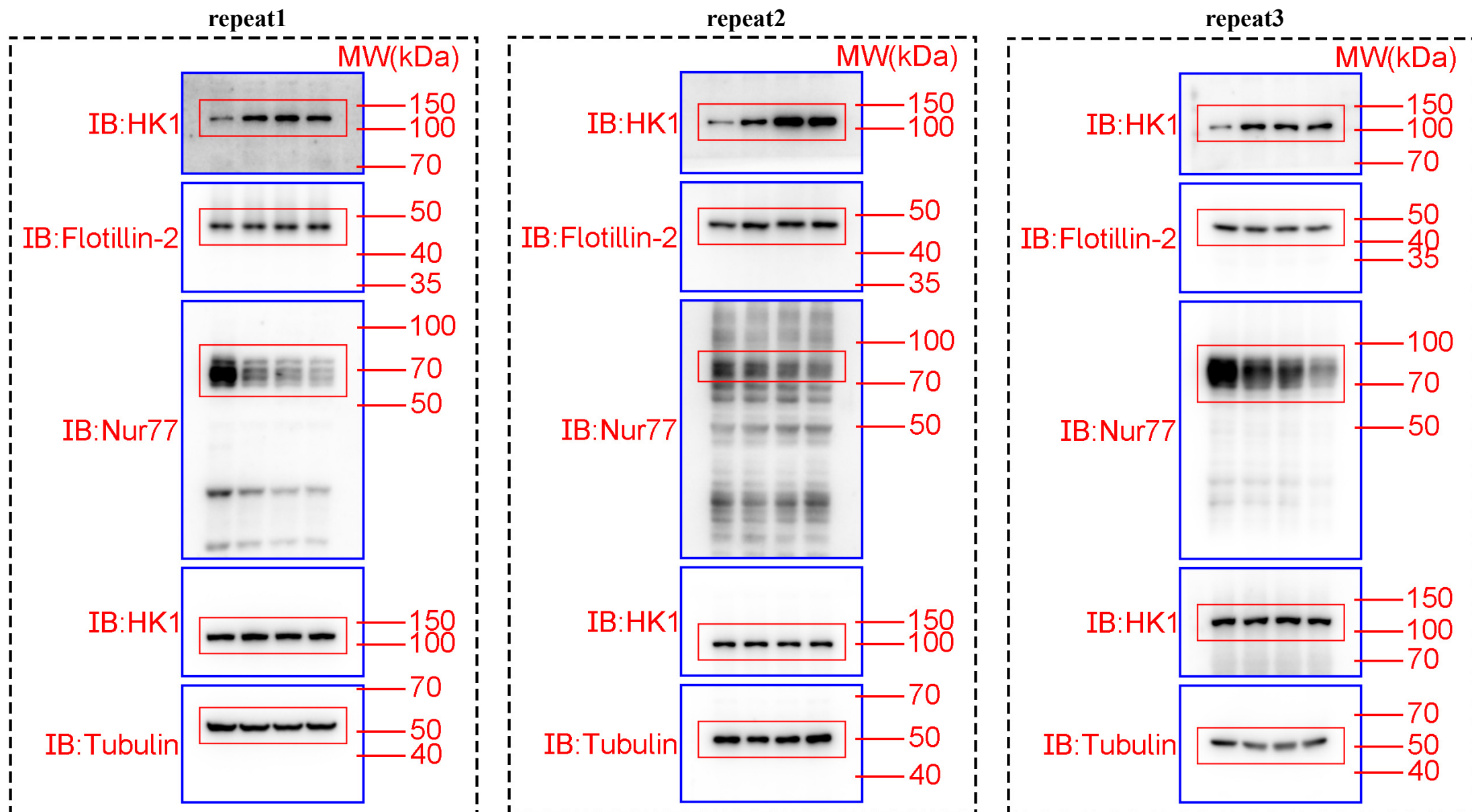

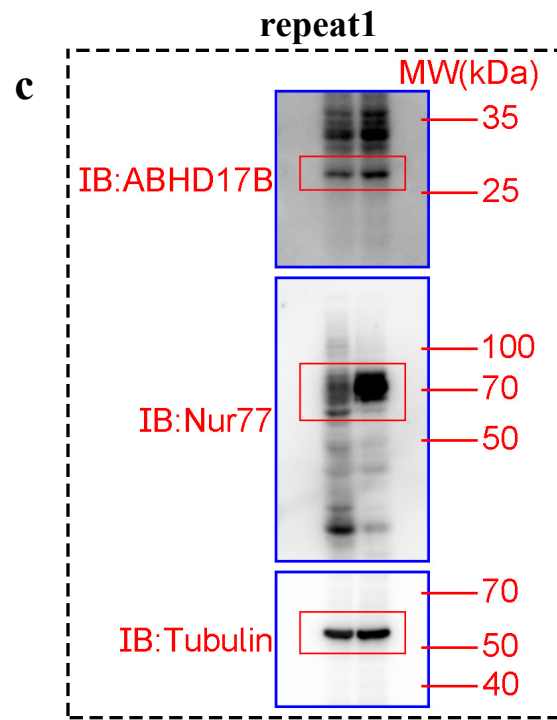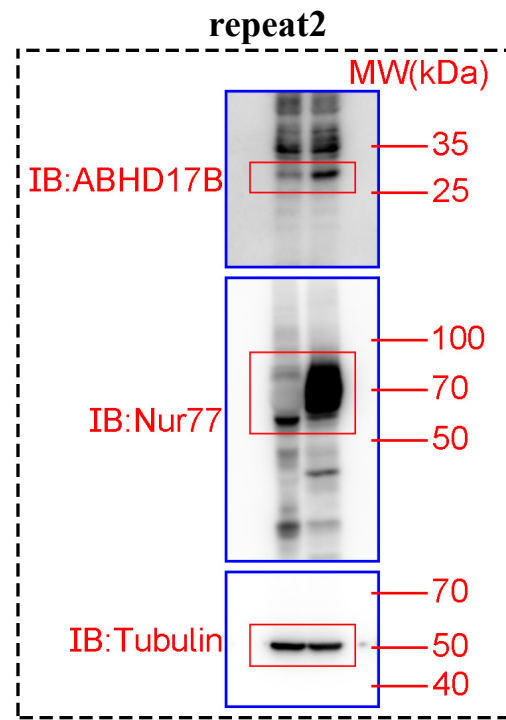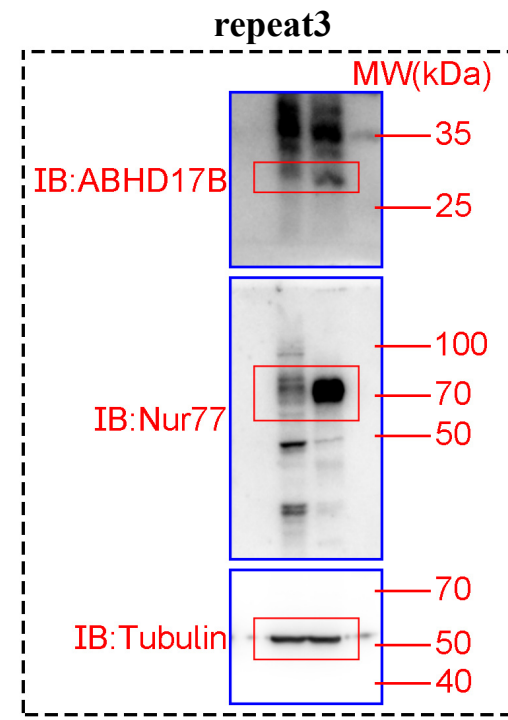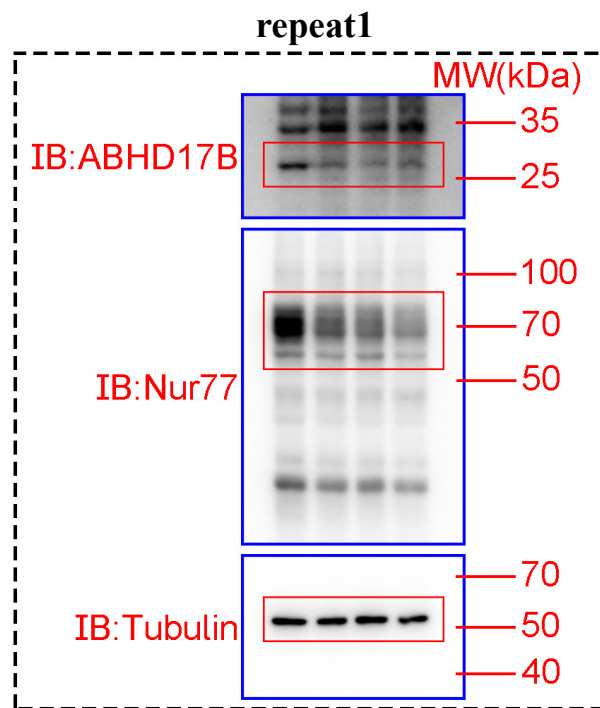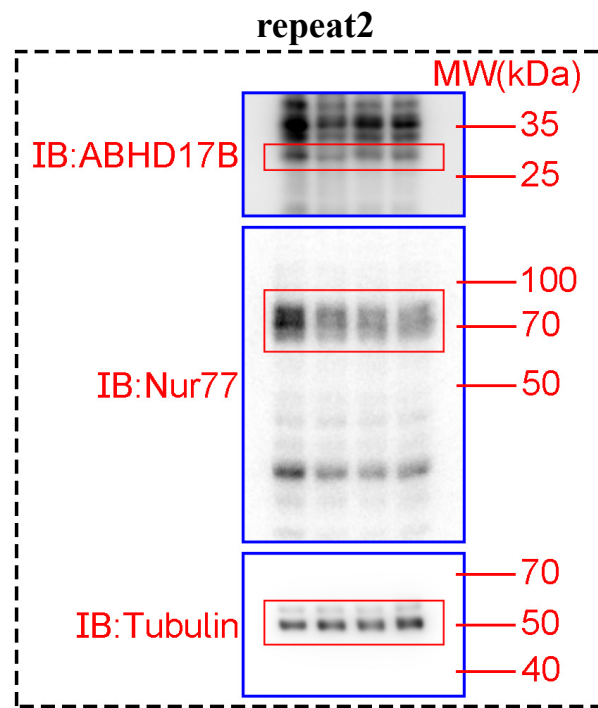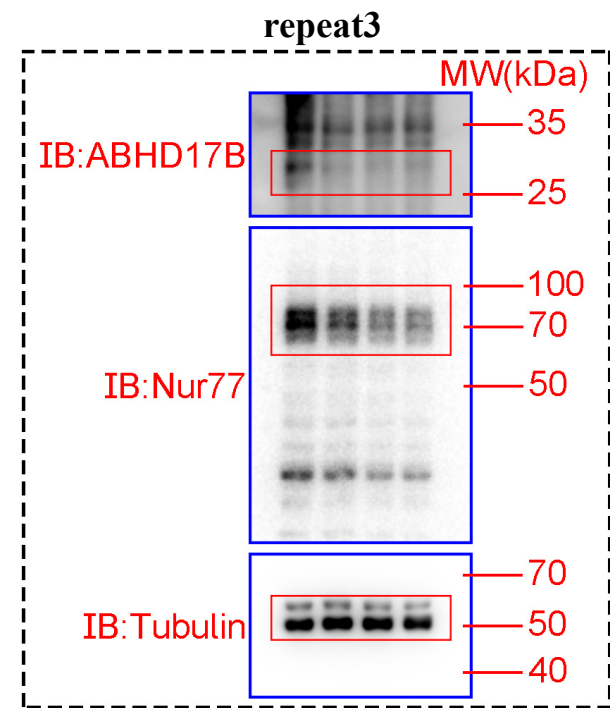

**f**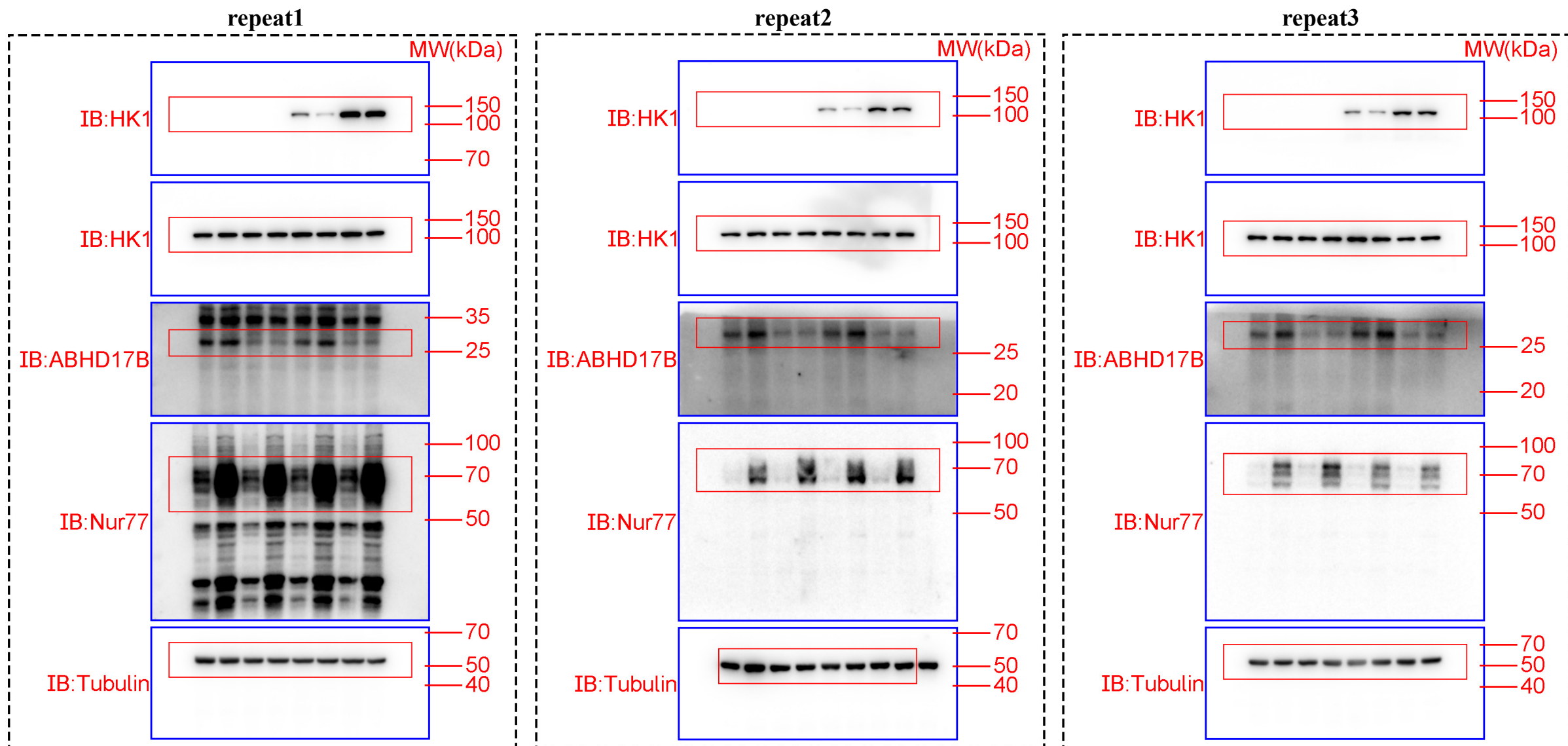

repeat1

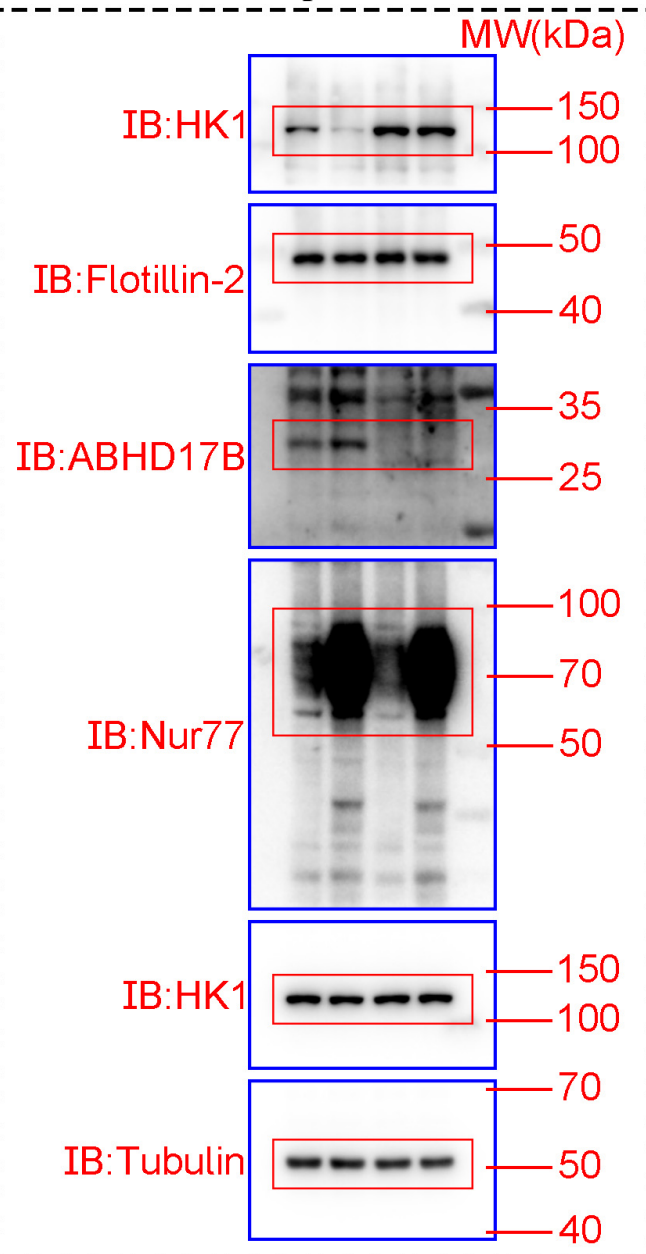

repeat2

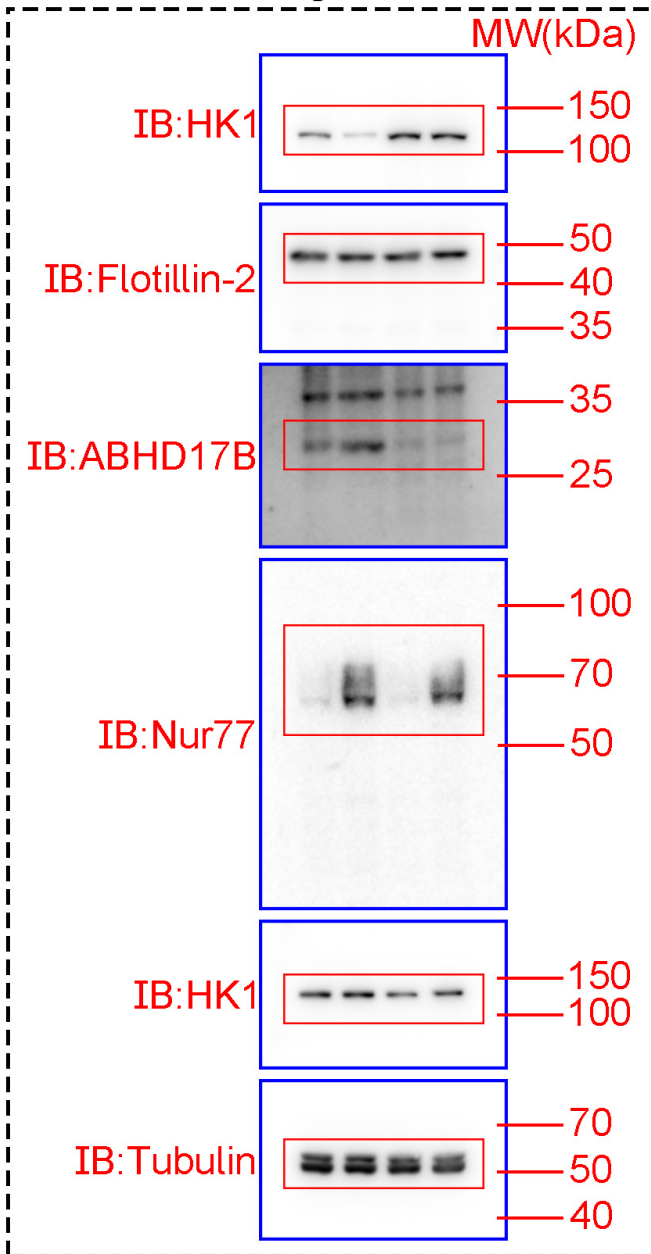

repeat3

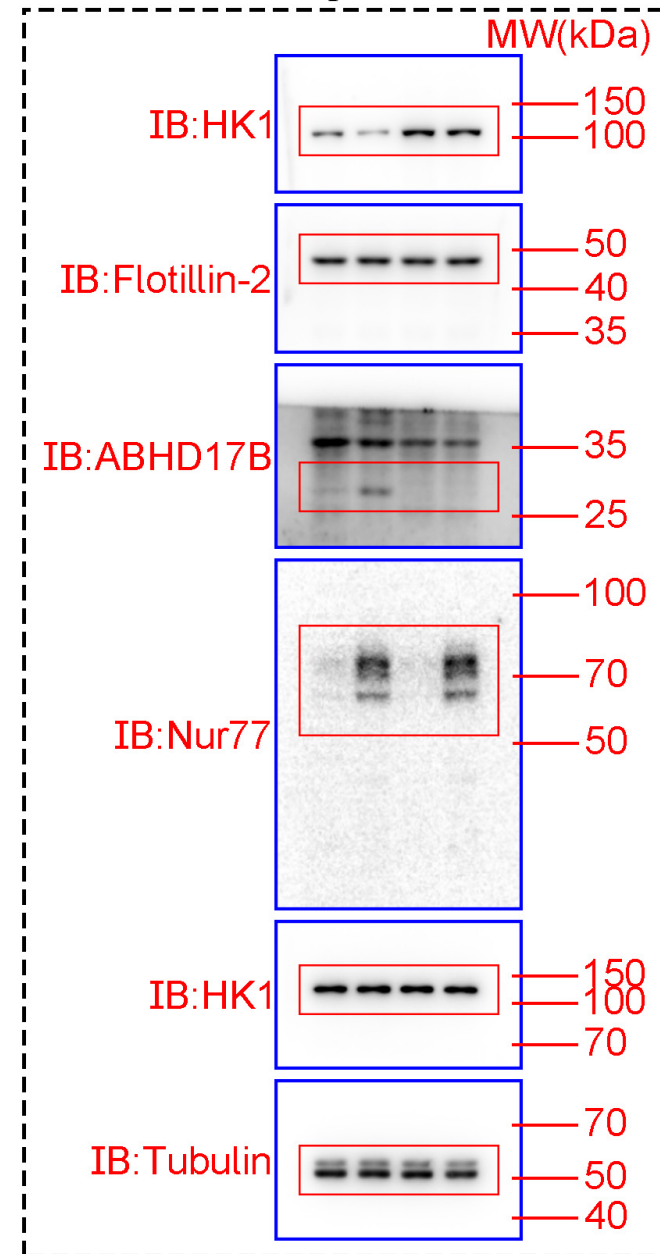

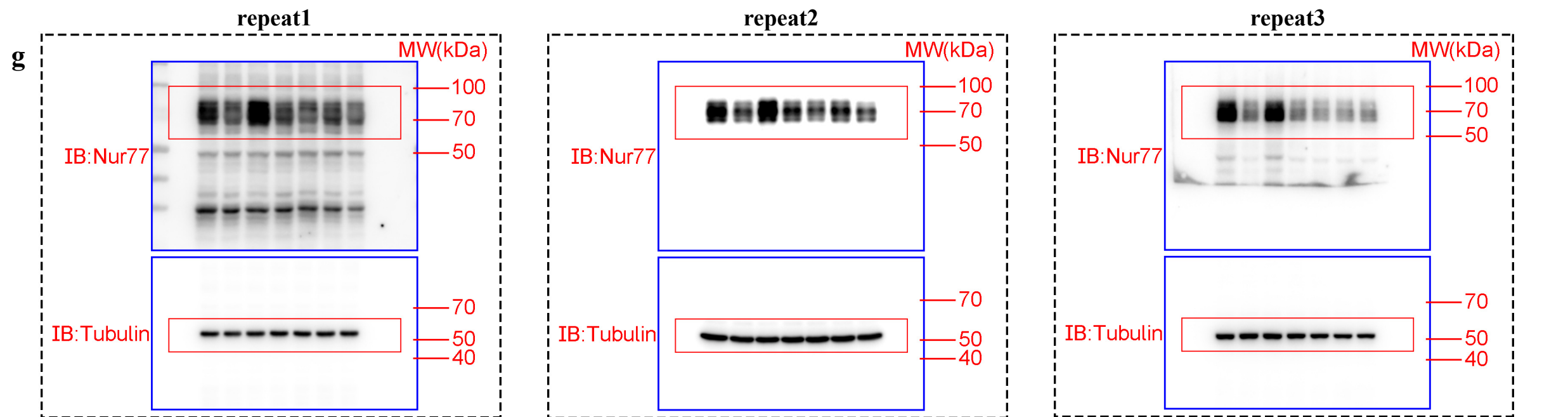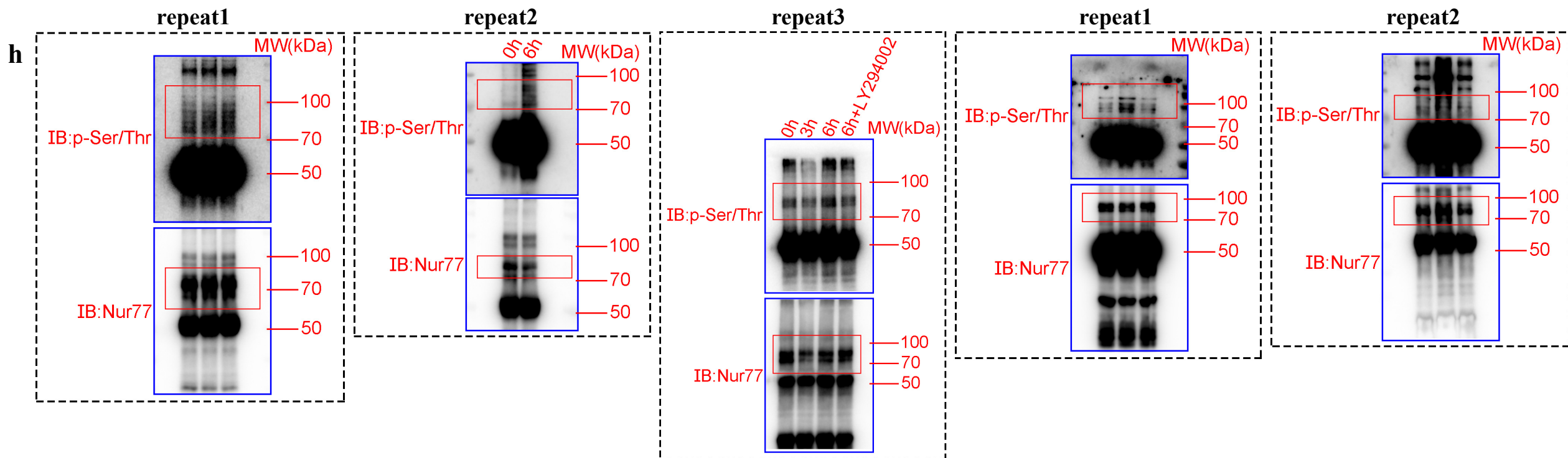

**i**

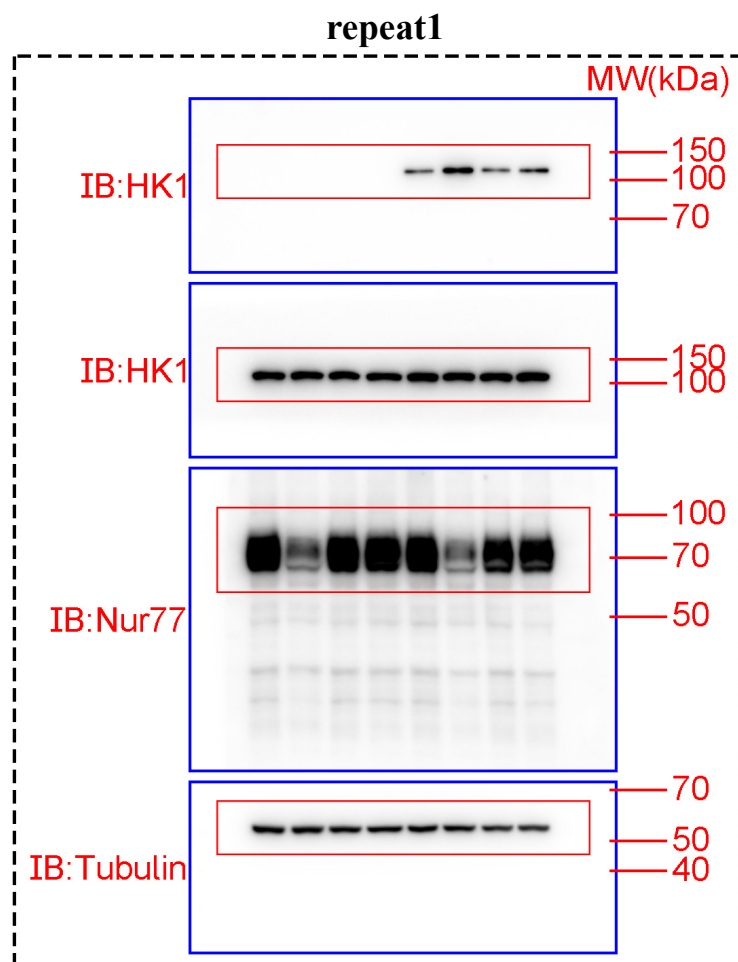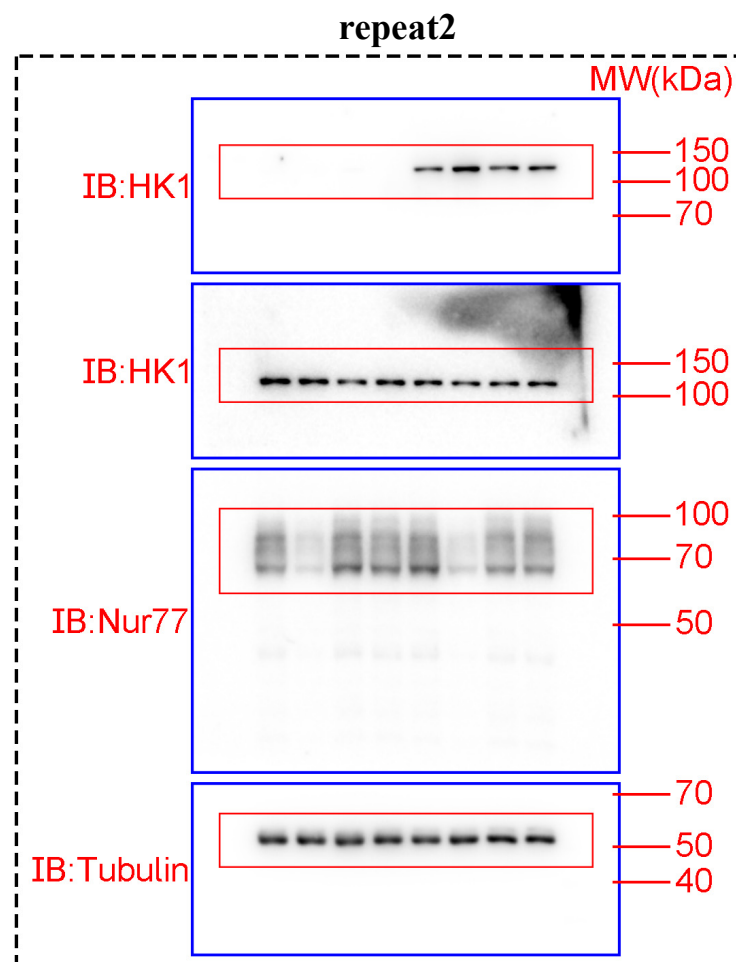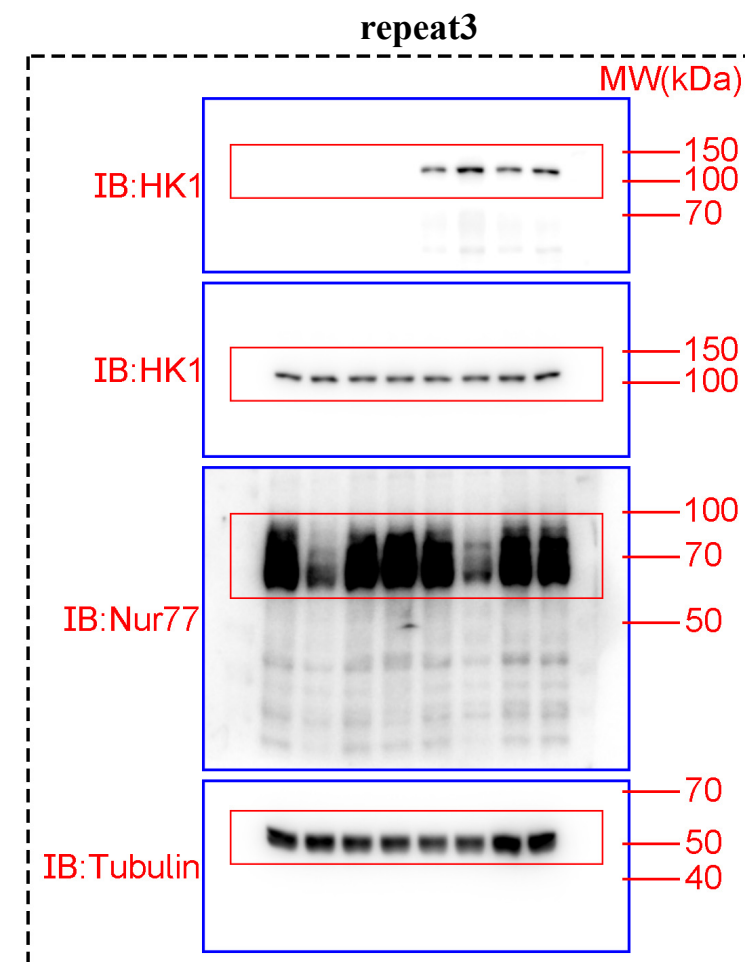

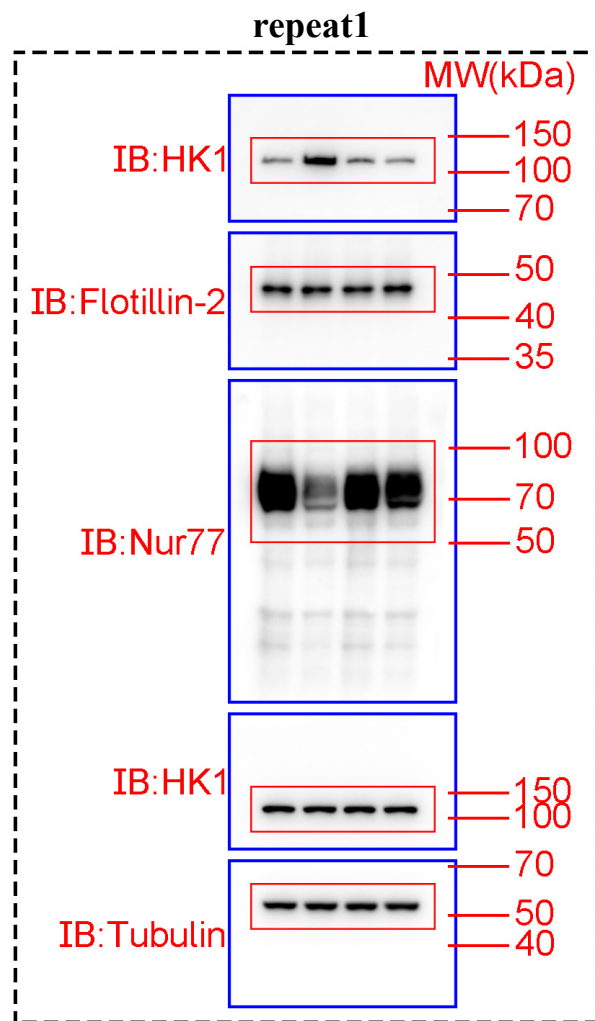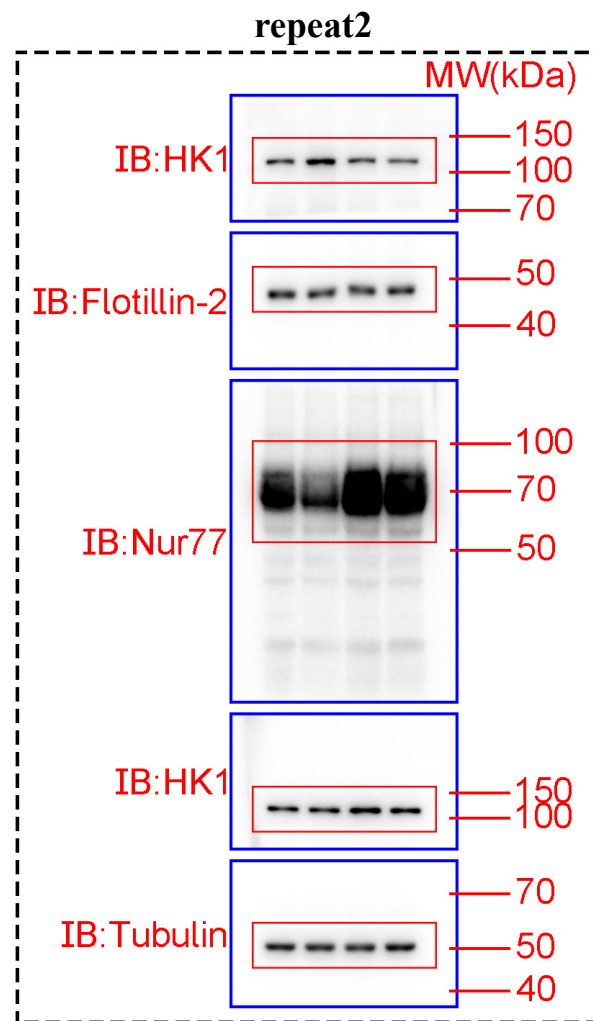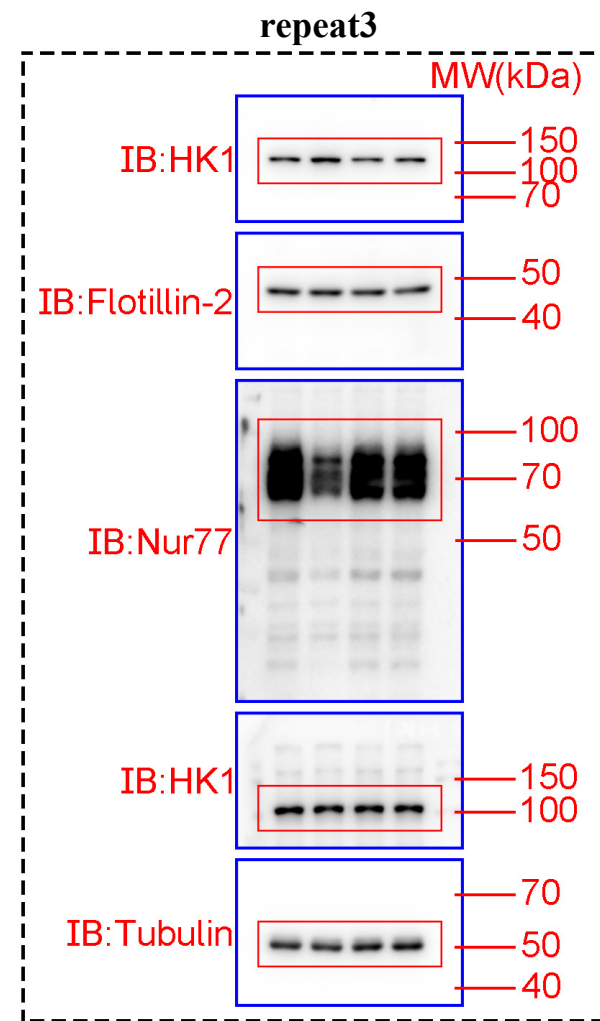

Supplement: Source Data Fig. 5 — Unprocessed western blots. [file 42255_2022_642_MOESM13_ESM.pdf]

### Figure 6

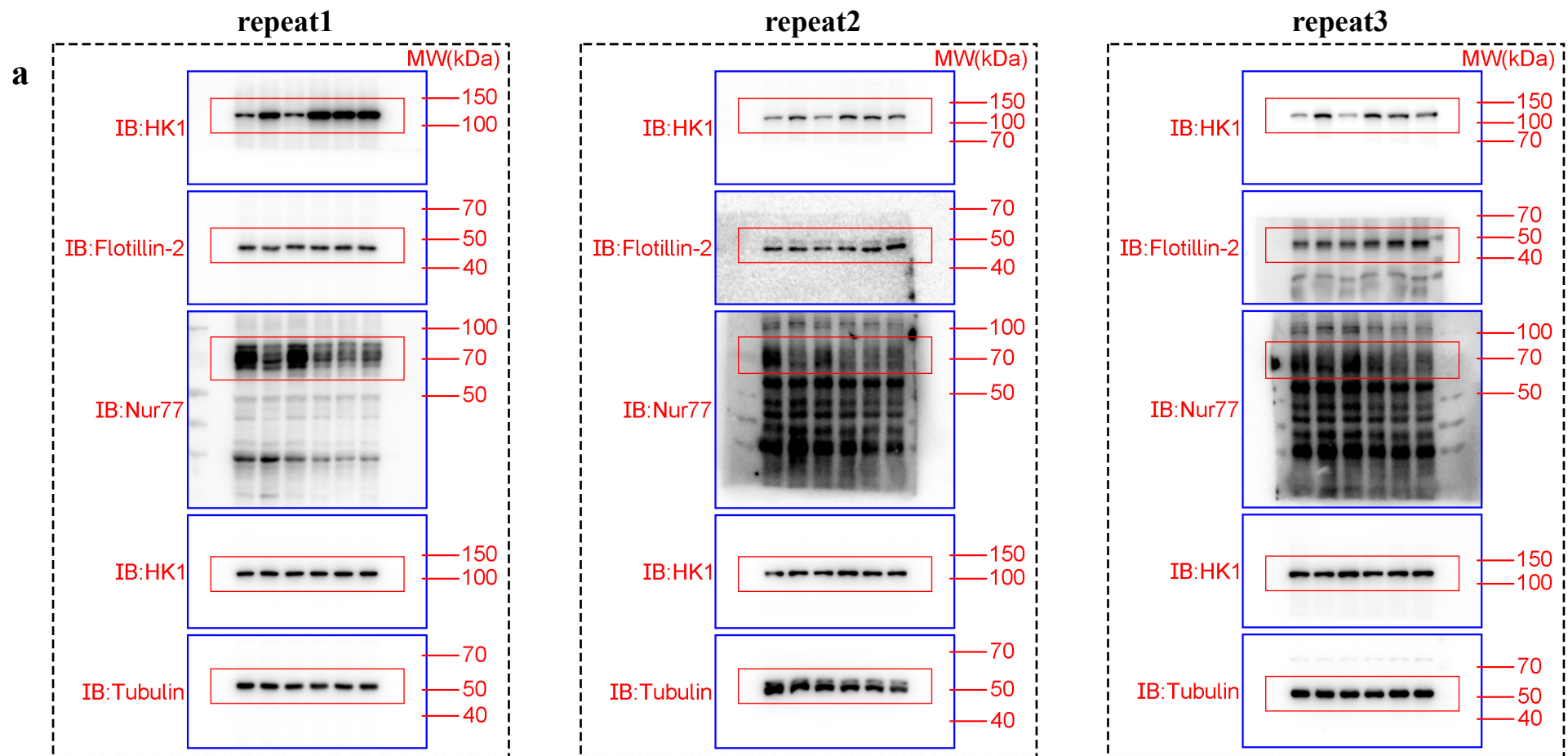

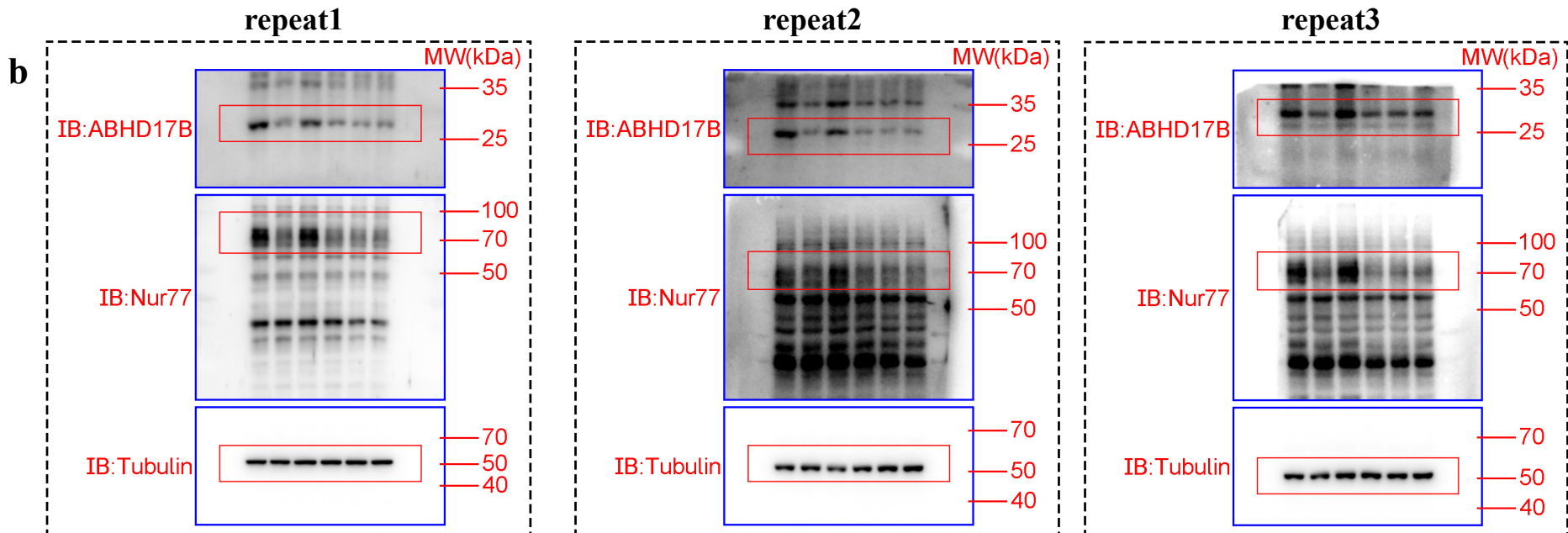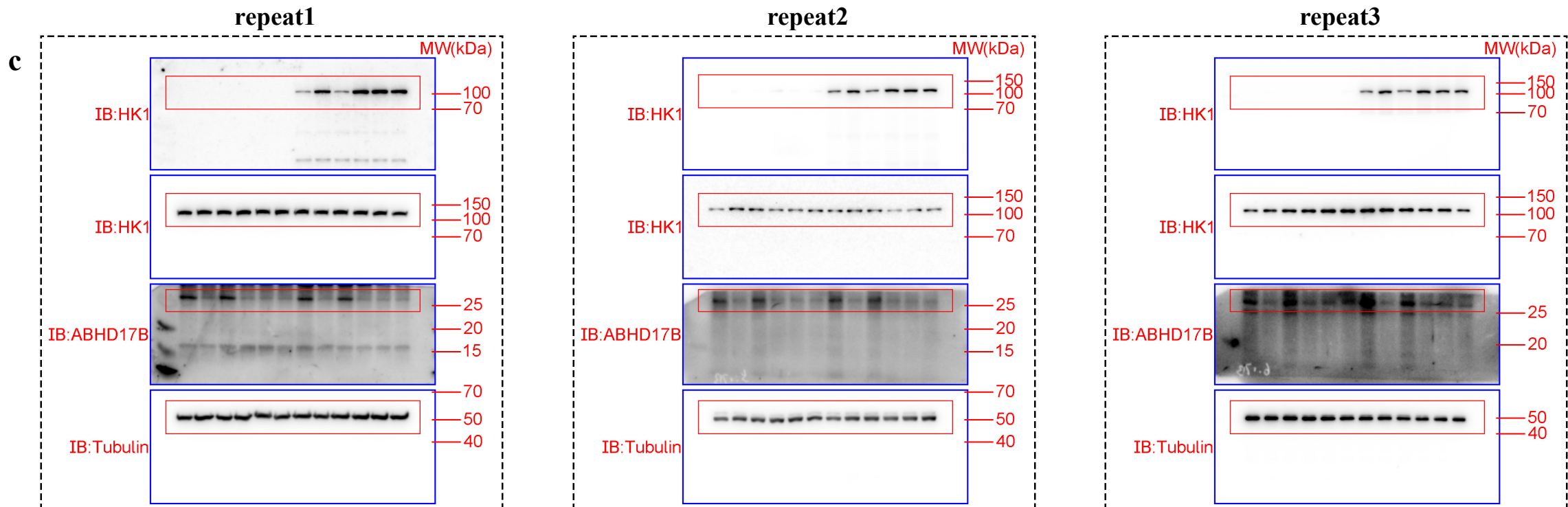

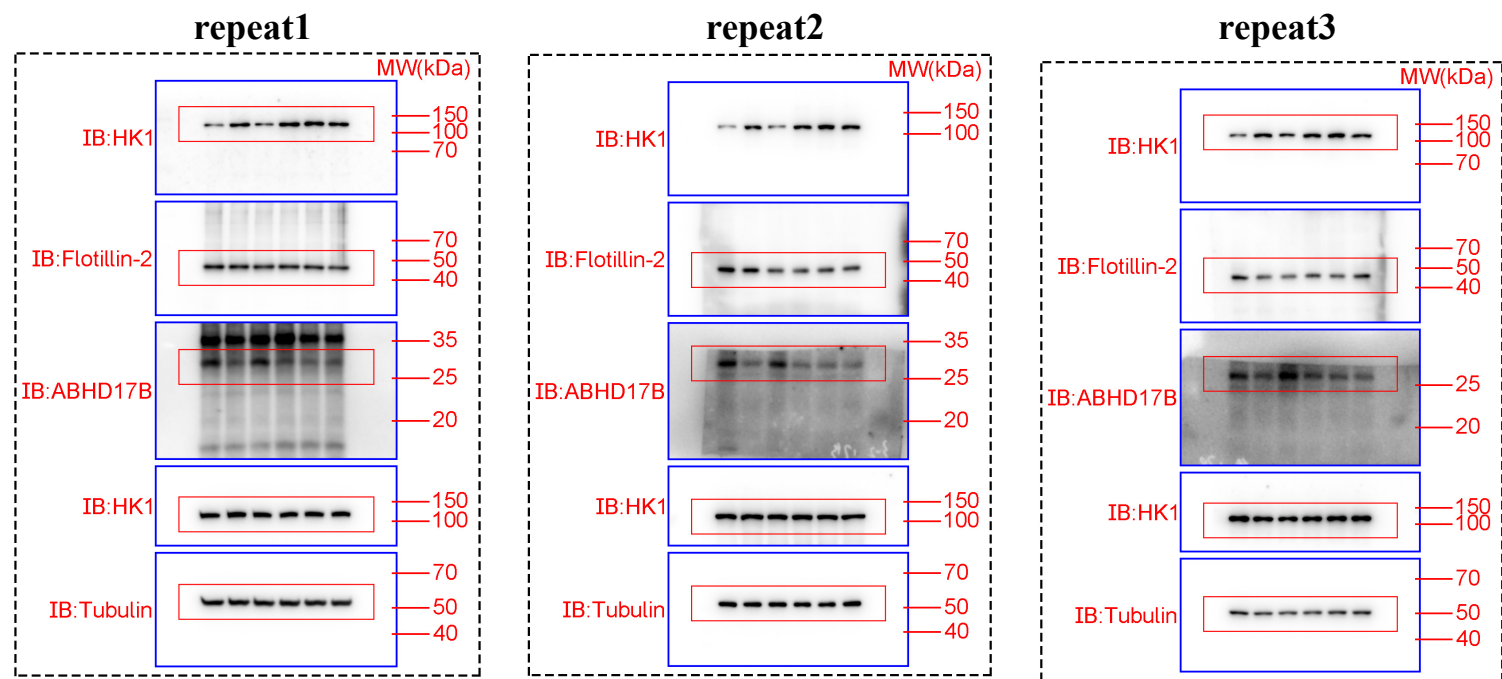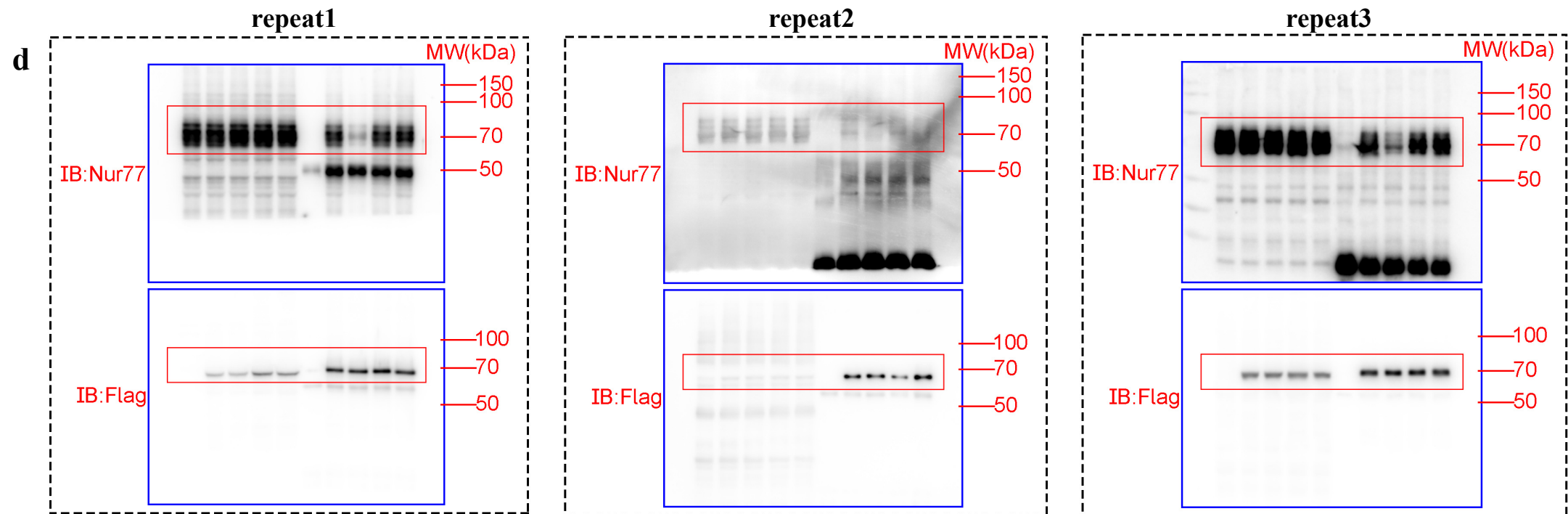

e

repeat1

repeat2

repeat3

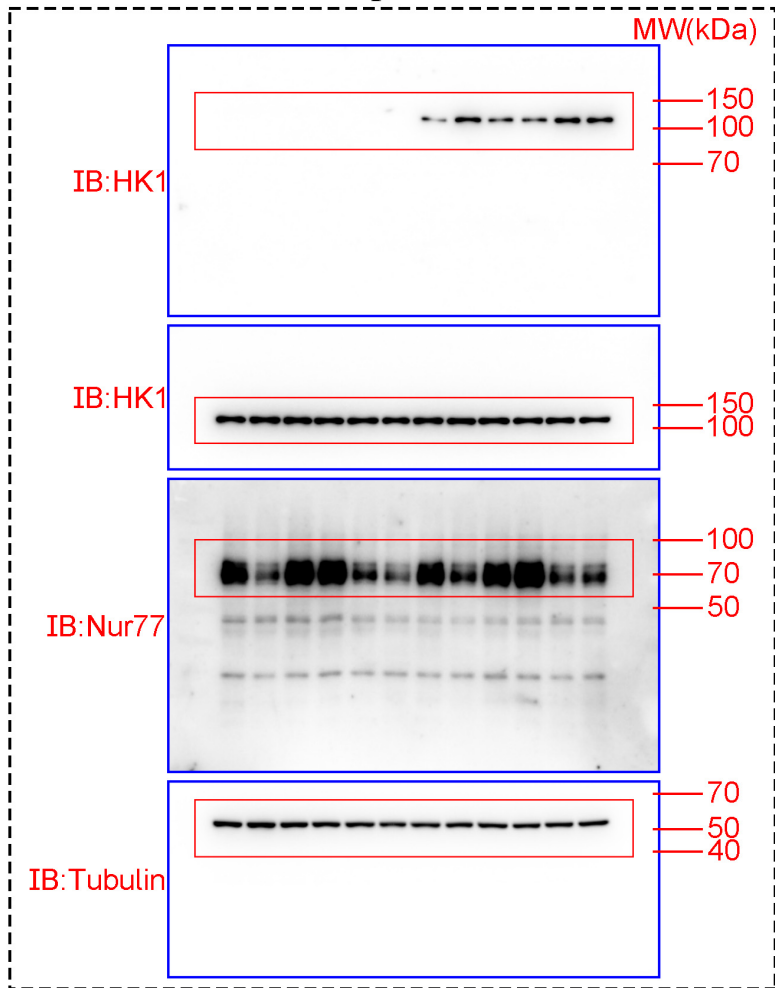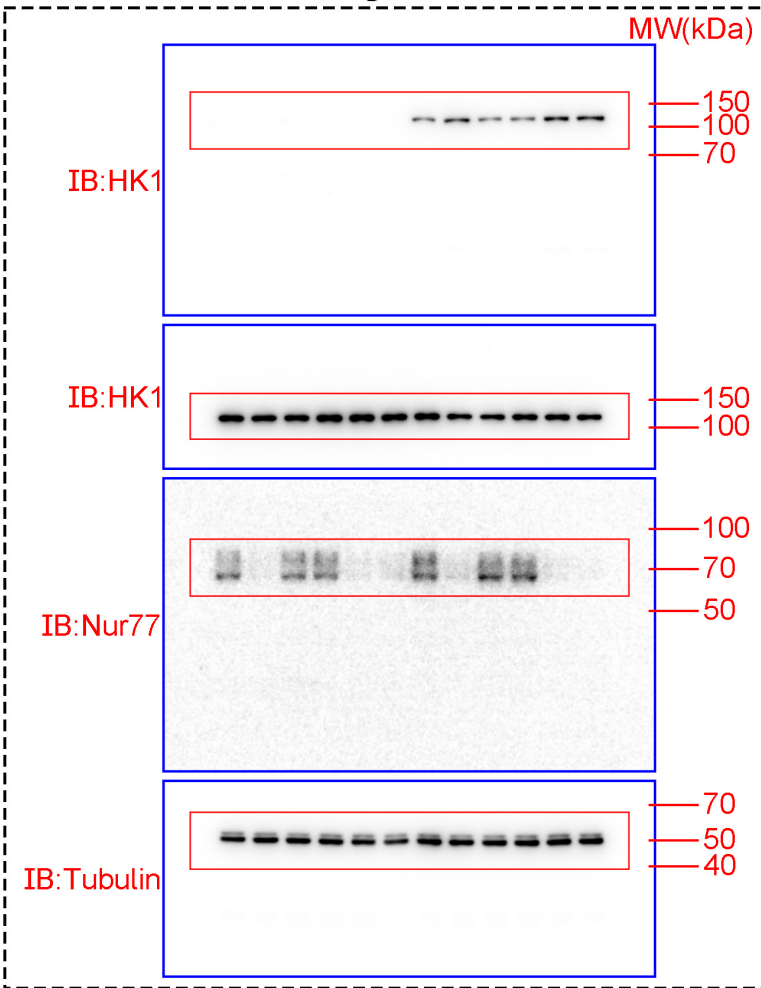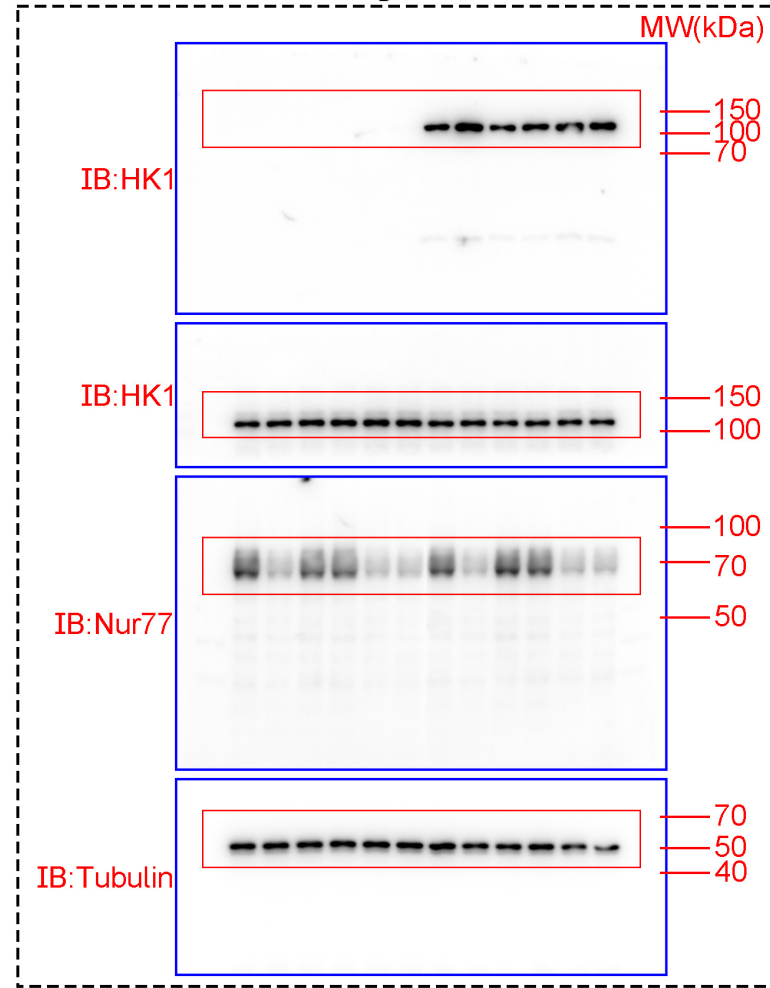

**repeat1**

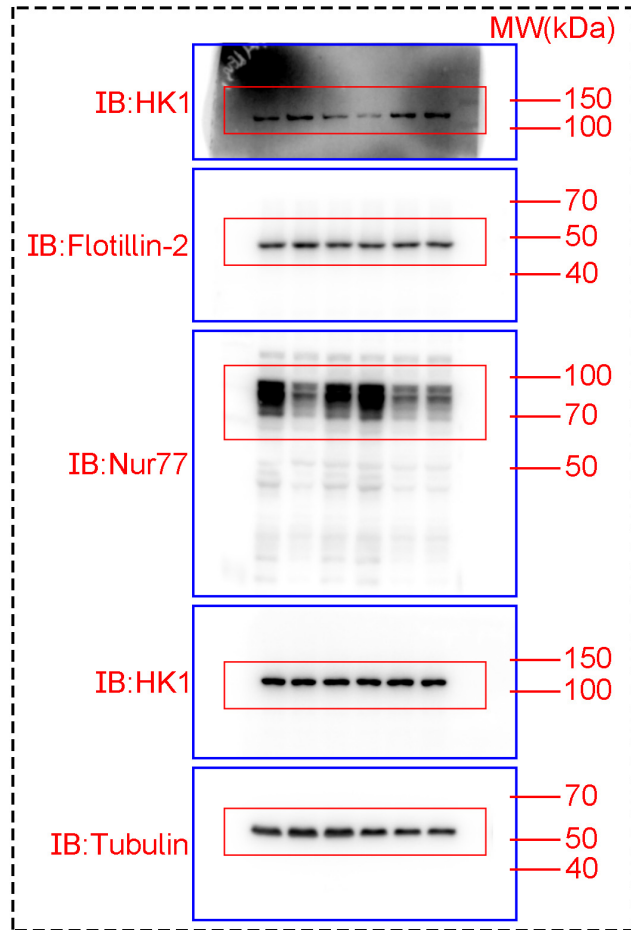

**repeat2**

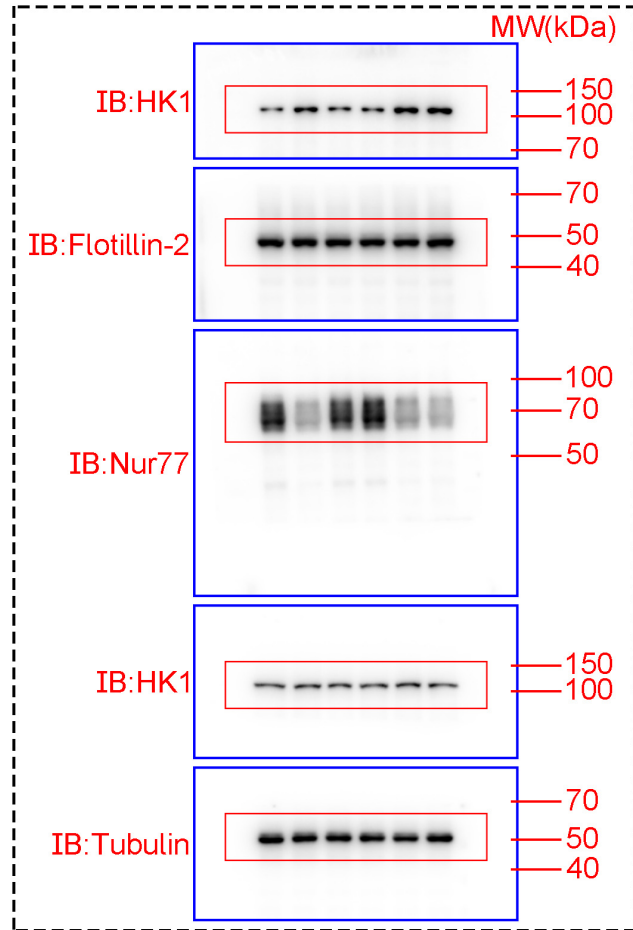

**repeat3**

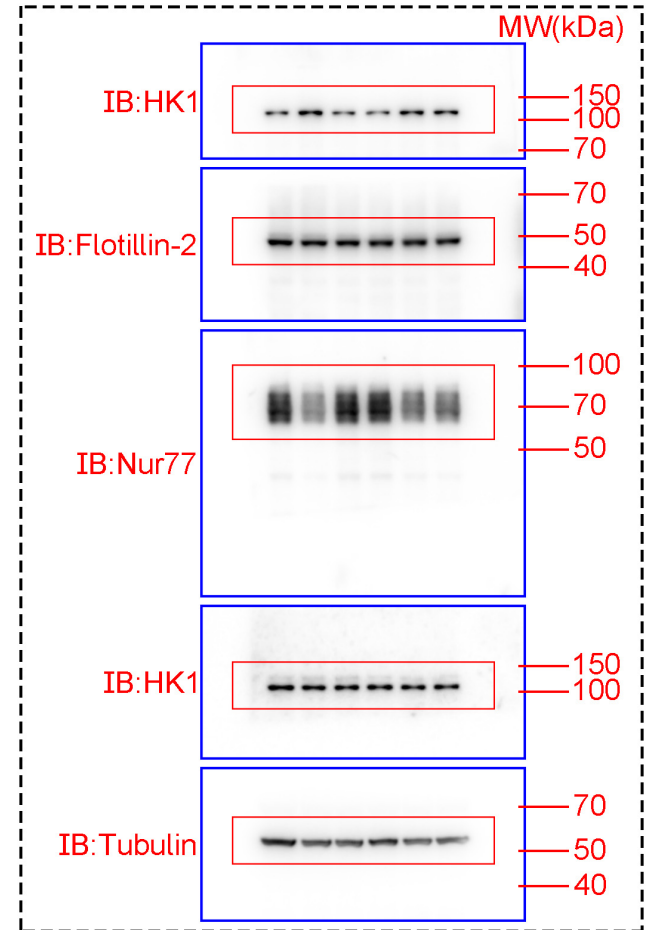

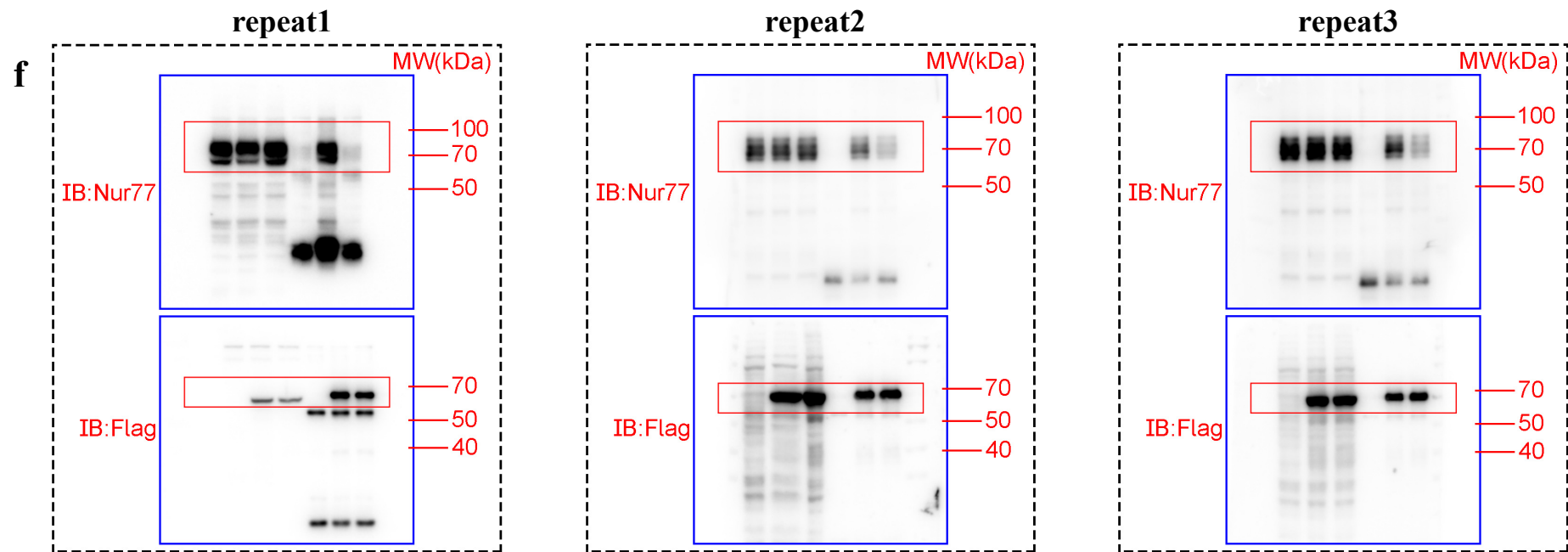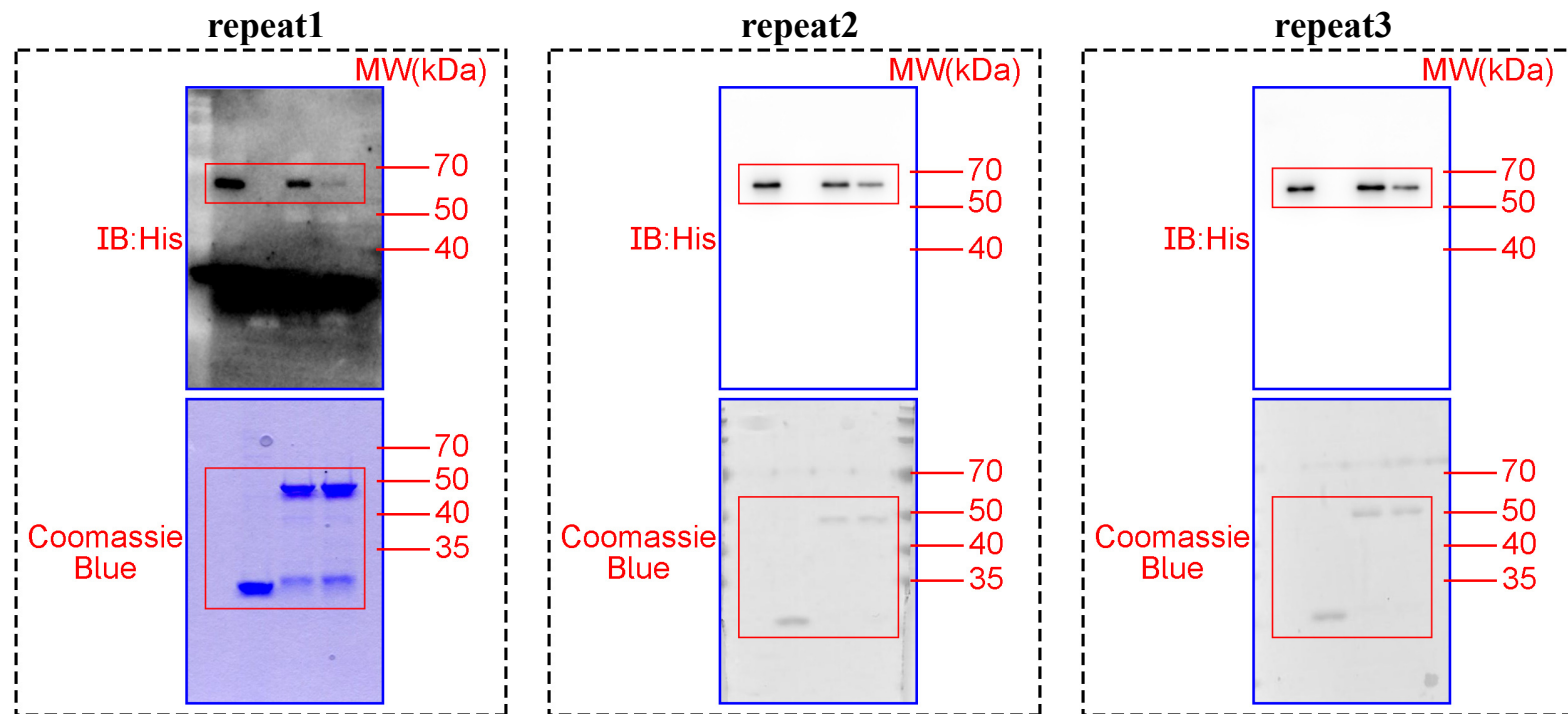

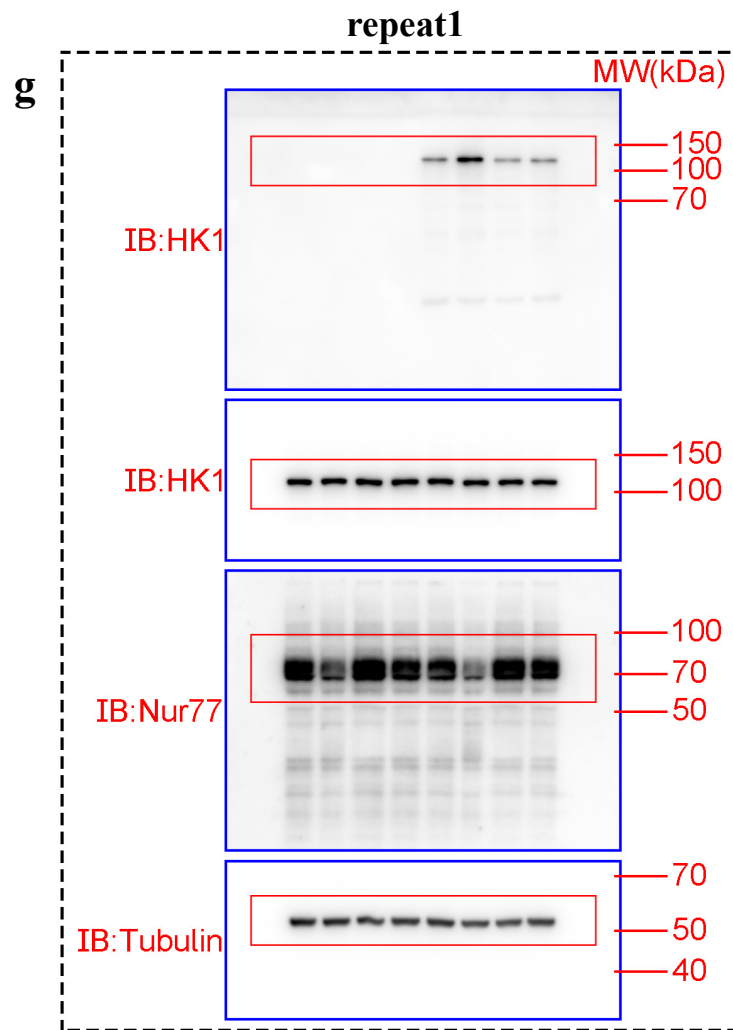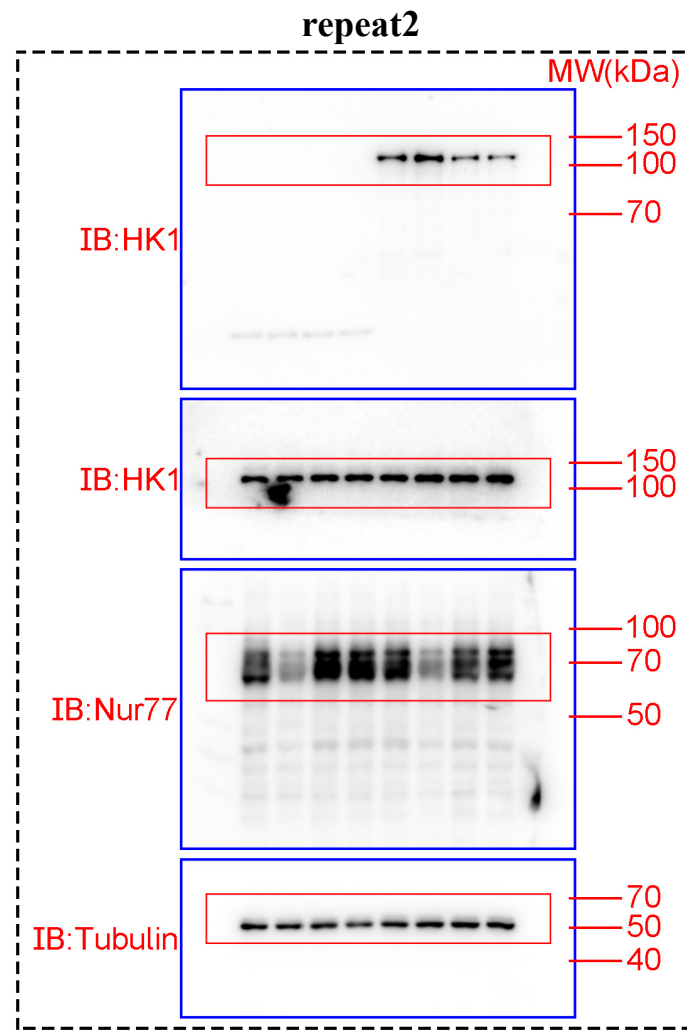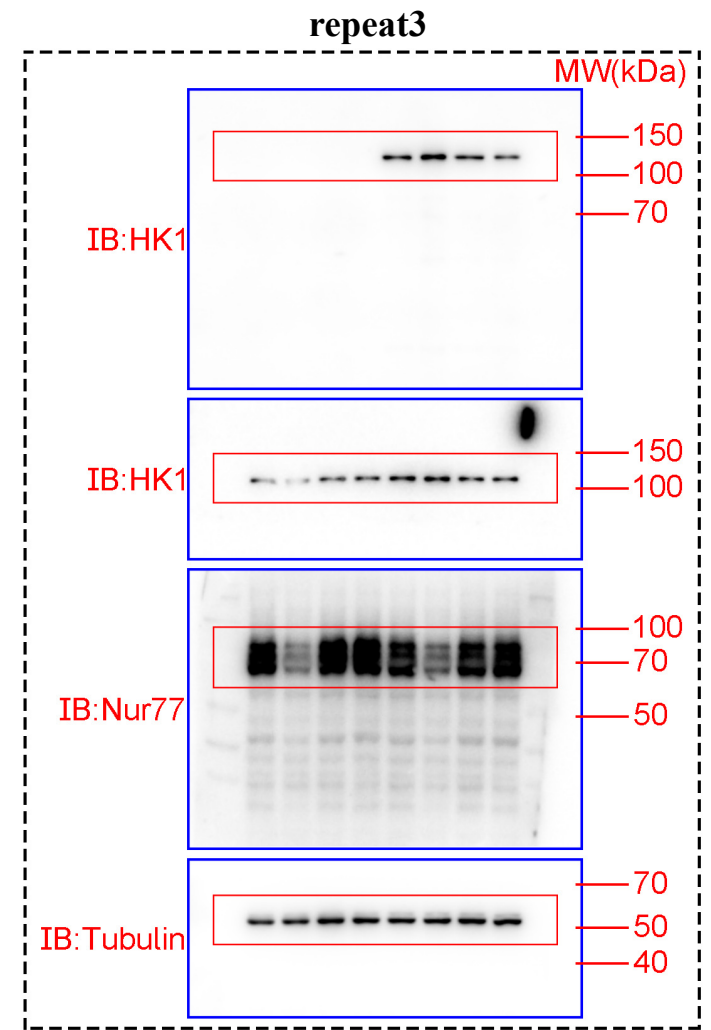

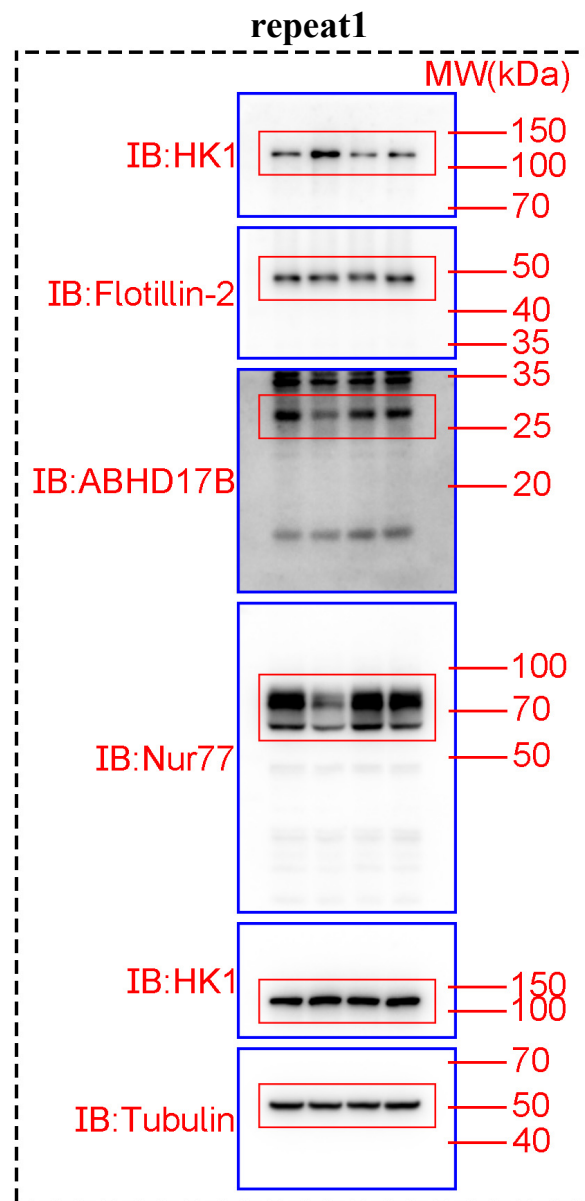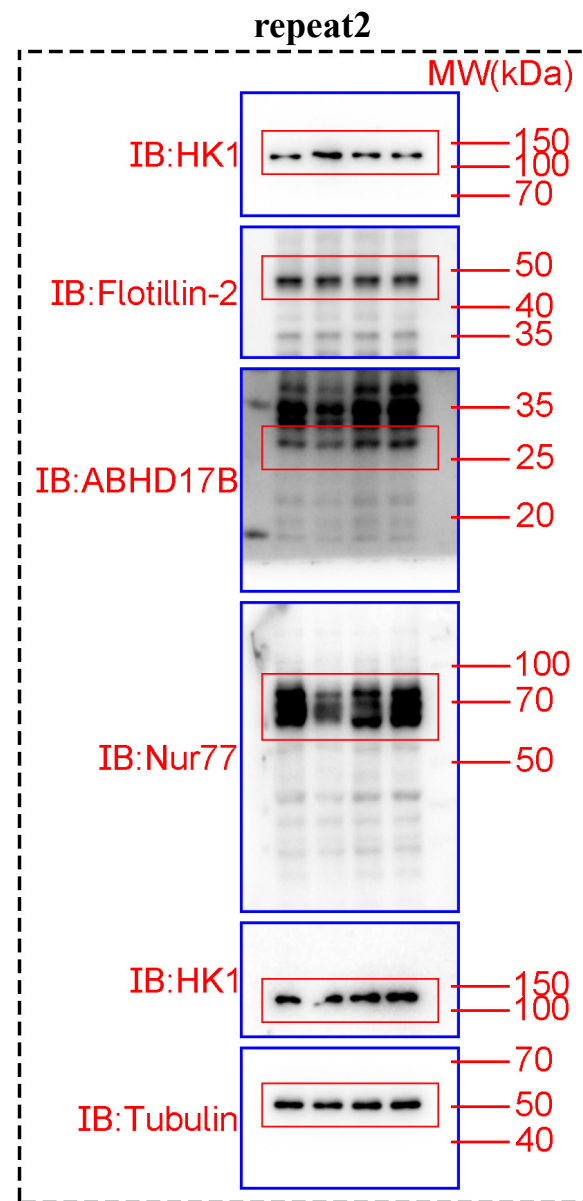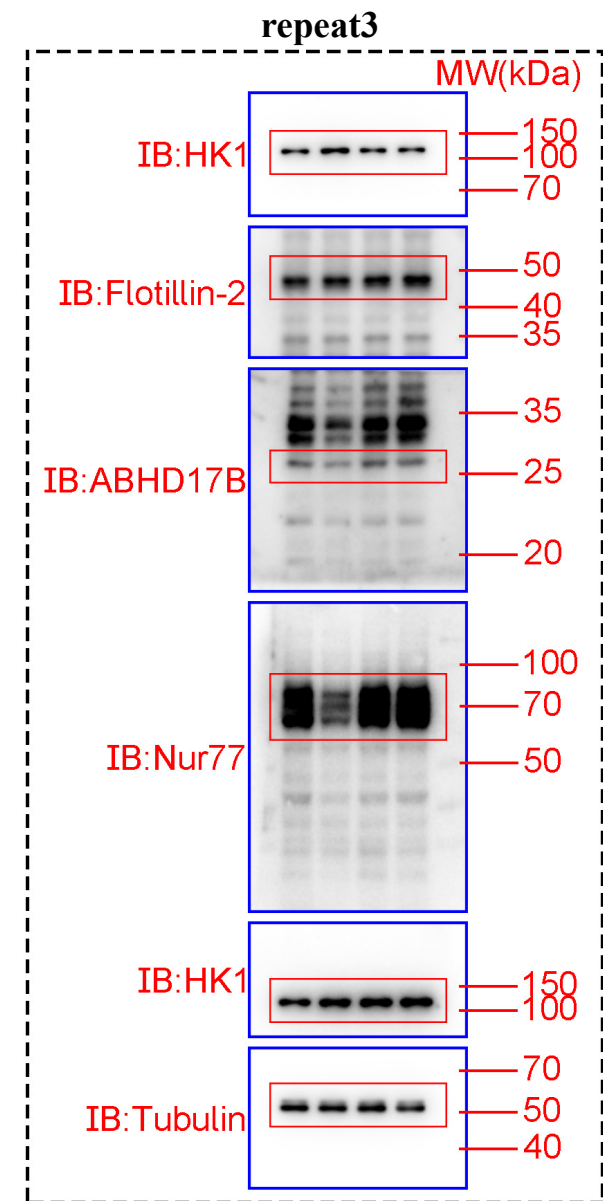

Supplement: Source Data Fig. 6 — Unprocessed western blots. [file 42255_2022_642_MOESM15_ESM.pdf]

# Uncropped western blot images

## Extended Data Figure 3

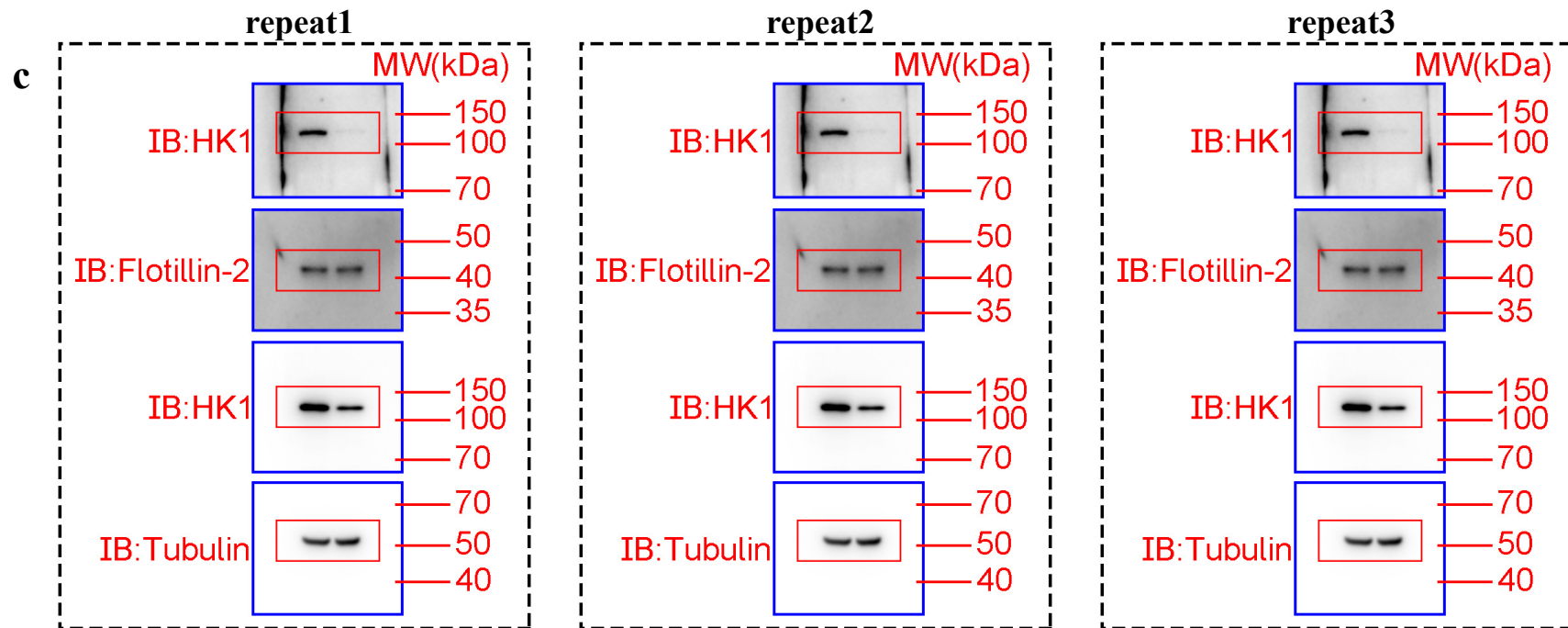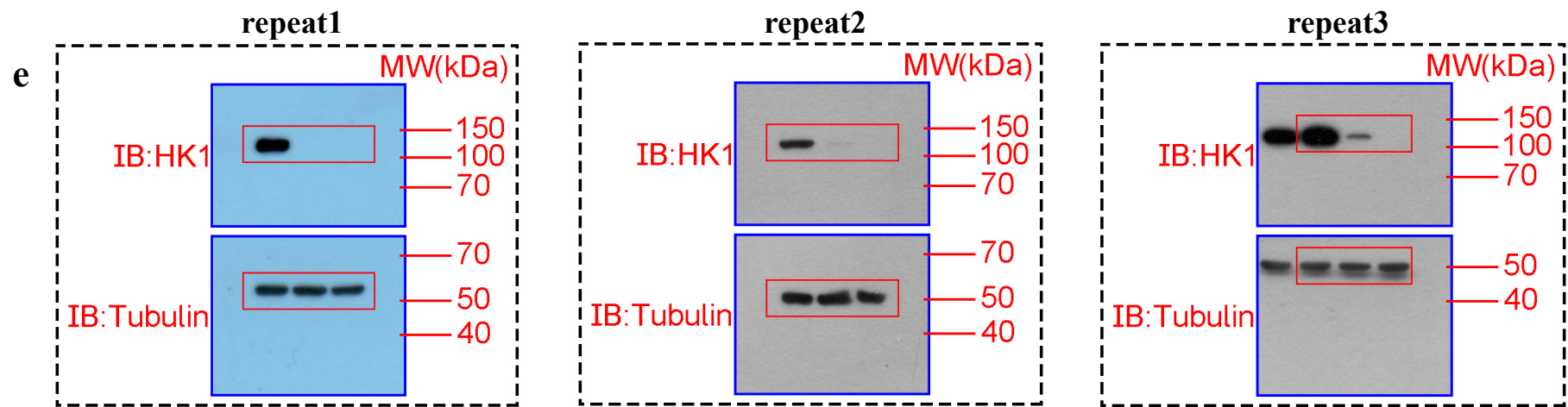



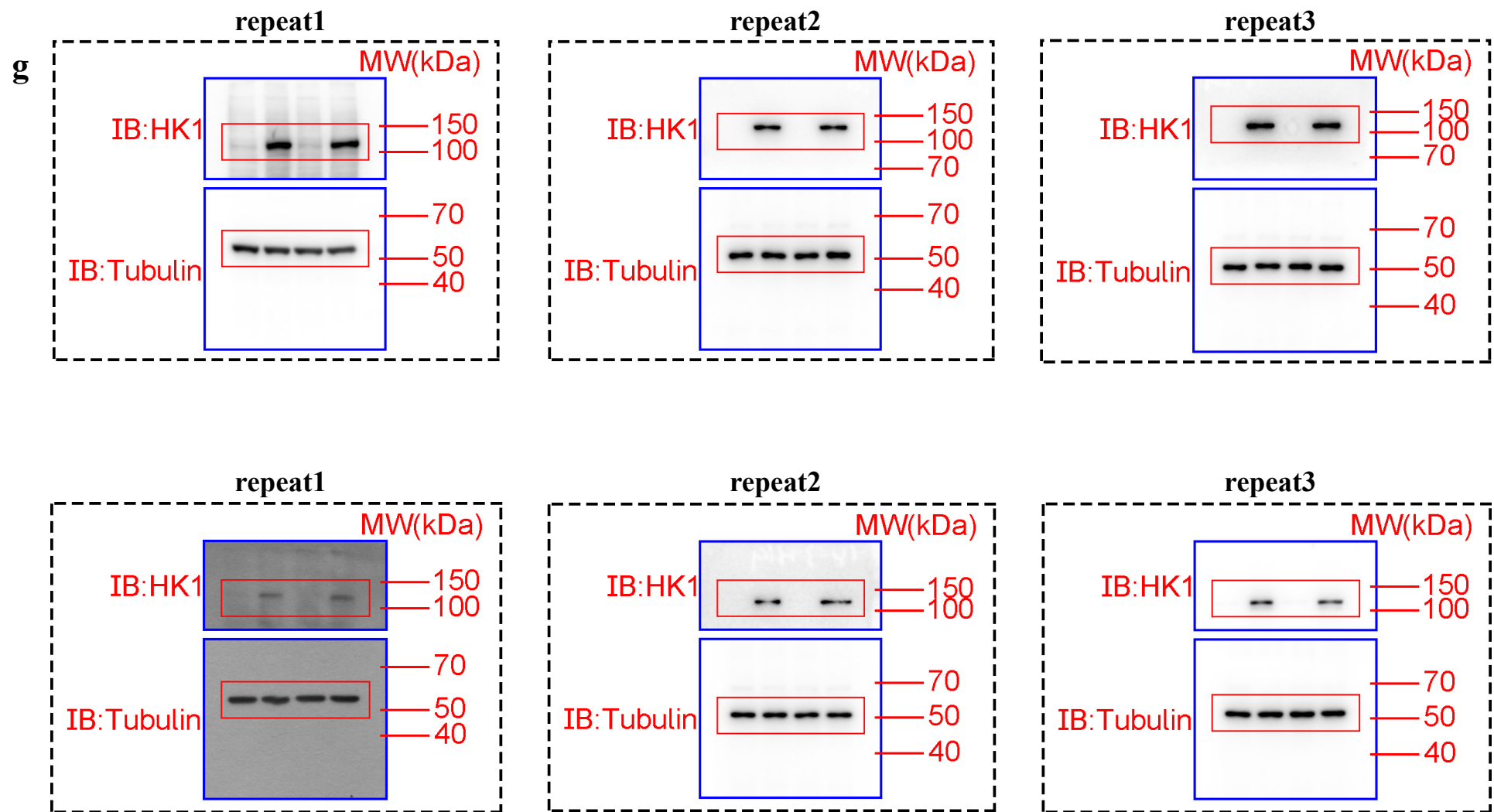

Supplement: Source Data Extended Data Fig. 3 — Unprocessed western blots. [file 42255_2022_642_MOESM21_ESM.pdf]

# Uncropped western blot images

## Extended Data Figure 4

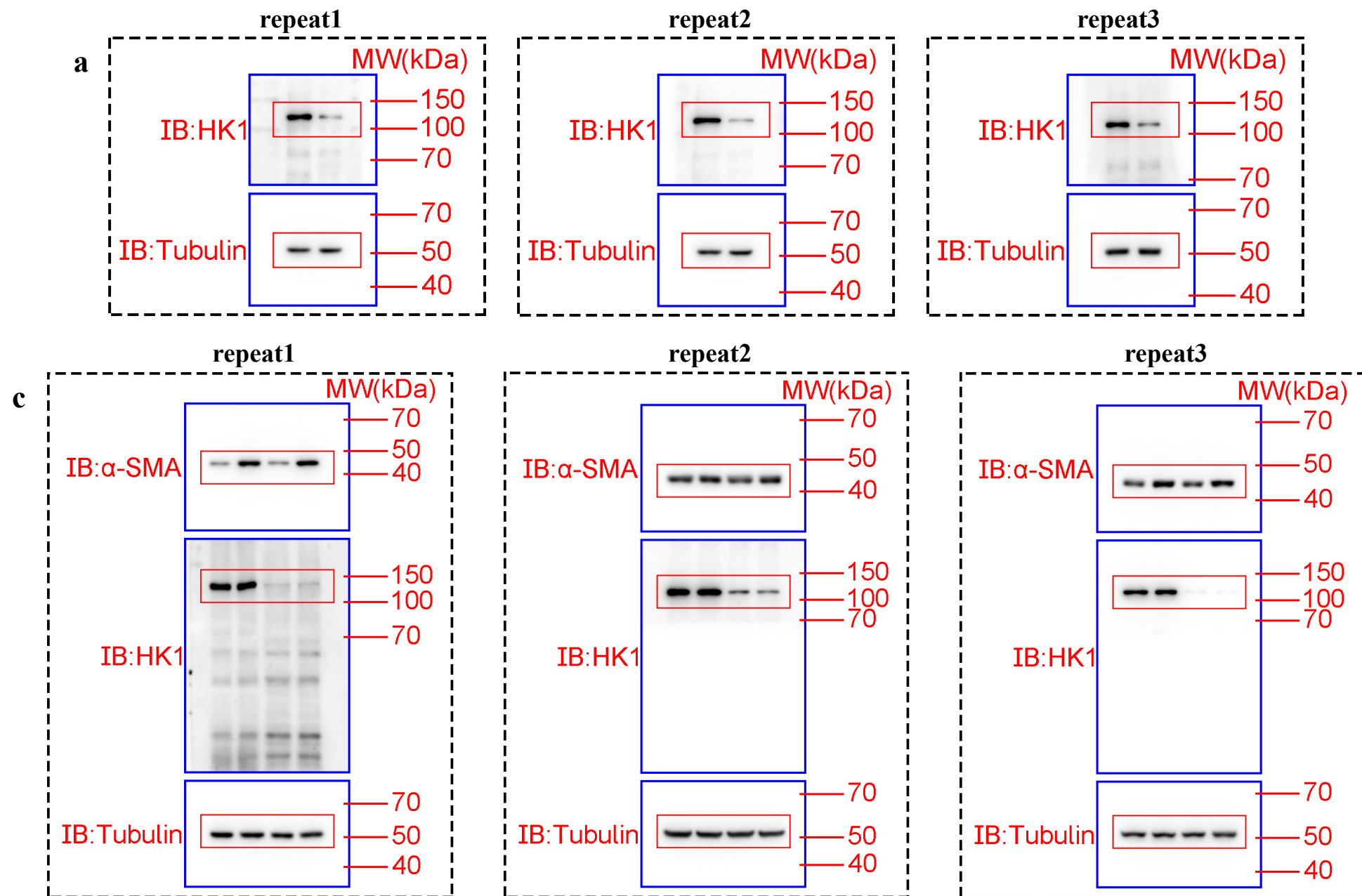

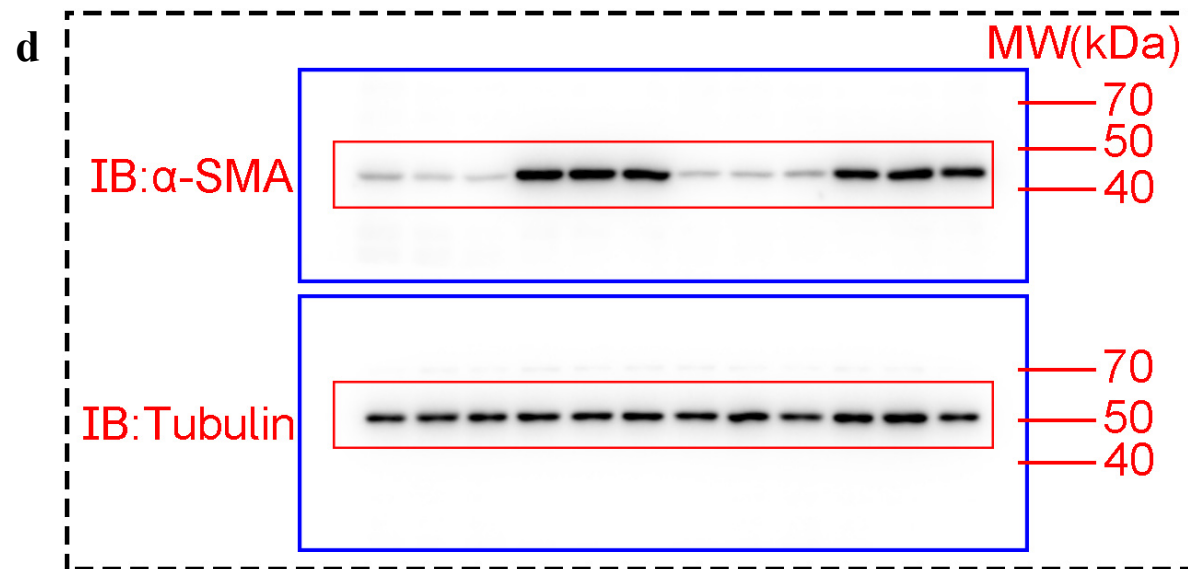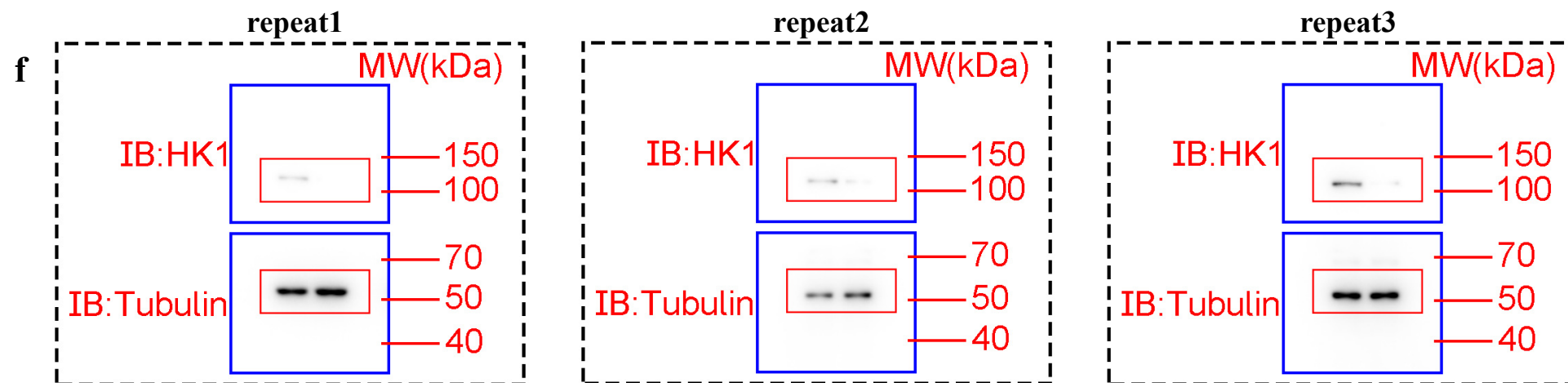

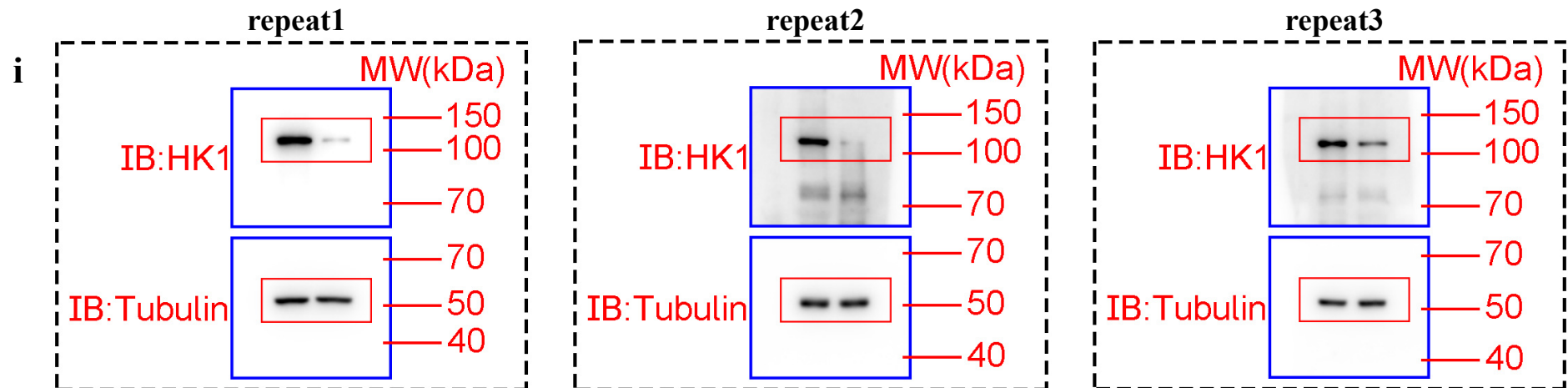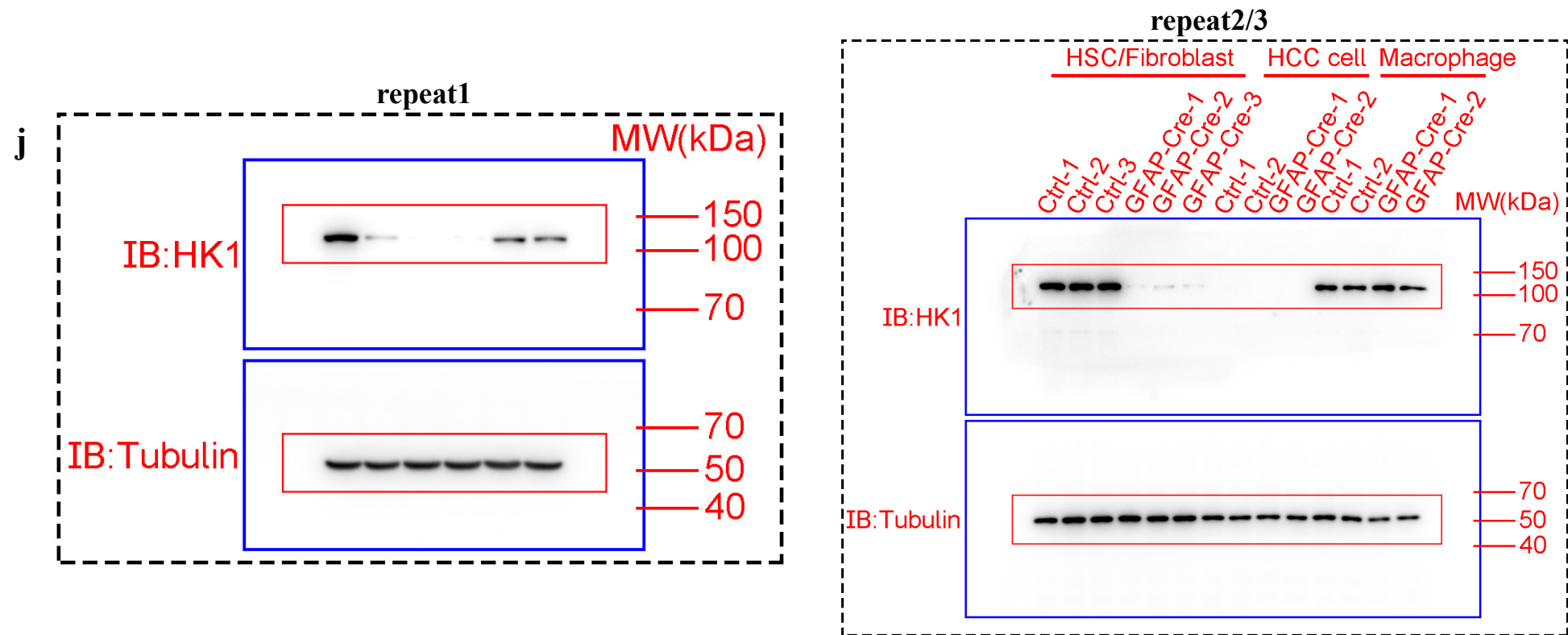

Supplement: Source Data Extended Data Fig. 4 — Unprocessed western blots. [file 42255_2022_642_MOESM23_ESM.pdf]
