# Supplementary material for: HK1 from hepatic stellate cell–derived extracellular vesicles promotes progression of hepatocellular carcinoma
Source: Nat Metab. 2022 Oct 3;4(10):1306–21. doi: 10.1038/s42255-022-00642-5 (PMC9584821; doi:10.1038/s42255-022-00642-5)
Supplement: Source Data Extended Data Fig. 1 — Unprocessed western blots. [file 42255_2022_642_MOESM17_ESM.pdf]

Uncropped western blot images

Extended Data Figure 1

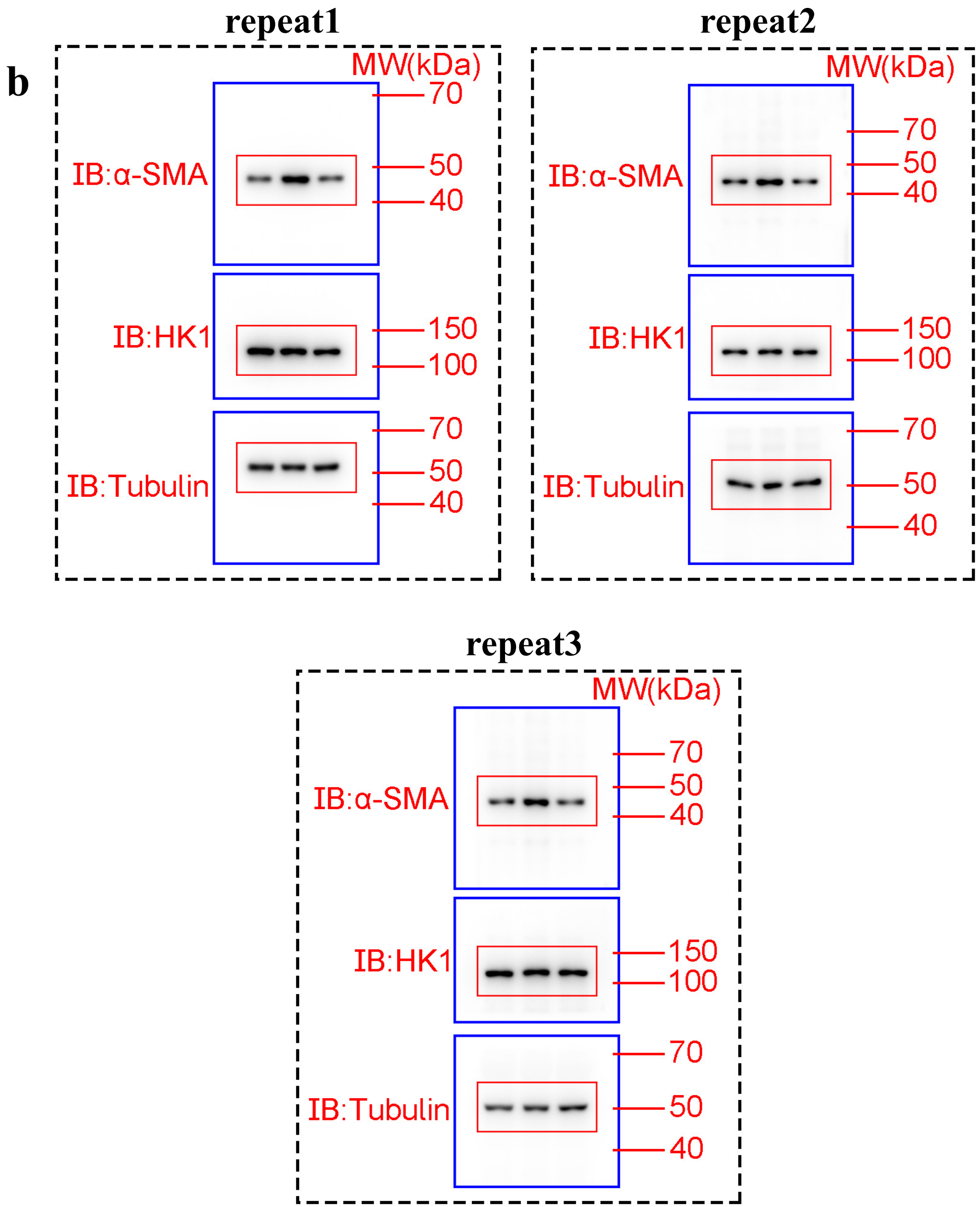

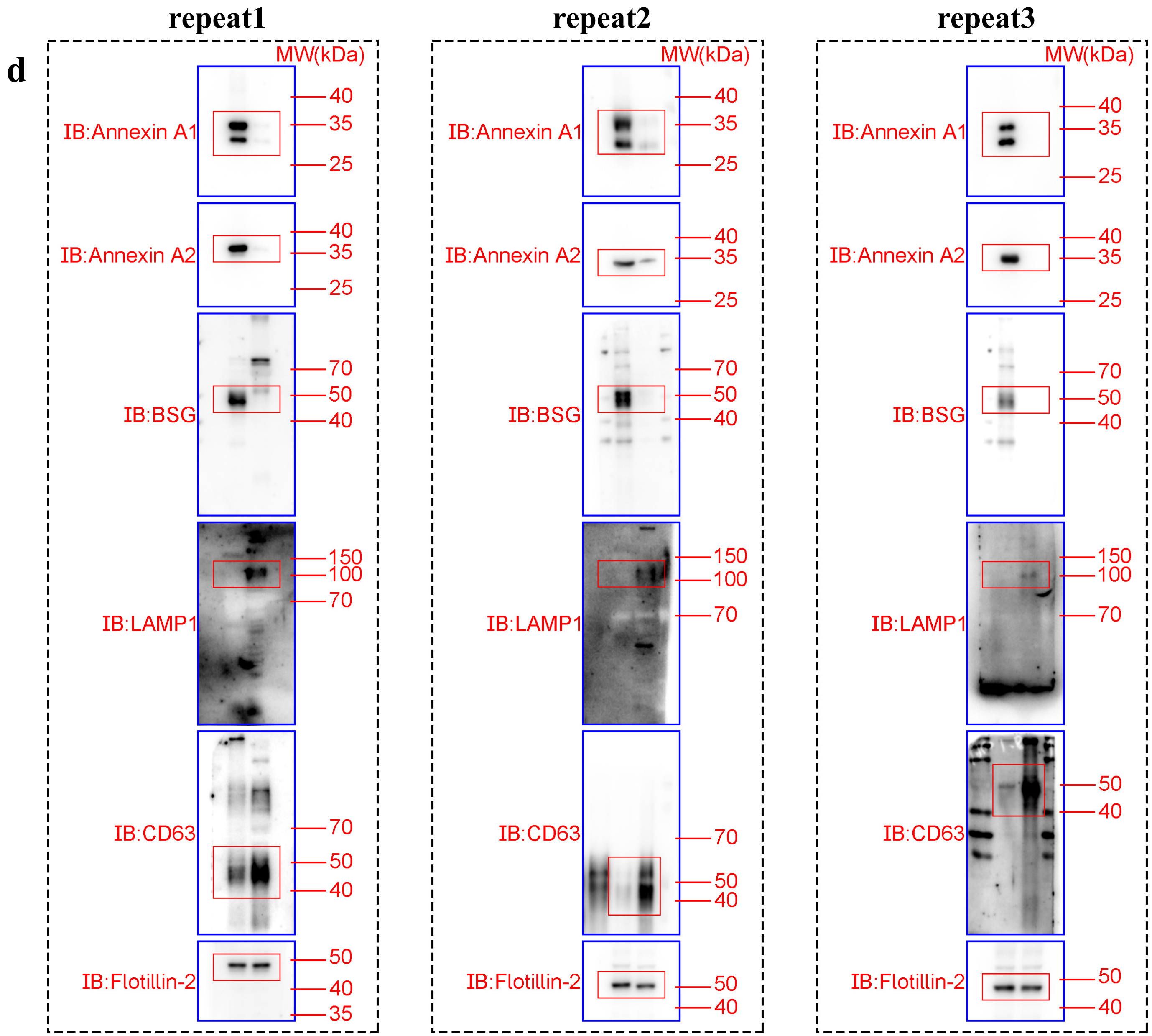

e

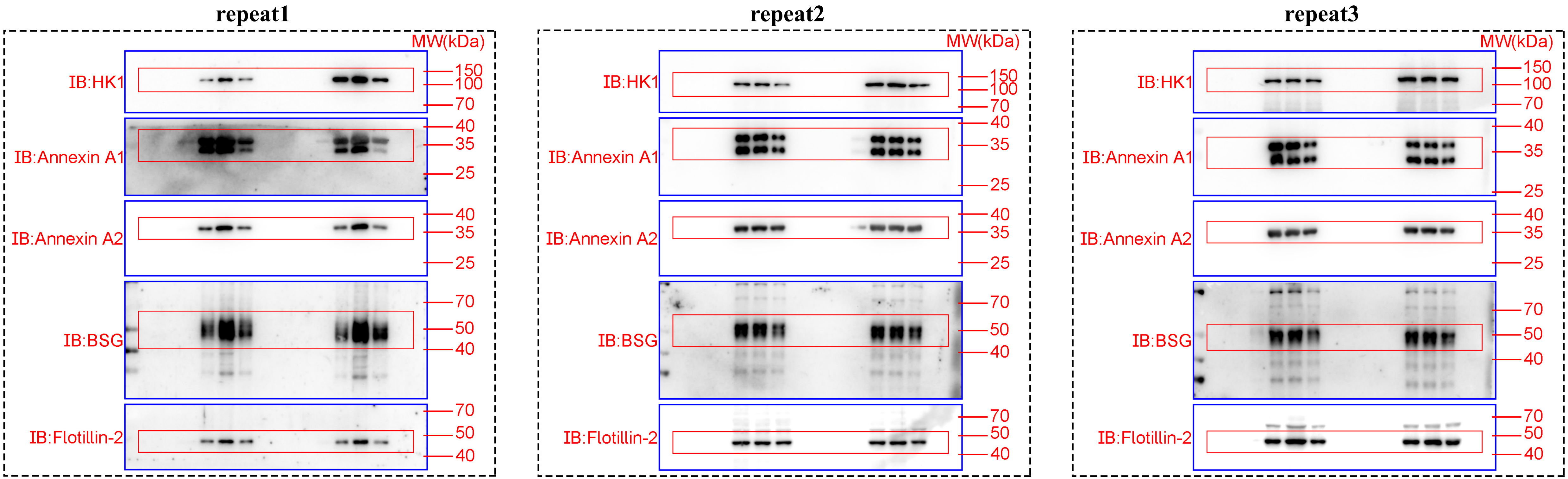
repeat1
repeat2
repeat3
MW(kDa)
IB:HK1
IB:Annexin A1
IB:Annexin A2
IB:BSG
IB:Flotillin-2
150
100
70
40
35
25
50

f

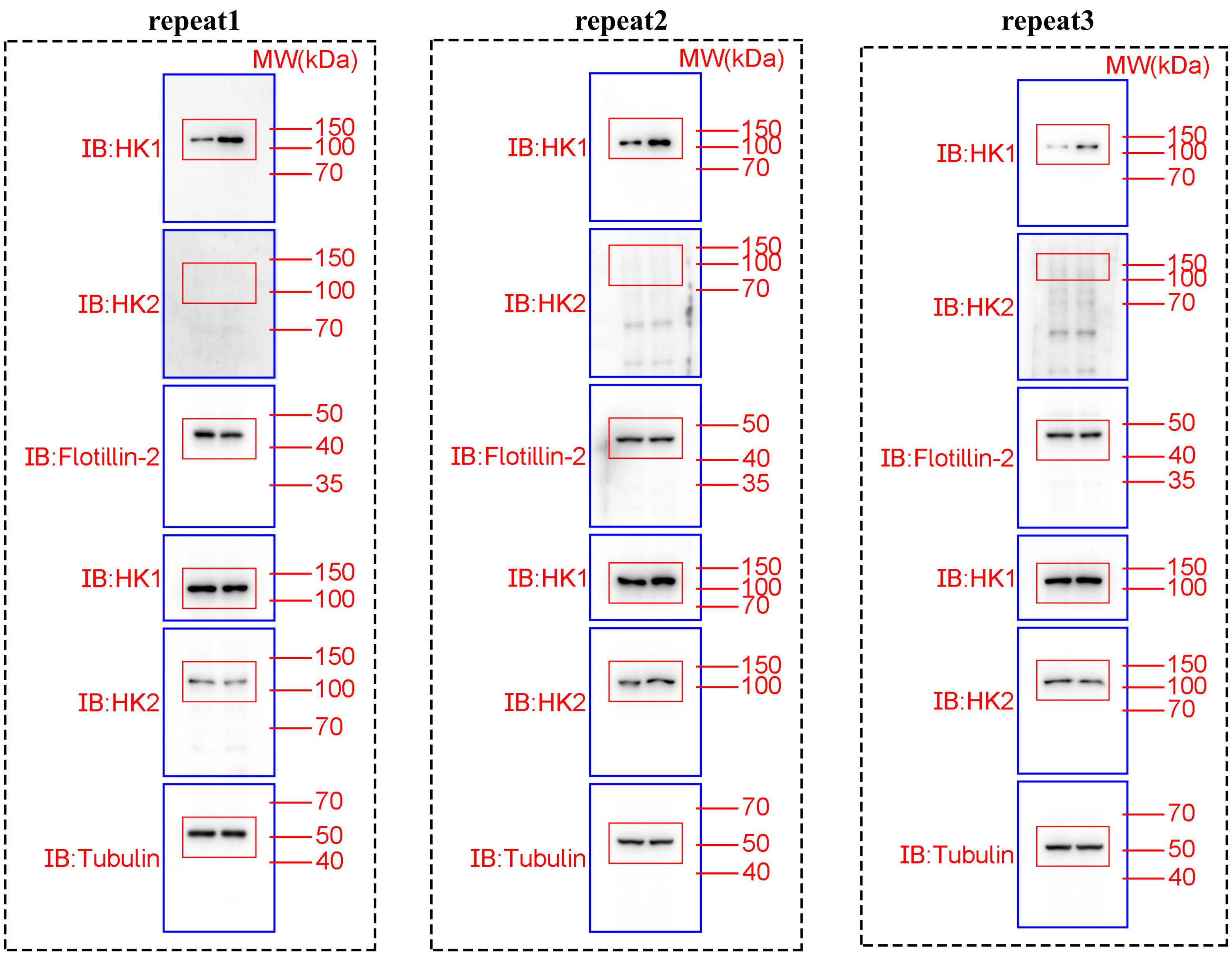
repeat1
repeat2
repeat3
MW(kDa)
IB:HK1
IB:HK2
IB:Flotillin-2
IB:HK1
IB:HK2
IB:Tubulin
150
100
70
50
40
35

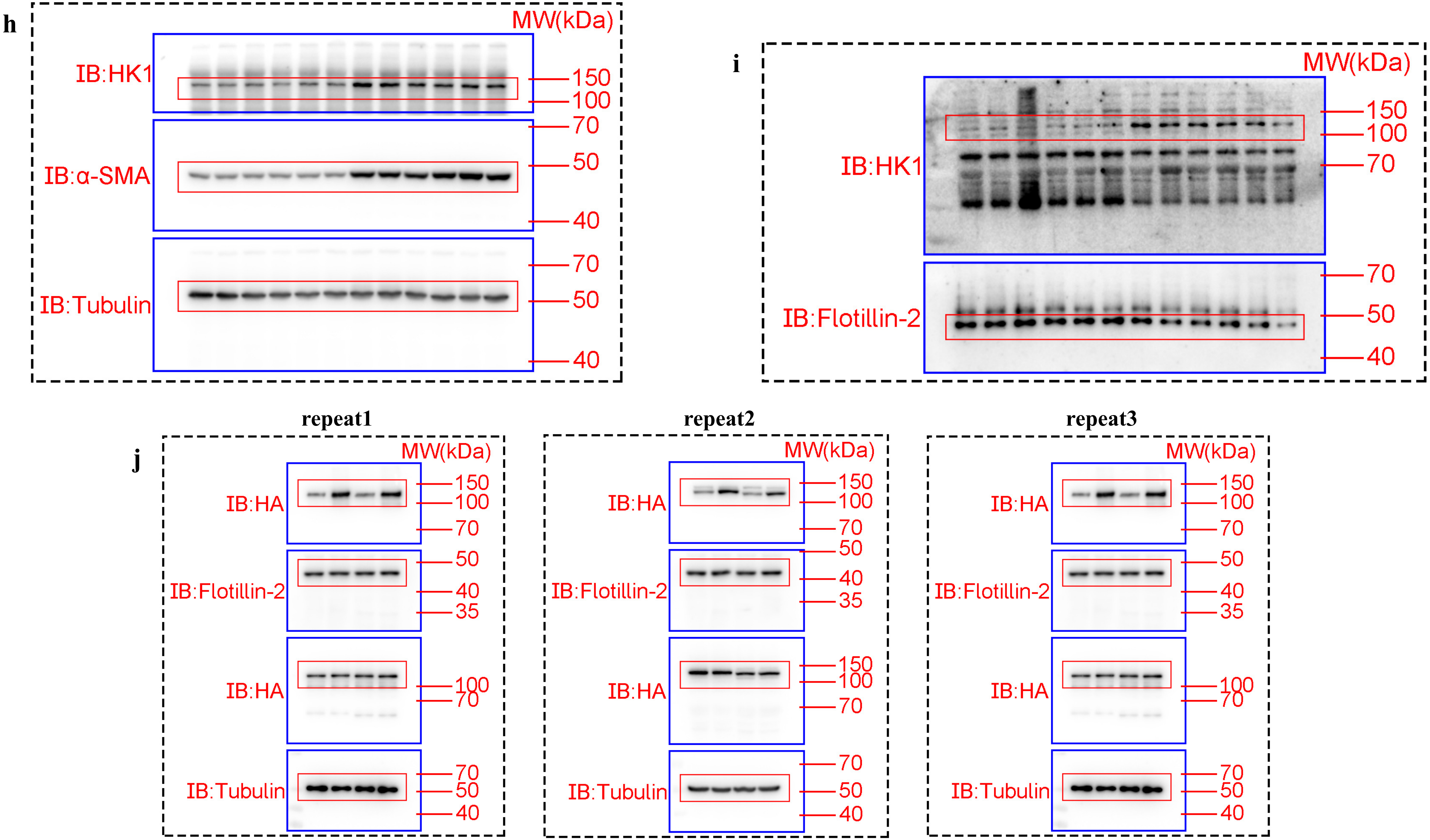

h
MW(kDa)
IB:HK1
150
100
IB:α-SMA
70
50
40
IB:Tubulin
70
50
40
i
MW(kDa)
IB:HK1
150
100
70
IB:Flotillin-2
70
50
40
j
repeat1
repeat2
repeat3
MW(kDa)
IB:HA
150
100
70
IB:Flotillin-2
50
40
35
IB:HA
150
100
70
IB:Tubulin
70
50
40

n

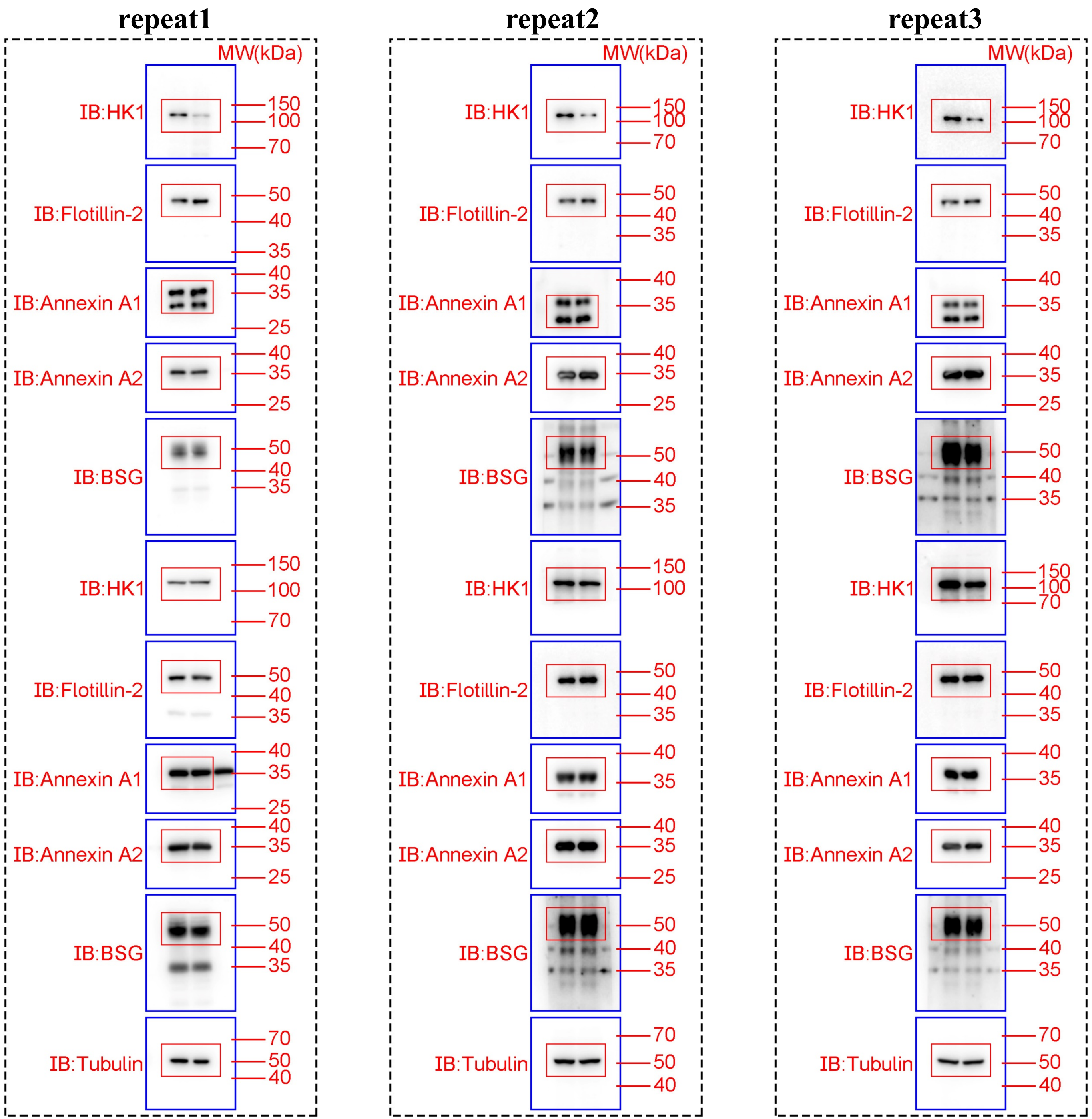
repeat1
repeat2
repeat3
MW(kDa)
IB:HK1
150
100
70
IB:Flotillin-2
50
40
35
IB:Annexin A1
40
35
25
IB:Annexin A2
40
35
25
IB:BSG
50
40
35
IB:HK1
150
100
70
IB:Flotillin-2
50
40
35
IB:Annexin A1
40
35
25
IB:Annexin A2
40
35
25
IB:BSG
50
40
35
IB:Tubulin
70
50
40
