# Supplementary material for: HK1 from hepatic stellate cell–derived extracellular vesicles promotes progression of hepatocellular carcinoma
Source: Nat Metab. 2022 Oct 3;4(10):1306–21. doi: 10.1038/s42255-022-00642-5 (PMC9584821; doi:10.1038/s42255-022-00642-5)
Supplement: Source Data Extended Data Fig. 2 — Unprocessed western blots. [file 42255_2022_642_MOESM19_ESM.pdf]

Uncropped western blot images
Extended Data Figure 2

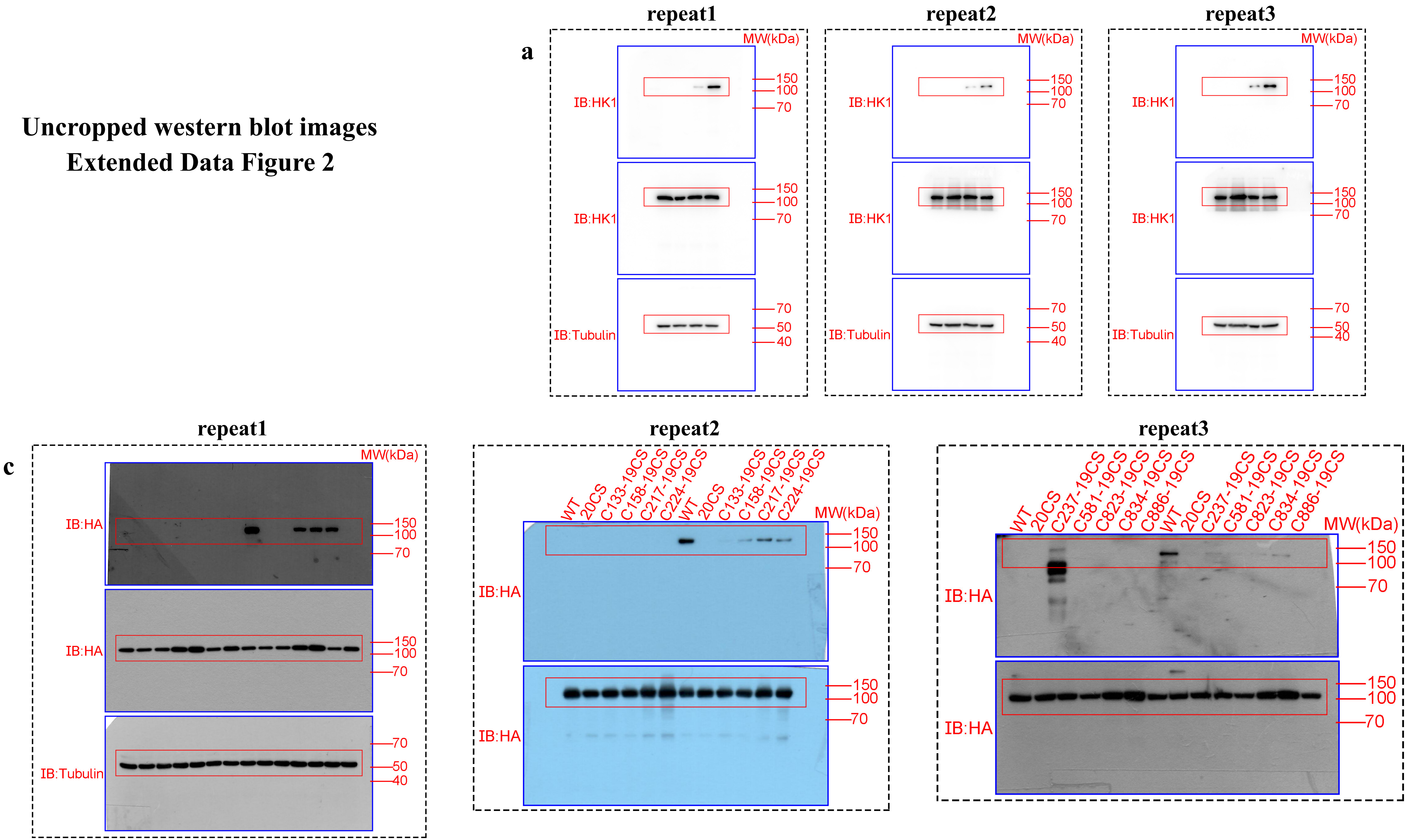

repeat1

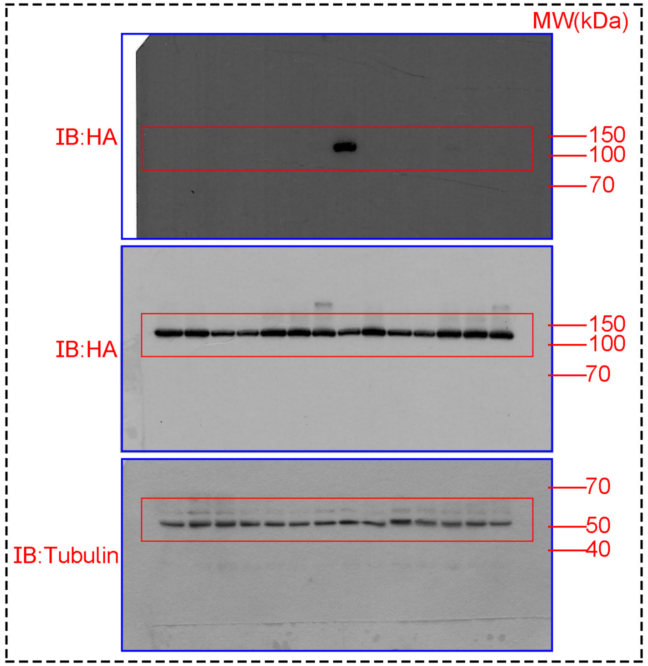

repeat2

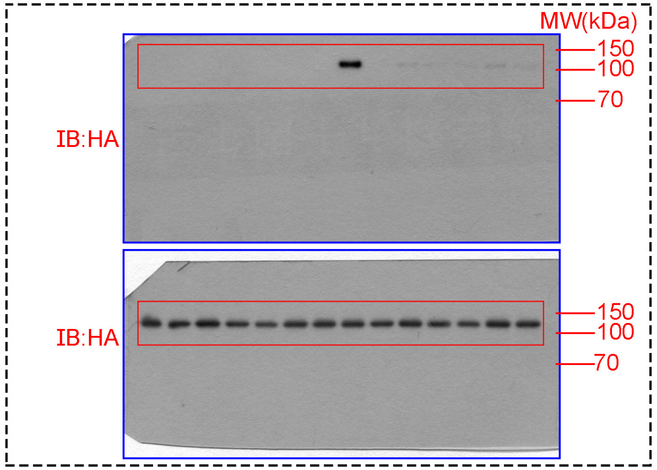

repeat1

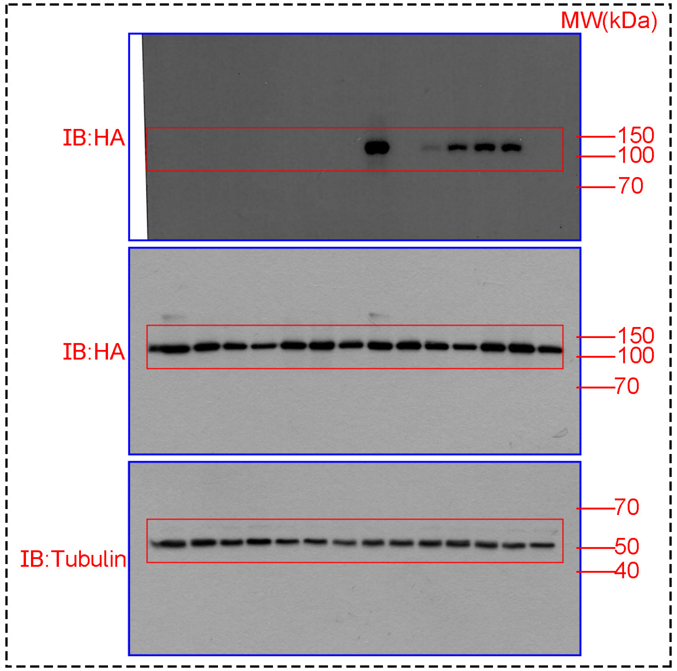

repeat2

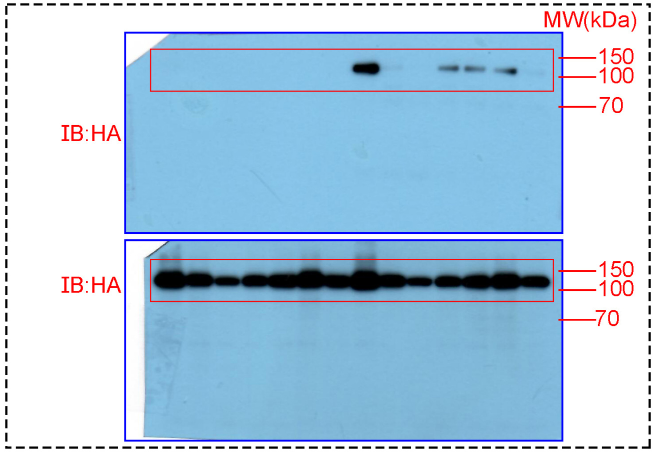

repeat1

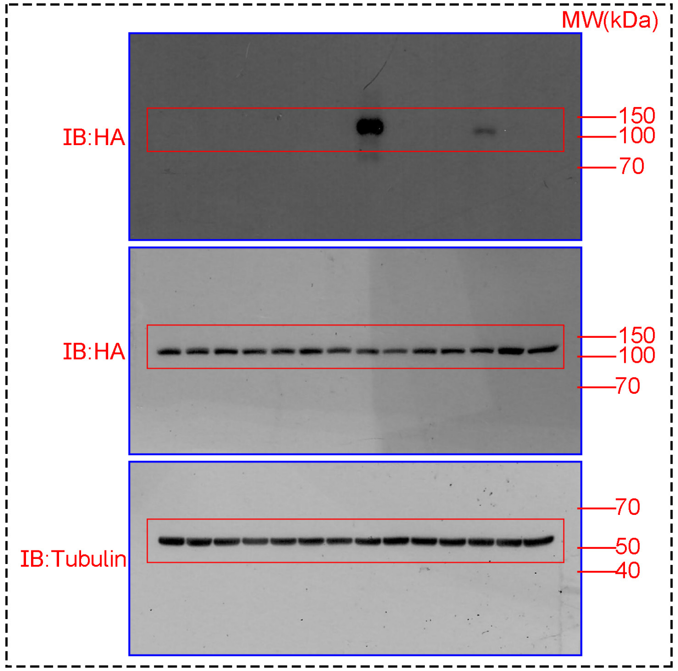

repeat2

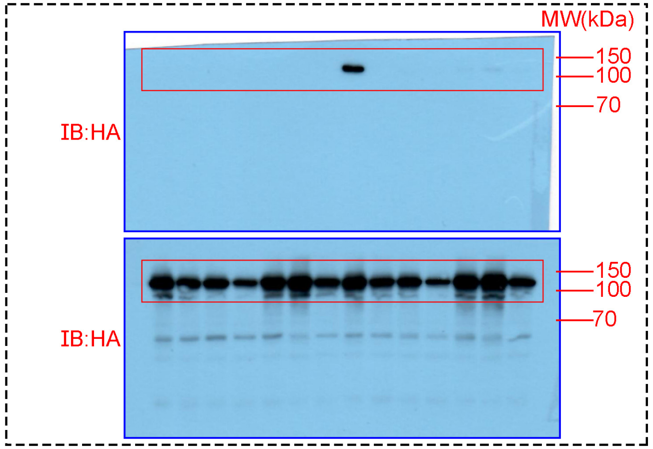

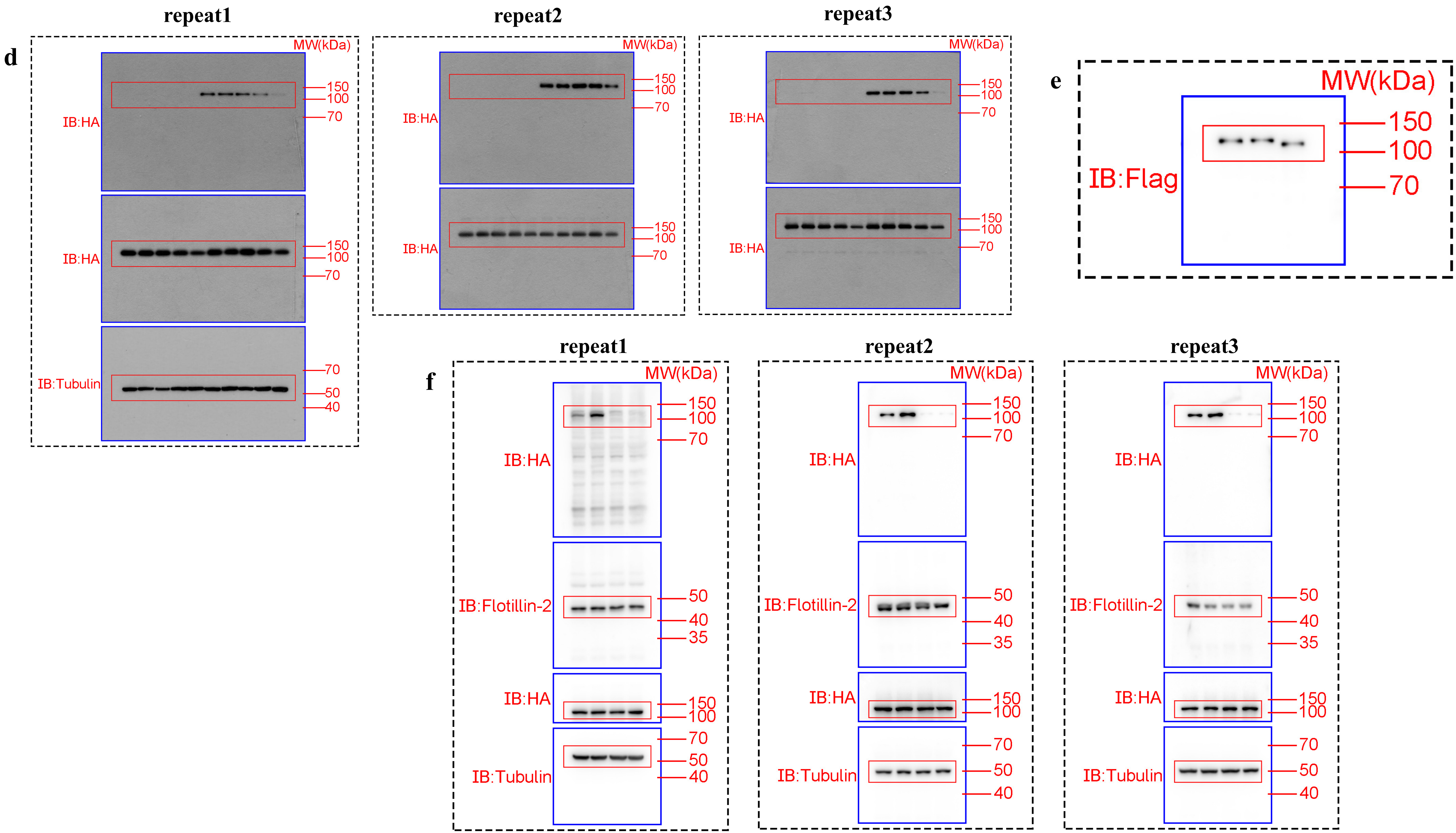

repeat1
repeat2
repeat3
d
MW(kDa)
IB:HA
IB:Tubulin
150
100
70
50
40
e
IB:Flag
f
IB:Flotillin-2
35

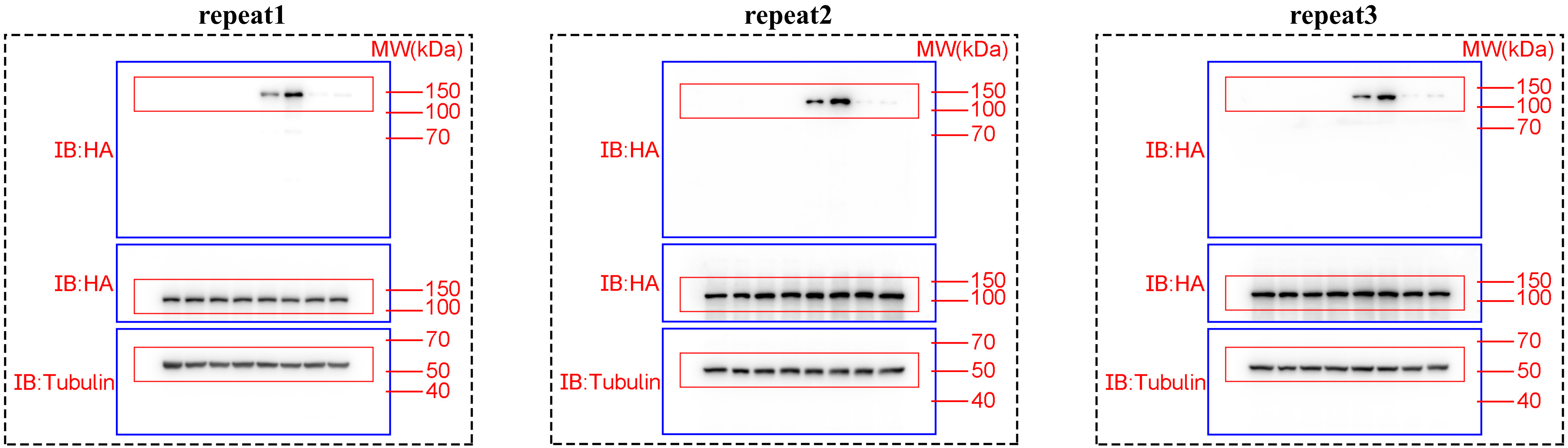
repeat1
repeat2
repeat3
MW(kDa)
IB:HA
IB:HA
IB:Tubulin
150
100
70
150
100
70
50
40

g

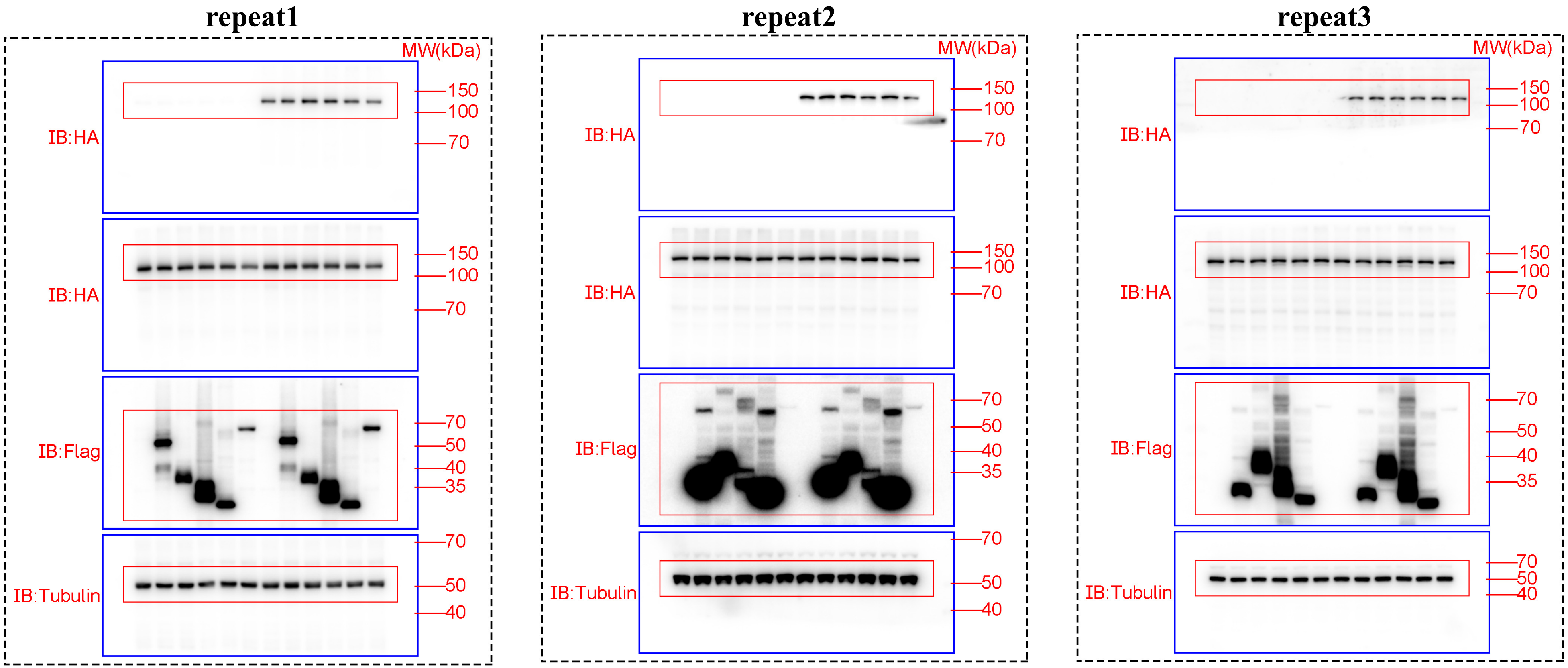
repeat1
repeat2
repeat3
MW(kDa)
IB:HA
IB:HA
IB:Flag
IB:Tubulin
150
100
70
150
100
70
70
50
40
35
70
50
40

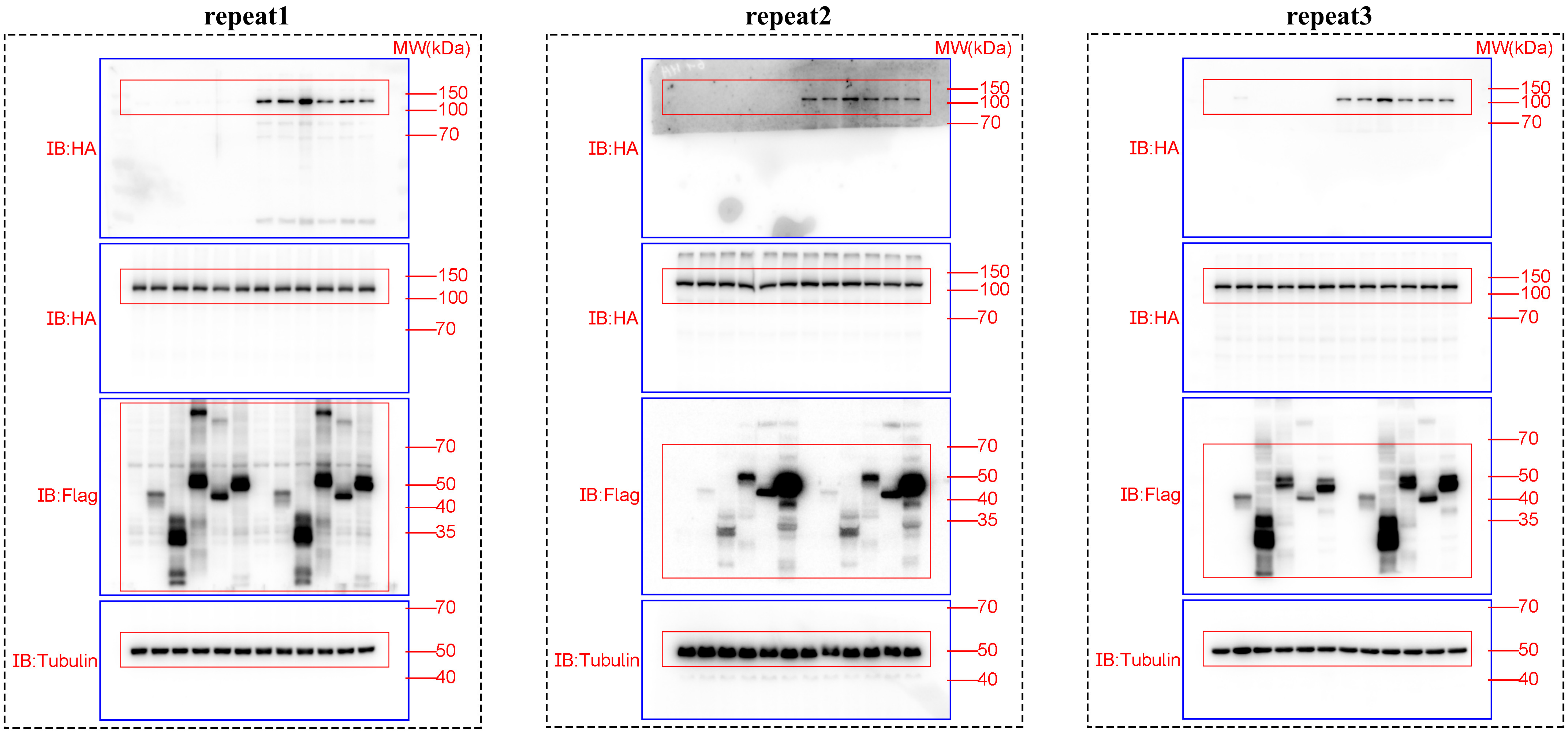
repeat1
repeat2
repeat3
MW(kDa)
IB:HA
IB:HA
IB:Flag
IB:Tubulin
150
100
70
50
40
35

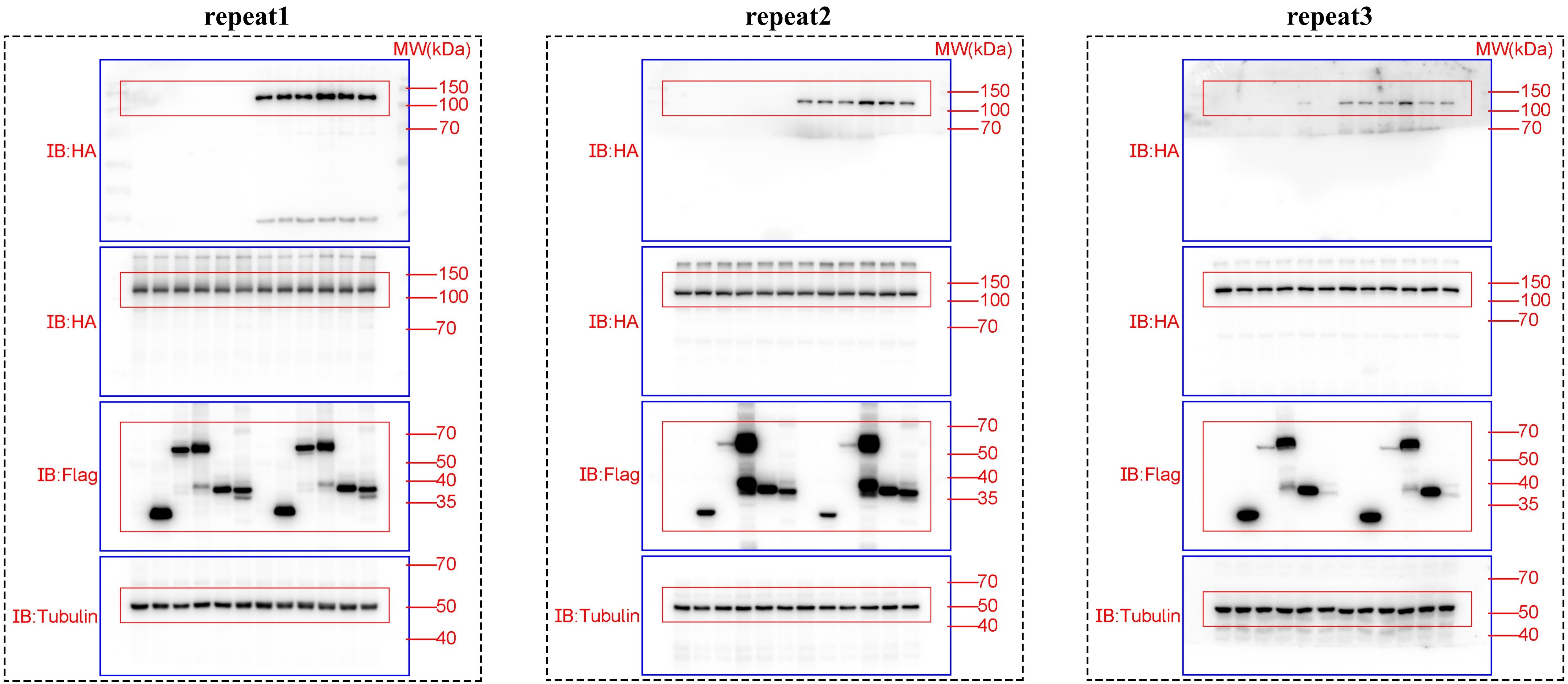
repeat1
repeat2
repeat3
MW(kDa)
IB:HA
IB:HA
IB:Flag
IB:Tubulin
150
100
70
50
40
35

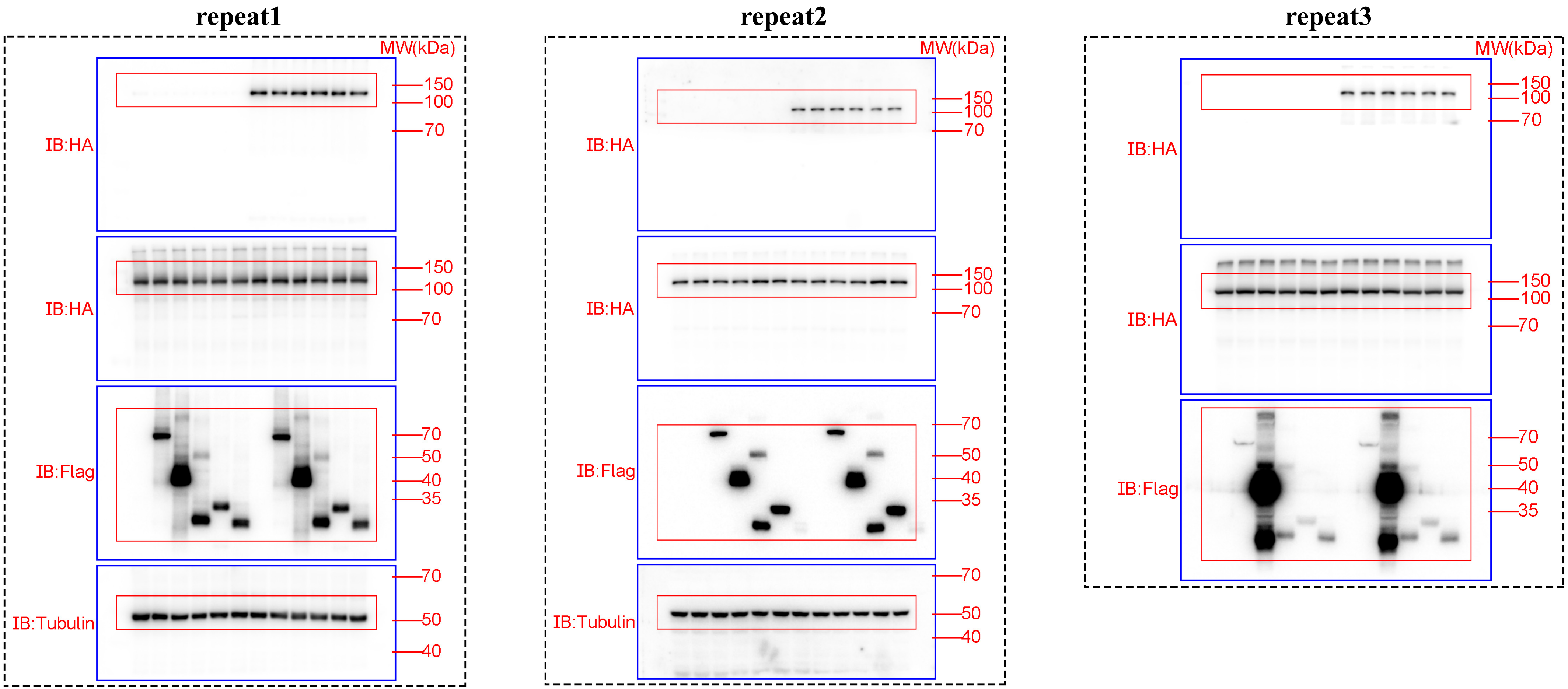
repeat1
repeat2
repeat3
MW(kDa)
IB:HA
IB:Flag
IB:Tubulin
150
100
70
50
40
35

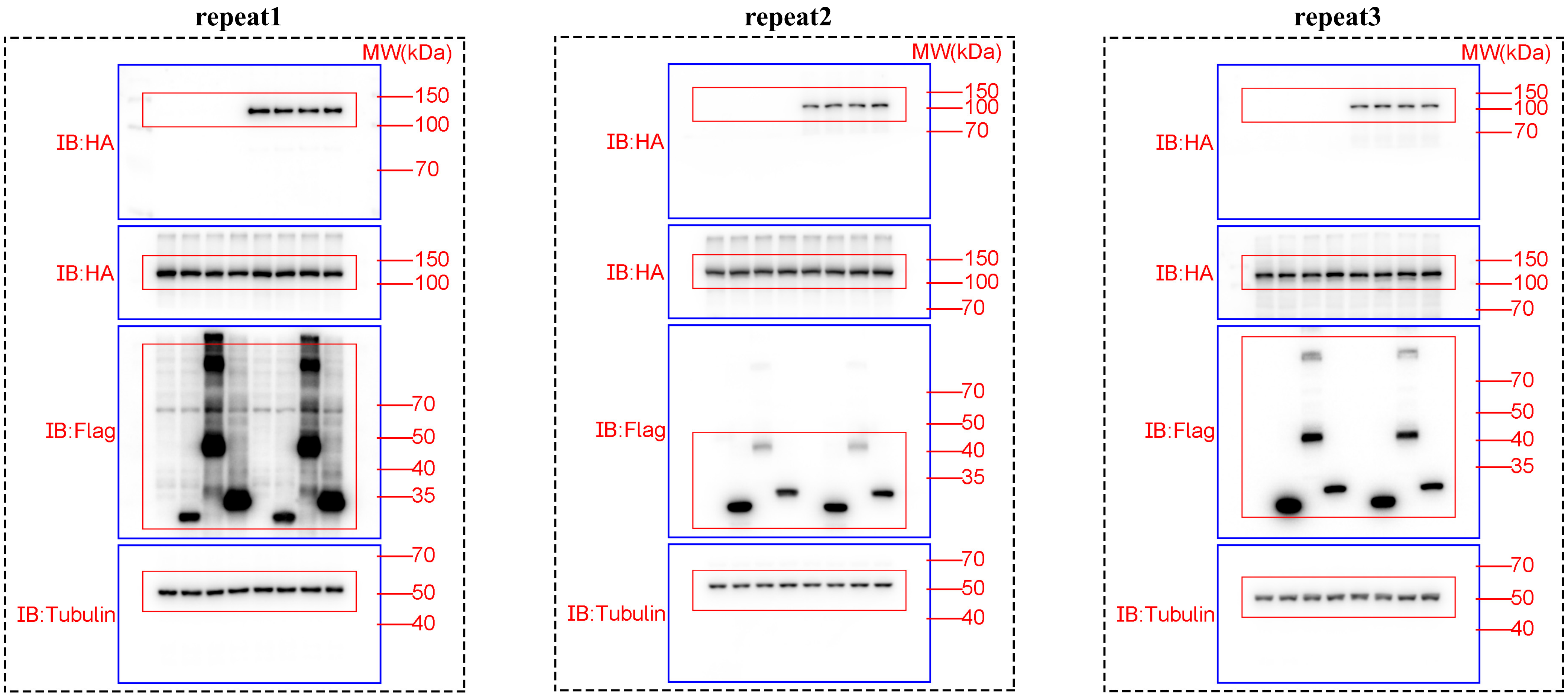
repeat1
repeat2
repeat3
MW(kDa)
IB:HA
IB:HA
IB:Flag
IB:Tubulin
150
100
70
50
40
35

h

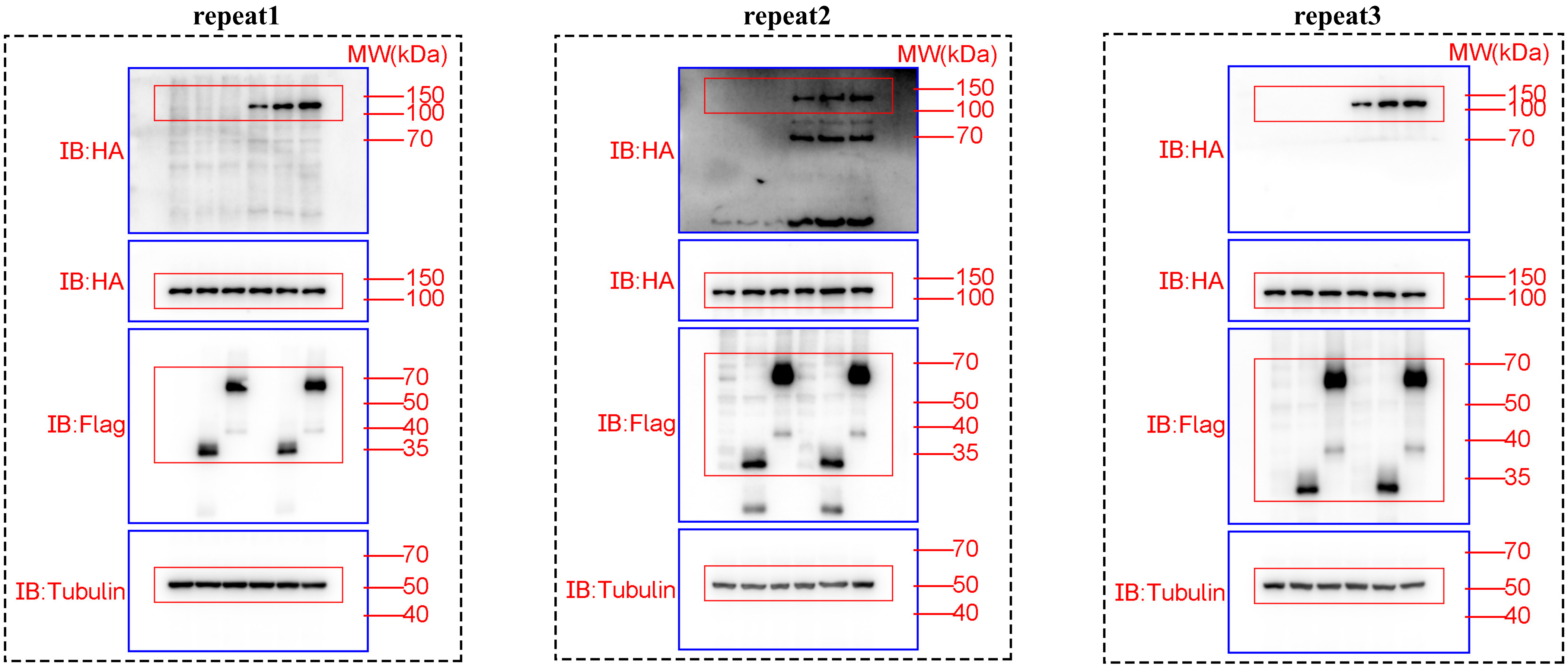
repeat1
MW(kDa)
IB:HA
150
100
70
IB:HA
150
100
IB:Flag
70
50
40
35
IB:Tubulin
70
50
40
repeat2
MW(kDa)
IB:HA
150
100
70
IB:HA
150
100
IB:Flag
70
50
40
35
IB:Tubulin
70
50
40
repeat3
MW(kDa)
IB:HA
150
100
70
IB:HA
150
100
IB:Flag
70
50
40
35
IB:Tubulin
70
50
40

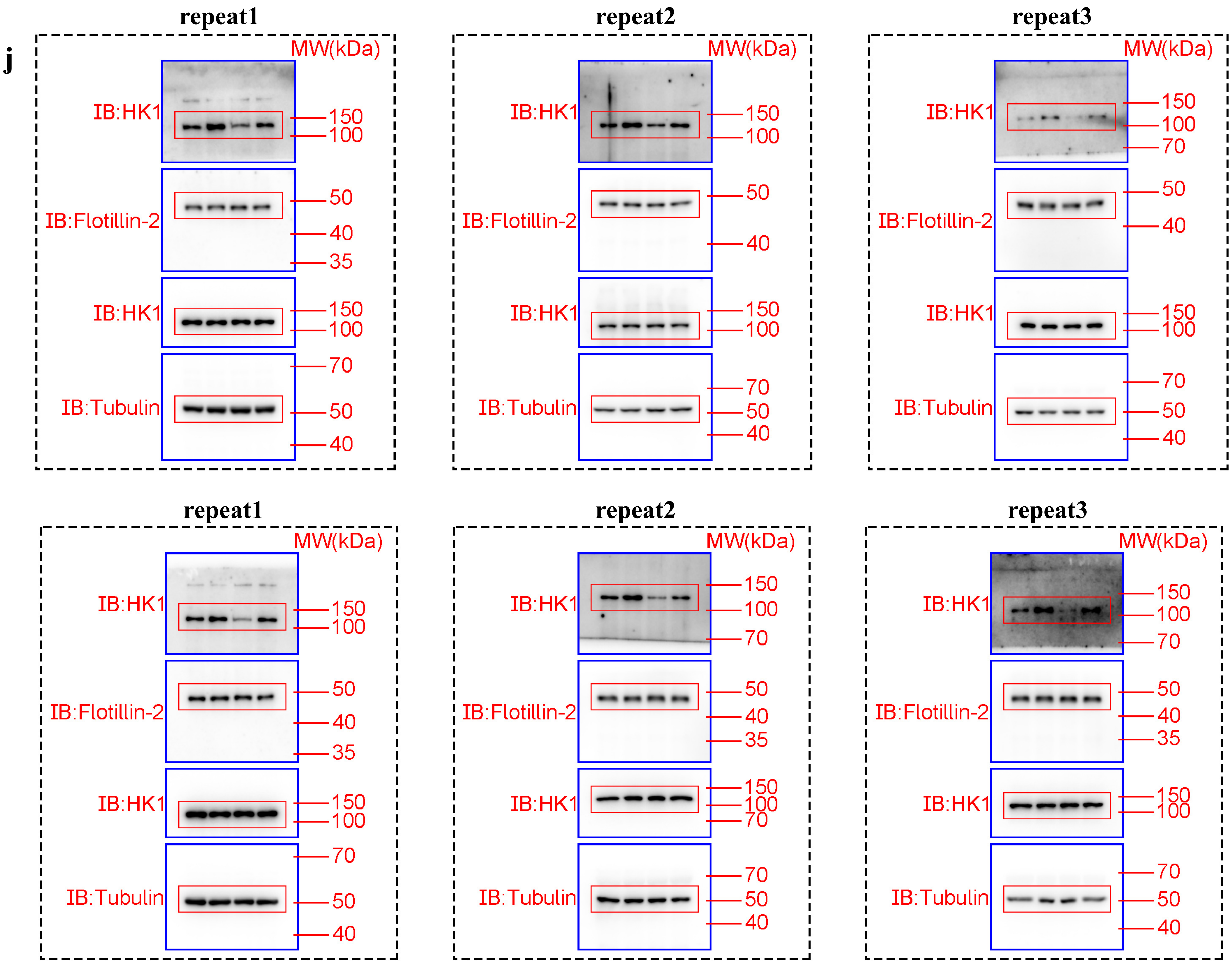
j
repeat1
MW(kDa)
IB:HK1
150
100
IB:Flotillin-2
50
40
35
IB:HK1
150
100
IB:Tubulin
70
50
40
repeat2
MW(kDa)
IB:HK1
150
100
IB:Flotillin-2
50
40
IB:HK1
150
100
IB:Tubulin
70
50
40
repeat3
MW(kDa)
IB:HK1
150
100
70
IB:Flotillin-2
50
40
IB:HK1
150
100
IB:Tubulin
70
50
40
repeat1
MW(kDa)
IB:HK1
150
100
IB:Flotillin-2
50
40
35
IB:HK1
150
100
IB:Tubulin
70
50
40
repeat2
MW(kDa)
IB:HK1
150
100
70
IB:Flotillin-2
50
40
35
IB:HK1
150
100
70
IB:Tubulin
70
50
40
repeat3
MW(kDa)
IB:HK1
150
100
70
IB:Flotillin-2
50
40
35
IB:HK1
150
100
IB:Tubulin
70
50
40

l

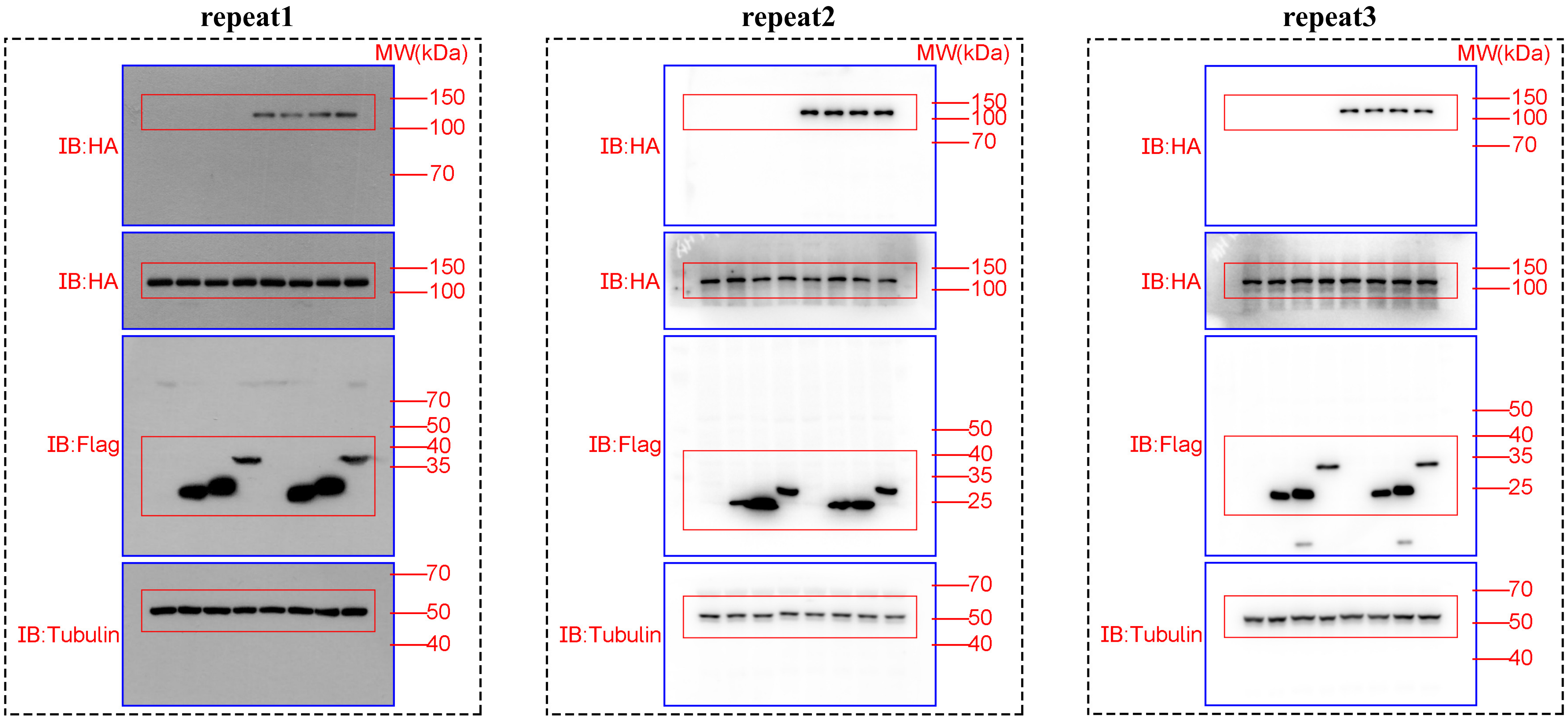
repeat1
MW(kDa)
IB:HA
150
100
70
IB:HA
150
100
IB:Flag
70
50
40
35
IB:Tubulin
70
50
40
repeat2
MW(kDa)
IB:HA
150
100
70
IB:HA
150
100
IB:Flag
50
40
35
25
IB:Tubulin
70
50
40
repeat3
MW(kDa)
IB:HA
150
100
70
IB:HA
150
100
IB:Flag
50
40
35
25
IB:Tubulin
70
50
40

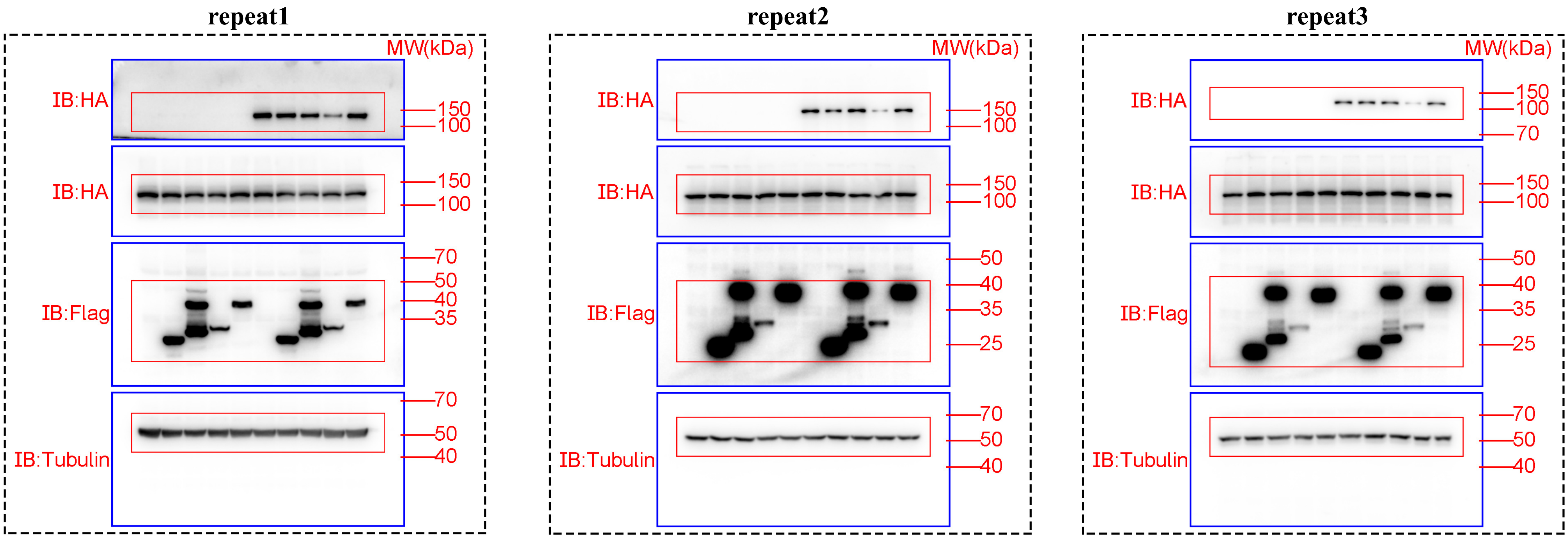
repeat1
repeat2
repeat3
MW(kDa)
IB:HA
150
100
70
IB:Flag
50
40
35
25
IB:Tubulin

m

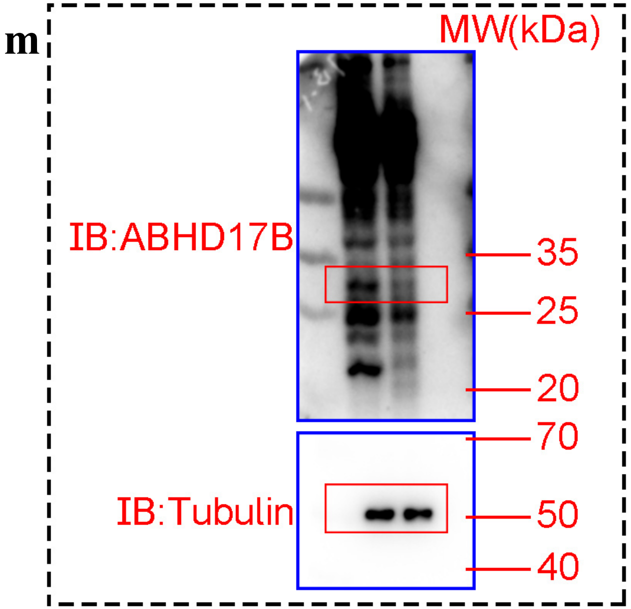
MW(kDa)
IB:ABHD17B
35
25
20
IB:Tubulin
70
50
40

n

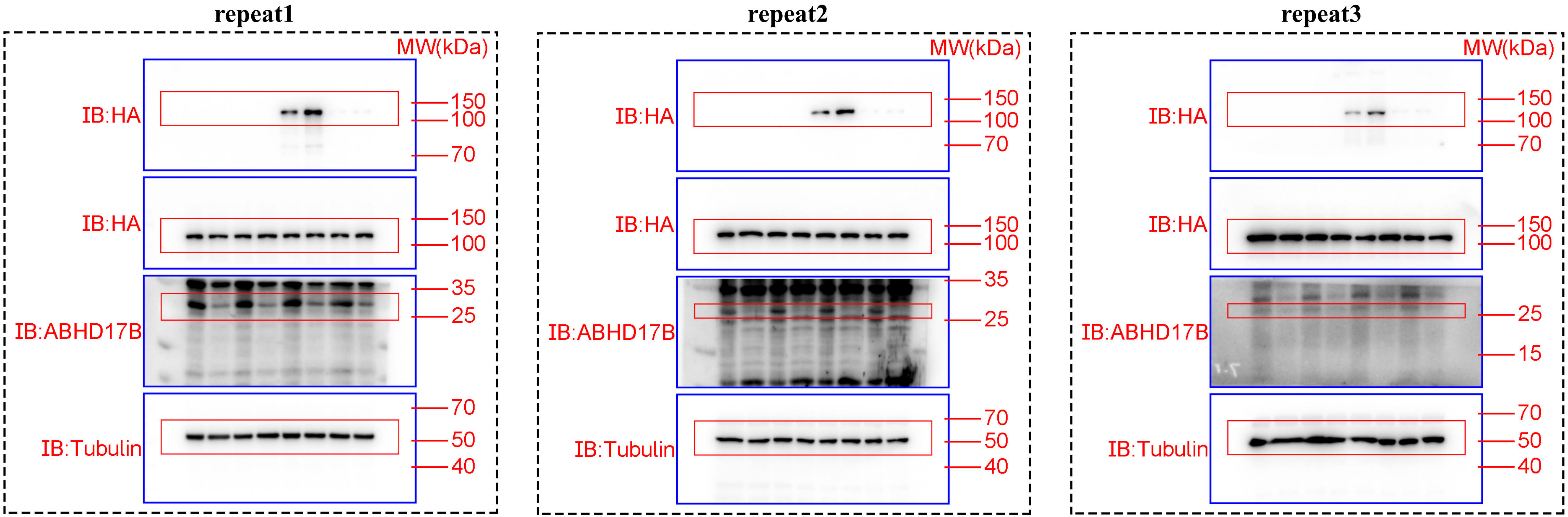
repeat1
repeat2
repeat3
MW(kDa)
IB:HA
IB:HA
IB:ABHD17B
IB:Tubulin
150
100
70
150
100
35
25
15
70
50
40

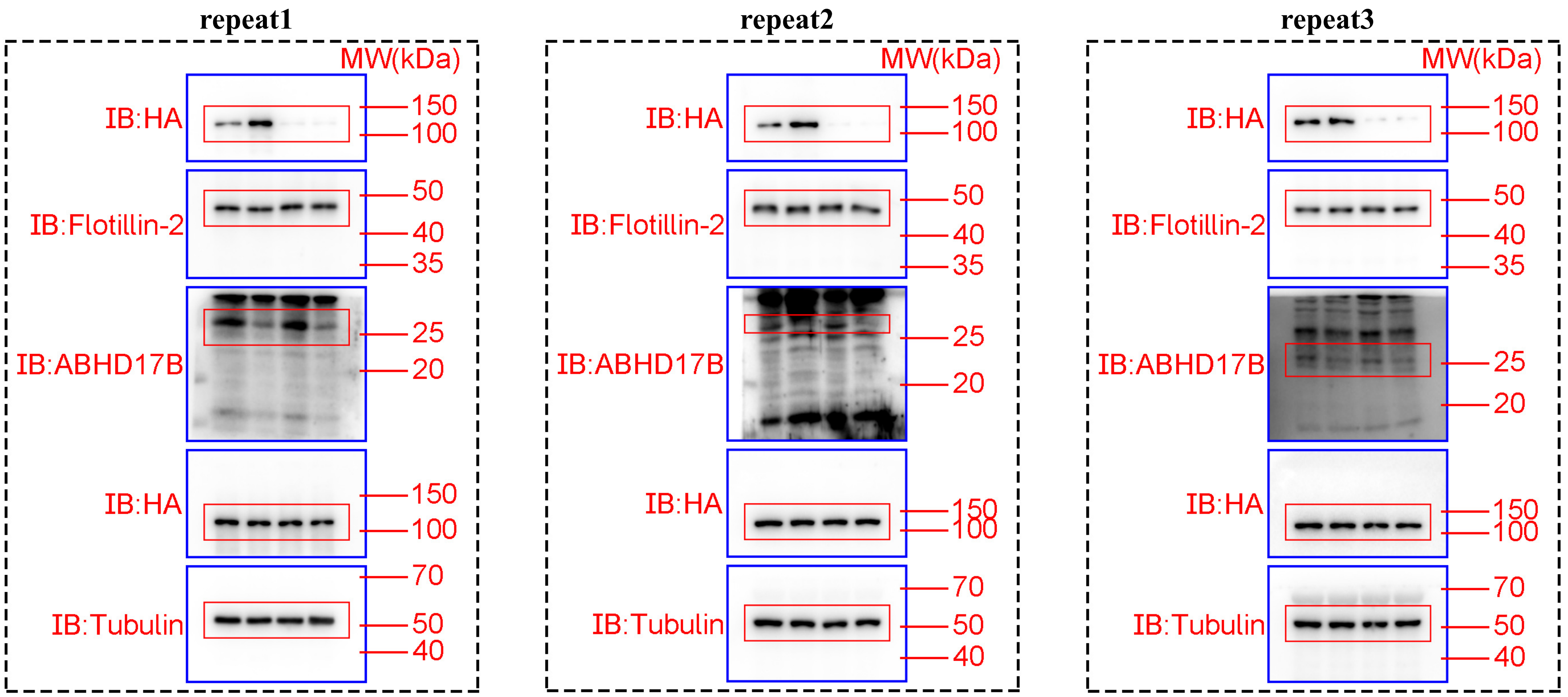
repeat1
repeat2
repeat3
MW(kDa)
IB:HA
IB:Flotillin-2
IB:ABHD17B
IB:HA
IB:Tubulin
150
100
50
40
35
25
20
150
100
70
50
40

o

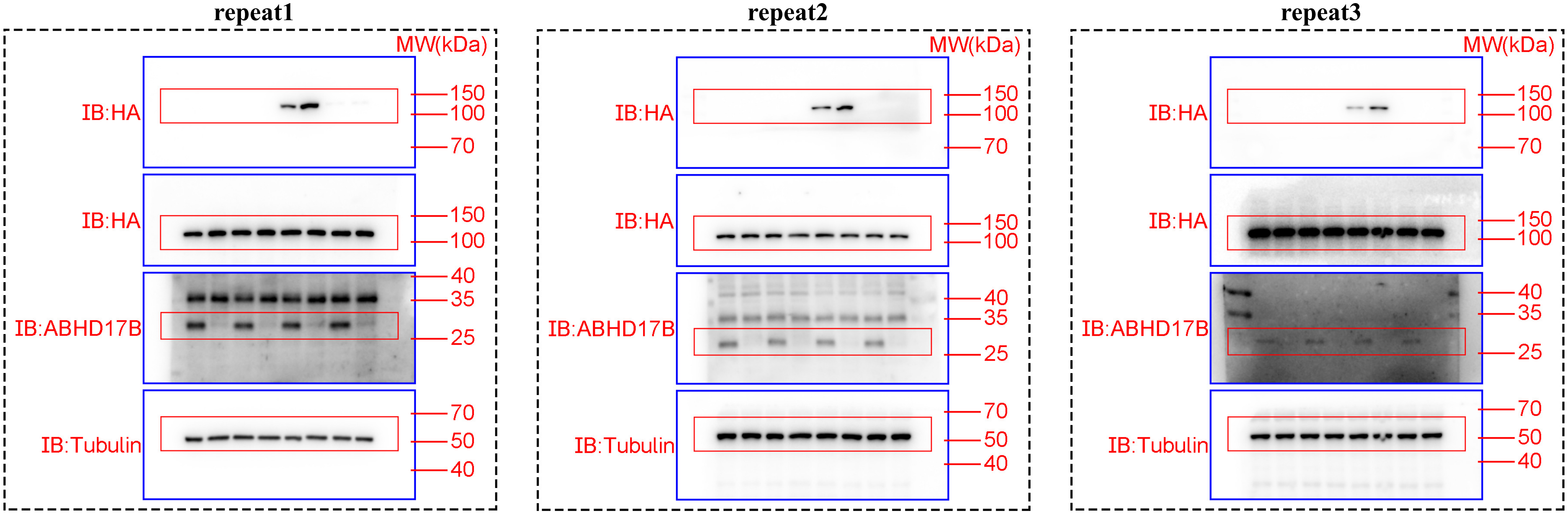

repeat1
repeat2
repeat3
MW(kDa)
IB:HA
IB:HA
IB:ABHD17B
IB:Tubulin
150
100
70
40
35
25
50

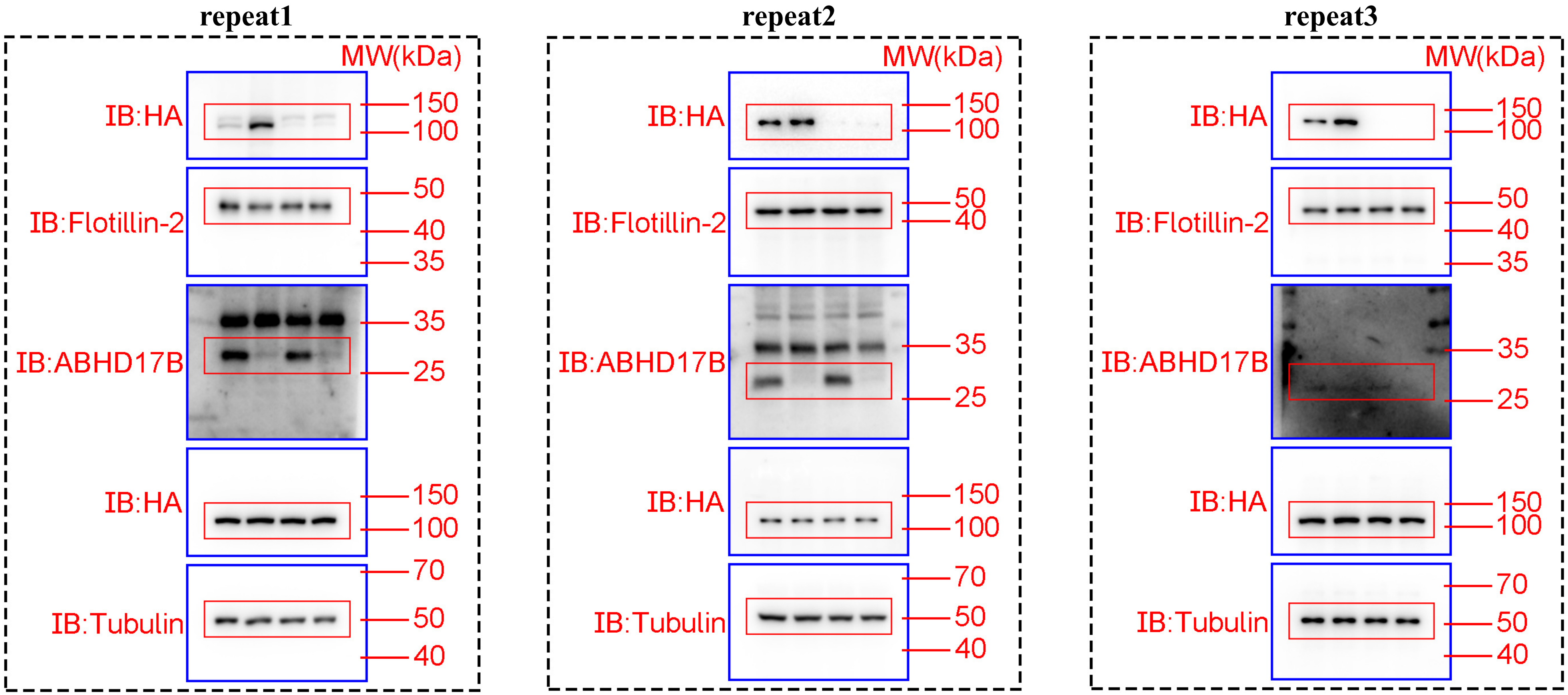
repeat1
repeat2
repeat3
MW(kDa)
IB:HA
150
100
IB:Flotillin-2
50
40
35
IB:ABHD17B
35
25
IB:HA
150
100
IB:Tubulin
70
50
40
