# Supplementary material for: HK1 from hepatic stellate cell–derived extracellular vesicles promotes progression of hepatocellular carcinoma
Source: Nat Metab. 2022 Oct 3;4(10):1306–21. doi: 10.1038/s42255-022-00642-5 (PMC9584821; doi:10.1038/s42255-022-00642-5)

Uncropped western blot images
Extended Data Figure 5

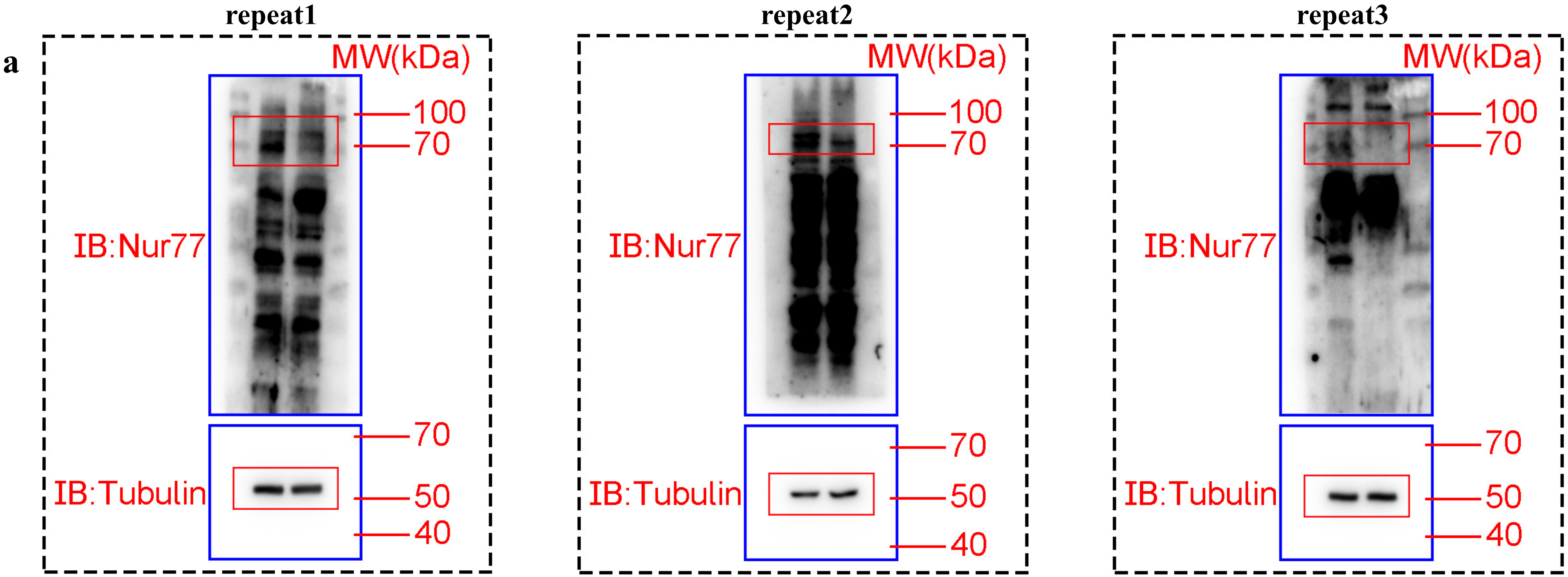

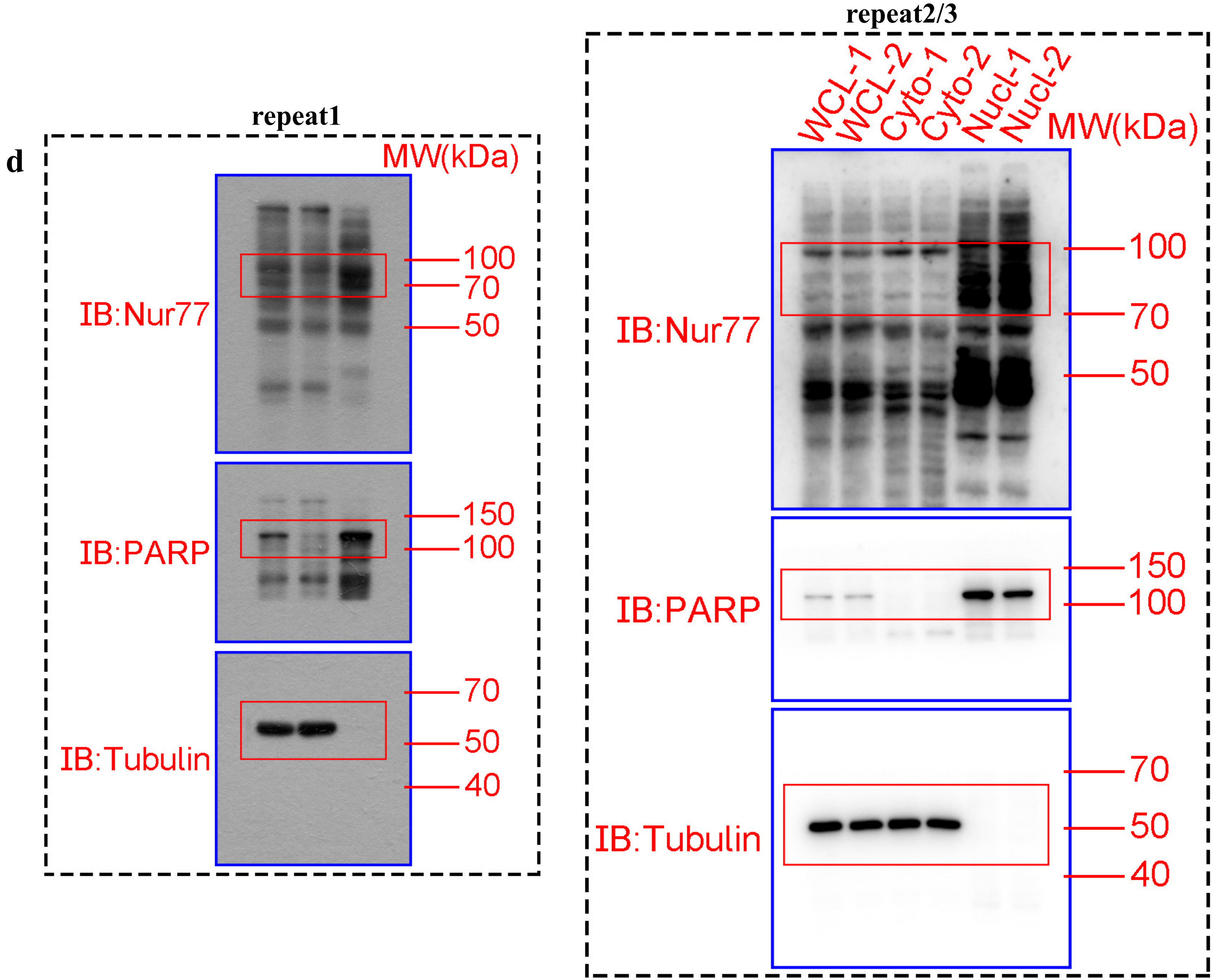
d
repeat1
MW(kDa)
IB:Nur77
100
70
50
IB:PARP
150
100
IB:Tubulin
70
50
40
repeat2/3
WCL-1
WCL-2
Cyto-1
Cyto-2
Nucl-1
Nucl-2
MW(kDa)
IB:Nur77
100
70
50
IB:PARP
150
100
IB:Tubulin
70
50
40

f

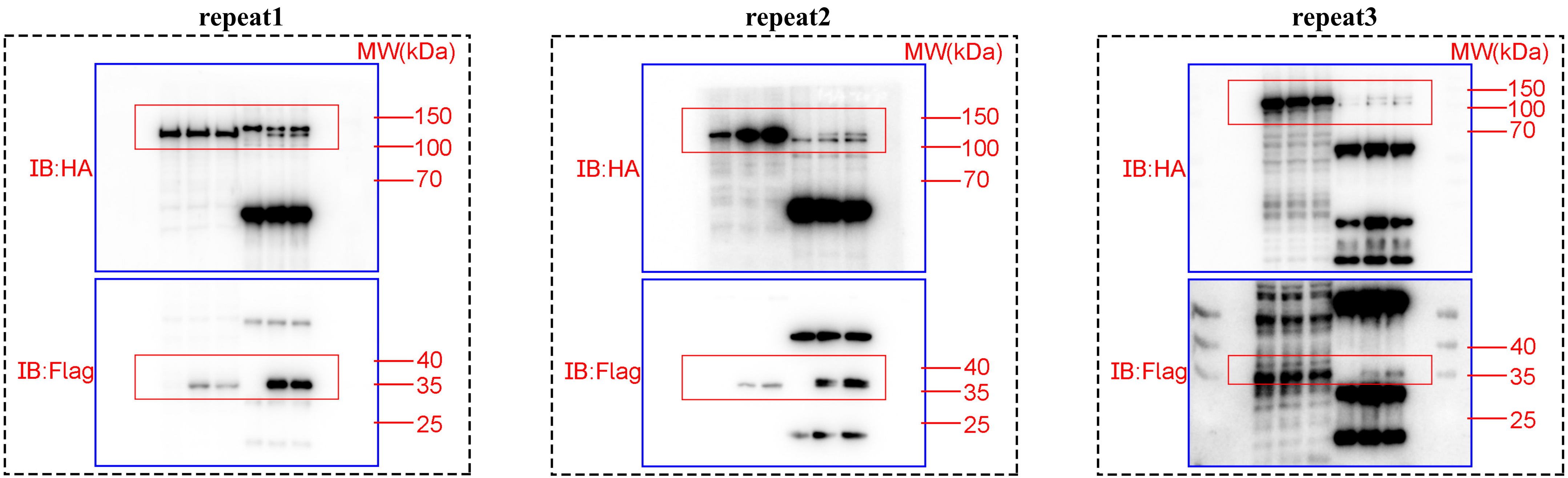
repeat1
MW(kDa)
IB:HA
150
100
70
IB:Flag
40
35
25
repeat2
MW(kDa)
IB:HA
150
100
70
IB:Flag
40
35
25
repeat3
MW(kDa)
IB:HA
150
100
70
IB:Flag
40
35
25

g

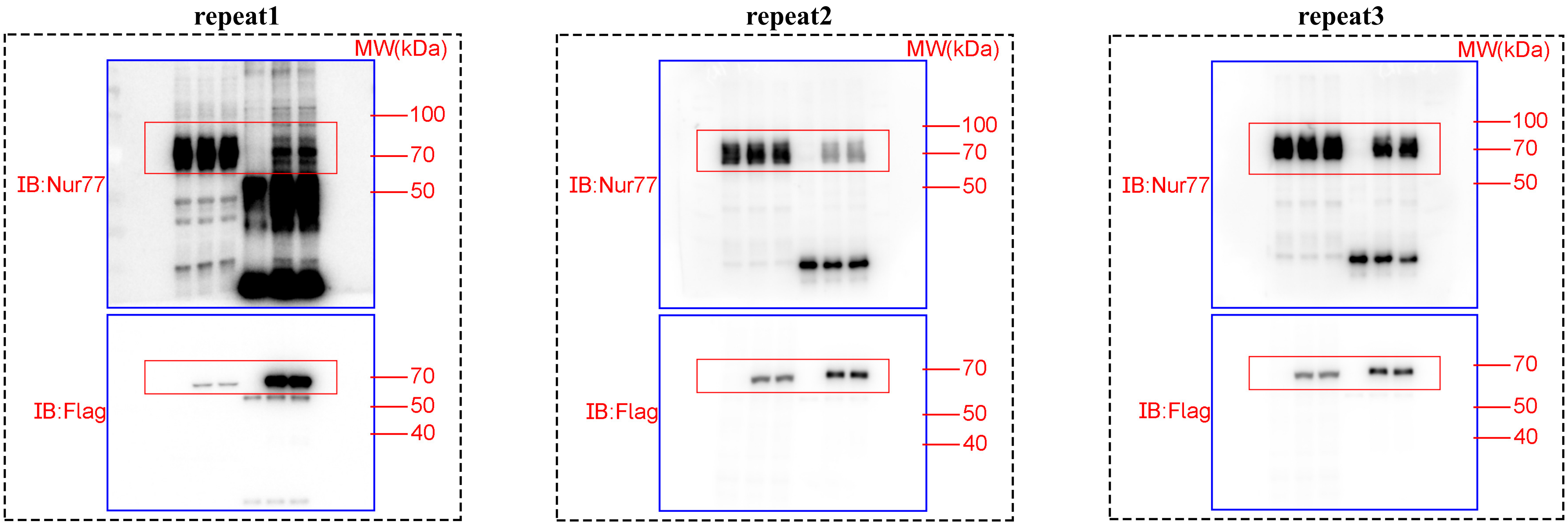
repeat1
MW(kDa)
IB:Nur77
100
70
50
IB:Flag
70
50
40
repeat2
MW(kDa)
IB:Nur77
100
70
50
IB:Flag
70
50
40
repeat3
MW(kDa)
IB:Nur77
100
70
50
IB:Flag
70
50
40

h

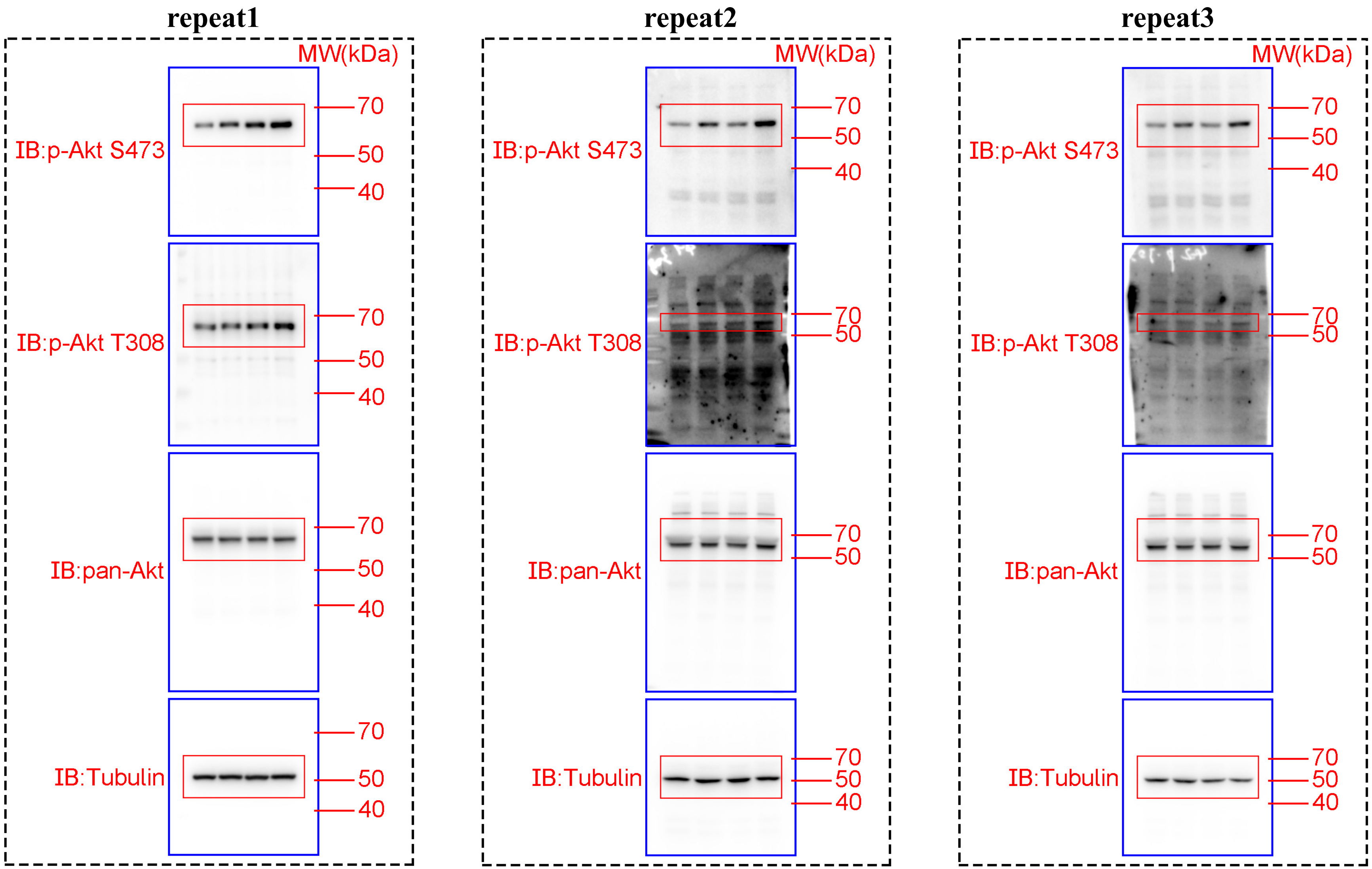
repeat1
repeat2
repeat3
MW(kDa)
IB:p-Akt S473
IB:p-Akt T308
IB:pan-Akt
IB:Tubulin
70
50
40

i

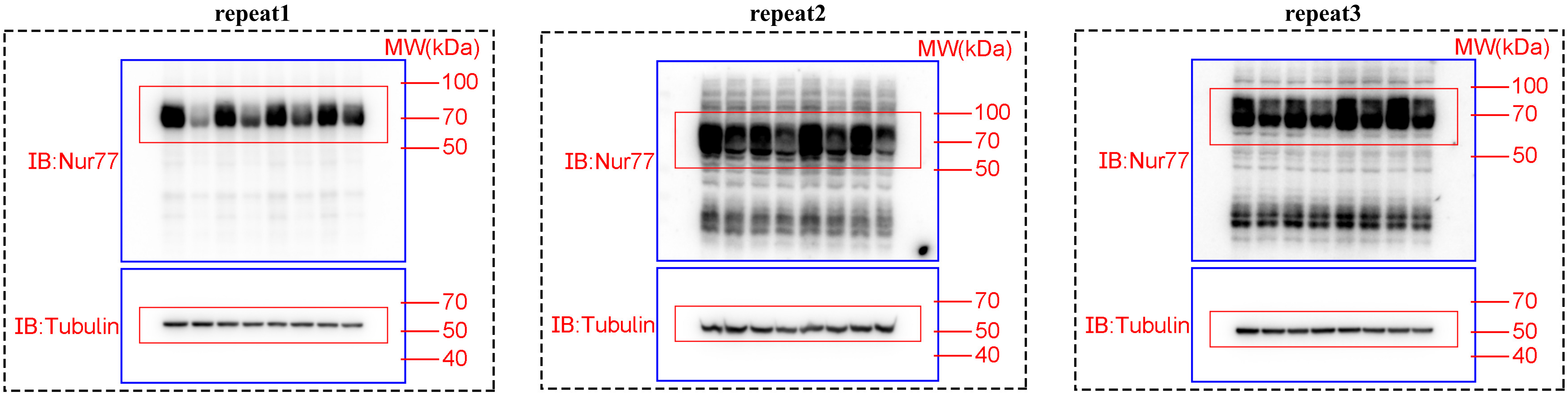

repeat1
MW(kDa)
100
70
50
IB:Nur77
70
50
40
IB:Tubulin
repeat2
MW(kDa)
100
70
50
IB:Nur77
70
50
40
IB:Tubulin
repeat3
MW(kDa)
100
70
50
IB:Nur77
70
50
40
IB:Tubulin

j

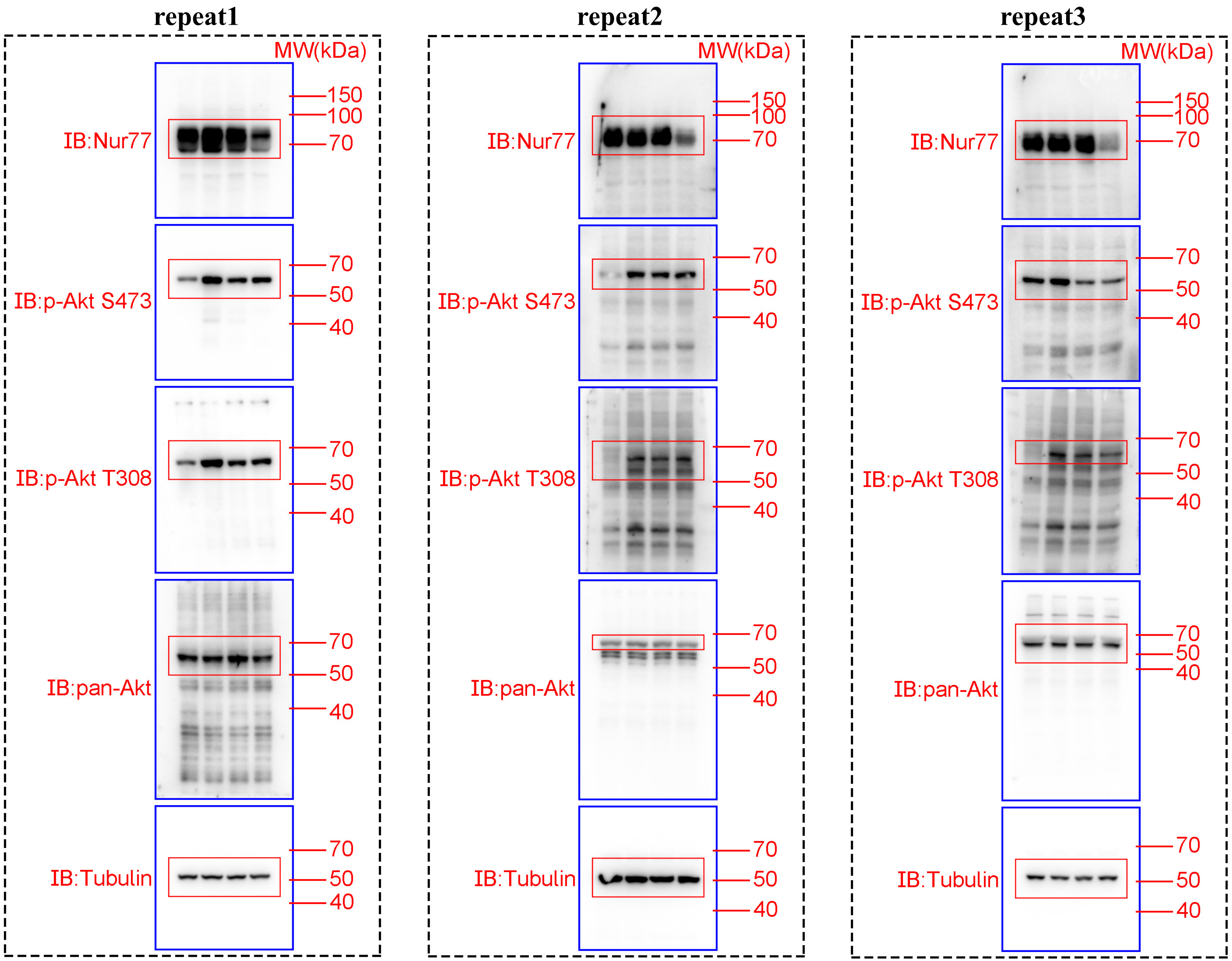
repeat1
repeat2
repeat3
MW(kDa)
IB:Nur77
IB:p-Akt S473
IB:p-Akt T308
IB:pan-Akt
IB:Tubulin
150
100
70
50
40

l

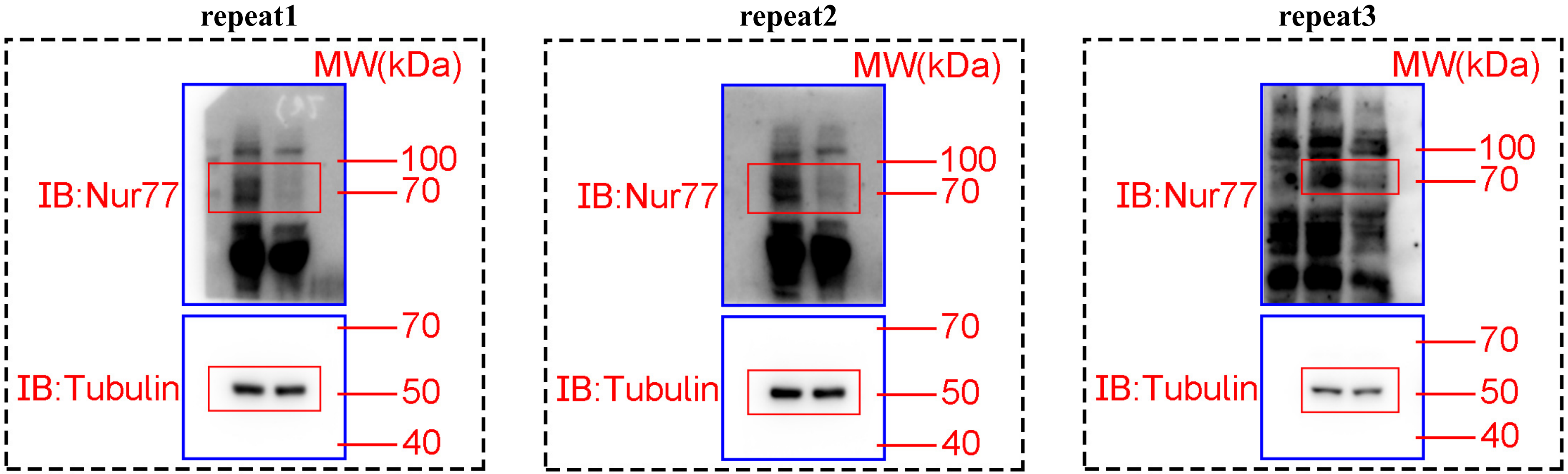
repeat1
MW(kDa)
IB:Nur77
100
70
IB:Tubulin
70
50
40
repeat2
MW(kDa)
IB:Nur77
100
70
IB:Tubulin
70
50
40
repeat3
MW(kDa)
IB:Nur77
100
70
IB:Tubulin
70
50
40

m

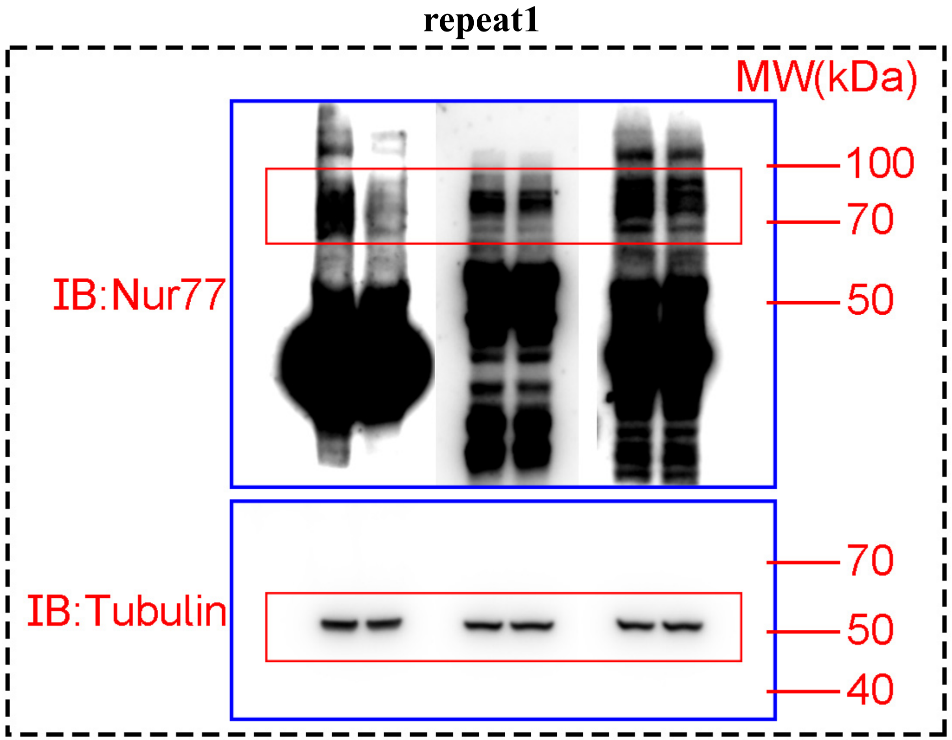
repeat1
MW(kDa)
IB:Nur77
100
70
50
IB:Tubulin
70
50
40

HSC repeat2/3

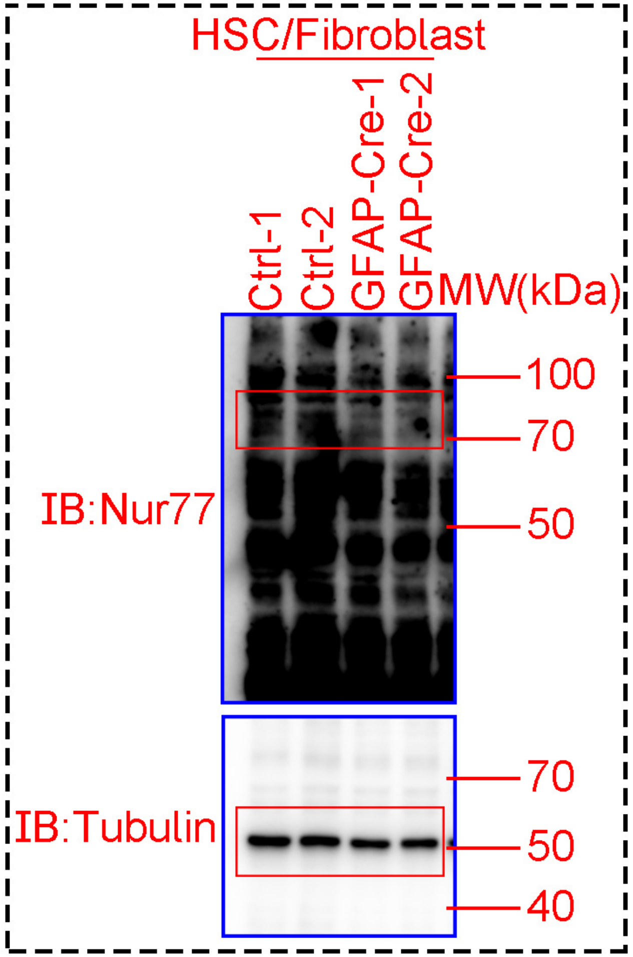

HCC repeat2

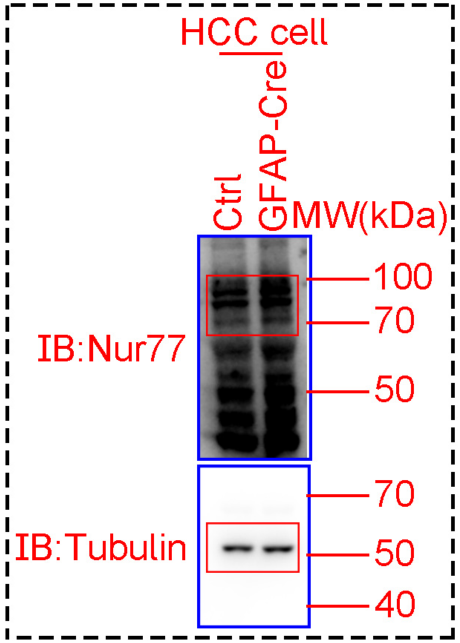

HCC repeat3

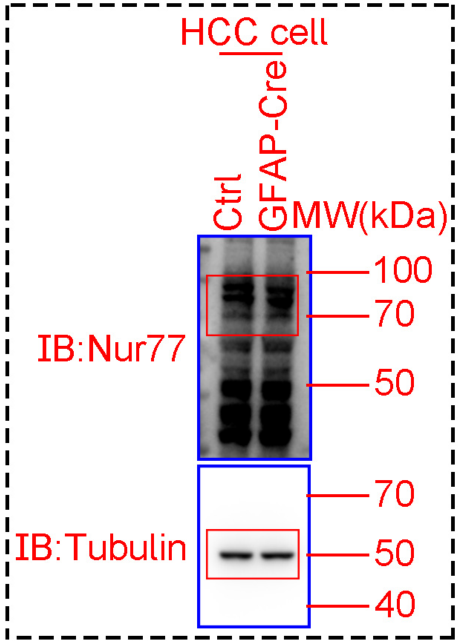

Macrophage repeat2

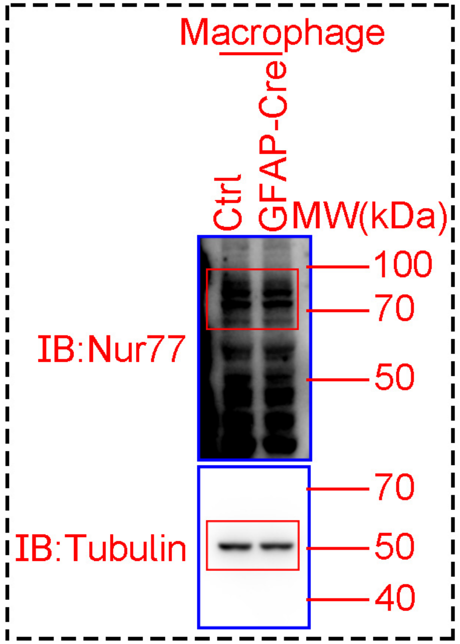

Macrophage repeat3

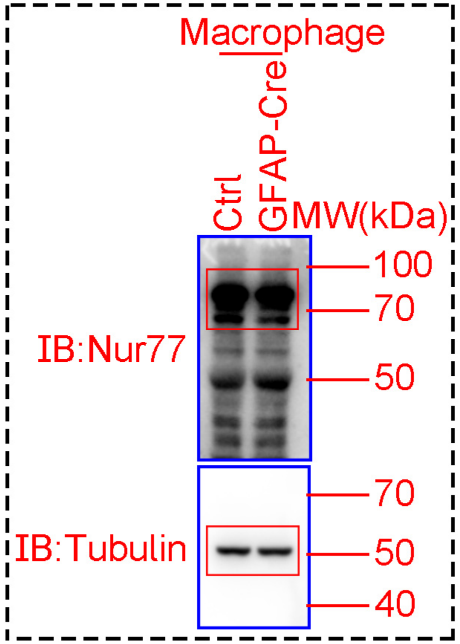

Supplement: Source Data Extended Data Fig. 5 — Unprocessed western blots. [file 42255_2022_642_MOESM25_ESM.pdf]
