# Supplementary material for: HK1 from hepatic stellate cell–derived extracellular vesicles promotes progression of hepatocellular carcinoma
Source: Nat Metab. 2022 Oct 3;4(10):1306–21. doi: 10.1038/s42255-022-00642-5 (PMC9584821; doi:10.1038/s42255-022-00642-5)
Supplement: Source Data Extended Data Fig. 6 — Unprocessed western blots. [file 42255_2022_642_MOESM26_ESM.pdf]

Uncropped western blot images
Extended Data Figure 6

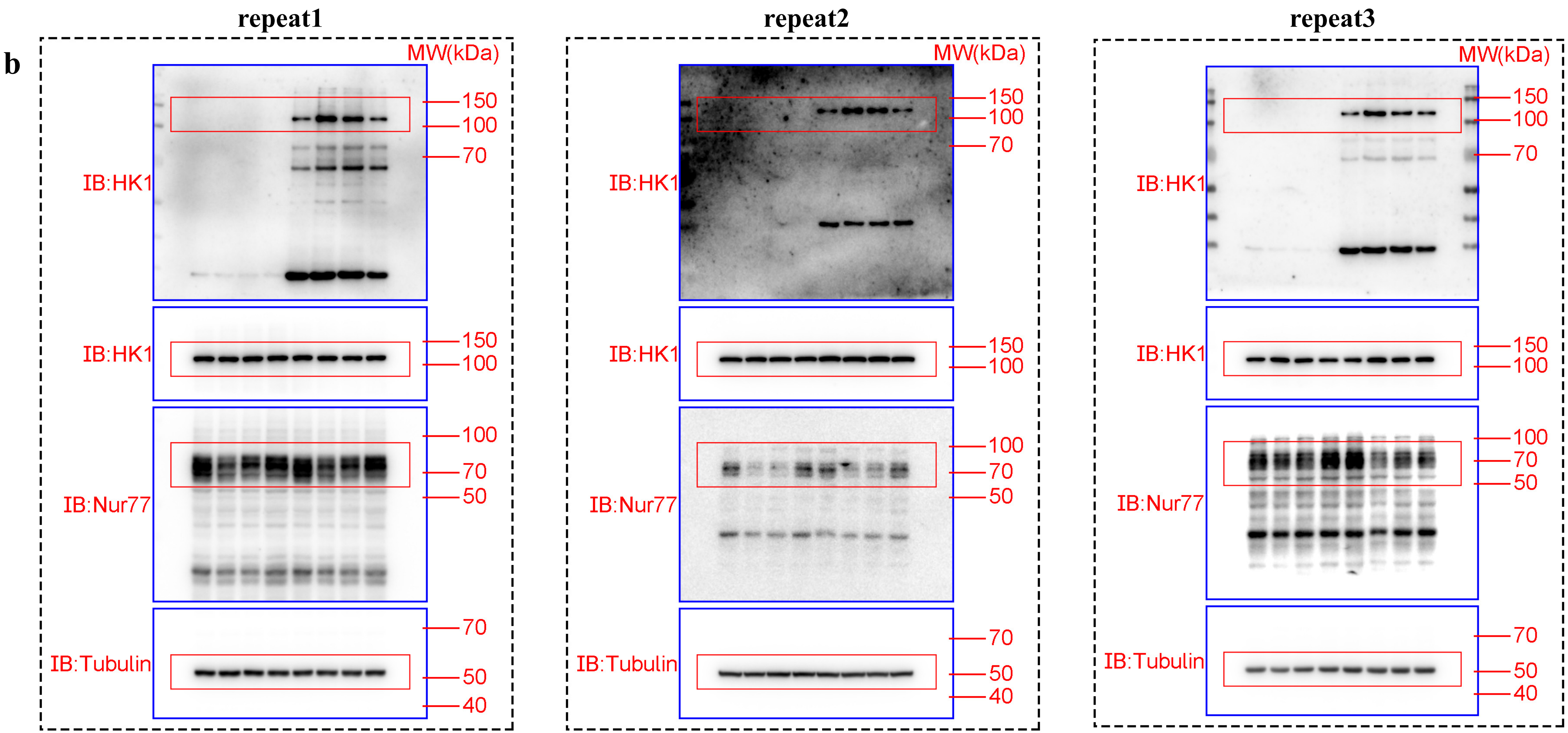

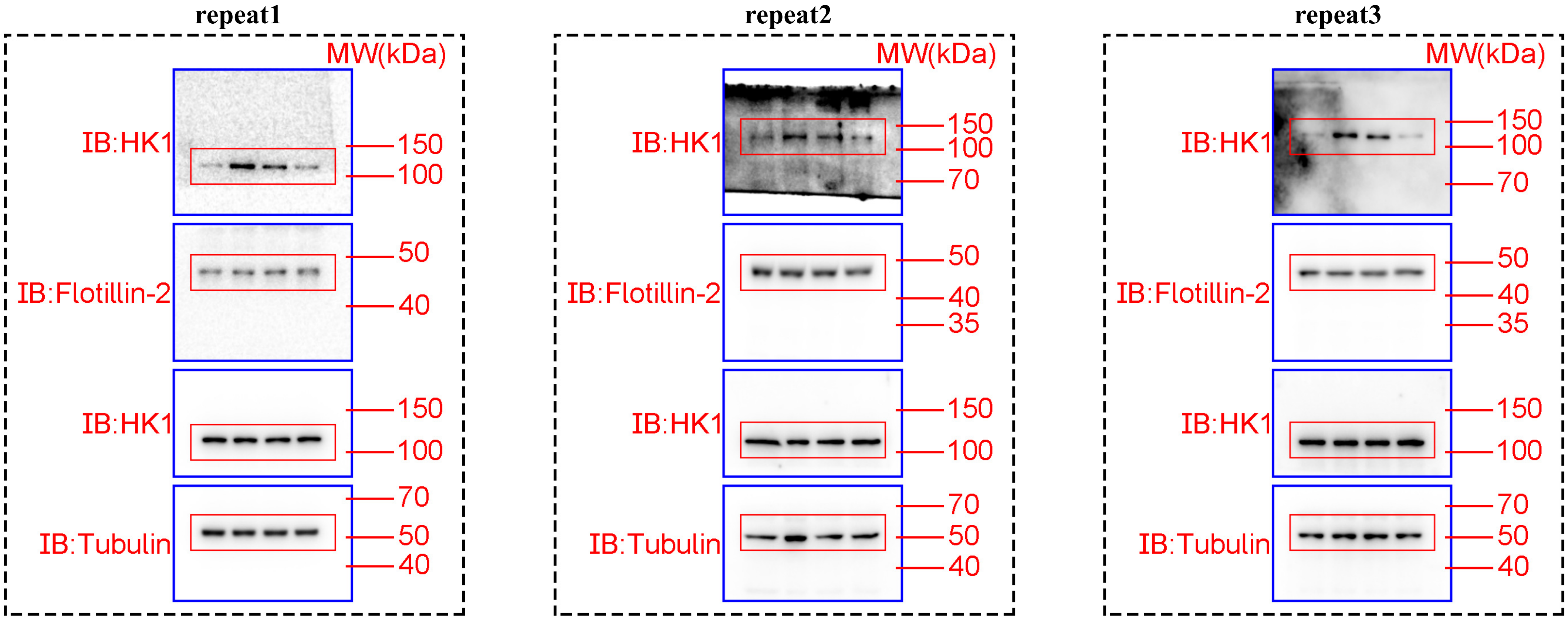
repeat1
repeat2
repeat3
MW(kDa)
IB:HK1
IB:Flotillin-2
IB:HK1
IB:Tubulin
150
100
50
40
150
100
70
50
40
70
35

c

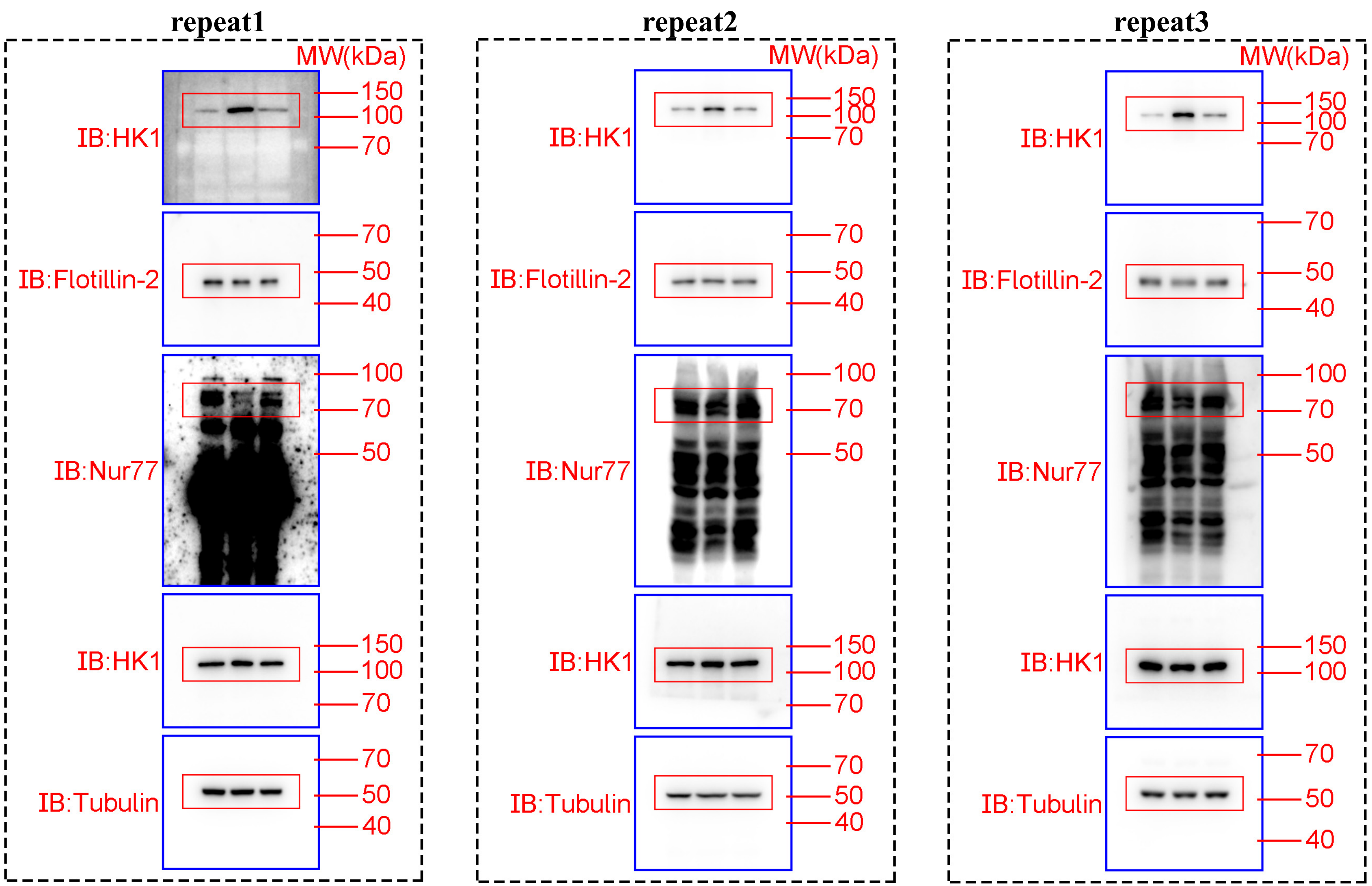
repeat1
repeat2
repeat3
MW(kDa)
IB:HK1
IB:Flotillin-2
IB:Nur77
IB:HK1
IB:Tubulin
150
100
70
50
40

d

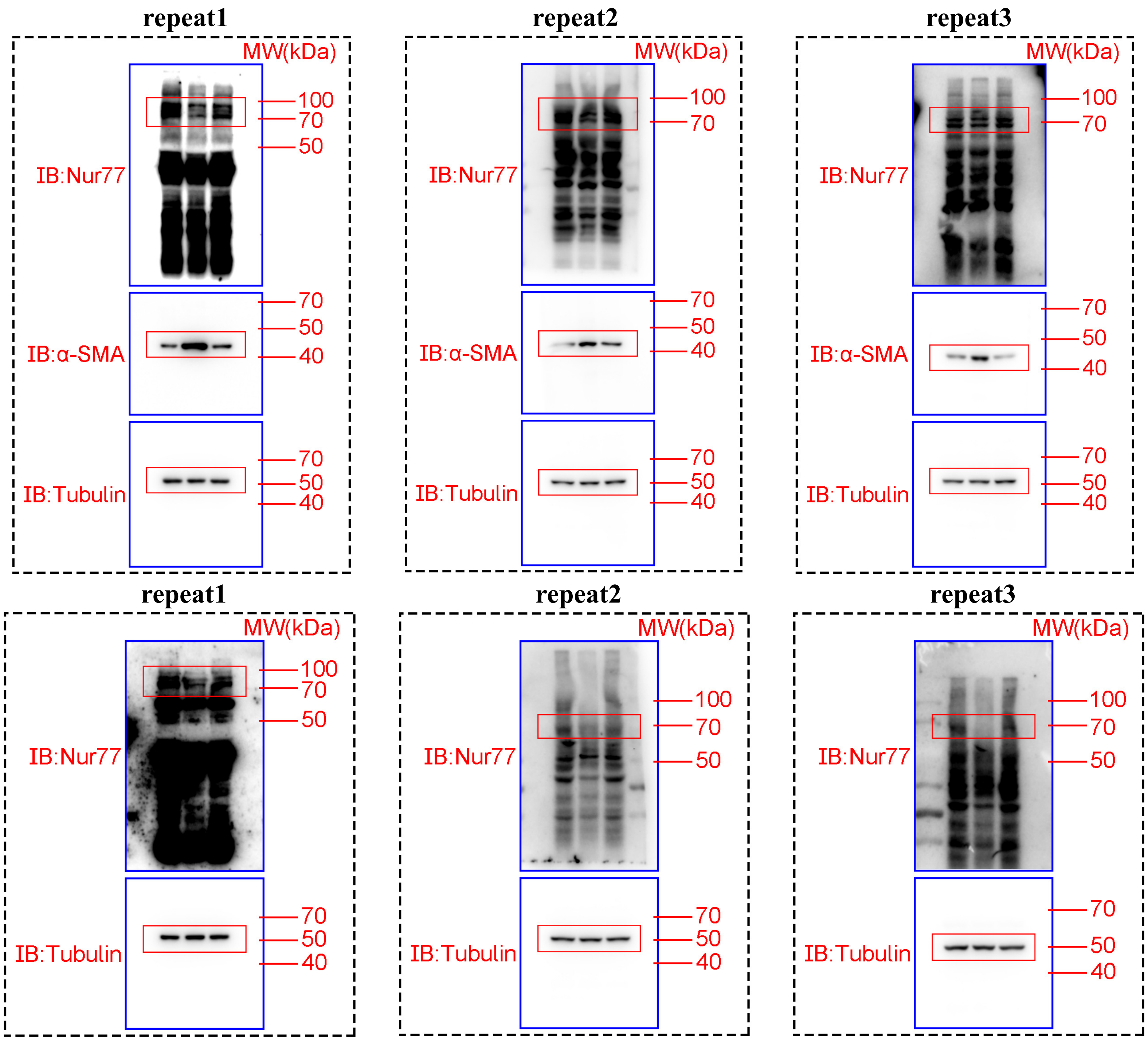

repeat1
repeat2
repeat3
MW(kDa)
IB:Nur77
IB:α-SMA
IB:Tubulin
100
70
50
40

e

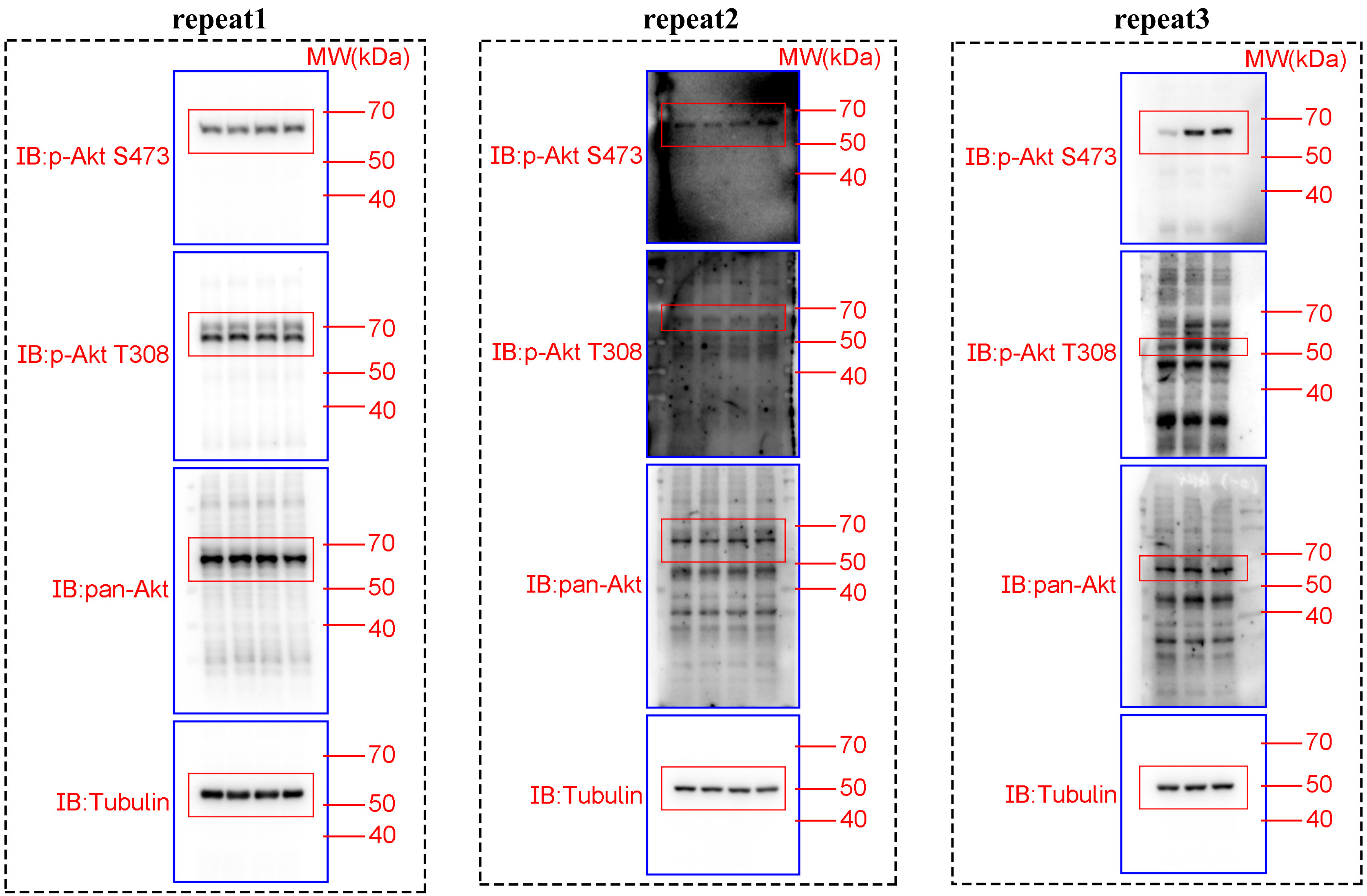

repeat1
repeat2
repeat3
MW(kDa)
IB:p-Akt S473
IB:p-Akt T308
IB:pan-Akt
IB:Tubulin
70
50
40

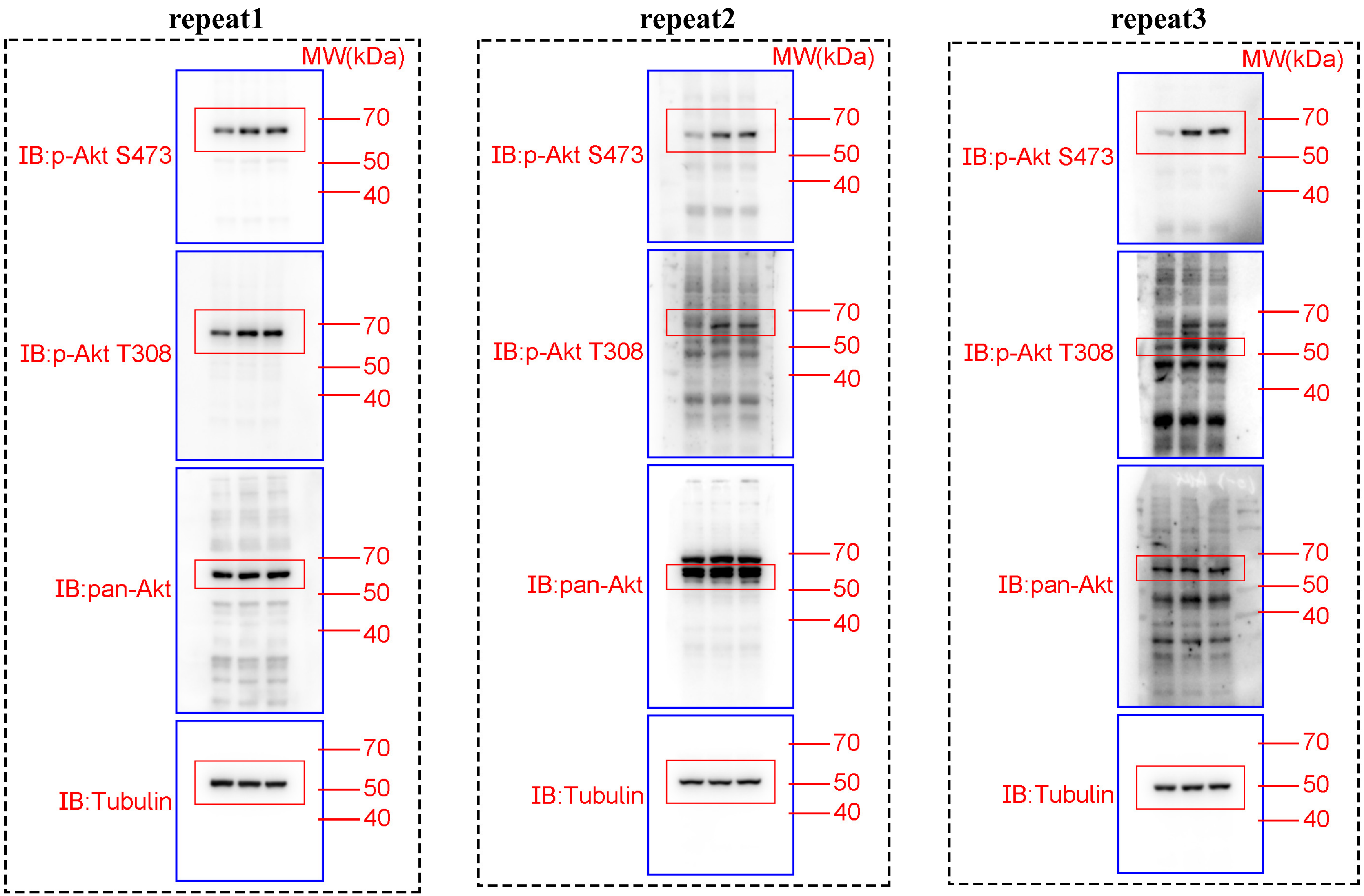

repeat1
repeat2
repeat3
MW(kDa)
IB:p-Akt S473
IB:p-Akt T308
IB:pan-Akt
IB:Tubulin
70
50
40

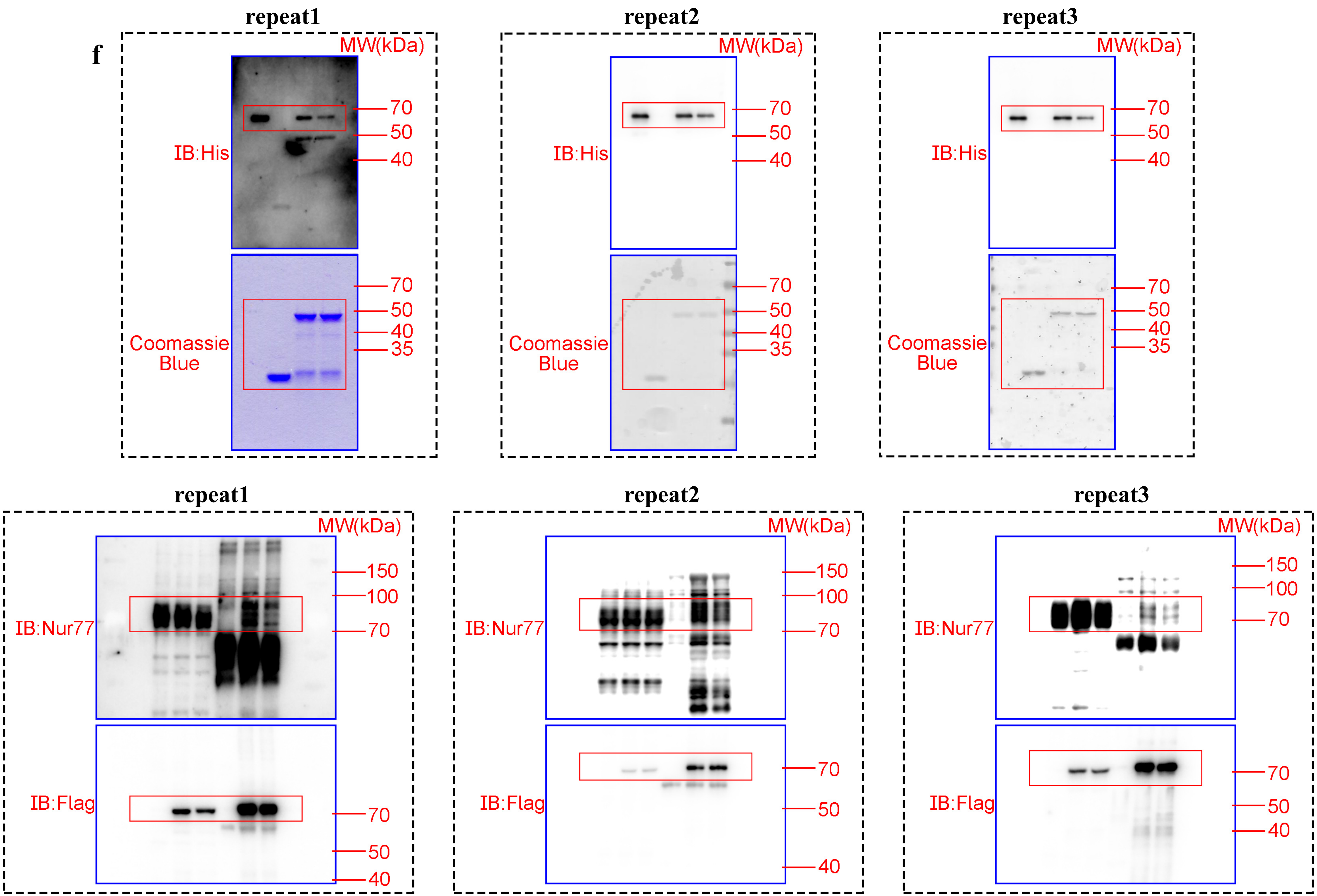
f
repeat1
repeat2
repeat3
MW(kDa)
IB:His
70
50
40
Coomassie Blue
35
IB:Nur77
IB:Flag
150
100
